# Structural Competency: Curriculum for Medical Students, Residents, and Interprofessional Teams on the Structural Factors That Produce Health Disparities

**DOI:** 10.15766/mep_2374-8265.10888

**Published:** 2020-03-13

**Authors:** Joshua Neff, Seth M. Holmes, Kelly R. Knight, Shirley Strong, Ariana Thompson-Lastad, Cara McGuinness, Laura Duncan, Nimish Saxena, Michael J. Harvey, Alice Langford, Katiana L. Carey-Simms, Sara N. Minahan, Shannon Satterwhite, Caitlin Ruppel, Sonia Lee, Lillian Walkover, Jorge De Avila, Brett Lewis, Jenifer Matthews, Nicholas Nelson

**Affiliations:** 1 Resident, Semel Institute for Neuroscience and Human Behavior, University of California, Los Angeles; 2 Associate Professor, Division of Society and Environment, Department of Environmental Science, Policy, and Management, University of California, Berkeley; 3 Associate Professor, Joint Program in Medical Anthropology, University of California, Berkeley; 4 Associate Professor, Department of Anthropology, History and Social Medicine, University of California, San Francisco; 5 Chief Diversity Officer, Samuel Merritt University; 6 Postdoctoral Fellow, Osher Center for Integrative Medicine, University of California, San Francisco; 7 Nurse Midwife, Boston Medical Center; 8 Clinical Instructor, Department of Obstetrics & Gynecology, Boston University School of Medicine; 9 MD/PhD Student in the Medical Scientist Training Program, Department of Anthropology, History and Social Medicine, University of California, San Francisco; 10 Undergraduate Student, University of California, Berkeley; 11 Assistant Professor, Department of Health Science and Recreation, San José State University; 12 Reproductive Health Specialist, Planned Parenthood Northern California; 13 Nurse Midwife, Highland Hospital, Oakland, California; 14 Medical Student in the Medical Scientist Training Program, Department of Anthropology, History and Social Medicine, University of California, San Francisco; 15 Health Policy and Management MPH Student, School of Public Health, University of California, Berkeley; 16 Senior Manager, Health Outreach Partners; 17 Postdoctoral Fellow in Global Health, Department of Sociology, Drexel University; 18 Medical Student, University of Chicago Pritzker School of Medicine; 19 Medical Student, Oregon Health & Science University School of Medicine; 20 Core Faculty, Department of Adolescent Medicine, UCSF Benioff Children's Hospital Oakland; 21 Associate Program Director, Internal Medicine Residency Program, Highland Hospital, Oakland, California

**Keywords:** Structural Competency, Structural Determinants of Health, Social Determinants of Health, Health Disparities, Racism, Structural Violence, Cultural Competency, Cultural Humility, Diversity, Inclusion, Health Equity

## Abstract

**Introduction:**

Research on disparities in health and health care has demonstrated that social, economic, and political factors are key drivers of poor health outcomes. Yet the role of such structural forces on health and health care has been incorporated unevenly into medical training. The framework of structural competency offers a paradigm for training health professionals to recognize and respond to the impact of upstream, structural factors on patient health and health care.

**Methods:**

We report on a brief, interprofessional structural competency curriculum implemented in 32 distinct instances between 2015 and 2017 throughout the San Francisco Bay Area. In consultation with medical and interprofessional education experts, we developed open-ended, written-response surveys to qualitatively evaluate this curriculum's impact on participants. Qualitative data from 15 iterations were analyzed via directed thematic analysis, coding language, and concepts to identify key themes.

**Results:**

Three core themes emerged from analysis of participants' comments. First, participants valued the curriculum's focus on the application of the structural competency framework in real-world clinical, community, and policy contexts. Second, participants with clinical experience (residents, fellows, and faculty) reported that the curriculum helped them reframe how they thought about patients. Third, participants reported feeling reconnected to their original motivations for entering the health professions.

**Discussion:**

This structural competency curriculum fills a gap in health professional education by equipping learners to understand and respond to the role that social, economic, and political structural factors play in patient and community health.

## Educational Objectives

Upon completing this curriculum, trainees will be able to:

1. Identify the influences of structures on patient health.
2. Identify the influences of structures on the clinical encounter.
3. Generate strategies to respond to the influences of structures in the clinic.
4. Generate strategies to respond to the influences of structures beyond the clinic.
5. Describe structural humility as an approach to apply in and beyond the clinic.

## Introduction

Five decades of research on health and health care disparities have shown that social inequalities are key drivers of poor health outcomes among marginalized members of society.^1-6^ Despite this research, the influence of these large-scale influences on health and health care has not been adequately incorporated into medical training.^7-9^ A 2017 report by the Accreditation Council for Graduate Medical Education (ACGME) evaluating 297 ACGME-accredited residency and fellowship programs reported that “there is currently a substantive deficiency in preparing residents and fellows to both identify and address disparities in health care outcomes, as well as ways to minimize or eliminate them.”^10^ A novel curricular approach is needed to address this deficiency—for medical residents and for medical students, attending physicians, other clinicians, and health care staff at all levels.^11-13^

Here, we report on the implementation of a brief structural competency curriculum designed to fill this gap. In this context, *structural* refers to social and economic policies; laws regulating the distribution of health and social resources; and social stratification based on race, ethnicity, religious affiliation, immigration status, ability, gender identity, sexual orientation, and so on. Metzl^14^ initially suggested the idea of structural competency in his 2010 book *The Protest Psychosis: How Schizophrenia Became a Black Disease*. In 2014, Metzl and Hansen formally proposed structural competency as a paradigm for medical education.^15^ The concept has since been taken up and developed in multiple educational settings.^16-19^ Building on Metzl and Hansen's 2014 definition, we define structural competency as the capacity for health professionals to recognize and respond to health and illness as the downstream effects of broad social, political, and economic structures.

As discussed throughout the curriculum described below, structural competency highlights the concepts of structural violence and the naturalization of inequality. Structural violence articulates how unjust social structures lead to bodily and emotional suffering among vulnerable individuals and communities. Expanding on the standard interpersonal connotation of the word *violence*, structural violence underscores that social hierarchies and power structures can similarly cause needless harm to people (i.e., be violent).^20^ The concept of naturalizing inequality refers to the ways in which health disparities are often attributed to the behaviors or innate characteristics of the individuals or groups of people most affected by these disparities.^21^ Such naturalization causes the social origins of health disparities and structural violence to be de-emphasized or overlooked entirely.

Structural competency builds upon existing social determinants of health, cultural competency, and cultural humility curricular efforts.^1,4,5,22-27^ In contrast to many curricula framed in terms of the social determinants of health that describe the existence of, but not the historical and contemporary drivers of, health disparities, structural competency situates these social determinants within a broader structural context. For example, a structural competency approach not only characterizes the epidemiology of racial health disparities—it examines the structures that have created and sustain racial inequity (i.e., structural racism). In contrast to cultural competency, structural competency shifts “attention to forces that influence health outcomes at levels above individual interactions.”^15^ Cultural competency arose in an effort to correct US medicine's blind Eurocentrism. However, many cultural competency curricula over time have reduced culture to a list-of-traits form of stereotyping.^22^ More broadly, as Gregg and Saha^23^ note, “in attempting to address *racial and ethnic* disparities in care through *cultural* competence training, educators too often conflate these distinct concepts” (emphasis in original). In recognition of the critique of cultural competency articulated by Tervalon and Murray-García^28^ in their framing of cultural humility, Metzl and Hansen^15^ identified structural humility as one of the core components of structural competency. All of these themes are discussed further in the curriculum.

The Structural Competency Working Group (SCWG), based in Oakland, California, is an interprofessional group of practicing clinicians, scholars, students, and administrators from a broad range of fields, including medicine, nursing, anthropology, sociology, social work, and public health, among others. The goal of the group, of which all this resource's authors have been members, is to integrate structural competency into the training and practice of health care providers. Since fall 2014, members of the SCWG have met biweekly both to discuss relevant literature on topics ranging from the medical social sciences and social epidemiology to critical pedagogy and effective facilitation (Appendix G) and to develop and refine structural competency curricula informed by those readings.

The SCWG began developing a stand-alone, 3-hour structural competency curriculum—which we sometimes refer to as a training—in fall 2014 and piloted this training in 2015 with a cohort of 12 residents in a family residency program.^29^ This was the first published example of well-developed structural competency training. Facilitators of this pilot effort were prepared through group discussions and practice training sessions. Based on the experience and evaluation of the pilot training, the curriculum was subsequently amended—adding an expanded focus on responding to harmful social structures at various levels, from individual patient encounters and clinic programs to research and policy. We developed the training to include a variety of instructional approaches within each section, which we refer to as modules. The first two modules include cases, discussion, and arrow diagrams combined with direct didactics with definitions of terms to provide trainees with shared frameworks and vocabulary. The third module includes multiple examples of responses to harmful social structures followed by a brainstorming exercise designed to inspire action at various scales among participants. Each module includes reflective segments to encourage trainees to apply the learning to their own experience thus far and to their intentions moving forward.

In our initial efforts, we focused on structural competency for physicians and physicians-in-training. However, we quickly expanded our target audience to include all persons working in health-related fields. As of October 2019, our group has run this training more than 75 times. Groups that have participated in our trainings include but are not limited to medical students in every year of medical school, in both large lectures and small groups; primary care and categorical internal medicine residents; family medicine residents and faculty; psychiatry residents and faculty; prenursing and premedicine students; multidisciplinary global health fellows from medicine, nursing, and social work; nurse-midwife students and faculty; social welfare graduate students; interprofessional teams including faculty and trainees from medicine, nursing, physician assistants, physical therapy, occupational therapy, and nurse-midwives; public health students and faculty; physical therapy students; nursing students and faculty; and global health master's students. When offering this training to multidisciplinary teams, we emphasize communication among all team members, the value of respecting the expertise of all members of the team, and the potential for structural competency to provide a basis for deeper interprofessional collaboration.^30^ As *MedEdPORTAL* is a source specifically for medical education, we focus on our trainings with medical students, residents, and practicing physicians.

We have found that the training can be effective in both large lecture halls and small seminar rooms with as few as 10 people, given the mix of didactic, individual exercise, and discussion components. In lecture hall settings, much of the participation occurs in pairs and small groups; in smaller classroom settings, all participants can share and discuss together. The version of the training presented here is a 4-hour session. By scaling back various exercises and discussion and reflection components, we have condensed this material into 3 hours; however, we recommend following the full 4-hour footprint described to maximize opportunities for participant reflection and engagement.

Although structural competency is a new framework in health care, several medical schools and residency programs around the United States and internationally have begun formally incorporating it into their curricula. A review of all medical schools and training programs implementing structural competency trainings is beyond the scope of this publication, although several authors of this work are currently undertaking a review of the extent to which health disparities are discussed in structural terms within medical school curricula. However, the SCWG has been consulted increasingly frequently in recent years by various institutions for assistance in developing and implementing structural competency curricula. We are thrilled for our training materials to be available to the medical education community at large through *MedEdPORTAL.* It is our hope that those interested in establishing or further developing structural competency curricula at their institutions find these resources helpful.

This work builds on prior publications in *MedEdPORTAL.* Brooks, Rougas, and George^31^ describe their efforts to develop a 1-hour structural competency–informed session for medical students that highlights the role of structural racism, interpersonal racial bias, and blaming the victim in exacerbating racial health disparities. Our work describes a more intensive (although potentially complementary) structural competency training that has been implemented for a wide range of medical professionals and trainees across numerous sites. More generally, our submission contributes to published literature within *MedEdPORTAL* addressing the relationship among social structures, equity, and health.^32-36^

In summary, structural competency seeks to fill a crucial gap in medical training and practice. The training described here, the materials for which are included in the appendices, is a half-day session that we have found to be effective for introducing structural competency to a wide variety of health care providers, including but not limited to physicians and physicians-in-training.

## Methods

### Curricular Context

We implemented this curriculum, revised in response to feedback from the pilot training,^29^ in 32 distinct instances between 2015 and 2017. These sessions took place in multiple contexts with diverse target learners. Those participating included physicians and physicians-in-training at various stages: medical students (MS1, 2, and 4), primary care and categorical-track internal medicine residents, family medicine residents and faculty, and global health fellows. We conducted the training with interprofessional teams including community health center staff (physicians, nurse practitioners, registered nurses, and medical assistants) and additionally trained students and faculty from nursing, physical therapy, occupational therapy, midwifery, and physician assistant programs. We also ran the training for various other single-profession audiences. No prerequisite knowledge was expected of learners.

### Implementation

In each of the preceding settings, all participants were physically present for the duration of the trainings. According to the availability of curricular time at host institutions, trainings have been between 3 and 4 hours in length (with greater time for interactive portions of the training in longer iterations). The trainings typically were led by two to three facilitators from diverse professional backgrounds, including physicians, social scientists, graduate and MD/PhD or MD/MS students studying social sciences, health professional educators, and administrators, among others. Those who did not facilitate the pilot session prepared for this role by observing the training and by reading preliminary versions of the training manual (Appendices A–F, N, and O) and the core background articles listed in the training manual (Appendix B, p. 4; Appendix C, pp. 4–5; Appendix D, pp. 4–5; Appendix E, p. 5; and compiled in Appendix G). The training was implemented in sequential modules as outlined below and explained in depth in the training manual (Appendices A–F, N, and O) with the associated slide deck (Appendices H–K) shown via a projector in the room. Additional materials included handouts (Appendix L), a participant sign-in sheet (Appendix P), and a whiteboard and/or flip chart for discussion notes.

As our group has grown, we have developed a yearlong apprenticeship to prepare new SCWG members to facilitate our trainings. This apprenticeship includes (a) reading articles and chapters in the syllabus (Appendix G) and completing written reflections about and participating in discussions of readings at biweekly meetings; (b) assisting established SCWG trainers in the facilitation of a minimum of three structural competency trainings, after each of which established trainers provide apprentices with verbal and written feedback; and (c) undergoing a review of reading reflections and written feedback from SCWG leadership to determine readiness to facilitate independently. Once the preceding have been completed, members graduate to full-scope trainers able to independently facilitate SCWG training sessions.

To test and refine our facilitator training manual (Appendices A–G), we piloted preliminary versions of our training materials with several organizations and individuals. We requested that presenters follow preparation instructions described in Appendices A–G and provide us with feedback about their experience. These pilots are not part of the evaluation formally presented here, but they did help us further develop and revise the material attached.

The training proceeds generally according to the following outline. In various instances, it has had minor modifications to be slightly shortened (to 3 hours) or lengthened (to 4 hours), depending on the curricular space available at the various institutions in which we have conducted this session.

Recommended Training Agenda (Time: 4 Hours)

• Welcome and introduction (15 minutes):
  ○ Facilitator and participant introductions.
  ○ Training overview and positionality.
  ○ Agenda and group agreements.
• Module 1: Structures and Health (100 minutes): This module discusses the social structures that influence health, then introduces and defines the concepts of structural violence and naturalizing inequality.
  ○ Section 1: Social Structures and Health (35 minutes):
    ■ Define structures (Appendix L, p. 3).
    ■ Review epidemiology illustrating the distribution of health disparities.
    ■ Patient case: exercise—participants read chart note and discuss patient case (Appendix L, p. 1); arrow diagram 1—discussion of this patient's life trajectory and structural influences on this trajectory (Appendix I, slides 11–14).
  ○ Section 2: Structural Violence and Structural Vulnerability (25 minutes):
    ■ Definition and discussion of concept: illustration of concept via examples of structural racism (mass incarceration; Appendix I, slides 15–22), definition of structural vulnerability and intersectionality (Appendix I, slides 23–25; Appendix L, p. 3).
    ■ Reflection: participants reflect on, write, and discuss influence of structural violence/vulnerability in cases from their clinical or personal experience (Appendix I, slide 26; Appendix L, p. 6).
  ○ Section 3: Naturalizing Inequality (40 minutes):
    ■ Definition/discussion of concept, including implicit frameworks (Appendix I, slide 30; Appendix L, p. 4).
    ■ Exercise: participants read passage, identifying the implicit frameworks/naturalization of inequality (Appendix I, slides 27–37; Appendix L, p. 7).
    ■ Examples of naturalizing inequality in health literature/practice (Appendix I, slides 38–39).
• Break (10 minutes).
• Module 2: The Origins of Structural Competency (45 minutes): This module addresses the relationship of structural competency to cultural competency and humility and the social determinants of health, then discusses why structural competency is important for health care providers to learn.
  ○ Section 1: Cultural Competency and Cultural Humility (5 minutes):
    ■ Intentions and limitations (Appendix J, slides 2–7).
  ○ Section 2: Structural Competency and Structural Humility (20 minutes):
    ■ Motivation behind and definition of structural competency (Appendix J, slide 8; Appendix L, p. 9).
    ■ Five goals of structural competency (Appendix J, slide 9).
    ■ Naming the framework (Appendix J, slide 11).
    ■ Relationship of structural competency to social determinants of health (Appendix J, slide 12).
  ○ Section 3: Why Is Structural Competency Important for Providers to Learn? (20 minutes):
    ■ The importance of structural competency to improve provider performance and patient outcomes and to empower providers to advocate for change (Appendix J, slides 13–17).
    ■ Flint, Michigan, example of provider advocacy (Appendix J, slide 16).
    ■ The structural influences on the practice of health care reflection exercise: what structures were influencing the provider in the patient case reviewed in module 1 (Appendix J, slides 19–20)?
    ■ Arrow diagram 2: presentation of provider's personal trajectory and major structural influences (Appendix J, slide 20).
    ■ Trainees reflect upon and discuss structural influences on their own practice (Appendix J, slides 21–22; Appendix L, p. 8).
• Module 3: Responding to Harmful Structures in and Beyond the Clinic (60 minutes): This module explores ways of responding to harmful social structures by providing examples of such responses, discussing the various levels at which providers can intervene, and prompting participants to brainstorm strategies.
  ○ Section 1: Structurally Competent Interventions (15 minutes):
    ■ Examples of structural responses to disparities in health (Appendix K, slides 3–4).
  ○ Section 2: Levels of Intervention (5 minutes):
    ■ Introduce levels: individual, interpersonal, clinic/institutional, community, policy, and research (Appendix K, slides 5–6; Appendix L, pp. 10–12).
  ○ Section 3: Imagining Structural Interventions (25 minutes):
    ■ Trainees brainstorm and discuss strategies for responding to harmful structural influences at various levels (Appendix K, slide 7).
    ■ Trainees reflect and write individually on their intentions (Appendix K, slide 15; Appendix L, p. 8).
  ○ Section 4: Beloved Community (5 minutes):
    ■ Introduce the concept of beloved community and explain its importance for structurally competent practice (Appendix K, slides 8–13).
  ○ Section 5: Putting Theory Into Practice (10 minutes):
    ■ Trainees identify at least one intervention strategy to implement to address structural causes of ill health (Appendix K, slides 14–15).
• Conclusion and evaluation (10 minutes).

### Evaluation Strategy

Our evaluation strategy included both process and knowledge assessment components consistent with the learning objectives of the training. After consulting with medical and interprofessional education experts, we developed qualitative evaluations for this training including a postsession survey that was piloted with resident trainees and revised. The survey (Appendix M) included open-ended, written-response questions to gain feedback on the training's effectiveness in general and in relation to the stated learning objectives of the curriculum. General process evaluation questions included “Please share your candid thoughts on this training: What parts worked well? What parts did you like? What should we change? How could we make this training more effective?” Questions related to the stated curricular learning objectives included the following:

• “Which elements of today's training did you find most valuable? Any tools/concepts/strategies that you found particularly useful?”
• “In what ways if any do you expect these frameworks to be useful to you in the coming weeks and months, or in your career/training in the longer term?”
• “Please briefly summarize your understanding of the following terms: (a) structural violence, (b) naturalizing inequality, and (c) structural competency.”

Participants completed the evaluation survey (Appendix M) upon completion of the half-end curriculum in each of the preceding settings.

## Results

We evaluated trainings with postsession, written-response surveys (Appendix M) administered immediately following the training. In such instances, all trainees completed the surveys (response rate: 100%). We conducted qualitative data analysis via directed thematic analysis,^37^ coding recurrent language and concepts to identify key themes. Evaluation was deemed exempt by the University of California, San Francisco, Committee on Human Research, IRB#15-16392.

Here, we focus on the data from physician and medical student participants (*N* = 275). Three primary themes emerged from the evaluation of these data. These themes, with representative quotes, include the following:

1. Participants valued the training's focus on application of the structural competency framework in real-world clinical, community, and policy contexts.
  • Medical student: “I really liked the concrete examples and strategies that were presented. It made the concepts and theories more applicable and tangible for me to grasp.”
  • Medical student: “I appreciated the time to discuss and think about responding to challenges within and across different levels.”
  • Resident: “Engaging Session! Appreciate that the training was grounded in clinical case studies and felt very applied. And loved the mix of lecture/presentation and small group discussion/activities.”
  • Fellow: “[The training] further inspires me to be an advocate for policy changes that can improve lives of patients.”
2. Participants with clinical experience (residents, fellows, and faculty) reported that the training helped them reframe how they think about patients, away from blaming and other possible misconceptions.
  • Resident: “Having a vocabulary for these structures helps to reframe clinical discussions from blaming [patients for poor health outcomes] to acknowledging [structural influences on illness].”
  • Fellow: “Recognizing the role of implicit frameworks in day to day work would reduce misconceptions in so many areas.”
  • Fellow: “[In the coming weeks and months, I plan to take] specific steps to change the interaction between myself, patients and staff to be more deliberate in stopping the naturalization of inequality.”
  • Faculty: “[The training] was an affirmation to not let the negative teachings of blaming the patient sneak into my thinking as well as an affirmation that taking the time to talk to patients about their social situation is an important use of time.”
3. Participants reported feeling reconnected to their original motivations for entering the health professions.
  • Medical student: “This session reminded me of the reasons I came to med school in the first place.”
  • Resident: “Many of the concepts presented were already familiar to me but I realize I have strayed from them in many of my clinical encounters. I went into medicine with the intention of contributing to systemic change within a broken system and it feels good to be discussing this in this forum.”
  • Faculty: “[This training] will absolutely help me to be more empathetic, understanding, accepting, and humble. It reminds me why I have chosen to work with [the] socioeconomically disadvantaged.”

Overall, medical students frequently discussed anticipatory hopes and anxieties for future practice, whereas residents and faculty generally reflected on prior clinical experience and challenges, as well as contemplating practical strategies for shifting current practice.

## Discussion

This curriculum, adapted after a pilot phase,^29^ represents a novel attempt to develop and disseminate a curriculum using a structural competency framework for medical students, residents, and other health professional trainees. Today, many medical students are entering their education and training with a stated desire for curricula that incorporate a structural analysis of health and health care disparities.^11,38-40^ Among all health professionals, there is a growing need for knowledge and skills that address the complex medical management of vulnerable populations with specific attention to interprofessional teams and systems-based learning. This structural competency training is designed to be responsive to each of these concerns.

This curriculum offers several modules consistent with the recommendations from the ACGME Clinical Learning Environment Review (CLER) Health Care Quality Pathway 5, “Resident/fellow and faculty member education on reducing health care disparities.” Specifically, the CLER recommends that residents/fellows and faculty members should “receive education on identifying and reducing health care disparities.”^41^ The curriculum outlines a process for developing actionable plans responsive to structurally driven health and health care disparities that could inform the CLER recommendation for “QI (quality improvement) activities addressing health care disparities for the vulnerable populations.”^41^

As noted earlier, evaluation of participants and subsequent analysis yielded three core themes. First, many participants commented that they appreciated components of the training focused on developing responses to the impact of structural violence on patient health at a variety of levels, from intrapersonal to policy. After the pilot of this training,^29^ we received participant feedback expressing feelings of being overwhelmed and uncertain of what to do next. In response to this feedback, we redeveloped Module 3 of the training (described above), which addresses the application of structural competency in and beyond clinical contexts. Findings from our evaluation of subsequent sessions suggest that these modifications helped participants feel more empowered and less distressed upon completing the training.

Second, participants in our training frequently reported that it led them to reframe their thinking about patients, away from blaming and other possible misconceptions. In this regard, participants found the section discussing the implicit frameworks that naturalize inequality to be particularly useful. In this section of Module 1 (Appendix C, p. 17), the training highlights how focusing primarily or exclusively on behavioral, cultural, or genetic explanations for health disparities can result in overlooking the structural influences on health—and thereby lead providers to inadvertently blame patients for the harms caused by structural violence.^23,42^ Participants did not generally state what they were moving toward in shifting away from blaming. Further research can explore how best to describe participants' shift in perspective following structural competency training.

Third, several participants expressed that the training reconnected them to their original motivations to pursue a career in health care. Comments such as those included above were volunteered without any questions prompting participants to reflect on this topic.

Two topics that often arise in discussions of structural competency trainings are their potential effects on providers' empathy for patients and on provider burnout.^29^ The shift in perspective away from blaming patients described in our second theme could plausibly promote provider empathy toward structurally vulnerable patients. Our third theme suggests that structural competency may help connect at least some physicians and medical students to a sense of meaning in their work and training. As burnout has been found to be inversely correlated with both empathy for patients^43-45^ and a sense of meaning or purpose in one's work,^43,46^ could structural competency training help reduce physician burnout? Furthermore, it has been argued that collective action to address the structural roots of the issues afflicting patients and providers—as structural competency encourages—can help address burnout.^47^ Further research is needed to explore the extent and durability of these possible effects of structural competency curricula on participants at various stages of training.

This evaluation has several limitations. Standardized measures of such curricular content have not yet been developed or validated with similar populations of learners. Recent evaluative data on structural competency have been collected for prehealth undergraduate education^48,49^ but not among health professionals—licensed or in training. Not all institutional and clinical settings have access to interdisciplinary teams like those that have facilitated our sessions, although we have attempted to mitigate this issue through our preparation of the training manual (Appendices A–F) and other supplementary materials for facilitators (Appendices G, N, and O). Further assessment and evaluation are needed to understand the potential drawbacks and benefits of working with interprofessional versus single-profession trainee audiences.

Based on these findings, we envision multiple future directions for this work. Structural competency requires further process and outcome evaluation research—including, as noted above, research into the impact of structural competency training on providers' well-being and providers' sense of their motivation and ability to help address the structural drivers of their patients' illnesses. In addition, participants have expressed the desire for further engagement with and integration of structural competency throughout their professional training. This requires the development of further iterative training materials. Finally, we see the potential to make structural competency training more impactful by tailoring it to the needs of specific groups of learners. Toward this end, we have begun adapting our training materials to meet the needs of diverse groups of health care professionals. Currently, we are developing versions of our training tailored to mental health providers, reproductive health providers, nursing students, and prehealth undergraduate students. Each of these adaptations includes the development of new case examples highlighting clinical and structural issues most pertinent to the intended audience—for example, a perinatal case for the reproductive health adaptation.

The implications of structural competency training are potentially far reaching. The impact of the social determinants of health on patient illness is widely acknowledged within the health professions, yet health disparities are not consistently contextualized within broader social, political, and economic structures. Furthermore, it is rare that providers are encouraged to explore how they might act to address those structures to improve patient health. Our above-described efforts to develop, implement, and evaluate a structural competency curriculum with several different audiences suggest that structural competency can begin to fill these gaps in the training and practice of physicians and other health professionals.

## Appendices

A. Manual Background Info.docx
B. Manual Intro.docx
C. Manual Module 1.docx
D. Manual Module 2.docx
E. Manual Module 3.docx
F. Manual Conclusion and Evaluation.docx
G. Supplemental Reading List.docx
H. Training Slides Intro.pptx
I. Training Slides Module 1.pptx
J. Training Slides Module 2.pptx
K. Training Slides Module 3.pptx
L. Participant Workbook.pdf
M. Posttraining Survey.pdf
N. Facilitator Guidelines.docx
O. Facilitator Preparation - Terms and Concepts.docx
P. Participant Sign-in Sheet.docx All appendices are peer reviewed as integral parts of the Original Publication.
