# Supplementary material for: Structural Competency: Curriculum for Medical Students, Residents, and Interprofessional Teams on the Structural Factors That Produce Health Disparities
Source: MedEdPORTAL. 2020 Mar 13;16:10888. doi: 10.15766/mep_2374-8265.10888 (PMC7182045; doi:10.15766/mep_2374-8265.10888)
Supplement: Supplementary file 1 — A. Manual Background Info.docx B. Manual Intro.docx C. Manual Module 1.docx D. Manual Module 2.docx E. Manual Module 3.docx F. Manual Conclusion and Evaluation.docx G. Supplemental Reading List.docx H. Training Slides Intro.pptx I. Training Slides Module 1.pptx J. Training Slides Module 2.pptx K. Training Slides Module 3.pptx L. Participant Workbook.pdf M. Posttraining Survey.pdf N. Facilitator Guidelines.docx O. Facilitator Preparation - Terms and Concepts.docx P. Participant Sign-in Sheet.docx [file mep-16-10888-s001.zip › A. Manual Background Info.docx]

STRUCTURAL COMPETENCY:

A Framework for Recognizing & Responding to Social, Political & Economic Structures to Improve Health


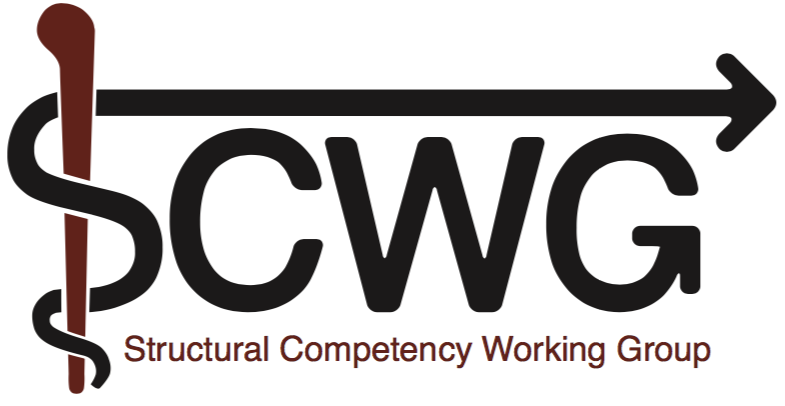


TRAINING CURRICULUM: BACKGROUND INFORMATION

Updated September 2018

SCWG logo: Author-owned.

Copyright:

The Structural Competency Working Group ([www.structcomp.org](http://www.structcomp.org)), in order to promote the widest possible dissemination of health curricula, has adopted an open copyright policy for its structural competency training materials. This means that we will grant permission to translate, adapt, or borrow our materials without charging fees or royalties under the following conditions:

- that you credit the Structural Competency Working Group for any borrowed/adapted Working Group materials and inform your audience about who we are and how to learn more about our organization ([structcomp.org](http://structcomp.org)). We suggest the following attribution (and request that it appear on any copyright page):
  - “*These materials have been [borrowed][adapted] from the Structural Competency Working Group,* [*www.structuralcompetency.org*](http://www.structuralcompetency.org)*; you can contact the Group at* [*structuralcompetency@gmail.com*](mailto:structuralcompetency@gmail.com)*”;*
- that your materials are distributed at no cost (or for your production cost only), that is, not-for-profit;
- that you allow others to reproduce/ adapt your edition or adaptation with no fees, royalties, etc. so long as they also do so at no cost or for production cost only, that is, not-for-profit;
- that you provide us with digital versions of your materials (including PDF and/or Microsoft Word files) and work with us so that we can host it on our website;
- that you send us your contact information so we can post it on our website and provide it to people who want to contact you about your edition, adaptation or publication;
- that you contact and stay in touch with the Structural Competency Working Group so that we can learn about your project and make sure you are using the most up-to-date materials.

If you decide to begin translating, adapting, or borrowing our materials, please use the most recently updated versions. Please contact us at [structuralcompetency@gmail.com](mailto:structuralcompetency@gmail.com) to find out if we are currently working on updating our materials, and if anyone else is working on a project similar to your own.

This training curriculum was prepared by Josh Neff, Seth M. Holmes, Kelly R. Knight, Shirley Strong, Ariana Thompson-Lastad, Cara McGuinness, Laura Duncan, Michael J. Harvey, Nimish Saxena, Katiana L. Carey-Simms, Alice Langford, Sara Minahan, Shannon Satterwhite, Lillian Walkover, Jorge De Avila, Brett Lewis, Gregory Chin, Jenifer Matthews, and Nick Nelson of the Structural Competency Working Group ([structcomp.org](http://structcomp.org/)), in collaboration with Sonia Lee and Caitlin Ruppel of Health Outreach Partners ([outreach-partners.org](http://outreach-partners.org/)).

The Structural Competency Working Group’s efforts have been supported by the Berkeley Center for Social Medicine and Deborah Lustig as well as the University of California Humanities Research Institute. Josh Neff’s work on this project has been supported by the UCSF Resource Allocation Program for Trainees (RAPtr), the Greater Good Science Center Hornaday Fellowship, UC Berkeley-UCSF Joint Medical Program Thesis Grant, and the Helen Marguerite Schoeneman Scholarship.

Table of Contents

Training Preface 5

Information for Facilitators 7

Sample Training Agendas 9

Training Preface

About the Curriculum

To promote health equity and provide appropriate patient care, it is imperative that health care providers serving vulnerable and underserved communities be aware of the factors that influence the health of their patients. Structural competency training is designed to strengthen the capacity of health care providers to recognize and respond to patient health and illness as the product of broad social, political, and economic structures. This curriculum expands upon themes sometimes discussed under the rubric of cultural competency, cultural humility, and the social determinants of health. Structural competency encourages providers to respond to structural issues at various levels, from individual interactions with patients to broader social and political interventions.

Structural Competency Working Group

Located in the San Francisco Bay Area of California, the Structural Competency Working Group (SCWG) is a collective of clinicians, researchers, public health professionals, students, educators, and other community members. The goal of the SCWG is to promote the training of health professionals in structural competency. The SCWG develops and disseminates open-use structural competency curricula. We have conducted trainings with students and practitioners of medicine, nursing, social work, and public health, among other disciplines.

Intended Audience

While the concepts included in this training curriculum could apply to many health-related organizations, it was initially developed specifically for clinicians and clinical trainees. It has since expanded and is now relevant to multidisciplinary healthcare audiences, including, but not limited to:

- Clinic or hospital administrative and management staff
- Health care professionals and trainees including nurses, clinicians, mental health care providers and students in any of these fields
- Staff involved with health outreach and support, including case managers, patient navigators, receptionists and medical billing staff.

Information for Facilitators

Using this Structural Competency Training Manual

This is intended to be a document that facilitators can use to deliver the structural competency training. The curriculum includes:

1. Facilitator guidelines (Appendix N),
2. A summary of helpful terms and concepts (Appendix O),
3. A companion compendium of required readings for the facilitator (Appendix G), and
4. A companion Participant Workbook (Appendix L) that includes all of the handouts required for training participants.

The curriculum was developed for a four-hour training, including breaks. The curriculum is organized as follows:

- Welcome and Introduction
- Module 1: Structures and Health
- Module 2: The Origins of Structural Competency
- Module 3: Responding to Harmful Structures in and Beyond the Clinic
- Conclusion and Evaluation

Each module starts with a section overview that provides an at-a-glance snapshot of the basic information that facilitators need to know in order to implement the module. These include timing, learning objectives, methods of instruction, supplies, required facilitator readings, and a list of corresponding participant workbook pages and PowerPoint slides. Inside each module there are detailed, step-by-step guidelines and facilitator notes for delivering the content and carrying out activities.

Training Supplies and Preparation Checklist

Use the checklist below to prepare for the training. This checklist includes the supplies needed and the preparation required for each module.

| Training Supplies | Facilitator Preparation |
| --- | --- |
| - Flipchart paper - Markers - Masking tape - Appendix N: Facilitator Guidelines - Customized Training Agenda - Appendix L: Participant Workbook - Appendices H, I, J and K | - Complete the required reading for each module. - Review the Participant Workbook (Appendix L). - Print one copy of Appendix L: Participant Workbook for each training participant. - Write key structural competency concepts and definitions on flipchart paper prior to presenting. Display the definitions on the wall in the room. |

Recommended Training Agenda

(Time: 4 Hours)

**Welcome and Introduction (15 minutes)**

- Facilitator and Participant Introductions
- Training Overview and Positionality
- Agenda and Group Agreements

**Module 1 (100 minutes): Structures and Health (Appendix I slide deck)**

- Section 1: Social Structures and Health (35 minutes)
  - Define Structures
  - Review epidemiology illustrating the distribution of health disparities
  - Patient Case
    - Exercise: Participants read chart note and discuss patient case
    - Arrow Diagram 1: Discussion of this patient’s life trajectory and structural influences on this trajectory
- Section 2: Structural Violence and Structural Vulnerability (25 minutes)
  - Definition & discussion of concept
    - Illustration of concept via examples of structural racism: mass incarceration and redlining
    - Definition of “structural vulnerability” and “intersectionality”
  - Reflection: Participants reflect on, write, and discuss influence of structural violence/vulnerability in cases from their clinical or personal experience
- Section 3: Naturalizing Inequality (40 minutes)
  - Definition/discussion of concept, including “implicit frameworks”
  - Exercise: Participants read passage, identifying the implicit frameworks/ naturalization of inequality
  - Examples of naturalizing inequality in health literature/ practice

**BREAK (10 minutes)**

**Module 2 (45 minutes): The Origins of Structural Competency (Appendix J slide deck)**

- Section 1: Cultural Competency and Cultural Humility (5 minutes)
  - Intentions and limitations
- Section 2: Structural Competency and Structural Humility (20 minutes)
  - Motivation behind and definition of structural competency
  - Five goals of structural competency
  - Naming the framework
  - Relationship of structural competency to social determinants of health
- Section 3: Why is Structural Competency Important for Providers to Learn? (20 minutes)
  - The importance of structural competency for providers to improve performance and patient outcomes, and to empower providers to advocate for change
  - Flint, MI example of provider advocacy
  - The structural influences on the practice of healthcare
  - Reflection exercise: What structures were influencing the provider in the patient case reviewed in Module 1?
  - Arrow Diagram 2: Present provider’s personal trajectory and major structural influences
  - Trainees reflect upon and discuss structural influences on their own practice

**Module 3 (60 minutes): Responding to Harmful Structures in and Beyond the Clinic (Appendix K, slides 1-17)**

- Section 1: Structurally Competent Interventions (15 minutes)
  - Examples of structural responses to disparities in health
- Section 2: Levels of Intervention (5 minutes)
  - Introduce Levels: Individual, Interpersonal, Clinic/Institutional, Community, Policy, and Research
- Section 3: Imagining Structural Interventions (25 minutes)
  - Trainees brainstorm and discuss strategies for responding to harmful structural influences at various levels
  - Trainees reflect and write individually on their intentions
- Section 4: Beloved Community (5 minutes)
  - Introduce the concept of beloved community and explain its importance for structurally competent practice
- Section 5: Putting Theory into Practice (10 minutes)
  - Trainees identify at least one intervention strategy to implement to address structural causes of ill health

**Conclusion and Evaluation (10 minutes) (Appendix K, slides 18-20)**
