# Supplementary material for: Structural Competency: Curriculum for Medical Students, Residents, and Interprofessional Teams on the Structural Factors That Produce Health Disparities
Source: MedEdPORTAL. 2020 Mar 13;16:10888. doi: 10.15766/mep_2374-8265.10888 (PMC7182045; doi:10.15766/mep_2374-8265.10888)
Supplement: Supplementary file 1 — A. Manual Background Info.docx B. Manual Intro.docx C. Manual Module 1.docx D. Manual Module 2.docx E. Manual Module 3.docx F. Manual Conclusion and Evaluation.docx G. Supplemental Reading List.docx H. Training Slides Intro.pptx I. Training Slides Module 1.pptx J. Training Slides Module 2.pptx K. Training Slides Module 3.pptx L. Participant Workbook.pdf M. Posttraining Survey.pdf N. Facilitator Guidelines.docx O. Facilitator Preparation - Terms and Concepts.docx P. Participant Sign-in Sheet.docx [file mep-16-10888-s001.zip › B. Manual Intro.docx]

STRUCTURAL COMPETENCY:

A Framework for Recognizing & Responding to Social, Political & Economic Structures to Improve Health


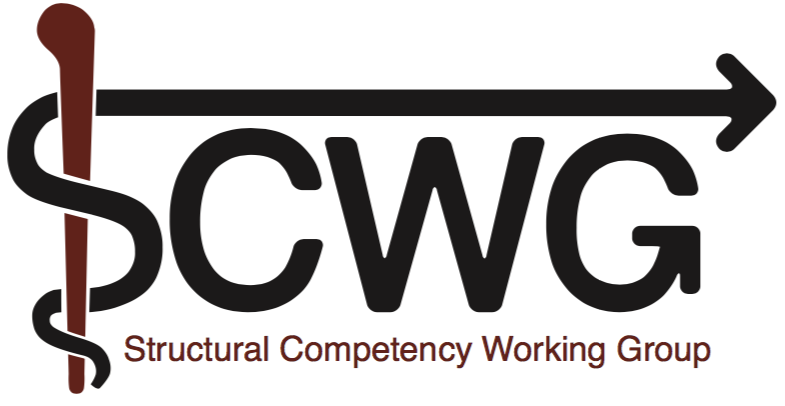


TRAINING CURRICULUM: WELCOME & INTRODUCTION

Updated September 2018

Copyright:

The Structural Competency Working Group ([www.structcomp.org](http://www.structcomp.org)), in order to promote the widest possible dissemination of health curricula, has adopted an open copyright policy for its structural competency training materials. This means that we will grant permission to translate, adapt, or borrow our materials without charging fees or royalties under the following conditions:

- that you credit the Structural Competency Working Group for any borrowed/adapted Working Group materials and inform your audience about who we are and how to learn more about our organization ([structcomp.org](http://structcomp.org)). We suggest the following attribution (and request that it appear on any copyright page):
  - “*These materials have been [borrowed][adapted] from the Structural Competency Working Group,* [*www.structuralcompetency.org*](http://www.structuralcompetency.org)*; you can contact the Group at* [*structuralcompetency@gmail.com*](mailto:structuralcompetency@gmail.com)*”;*
- that your materials are distributed at no cost (or for your production cost only), that is, not-for-profit;
- that you allow others to reproduce/ adapt your edition or adaptation with no fees, royalties, etc. so long as they also do so at no cost or for production cost only, that is, not-for-profit;
- that you provide us with digital versions of your materials (including PDF and/or Microsoft Word files) and work with us so that we can host it on our website;
- that you send us your contact information so we can post it on our website and provide it to people who want to contact you about your edition, adaptation or publication;
- that you contact and stay in touch with the Structural Competency Working Group so that we can learn about your project and make sure you are using the most up-to-date materials.

If you decide to begin translating, adapting, or borrowing our materials, please use the most recently updated versions. Please contact us at [structuralcompetency@gmail.com](mailto:structuralcompetency@gmail.com) to find out if we are currently working on updating our materials, and if anyone else is working on a project similar to your own.

This training curriculum was prepared by Josh Neff, Seth M. Holmes, Kelly R. Knight, Shirley Strong, Ariana Thompson-Lastad, Cara McGuinness, Laura Duncan, Michael J. Harvey, Nimish Saxena, Katiana L. Carey-Simms, Alice Langford, Sara Minahan, Shannon Satterwhite, Lillian Walkover, Jorge De Avila, Brett Lewis, Gregory Chin, Jenifer Matthews, and Nick Nelson of the Structural Competency Working Group ([structcomp.org](http://structcomp.org/)), in collaboration with Sonia Lee and Caitlin Ruppel of Health Outreach Partners ([outreach-partners.org](http://outreach-partners.org/)).

The Structural Competency Working Group’s efforts have been supported by the Berkeley Center for Social Medicine and Deborah Lustig as well as the University of California Humanities Research Institute. Josh Neff’s work on this project has been supported by the UCSF Resource Allocation Program for Trainees (RAPtr), the Greater Good Science Center Hornaday Fellowship, UC Berkeley-UCSF Joint Medical Program Thesis Grant, and the Helen Marguerite Schoeneman Scholarship.

Welcome and Introduction

| Content Time | 15 minutes |
| --- | --- |
| Learning Objective(s): | 1. Set expectations and establish confidence in the facilitator(s). 2. Create a safe and productive learning environment that is engaging and enjoyable. 3. Encourage active participation and listening. |
| Methods of Instruction: | - Facilitator Instruction |
| Sections: | 1. Introductions 2. Training Overview and Positionality 3. Agenda and Group Agreements |
| Supplies: | - Flipchart paper - Markers - Masking tape - Appendix N: Facilitator Guidelines - Appendix L: Participant Workbook - Appendix H: Training Slides Introduction - Appendix P: Participant Sign-in sheet |
| Required Reading for Facilitator: | - Neff, J., Knight, K. R., Satterwhite, S., Nelson, N., Matthews, J., & Holmes, S. M. (2017). Teaching Structure: A Qualitative Evaluation of a Structural Competency Training for Resident Physicians. *Journal of General Internal Medicine*, 32(4), 430-443. - Appendix N: Facilitator Guidelines - Appendix O: Facilitator Preparation -Terms and Concepts |
| Handout(s): | - None |

Section 1: Introductions

**Time: 5 minutes**

**Learning Objective:** To set expectations and establish confidence in the facilitator(s).

**Supplies:**

- Appendix L: Participant Workbook
- Appendix H: Slides: 1-3

**Handout(s):**

- Appendix P: Participant Sign-In Sheet

**Preparation:**

- Complete the required reading for the module prior to presenting.
- Review all handouts for this section prior to presenting the information. Refer to them in the participant workbook, as necessary, throughout the module.
- Distribute one Participant Workbook (Appendix L) to each attendee prior to beginning the training.
- Distribute the participant sign-in sheet (Appendix P) to the participants. Confirm that all participants have signed it.
- Slide 1 (Appendix H) should be displayed (full-screen) as participants enter the room.

1. **Introductions (5 minutes)**
   1. **About the Structural Competency Working Group (2 minutes) (Appendix H: Slide 2):** Provide a description of SCWG.
      - Welcome to the SCWG’s Structural Competency training.
      - We will begin today’s training by introducing ourselves as well as the training and the Structural Competency Working Group that created it.
      - [*Confirm that the participant sign-in sheet is circulating the room and remind all participants to sign it.*]
      - This training is based on SCWG’s structural competency curriculum.
      - Located in the San Francisco Bay Area of California, the Structural Competency Working Group is a collective of clinicians, scholars, public health professionals, medical students, educators, and other community members.
      - The SCWG originally created the structural competency curriculum as a training for residents and clinicians to expand their medical education.
      - The SCWG’s goal is to help promote the training of healthcare professionals in structural competency.
   2. **Facilitator Introductions (1 minute) (Appendix H: Slide 3):** Welcome participants. Provide brief individual introductions of the facilitators.
   3. **Group Introductions (2 minutes) (Appendix H: Slide 3): (Choose one of the two options.)**
      - **Option #1:** Small Group Participant Introductions: Facilitate group introductions.
        - We are now going to ask everyone to please introduce themselves to the group.
        - The challenge is that you have just 10 seconds to complete your introduction.
        - To keep things brief, state your name, title, and years at your organization.
      - **Option #2:** Large Group Participant Introductions. Facilitate group introductions.
        - Due to limited time, and because the group is large, we won’t have enough time to do individual introductions.
        - We have a lot of content to cover today, and we want to be respectful of people’s time and make sure to finish the training on schedule.

- Let’s quickly get to know who is here today by raise of hands.
- [*Ask people to raise their hand if they are in a certain professional position (i.e.: clinicians, outreach workers, front desk staff).*]

Section 2: Training Overview and Positionality

**Time: 5 minutes**

**Learning Objective:** To set expectations and establish confidence in the facilitator(s).

**Supplies:**

- Appendix L: Participant Workbook
- Appendix H: Slides: 4-5

**Handout(s):**

- None

**Preparation:**

- Complete the required reading for the module prior to presenting.

1. **Introduction to the Structural Competency Training (5 minutes)**
   1. **Training Context (2 minutes) (Appendix H: Slide 4):** Provide a description of the Structural Competency training.
      - Review learning objectives listed on slide 4 (Appendix H).
      - To promote health equity and provide appropriate care, health professionals serving vulnerable and underserved communities must be familiar with the factors that influence the health of their patients.
      - Structural competency training aims to strengthen the capacity of health professionals to recognize and address patient health and illness as the product of the broad social, political, and economic systems in which people live, work, and spend their time.
   2. **Facilitator Positionality (3 minutes) (Appendix H: Slide 5):** Highlight the facilitators’ positionality.
      - As facilitators, we want to acknowledge our respective positionalities, privileges, and blind spots.
      - What do we mean by this?
      - [*Facilitators share their respective positionality*.]
      - Our perspectives are often determined by factors including our race, ethnicity, sexual orientation, gender identity, family dynamics, culture, language, nationality, social class, and lived experience, among other things.
      - These factors also contribute to how we are perceived by and treated by others on a daily basis.
      - Privilege contributes to blind spots; we often do not think about problems that do not affect us.
      - It is very important to note that no matter who we are, or what our lived experience is, we all have our own blind spots.
      - If we do not do the work to recognize our blind spots, we risk causing harm to others even if it is not intended.
      - We as facilitators do not claim to be experts. We fully embrace that we are continuing to learn, and we welcome your feedback and perspective throughout this training.

Section 3: Agenda and Group Agreements

**Time: 5 minutes**

- - - **Learning Objective:** To create a safe and productive learning environment that is engaging and enjoyable.

**Supplies:**

- Flipchart paper
- Markers
- Appendix H: Slides: 6-7

**Preparation:**

- Complete the required reading for the module prior to presenting.
- Have blank flipchart paper available on the wall to add proposed group agreements during the group brainstorm.

1. **Training Overview (5 minutes)**
   1. **Training Agenda and Logistics (2 minutes) (Appendix H: Slide 6):**
      - Discuss an overview of planned agenda, including breaks (see below for recommended training agenda) (Appendix B: pg. 10-12)
      - Explain that the training includes different types of activities (i.e.: individual reflection, pair work, and group discussion) that will ask participants to reflect on and share their own experiences and beliefs.
      - Clarify that the content of the training progresses from conceptual material in modules one and two to applied content in module three.
      - Explain the logistics of the training space (i.e.: location of bathrooms, drinking fountains, and exits; Wi-Fi name and password; building passcode; etc.)
   2. **Group Agreements (3 minutes) (Appendix H: Slide 7):** Establish an agreed-upon code of behavior for the group to feel safe and comfortable during the training.
      - The structural competency training covers topics such as health equity, social justice, politics, and identity.
      - These topics can be sensitive in nature, can elicit strong emotional reactions, and require conversations that may be uncomfortable for some participants.
      - Establishing and maintaining a respectful, inclusive training environment is crucial. In order to create a safe and productive learning environment, we’d like to propose group agreements (“ground rules”) for the training.
      - [*Present the established group agreements*.]
        1. Respect the opinions of everyone.
        2. Assume positive intent.
        3. Recognize your blind spots.
        4. Be on time after breaks.
        5. Ask questions and participate.
        6. Silence cell phones and other electronic devices.
        7. Take turns speaking.
        8. Practice self-care.
      - Are there any group agreements listed here that people would like removed?
      - Are there additional group agreements that anyone would like to have added? (if so, add them on flipchart paper on the wall.)

**Recommended Training Agenda (Time: 4 Hours)**

Welcome and Introduction (15 minutes)

- Facilitator and Participant Introductions
- Training Overview and Positionality
- Agenda and Group Agreements

Module 1 (100 minutes): Structures and Health

This module discusses the social structures that influence health, then introduces and defines the concepts of structural violence and naturalizing inequality.

- Section 1: Social Structures and Health (35 minutes)
  - Define Structures (Appendix L pg. 3)
  - Review epidemiology illustrating the distribution of health disparities
  - Patient Case
    - Exercise: Participants read chart note and discuss patient case (Appendix L pg. 1)
    - Arrow Diagram 1: Discussion of this patient’s life trajectory and structural influences on this trajectory (Appendix I pg. 12-15)
- Section 2: Structural Violence and Structural Vulnerability (25 minutes)
  - Definition & discussion of concept
    - Illustration of concept via examples of structural racism: mass incarceration (Appendix I pg. 20-23)
    - Definition of “structural vulnerability” and “intersectionality” (Appendix I pg. 24-26, Appendix L pg. 3)
  - Reflection: Participants reflect on, write, and discuss influence of structural violence/vulnerability in cases from their clinical or personal experience (Appendix I pg. 27, Appendix L pg. 6)
- Section 3: Naturalizing Inequality (40 minutes)
  - Definition/discussion of concept, including “implicit frameworks” (Appendix I pg. 28-31, Appendix L pg. 4)
  - Exercise: Participants read passage, identifying the implicit frameworks/ naturalization of inequality (Appendix I pg. 37-38, Appendix L pg. 7)
  - Examples of naturalizing inequality in health literature/ practice (Appendix I pg. 39-40)

BREAK (10 minutes)

Module 2 (45 minutes): The Origins of Structural Competency

This module addresses the relationship of structural competency to cultural competency and humility and the social determinants of health, then discusses why structural competency is important for healthcare providers to learn.

- Section 1: Cultural Competency and Cultural Humility (5 minutes)
  - Intentions and limitations (Appendix J pg. 2-7)
- Section 2: Structural Competency and Structural Humility (20 minutes)
  - Motivation behind and definition of structural competency (Appendix J pg. 9, Appendix L pg. 9)
  - Five goals of structural competency (Appendix J pg. 10)
  - Naming the framework (Appendix J pg. 12)
  - Relationship of structural competency to social determinants of health (Appendix J pg. 13)
- Section 3: Why is Structural Competency Important for Providers to Learn? (20 minutes)
  - The importance of structural competency for providers to improve performance and patient outcomes, and to empower providers to advocate for change (Appendix J pg. 14-16)
  - Flint, MI example of provider advocacy (Appendix J pg. 17)
  - The structural influences on the practice of healthcare Reflection exercise: What structures were influencing the provider in the patient case reviewed in Module 1? (Appendix J pg. 19-20)
  - Arrow Diagram 2: Present provider’s personal trajectory and major structural influences (Appendix J pg. 21)
  - Trainees reflect upon and discuss structural influences on their own practice (Appendix J pg. 22, Appendix L pg. 8)

Module 3 (60 minutes): Responding to Harmful Structures in and Beyond the Clinic

This module explores ways of responding to harmful social structures, by providing examples of such responses, discussing the various levels at which providers can intervene, and prompting participants to brainstorm strategies.

- Section 1: Structurally Competent Interventions (15 minutes)
  - Examples of structural responses to disparities in health (Appendix K pg. 4-5)
- Section 2: Levels of Intervention (5 minutes)
  - Introduce Levels: Individual, Interpersonal, Clinic/Institutional, Community, Policy, and Research (Appendix K pg. 6-7, Appendix L pg. 10-12)
- Section 3: Imagining Structural Interventions (25 minutes)
  - Trainees brainstorm and discuss strategies for responding to harmful structural influences at various levels (Appendix K pg. 8)
  - Trainees reflect and write individually on their intentions (Appendix K pg. 17, Appendix L pg. 8)
- Section 4: Beloved Community (5 minutes)
  - Introduce the concept of beloved community and explain its importance for structurally competent practice (Appendix K pg. 9-14)
- Section 5: Putting Theory into Practice (10 minutes)
  - Trainees identify at least one intervention strategy to implement to address structural causes of ill health (Appendix K pg. 15-17)

Conclusion and Evaluation (10 minutes)
