# Supplementary material for: Structural Competency: Curriculum for Medical Students, Residents, and Interprofessional Teams on the Structural Factors That Produce Health Disparities
Source: MedEdPORTAL. 2020 Mar 13;16:10888. doi: 10.15766/mep_2374-8265.10888 (PMC7182045; doi:10.15766/mep_2374-8265.10888)
Supplement: Supplementary file 1 — A. Manual Background Info.docx B. Manual Intro.docx C. Manual Module 1.docx D. Manual Module 2.docx E. Manual Module 3.docx F. Manual Conclusion and Evaluation.docx G. Supplemental Reading List.docx H. Training Slides Intro.pptx I. Training Slides Module 1.pptx J. Training Slides Module 2.pptx K. Training Slides Module 3.pptx L. Participant Workbook.pdf M. Posttraining Survey.pdf N. Facilitator Guidelines.docx O. Facilitator Preparation - Terms and Concepts.docx P. Participant Sign-in Sheet.docx [file mep-16-10888-s001.zip › C. Manual Module 1.docx]

STRUCTURAL COMPETENCY:

A Framework for Recognizing & Responding to Social, Political & Economic Structures to Improve Health


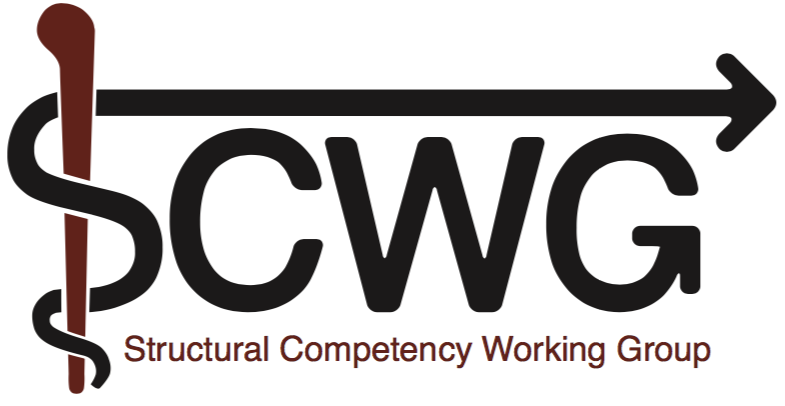


TRAINING CURRICULUM: MODULE 1

Updated September 2018

Copyright:

The Structural Competency Working Group ([www.structcomp.org](http://www.structcomp.org)), in order to promote the widest possible dissemination of health curricula, has adopted an open copyright policy for its structural competency training materials. This means that we will grant permission to translate, adapt, or borrow our materials without charging fees or royalties under the following conditions:

- that you credit the Structural Competency Working Group for any borrowed/adapted Working Group materials and inform your audience about who we are and how to learn more about our organization ([structcomp.org](http://structcomp.org)). We suggest the following attribution (and request that it appear on any copyright page):
  - “*These materials have been [borrowed][adapted] from the Structural Competency Working Group,* [*www.structuralcompetency.org*](http://www.structuralcompetency.org)*; you can contact the Group at* [*structuralcompetency@gmail.com*](mailto:structuralcompetency@gmail.com)*”;*
- that your materials are distributed at no cost (or for your production cost only), that is, not-for-profit;
- that you allow others to reproduce/ adapt your edition or adaptation with no fees, royalties, etc. so long as they also do so at no cost or for production cost only, that is, not-for-profit;
- that you provide us with digital versions of your materials (including PDF and/or Microsoft Word files) and work with us so that we can host it on our website;
- that you send us your contact information so we can post it on our website and provide it to people who want to contact you about your edition, adaptation or publication;
- that you contact and stay in touch with the Structural Competency Working Group so that we can learn about your project and make sure you are using the most up-to-date materials.

If you decide to begin translating, adapting, or borrowing our materials, please use the most recently updated versions. Please contact us at [structuralcompetency@gmail.com](mailto:structuralcompetency@gmail.com) to find out if we are currently working on updating our materials, and if anyone else is working on a project similar to your own.

This training curriculum was prepared by Josh Neff, Seth M. Holmes, Kelly R. Knight, Shirley Strong, Ariana Thompson-Lastad, Cara McGuinness, Laura Duncan, Michael J. Harvey, Nimish Saxena, Katiana L. Carey-Simms, Alice Langford, Sara Minahan, Shannon Satterwhite, Lillian Walkover, Jorge De Avila, Brett Lewis, Gregory Chin, Jenifer Matthews, and Nick Nelson of the Structural Competency Working Group ([structcomp.org](http://structcomp.org/)), in collaboration with Sonia Lee and Caitlin Ruppel of Health Outreach Partners ([outreach-partners.org](http://outreach-partners.org/)).

The Structural Competency Working Group’s efforts have been supported by the Berkeley Center for Social Medicine and Deborah Lustig as well as the University of California Humanities Research Institute. Josh Neff’s work on this project has been supported by the UCSF Resource Allocation Program for Trainees (RAPtr), the Greater Good Science Center Hornaday Fellowship, UC Berkeley-UCSF Joint Medical Program Thesis Grant, and the Helen Marguerite Schoeneman Scholarship.

Module 1: Structures and Health

| Content Time | 100 minutes |
| --- | --- |
| Learning Objective(s): | 1. To identify examples of structures and understand the influence of these structures on health and the provision of care. 2. To define structural violence and structural vulnerability and identify examples of how they influence health. 3. To identify the processes through which inequality is naturalized and examine three implicit frameworks. |
| Methods of Instruction: | - Facilitator Instruction - Large Group Discussion - Individual and Group Activities |
| Sections: | 1. Social Structures and Health 2. Structural Violence and Structural Vulnerability 3. Naturalizing Inequality |
| Supplies: | - Flipchart - Markers - Tape - Appendix N: Facilitator Guidelines - Appendix L: Participant Workbook - Appendix O: Facilitator Preparation -Terms and Concepts - Appendix I: Slides. Module 1 |
| Required Reading for Facilitator: | - Bourgois, P., Holmes, S. M., Sue, K., & Quesada, J. (2017). Structural Vulnerability: Operationalizing the Concept to Address Health Disparities in Clinical Care. *Academic Medicine*, 92(3), 299-307. - Coates, T. N. (2014, June). The Case for Reparations. *The Atlantic.* Retrieved from http://www.theatlantic.com/magazine/archive/2014/06/the-case-for-reparations/361631/ - Farmer, P. E., Nizeye, B., Stulac, S., & Keshavjee, S. (2006). Structural Violence and Clinical Medicine. *Public Library of Science Medicine*, *3*(10), e449. - Martin, N., & Montagne, R. (2017, December 7)*.* Black Mothers Keep Dying After Giving Birth: Shalon Irving’s Story Explains Why. *National Public Radio*. Retrieved from https://www.npr.org/2017/12/07/568948782/black-mothers-keep-dying-after-giving-birth-shalon-irvings-story-explains-why - Rivkin-Fish, M. (2011). Learning the Moral Economy of Commodified Health Care: ‘Community Education,’ Failed Consumers, and the Shaping of Ethical Clinician-Citizens. *Culture, Medicine and Psychiatry,* 35(2), 183-205. - Suarez-Orozco, C., Casanova, S., Martin, M., Katsiaficas, D., Cuellar, V., Smith, N. A., & Dias, S. A. (2015). Toxic Rain in Class: Classroom Interpersonal Microaggressions. *Educational Researcher,* 44(3), 151-160. - Sue, D., Lin, A., Torino, G., Capodilupo, C., & Rivera, D. (2009). Racial Microaggressions and Difficult Dialogues on Race in the Classroom. *Cultural Diversity and Ethnic Minority Psychology,* 15(2), 183-190. |
| Participant Workbook (Appendix L): | - Case Study - Key Concepts - Structural Vulnerability Checklist - Structural Violence Exercise - Naturalizing Inequality Exercise |

Module 1 — Section 1: Social Structures and Health

**Time: 35 minutes**

**Learning Objective: To define and identify social structures that influence health**

**Supplies:**

- Flipchart paper
- Flipchart markers
- Appendix L: Participant Workbook
- Appendix I: Slides 2-15

**Handout:**

- Case Study (Appendix L, pg. 1-2)

**Preparation:**

- Complete the required reading for the module prior to presenting.
- Write the key structural competency concepts and definitions discussed in this section on flipchart paper prior to presenting. Display the definitions on the wall in the room.
  - Key concepts: Social Structures
- Review and refer to the participant workbook, as necessary, throughout the module.
- Slide 2 (Appendix I) should be displayed as the transition into this section.

1. **Introduction to Social Structures (10 minutes)**
   1. **Introduction (5 minutes) (Appendix I: Slide 3-7):** Introduce the section and its learning objectives.

[**Appendix I: Slide 3**]

- - - Review the stated objectives for this particular module.

[**Appendix I: Slide 4**]

- - - The quote on this slide is from Barbara Major, the former Director of St. Thomas Health Clinic.
    - [*Read quote*] “No one has a right to work with poor people unless they have a real analysis of why people are poor.”
    - [*Ask participants to share their reactions*.]
    - This is one way of framing our overarching goal for this session – we want to develop an analysis of why certain people are poor while other people are not poor or, in some cases, are exorbitantly wealthy – and by extension why some people have much higher burdens of disease than others.
    - Much of what we will discuss today may be familiar to you; however, the content we will present has not been systematically incorporated into healthcare-related training.
    - As a note, this training was developed to include all individuals working within and beyond health care settings to support health and access to health care. People who have participated in this training including clinicians, nurses, social workers, case managers, front desk staff, and many others.

[**Appendix I: Slide 5**]

- - - For the purpose of this training, we will use the following definition of social structures:
      - - Social structures are, “The policies, economic systems, and other institutions (judicial systems, schools, etc.) that have produced and maintain modern social inequities as well as health disparities, often along the lines of social categories such as race, class, gender, sexuality, and ability.”
    - One goal of this training is to build a common vocabulary so that we can better address the identified issues.

[**Appendix I: Slide 6**]

- - - This slide provides a visual depiction of how social structures influence health outcomes, either directly or indirectly, by contributing to poverty and inequality.
    - This is an important theme of this session. We are building our capacity to not only describe the link between social inequities and health disparities, but to also look further upstream. We want to understand and analyze the structural factors – policies, economic systems, and social hierarchies such as racism – that create both social inequities and health disparities.

[**Appendix I: Slide 7**]

- - - On this slide is a quote from Rudolph Virchow, a pathologist famous for linking disease to social structures in the late 19^th^ century. This quote is provided to highlight that many of the ideas presented today have been around for a long time.
    - [*Read quote.*] “If medicine is to fulfill her great task, then she must enter the political and social life. Do we not always find the disease of the populace traceable to deficits in society?”
    - [*Ask participants to share their reactions.*]
  1. **Examples (5 minutes) (Appendix I: Slides 8-11):** Provide context by presenting epidemiologic data. Slide 8 is the section/transition slide.

[**Appendix I: Slide 9**]

- - - This graph is from a study conducted in England, led by Michael Marmot. The study used a very large data set collected between 1999 and 2003. The results showed very compellingly that health outcomes are directly correlated with income.
    - [*Indicate on slide.*] On this chart the x-axis is “neighborhood income deprivation,” with the poorest neighborhoods on the left and the wealthiest neighborhoods on the right. The y-axis of the chart is, “disability-free life expectancy.” Each of the lines represents a different region in England.
    - What the chart shows is that there is a difference of 15 years or more in disability-free life expectancy between the poorest and the wealthiest neighborhoods across different regions in England.
    - This graph demonstrates that structures influence how long people live without experiencing disabling harm. This is not meant to devalue the lives of those who have disabilities, only to recognize the inequitable distribution of harms that can lead to disability.

[**Appendix I: Slide 10**]

- - - Let’s look at another example. This map shows the county-level prevalence of diabetes across the United States in 2007. The darker a county is shaded, the higher the proportion of people who have diabetes. The darkest red sections represent rates above 10.6%.
    - What is apparent right away is that there is an uneven distribution of diabetes throughout the country. [*Indicate on map*.] For example, Colorado counties have very low rate of diabetes whereas the state of Mississippi is almost entirely dark red.
    - Healthcare training tends to focus on genetic susceptibility and individual behaviors around diet and exercise to address diabetes. Looking at this map, however, we have to recognize that there is something going on with large-scale influences beyond the individual.
    - [*Click to bring up second map.*] This second map shows persistent poverty in the U.S. by county between 1970 and 2000. The shading on this map represents only metro or non-metro areas. The key part to pay attention to is the counties that are in red. These are the counties that had persistent poverty.
    - Looking at both we see a strikingly similar pattern between counties with a high prevalence of diabetes and counties with persistent poverty.
    - These maps further illustrate the key takeaway from the England study – large-scale influences, what we are collectively referring to as structures, contribute to health outcomes.

[**Appendix I: Slide 11**]

- - - This chart shows that the proportion of the U.S. population with diabetes nearly tripled between 1990 and 2010, from about 3% to about 9% of the population. This further demonstrates that the prevalence of diabetes is driven by some societal-level phenomenon.
    - To further explore the idea of structural influences on health outcomes, we are going to walk through a case study and activity. Before we move on, are there any questions on the content presented thus far?

1. **Activity: Patient Case Study (25 minutes)**
   1. **Introduction (5 minutes) (Appendix I: Slide 12):** Introduce the section.
      - Let’s walk through a case study that illustrates large-scale influences on health. This case study is based on interviews conducted by anthropologist James Quesada with Latino day laborers in California.
      - You can find the information on this slide in your participant workbook (Appendix L) on pages 1-2.
      - [*Note to Facilitator: the following are optional talking points to share as needed.*
        1. *HPI stands for “History of Present Illness”;*
        2. *PMH stands for “Past Medical History”;*
        3. *PSH stands for “Past Surgical History”;*
        4. *SH stands for “Social History”;*
        5. *“Meds” is an abbreviation of medications; and*
        6. *“Neuro/Mental status” refers to observations of the neurological or mental status of the patient.*]
      - What you see written on the slide is a fairly typical example of the information that might be included in a medical note for an emergency room visit.
      - [*Indicate on slide as reading through the talking points*.] What we know from the information available in this note is that:
        1. The patient is a, “37-year-old Spanish-speaking male who was found lying down with a loss of consciousness (LOC).”
        2. In addition, the note states that the patient is a, “frequent flyer well known to the emergency department for alcohol (EtOH)-related trauma and withdrawal associated with seizures.”
        3. The patient history note indicates the patient previously experienced a right orbital fracture due to an assault, but he did not receive surgery for this fracture.
        4. The social history note reads, “heavy alcohol (EtOH) use, other habits unknown. Apparently homeless.”
        5. Furthermore, the medical note reads that the patient is, “noncompliant with all meds.”
        6. Finally, the observation of the mental status of the patient reads, “patient muttering in incoherent Spanish, inconsistently able to answer ‘yes/no’ and follow simple commands.”
   2. **Individual Reactions and Partner and Group Discussion (10 minutes) (Appendix I: Slide 13):** Facilitate discussion of the case study.
      - Turn to the person next to you and spend the next 5 minutes reviewing the case study in your participant workbook (Appendix L) on pages 1-2.
      - Discuss together and write down any thoughts or impressions that you have related to the following questions:
        1. What questions do you have that might help you to better understand the patient’s situation?
        2. What do you notice about the language used in the medical note?

*[Note to facilitator: While facilitating small- or large-group discussion, prompt reflection on the implications and stigmatizing effects of language like “frequent flyer” and “non-compliant.” You may return to slide 12 to highlight the language in the note.]*

- - - 1. Do you think that the writer’s assessment and differential diagnosis are adequate? What would you add? [*Note the author’s emphasis on ethanol use to the possible exclusion of other causes of acute LOC.]*
      2. What social, political, and economic structures might be contributing to the patient’s health outcomes?
    - Let’s spend the next 5 minutes talking as a group about the structural forces that you all see affecting the patient’s story.
    - As a reminder, for the purpose of this training we are choosing to define social structures as, “The policies, economic systems, and other institutions (judicial systems, schools, etc.) that have produced and maintain modern social inequities as well as health disparities, often along the lines of social categories such as race, class, gender, sexuality, and ability.”
    - [*Ask participants to share 1-2 structural forces that they have identified as affecting the patient.]*
  1. **The Corn Farmer Arrow Diagram (10 minutes) (Appendix I: Slides 14-15):** Explain the social structures influencing the patient in the case study.

**[Appendix I: Slide 14]**

- - - We’re going to now go through the structural factors that influenced this particular patient’s case, many aspects of which you all have already suggested.
    - On this slide you will see the parts of the patient’s life that were included in the standard medical history. [*Indicate on slide*.] Specifically, that the patient was a heavy drinker and that he was brought to the emergency department after being found unconscious on the street.
    - As you can see, there are many additional elements of the patient’s life that were not included in his medical chart or in the note made by the emergency room doctor. Let’s look at the patient’s life trajectory.
    - The patient in this case study was born a 4^th^ generation corn farmer in Oaxaca, Mexico. More specifically, he is from an indigenous (Mixtec) community.
    - During the mid-nineties an influx of cheap corn from the United States to Mexico made it so that the patient could not continue to make a living as a farmer, as his parents, grandparents, and great-grandparents had done.
    - In order to support his family, the patient immigrated to the United States to look for work. Specifically, he moved to the Mission District of San Francisco, CA because an acquaintance from his hometown was living there.
    - As an undocumented immigrant, the patient could only find work as a day laborer – work with unreliable income and minimal workplace protections.
    - After several years of working as a day laborer the patient suffered an injury on the job due to unsafe conditions. This injury prevented him from being able to work, and he was unable to obtain workers compensation from the employer at the site where he was injured.
    - Consequently, the patient was unable to afford to pay his portion of rent in the one-bedroom apartment he was sharing with seven other people. He began to sleep on the street. While he was sleeping on the street, the patient was assaulted several times. This trauma and the ongoing precariousness of his situation made the patient start drinking more heavily.
    - Heavy drinking led to the patient losing consciousness and resulted in him being brought to the emergency department.
    - You could describe all of this information that was not included in the medical note as the social determinants of the patient’s health. What we want to do now; however, is look at the social structures, or “upstream” factors, that influenced these social determinants of health.

**[Appendix I: Slide 15]**

- - - First, we must consider the influence of the legacy of colonialism and the systemic marginalization and violence against indigenous communities in Southern Mexico. These factors contributed to the patient’s community in Oaxaca being very poor.
    - Without these factors contributing to the level of poverty in the patient’s hometown, there could have been additional opportunities for local employment when the patient could no longer earn a living as a farmer.
    - Second, we have to examine the factors that led to the patient no longer being able to earn a living as a farmer in his home community. The farmer was put out of business by an influx of cheap corn from the United States into Mexico.
    - This influx was largely the result of the North American Free Trade Agreement (NAFTA), a bill signed into law by President Clinton in 1993. NAFTA prevented Mexico from taxing imported American corn but did not prevent the United States government from continuing to heavily subsidize American agribusiness. As a result, artificially cheap American corn could be sold in Mexico for less than the cost of growing it locally, and an estimated 1-2 million Mexican corn farmers were put out of work. Many of them subsequently immigrated to the US.
    - When the patient moved to the United States to look for work he was unable to find employment except as a day laborer. U.S. immigration policy, racism, and racialized low wage labor markets contributed to the patient’s inability to find stable, well-compensated, safe work.
    - U.S. immigration policy, restrictions in insurance eligibility, and the design of the U.S. healthcare system limit the patient’s access to health care. Although he would have been eligible for care in San Francisco, he was unaware of this. Consequently, when the patient was injured during work he was unable to get the care he needed to recover fully.
    - City and federal policies contributed to gentrification and displacement in San Francisco, with the result being very limited availability of affordable housing for low-income earners. This factored into the patient not being able to afford rent and his subsequent homelessness.
    - Each of the large-scale influences mentioned on this slide – colonialism, NAFTA, immigration policy, gentrification, etc. – could be the topic of their own multi-day trainings. This case study is not intended to focus specifically on each factor that I mentioned, but rather to start the process of thinking through how large-scale social structures may be contributing to the health outcomes of the communities and individual patients that you serve.
    - *[Ask participants if they have any questions and then conclude section one of module one.]*

Module 1 — Section 2: Structural Violence and Structural Vulnerability

**Time: 25 minutes**

**Learning Objective:** To define structural violence and structural vulnerability and identify examples of how they influence patient health.

**Supplies:**

- Flipchart paper
- Flipchart markers
- Appendix L: Participant Workbook
- Appendix I: Slides: 16-27

**Handout(s):**

- Key Concepts
- Structural Vulnerability Checklist
- Structural Violence Exercise

**Preparation:**

- Complete the required reading for the module prior to presenting.
- Write the key structural competency concepts and definitions discussed in this section on flipchart paper prior to presenting. Display the definitions on the wall in the room.
  - Key concepts: Structural Violence and Structural Vulnerability
- Review all handouts for this section prior to presenting the information. Refer to them in the participant workbook, as necessary, throughout the module.

1. **What are Structural Violence and Structural Vulnerability? (25 minutes)**
   1. **Introduction (3 minutes) (Appendix I: Slide 16):** Introduce the section.
      - The goal for this section is to build a shared understanding of structural violence and structural vulnerability, and to develop a vocabulary that you as health care professionals can collectively use to identify, discuss, and address structural causes of harm.
      - To understand the term “structural violence”, it is important that we are clear about what the terms, “structural” and “violence” mean in this context.
      - Violence can be a difficult concept to define, but one common theme through many definitions of violence is unwanted, detrimental force that causes emotional, social, or bodily harm.
      - Interpersonal violence is a phenomenon that many healthcare professionals often hear about and may have experience treating – and we automatically respond to interpersonal violence as morally unacceptable.
      - We’re now going to talk now about structural violence and structural vulnerability. That means looking at how structures (economic, political, social) influence the ways in which people get hurt and looking at who is more or less vulnerable to those structures.
      - Because this kind of violence is harder to recognize than interpersonal violence we don’t always notice it or think of it as equally unacceptable – even though the harm done can be as devastating as a physical act of violence.
   2. **Structural Violence (2 minutes) (Appendix I: Slides 17-18):** Define the key concept and present an example.

**[Appendix I: Slide 17]**

- - - The quote on this slide is from Paul Farmer, a physician and medical anthropologist. This is one definition of structural violence.
    - [*Read quote*.] “Structural violence is one way of describing social arrangements that put individuals and populations in harm’s way... The arrangements are structural because they are embedded in the political and economic organization of our social world; they are violent because they cause injury to people.”
    - It’s important to note the inclusion and meaning of the words “structure” and “violence” in this definition, both bolded on the slide.

**[Appendix I: Slide 18]**

- - - We can apply this definition to our analysis of the case study that we looked at in the first section of today’s training. The large-scale social structures that we discussed – colonialism, NAFTA, city housing policy – are social, economic and political structures that contributed to the physical harm experienced by the patient, including assault and loss of consciousness due to alcohol consumption. This case study is just one representation of the many manifestations of structural violence.
  1. **Structural Racism (10 minutes) (Appendix I: Slides 19-23):** Define the key concept and present an example.

**[Appendix I: Slide 19]**

- - - We’re now going to look at structural racism as one form of enduring structural violence that has been particularly damaging in the United States.
    - The quote on this slide is from Kwame Ture, a prominent organizer in the U.S. Civil Rights Movement, who is also known by his given name Stokely Carmichael and Charles V. Hamilton
    - This quote, taken from the book *Black Power: The Politics of Liberation*, highlights the difference between individual racism and what Ture and Hamilton call “institutional racism” – here analogous to what we are calling structural racism.
    - [*Trainer can read out loud or ask an audience member to do so.*]
    - To emphasize Ture’s point, institutional or structural racism is less noticeable as being caused by any specific individuals, and, in fact, can originate from what Ture calls established and respected forces in society. Ture emphasizes that structural racism is part of the social status quo, not an aberration caused solely by cruel individuals.
    - In spite of not being caused by one particular person, structural racism leaves communities and individuals, “destroyed and maimed physically, emotionally, and intellectually.”

**[Appendix I: Slide 20]**

- - - One contemporary example of structural racism is the War on Drugs and subsequent mass incarceration.
    - This chart illustrates that between the 1800’s and 1970, the proportion of the U.S. population that was incarcerated remained relatively steady. However, between 1970 and 2000 this population quintupled [*became 5x greater*].
    - This rapid growth in the proportion of the U.S. population that was incarcerated was not an accident. Furthermore, it was not the result of increased crime. Most importantly, this increased rate of incarceration did not affect all groups equally.

**[Appendix I: Slide 21]**

- - - This infographic, taken from The Sentencing Project, shows that among U.S. residents born in the country in 2001, one in three black men and one in six Latino men will likely be incarcerated during their life as compared to a lifetime likelihood of one in seventeen for white men.

**[Appendix I: Slide 22]**

- - - The key point is that there is more to history and current events than what we learn in school or see on the news. Often there is a common explanation provided for a given phenomenon that does not hold up to additional facts.
    - For example, some assume or assert that the high rates of incarceration in African American, and to a slightly lesser extent Latinx, communities in the U.S. is due to higher rates of drug use and/or selling and drug-related charges. There are, however, similar rates of drug selling and using across all racial groups in the United States. Communities of color are more heavily policed and punished.
    - [*Optional talking point*.] One common assumption for the increasing rates of incarceration in the U.S. is that crime rates have increased. However, there is no correlation between crime rates and an increase in incarceration in the U.S.
    - [*Optional talking point*.] Another common explanation for the War on Drugs is that it was a policy response to the crack epidemic; however, the War on Drugs was launched in 1982, which was in advance of the crack epidemic.

**[Appendix I: Slide 23]**

- - - The quote on this slide is from President Nixon’s advisor John Ehrlichman. [*Read quote*.] “The Nixon campaign in 1968, and the Nixon White House after that, had two enemies: the antiwar left and black people. You understand what I’m saying? We knew we couldn’t make it illegal to be either against the war or black, but by getting the public to associate the hippies with marijuana and blacks with heroin. And then criminalizing both heavily, we could disrupt those communities. We could arrest their leaders, raid their homes, break up their meetings, and vilify them night after night on the evening news. Did we know we were lying about the drugs? Of course we did.”
    - The deliberate strategy to vilify and undercut the African American community is a clear example of structural racism, one form of structural violence.
    - Structural racism is just one example of structural violence. To further explore the idea of structural influences on health outcomes, we are going to discuss structural vulnerability.
    - Before we move on, are there any questions on the content presented thus far?
  1. **Structural Vulnerability and Intersectionality (10 minutes) (Appendix I: Slides 24-27):** Define the key concepts and present an example.

**[Appendix I: Slide 24]**

- - - The concept of structural vulnerability is closely related to that of structural violence. For the purpose of this training, we will use the following definition of structural vulnerability:
      - - Structural Vulnerability is: “The risk that an individual experiences as a result of structural violence – including their location in multiple socioeconomic hierarchies. Structural vulnerability is not caused by, nor can it be repaired solely by, individual agency or behaviors.”

**[Appendix I: Slide 25]**

- - - Healthcare professionals can use structural vulnerability as a framework for approaching, engaging, and working with patients and communities.
    - For example, if you turn to page 5 of your workbook you will find a document titled, “Structural Vulnerability Checklist.” Spend the next 1-2 minutes reviewing this document. (Appendix L)
    - [*Optional talking point*.] On the document you will see questions that are in bold as well as non-bolded questions. The bold ones are screening questions for the patient. Non-bolded questions are only to be asked as follow-up if the patient in question answers “yes” to the bolded question.
    - Structural vulnerability not only offers healthcare providers a means to talk about structural violence, such as structural racism, as a risk factor for poor health outcomes – just like they already talk about other risk factors.
    - Furthermore, understanding a patient’s structural vulnerability – their risk for experiencing structural violence – can support healthcare professionals in offering patients personalized, holistic preventative medicine and treatment. It helps engage with the causes of poor health, not just the symptoms.

**[Appendix I: Slide 26]**

- - - Related to structural vulnerability is the idea of intersectionality. Intersectionality was a term coined by Kimberlé Williams Crenshaw, a legal scholar.
    - As defined by Ms. Crenshaw, intersectionality, “Holds that the classical conceptualization of oppression within society – such as racism, sexism, classism, ableism, homophobia, transphobia, xenophobia, and belief-based bigotry – do not act independently of each other. Instead, these forms of oppression interrelate, creating a system of oppression that reflects the ‘intersection’ of multiple forms of discrimination.”
    - Another way of saying this is that the whole of various forms of oppression is greater than the sum of each form of oppression.
    - For example, if you are a person of color and queer and a woman, there is a “multiplier” effect in terms of your experience of oppression beyond just simply adding together the marginalization experienced by each of these three oppressed groups.

**[Appendix I: Slide 27]**

- - - Spend the next five minutes writing about examples of structural violence leading to poor health for patients that you have encountered or that other people you know have encountered.
    - What are the structures involved, and how are they violent (how do they harm people)?
    - You can find these prompts and space to write on page 6 in your workbook. (Appendix L)
    - [*Give participants 5 minutes to write. Ask participants to share responses and then conclude section two of module one.*]

Module 1 — Section 3: Naturalizing Inequality

**Time: 40 minutes**

**Learning Objective:** To identify the processes through which inequality is naturalized and examine three implicit frameworks.

**Supplies:**

- Flipchart paper
- Flipchart markers
- Appendix L: Participant Workbook
- Appendix I Slides: 28- 40

**Handout(s):**

- Naturalizing Inequality Exercise (Appendix L pg. 7)

**Preparation:**

- Complete the required reading for the module prior to presenting.
- Write the key structural competency concepts and definitions discussed in this section on flipchart paper prior to presenting. Display the definitions on the wall in the room.
  - Key concepts: Naturalizing Inequality
- Review all handouts for this section prior to presenting the information. Refer to them in the participant workbook, as necessary, throughout the module.

1. **The Process of Naturalizing Inequality (10 minutes)**
   1. **Introduction (1 minute) (Appendix I: Slides 28-29):** Introduce the section.

**[Appendix I: Slide 28]**

- - - In this section we will discuss the concept of naturalizing inequality, a subtler and sometimes more challenging concept than structural violence that is essential for healthcare professionals to understand.

**[Appendix I: Slide 29]**

- - - Why is there not more widespread discussion of structural violence and structural vulnerability in our society and, more specifically, in health and healthcare? [*Ask 3 participants to share brief reactions*.]
  1. **Naturalizing Inequality and Implicit Frameworks (9 minutes) (Appendix I: Slides 30-35):** Define the key concepts and provide an example.

**[Appendix I: Slide 30]**

- - - Naturalizing inequality refers to the ways in which structural violence is ignored and made to seem natural, either by attributing disparities to nature or to individual behavior rather than to structural forces.
    - Ignoring structural violence – naturalizing inequality – makes an inequitable and unjust status quo seem to be appropriate and deserved, consequently contributing to the preservation of that status quo.

**[Appendix I: Slide 31]**

- - - Inequalities can be naturalized through the use of implicit frameworks.
    - Implicit frameworks emphasize individual behaviors, biology, and a misguided application of the term “culture” as explanations for inequities.
    - The word implicit is being used here in the same way that it is used in the term implicit bias. That is to say, implicit frameworks were not necessarily explicitly taught as a way of thinking nor are they necessarily a conscious decision. Rather, implicit frameworks are a way of thinking that has been ingrained in us through society.
    - Implicit frameworks, when over-emphasized, can prevent us from recognizing the structural roots of health disparities: the social systems – such as institutional racism – political systems, and economic policies that have contributed to health disparities.
    - To illustrate this, we will look again at the corn farmer patient case study and the application of three implicit frameworks: culture, individual behavior, and biology.

**[Appendix I: Slide 32]**

- - - In red you see the structures that influenced the patient’s life and in gray you see the word culture in quotations.
    - It is in quotes because this implicit framework misapplies the notion of culture – a rich and nuanced concept in its true meaning – to invoke stereotypes about marginalized groups and deems those stereotypes that group’s “culture.”
    - For example, in the case of the patient, one might attribute the man’s drinking [*Indicate on slide*.] to the stereotype that Latino or Mexican men drink too much, and deem this interpretation as the patient’s health as the result of his culture.
    - This interpretation ignores the structural forces that contributed to the man drinking heavily, such as limited and low paying work, racism, gentrification and displacement [*Indicate on map.]*
    - Cultural stereotypes could be used to explain many parts of the patient’s life trajectory.
    - As a second example, someone might attribute the patient’s choice to immigrate to the U.S. to the stereotype that it is the “culture” of Mexican farmworkers to immigrate.
    - This interpretation ignores the role of NAFTA in limiting work opportunities for farmworkers in Mexico.

[**Appendix I: Slide 33**]

- - - Let’s look at the second implicit framework of individual behavior and choice.
    - This framework, when emphasized, privileges interpretations of health outcomes as the result of individual behaviors and choices rather than a consequence of structural forces.
    - For example, the patient’s drinking might be attributed to his personal shortcomings – his lack of “moral character” – or poor personal choices.
    - This interpretation leaves out the structural influences on his life.
    - Individual moralistic thinking such as this is very common in healthcare, particularly when it comes to addiction.
    - Similar assumptions about the patient might be used to interpret any part of his life path, as indicated by the arrows on the slide.
    - For example, his injury could be interpreted as a personal mistake caused by carelessness. This interpretation does not recognize the occupational safety hazards of low wage work as a day laborer that contributed to the accident.

[**Appendix I: Slide 34**]

- - - A third implicit framework that can be emphasized at the expense of recognizing structural influences on health outcomes is the biology and genetics implicit framework.
    - For example, in the case of the patient, someone could claim that the man was drinking because of a biological predisposition to alcoholism.
    - This claim is often falsely made about indigenous communities. This in turn can prevent recognition of the role of hundreds of years of ongoing oppression, including the history of genocide and land dispossession, which contributes to higher rates of alcoholism and generally worse health outcomes in these communities.

[**Appendix I: Slide 35**]

- - - It is important to note that biology and genetics, individual behaviors and choices, and, in some instances, culture can and do matter for health.
    - This discussion of implicit frameworks does not deny the significant of these factors for individual and community health.
    - The key take-away from this part of the training is that when biology and genetics, individual behaviors and choices, and culture are overemphasized then it distracts from noticing, discussing, and attempting to change the structural injustices that are the primary drivers of health disparities.

1. **Naturalizing Inequality Exercise (15 minutes)**
   1. **Activity (15 minutes) (Appendix I: Slides 36-38):** Introduce and facilitate the activity.

[**Appendix I: Slide 36**]

- - - Turn to page 7 in your participant workbook (Appendix L) where you will find two passages.
    - Spend the next 5 minutes reading through the two passages. As you read through each passage, underline the parts where you see inequality or injustice naturalized through implicit frameworks.
    - Implicit frameworks we discussed include focusing on any of the following instead of the influence of structures: “culture”; individual level behavior or choices; and biology or genetics.
    - Let’s spend the next 10 minutes talking together about the implicit frameworks that people see being used in the two passages.

[**Appendix I: Slide 37**]

- - - *[Ask participants to share the uses of implicit frameworks that they identified for passage one. Share the “biology and genetics” framework and the “cultural” framework notes on the slide. Highlight that Dr. Holmes uses the phrase ‘naturalize inequalities’ near the end of the passage.]*

[**Appendix I: Slide 38**]

- - - *[Ask participants to share the uses of implicit frameworks that they identified for passage two. Share the “individual behavior/choices” framework notes on the slide. Share the “contextually clueless” framework notes on the slide.]*
    - Note that the first two doctors that Abelino visited recommended treatment that was “contextually clueless” with regard to the structural forces at play. Abelino worked as a strawberry picker. If he did not pick enough pounds per hour he could be fired. Slowing down, taking it easy while working, or taking time off to heal was not an option.
    - The key point here is that implicit frameworks can influence not only diagnosis but also treatment of a health issue.
    - [*Ask participants if they have any questions*.]

1. **Naturalizing Inequality in Healthcare (15 minutes)**
   1. **Examples of Naturalizing Inequality (15 minutes) (Appendix I: Slides 39-40):** Illustrate the concept by providing examples from health literature.

[**Appendix I: Slide 39**]

- - - The following slides provide examples from health literature of how inequality is naturalized through implicit frameworks.
    - The first example is an article that was published in 2011 in the International Journal of Behavioral Medicine titled, “The Relevance of Fatalism in the Study of Latinas’ Cancer Screening Behavior: A Systematic Review of the Literature.”
    - An excerpt from the article reads, “Fatalism has been identified as a dominant belief among Latinos and is believed to act as a barrier to cancer prevention.”
    - This study is an example of the implicit framework of culture being used to explain health disparities in Latinos. Emphasizing “culture” in this instance comes at the expense of ignoring structural factors that influence cancer screening. These include a variety of factors that could be included under the banner of “access to care,” ranging from absence of language-concordant clinicians to transportation challenges to lack of health insurance.

[**Appendix I: Slide 40**]

- - - A forthcoming paper from Harvey and McGladrey illustrates the prevalence and power of the individual behaviors and choices implicit framework in public health.
    - The researchers surveyed public health theory courses and found that 93% of the theories taught focused on individual behavior and lifestyle choices.
    - Only one theory course focused on the structural causes of disease distribution.

**4) Module Summary**

**[Appendix I: Slide 41]**

- This Module focused on building a shared language around structures, their effects on health, and frameworks for exploring health outcomes.
- This shared language can allow us to more easily discuss these topics and reflect on how and when structures come into play in our work and daily lives.
- This slide explores the key terms introduced in this module and will serve as a wrap-up slide for this section of the presentation.
- We first defined social structures as policies, economic systems, and other institutions that maintain modern social inequities as well as health disparities.
- Structural competency directly addresses the effect on health of these structures.
- We then described the term structural violence to explore how social structures can cause harm, for example, through structural racism or mass incarceration.
- Structural vulnerability allowed us to explore how risks for such violence are distributed throughout society and think about how to address such risks.
- Lastly, we explored the term naturalizing inequality and how structural forces can be explained away by various implicit frameworks.
- *Ask for any questions or reflections then conclude Module 1*
