# Supplementary material for: Structural Competency: Curriculum for Medical Students, Residents, and Interprofessional Teams on the Structural Factors That Produce Health Disparities
Source: MedEdPORTAL. 2020 Mar 13;16:10888. doi: 10.15766/mep_2374-8265.10888 (PMC7182045; doi:10.15766/mep_2374-8265.10888)
Supplement: Supplementary file 1 — A. Manual Background Info.docx B. Manual Intro.docx C. Manual Module 1.docx D. Manual Module 2.docx E. Manual Module 3.docx F. Manual Conclusion and Evaluation.docx G. Supplemental Reading List.docx H. Training Slides Intro.pptx I. Training Slides Module 1.pptx J. Training Slides Module 2.pptx K. Training Slides Module 3.pptx L. Participant Workbook.pdf M. Posttraining Survey.pdf N. Facilitator Guidelines.docx O. Facilitator Preparation - Terms and Concepts.docx P. Participant Sign-in Sheet.docx [file mep-16-10888-s001.zip › D. Manual Module 2.docx]

STRUCTURAL COMPETENCY:

A Framework for Recognizing & Responding to Social, Political & Economic Structures to Improve Health


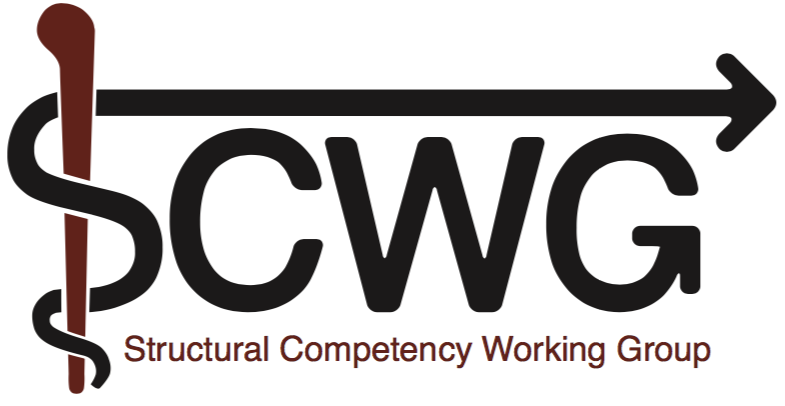


TRAINING CURRICULUM: MODULE 2

Updated September 2018

Copyright:

The Structural Competency Working Group ([www.structcomp.org](http://www.structcomp.org)), in order to promote the widest possible dissemination of health curricula, has adopted an open copyright policy for its structural competency training materials. This means that we will grant permission to translate, adapt, or borrow our materials without charging fees or royalties under the following conditions:

- that you credit the Structural Competency Working Group for any borrowed/adapted Working Group materials and inform your audience about who we are and how to learn more about our organization ([structcomp.org](http://structcomp.org)). We suggest the following attribution (and request that it appear on any copyright page):
  - “*These materials have been [borrowed][adapted] from the Structural Competency Working Group,* [*www.structuralcompetency.org*](http://www.structuralcompetency.org)*; you can contact the Group at* [*structuralcompentency@gmail.com*](mailto:structuralcompentency@gmail.com)*”;*
- that your materials are distributed at no cost (or for your production cost only), that is, not-for-profit;
- that you allow others to reproduce/ adapt your edition or adaptation with no fees, royalties, etc. so long as they also do so at no cost or for production cost only, that is, not-for-profit;
- that you provide us with digital versions of your materials (including PDF and/or Microsoft Word files) and work with us so that we can host it on our website;
- that you send us your contact information so we can post it on our website and provide it to people who want to contact you about your edition, adaptation or publication;
- that you contact and stay in touch with the Structural Competency Working Group so that we can learn about your project and make sure you are using the most up-to-date materials.

If you decide to begin translating, adapting, or borrowing our materials, please use the most recently updated versions. Please contact us at [structuralcompetency@gmail.com](mailto:structuralcompetency@gmail.com) to find out if we are currently working on updating our materials, and if anyone else is working on a project similar to your own.

This training curriculum was prepared by Josh Neff, Seth M. Holmes, Kelly R. Knight, Shirley Strong, Ariana Thompson-Lastad, Cara McGuinness, Laura Duncan, Michael J. Harvey, Nimish Saxena, Katiana L. Carey-Simms, Alice Langford, Sara Minahan, Shannon Satterwhite, Lillian Walkover, Jorge De Avila, Brett Lewis, Gregory Chin, Jenifer Matthews, and Nick Nelson of the Structural Competency Working Group ([structcomp.org](http://structcomp.org/)), in collaboration with Sonia Lee and Caitlin Ruppel of Health Outreach Partners ([outreach-partners.org](http://outreach-partners.org/)).

The Structural Competency Working Group’s efforts have been supported by the Berkeley Center for Social Medicine and Deborah Lustig as well as the University of California Humanities Research Institute. Josh Neff’s work on this project has been supported by the UCSF Resource Allocation Program for Trainees (RAPtr), the Greater Good Science Center Hornaday Fellowship, UC Berkeley-UCSF Joint Medical Program Thesis Grant, and the Helen Marguerite Schoeneman Scholarship.

Module 2: The Origins of
Structural Competency

| Content Time | 45 minutes |
| --- | --- |
| Learning Objective(s): | 1. To reflect on the strengths and limitations of using the cultural competency and cultural humility approaches to explain disparities in health and health care. 2. To define structural competency and describe the five goals of the framework. 3. To explain the relationship between structural competency and the social determinants of health. 4. To equip healthcare professionals with concepts and vocabulary to analyze, discuss, and respond to structural violence and structural vulnerability. |
| Methods of Instruction: | - Facilitator Instruction - Large Group Discussion - Individual and Group Activities |
| Sections: | 1. Cultural Competency and Cultural Humility 2. Structural Competency and Structural Humility 3. Naming the Framework 4. Why is Structural Competency Important for Providers to Learn? |
| Supplies: | - Flipchart - Markers - Tape - Appendix N: Facilitator Guidelines - Appendix L: Participant Workbook - Appendix O: Facilitator Preparation -Terms and Concepts - Appendix J: Slides. Module 2 |
| Required Reading for Facilitator: | - Gregg, J., & Saha, S. (2006). Losing Culture on the Way to Competence: The Use and Misuse of Culture in Medical Education. *Academic Medicine*, 81(6), 542-546. - Metzl, J. M., & Roberts, D. E. (2014). Structural competency meets structural racism: race, politics, and the structure of medical knowledge. *Virtual Mentor,* 16(9), 674-690. - Thackrah, R. D., & Thompson, S. (2013). Refining the concept of cultural competence: building on decades of progress. *The Medical Journal of Australia,* 199 (1), 35-38. |
| Handout(s): | - Key Concepts (Appendix L, pg. 3-4) - Components of Structural Competency (Appendix L, pg. 9) - Your Arrow Exercise (Appendix L, pg. 8) |

Module 2 — Section 1: Cultural Competency and Cultural Humility

**Time: 5 minutes**

**Learning Objective:** To reflect on the strengths and limitations of using the cultural competency and cultural humility approaches to explain disparities in health and health care.

**Supplies:**

- Flipchart paper
- Flipchart markers
- Appendix L: Participant Workbook
- Appendix J: Slides 2-8

**Handout(s):**

- Key Concepts (Appendix L, pg. 3-4)

**Preparation:**

- Complete the required reading for the module prior to presenting.
- Write the key concepts and definitions discussed in this section on flipchart paper prior to presenting. Display the definitions on the wall in the room.
  - Key concepts: Cultural Competency and Cultural Humility
- Review all handouts for this section prior to presenting the information. Refer to them in the participant workbook, as necessary, throughout the module.

1. **Cultural Competency and Cultural Humility (5 minutes)**
   1. **Introduction (1 minute) (Appendix J: Slides 2-3):** Introduce the section and its learning objectives.

[**Appendix J: Slide 3**]

- - - Structural competency is about building our capacity to not only describe the link between social inequities and health disparities, but to also understand, analyze, and address the structural factors – policies, economic systems, and social hierarchies – that create both social inequities and health disparities.
    - To understand the origin and intent of the structural competency framework, it is important to first discuss cultural competency and cultural humility, and the strengths and limitations of both these concepts for explaining disparities in health and health care.
  1. **The Intention of Cultural Competency (2 minutes) (Appendix J: Slides 4-5):** Explain the key concept and provide an example.

[**Appendix J: Slide 4**]

- - - The concept of cultural competence originated in the late 1980s and has been growing more popular since.
    - For the purpose of this training we will define culture simply. Culture is the, “attitudes and behaviors, which are characteristic of a social group or community.”
    - Cultural competency, in its original form, was intended to help care providers to intentionally think through how their culture – their attitudes and behaviors characteristic of their social group or community – influenced their interactions with and perceptions of patients.

[**Appendix J: Slide 5**]

- - - As cultural competency training has spread the framework has taken on a very different form than that introduced by the framers in the 1980’s.
    - Cultural competency has become, in many instances of medical training, a “list of traits” to memorize. Not infrequently, this “list of traits” is tantamount to a list of stereotypes about various non-White ethnic or religious groups, as illustrated here.
    - This slide describes an excerpt from a Nursing textbook which was published in 2014.
    - As you can see, this table lists “cultural differences in response to pain” for several groups:
    - There are many issues to note with these categories of people. To highlight a few:
      1. “Arabs/Muslims” is not a category. Not all Arab peoples are Muslim. Fewer than 20% of Muslims are Arab, and the majority of Muslims are “Asian.”
      2. The category of “Asians” includes 60% of the world’s population and immense cultural diversity.
      3. Though many groups were highlighted, there was no “White” category included on the textbook’s list. We are led to believe that white people are an undifferentiated mass of people that can be described in broad strokes.
    - This version of cultural competency encourages providers to see patients as a part of groups that can be defined in simplistic, essentializing ways.
  1. **The Intention of Cultural Humility (1 minute) (Appendix J: Slide 6):** Explain the key concept and provide an example.
     - One example of a more intentional approach to understanding and addressing cultural differences between and among providers and patients is cultural humility.
     - In 1998, two African American pediatricians Melanie Tervalon and Jann Murray-Garcia, introduced the concept of cultural humility.
     - In a now frequently cited article, they defined cultural humility as, “A commitment and active engagement in a lifelong process that individuals enter into on an ongoing basis with patients, communities, colleagues, and with themselves.”
     - The cultural humility approach is a critique of and direct response to the “list of traits” version of cultural competency training.
     - The approach emphasizes that one cannot know and should not assume what a person’s relationship to their health will be based on their appearance and perceived belonging to a certain group.
     - The cultural humility approach is a tremendous improvement on cultural competency. Even so, this approach has limitations for explaining patient and community health outcomes.
  2. **Limitations of Cultural Humility (1 minute) (Appendix J: Slides 7-8):** Explain the key concept and provide an example.

[**Appendix J: Slide 7**]

- - - The concept of cultural humility does not attempt to explain or address broader structural drivers of inequality in health and health care.
    - Structural drivers of health and health care are increasingly being recognized as issues that warrant attention.
    - However, in medical training, these issues are often taught within the context of cultural competency or cultural humility.

- - - This can lead to the conflation of cultural difference and structural violence, when the two often have little to do with one another. Culture is not the root cause of the vast majority of health disparities.
    - Rather, these health disparities are driven by social, political, and economic structural factors.

[**Appendix J: Slide 8**]

- - - As we discussed earlier in the section on implicit frameworks, focusing on culture rather than social, political, and economic factors is one way that structural violence can be naturalized and perpetuated.
    - [*Ask participants if they have any questions and then conclude section one of module two.*]

Module 2 — Section 2: Structural Competency and Structural Humility

**Time: 20 minutes**

**Learning Objective:** To define structural competency and describe the five goals of the framework.

**Supplies:**

- Flipchart paper
- Flipchart markers
- Appendix L: Participant Workbook
- Appendix J: Slides 9-13

**Handout(s):**

- Key Concepts
- Components of Structural Competency

**Preparation:**

- Complete the required reading for the module prior to presenting.
- Write the key structural competency concepts and definitions discussed in this section on flipchart paper prior to presenting. Display the definitions on the wall in the room.
  - Key concepts: Structural Competency and Structural Humility
- Review all handouts for this section prior to presenting the information. Refer to them in the participant workbook (Appendix L), as necessary, throughout the module.

1. **What are Structural Competency and Structural Humility? (10 minutes)**
   1. **Structural Competency (5 minutes) (Appendix J: Slide 9):** Define the key concept.
      - The structural competency framework was developed in response to the identified lack of systematic training on structural factors – policies, economic systems, and social hierarchies – in medical education, and, more broadly, in the training of providers.
      - Dr. Jonathan Metzl first proposed the concept of structural competency in his book titled, *The Protest Psychosis: How Schizophrenia Became a Black Disease,* published in 2011*.*
      - Dr. Metzl and his colleague, Dr. Helena Hansen, jointly proposed structural competency as a model for medical education in 2014. They are both MDs with PhDs in social sciences.
      - Structural competency training equips healthcare professionals with, “the capacity...to recognize and respond to health and illness as the downstream effects of broad social, political, and economic structures.”
   2. **The Five Goals of Structural Competency (2 minutes) (Appendix J: Slide 10):** Define the five goals of the structural competency training.
      - This slide lists the five principle goals of structural competency training, as articulated by the Structural Competency Working Group. Through training, providers will develop their capacity to:
        1. Recognize the influence of social structures on patient health;
        2. Recognize the influence of social structures on the practice of health care;
        3. Respond to the influences of social structures in the clinical setting;
        4. Respond to the influences of social structures beyond the clinic; and
        5. Practice structural humility.
   3. **Structural Humility (3 minutes) (Appendix J: Slide 11):** Define the key concept.
      - When proposing the structural competency framework, Drs. Metzl and Hansen took a cue from the cultural humility critique of cultural competency and generated a working definition of structural humility.
      - According to Dr. Hansen, “Structural humility cautions providers against making assumptions about the role of structures in patients’ lives, instead encouraging collaboration with patients and communities in developing understanding of and responses to structural vulnerability.”
      - The idea to caution, “...providers against making assumptions about the role of structures in patients’ lives,” is intended to prevent structural stereotyping of patients in order to avoid the “list of traits” approach to cultural competency.
      - Another key point of structural humility is that it encourages, “...collaboration with patients and communities in developing understanding of and responses to structural vulnerability.”
      - The takeaway is that expertise in understanding and addressing structural issues does not necessarily lie with providers. Rather, providers must work with and follow the lead of community members, particularly those who already address structural issues.
2. **Naming the Framework (5 minutes)**
   1. **“Structural” and “Competency” (5 minutes) (Appendix J: Slide 12):** Explain the name of the framework.
      - What is the intent behind naming the framework structural competency?
      - The word “structural” is used here in the same sense as it is used in the terms “structural forces” or “structural violence.”
        - - [*Optional talking point*.] As a reminder, for the purpose of this training social structures are defined as, “The policies, economic systems, and other institutions (judicial systems, schools, etc.) that have produced and maintain modern social inequities as well as health disparities, often along the lines of social categories such as race, class, gender, sexuality, and ability.”
      - The word “structural” was specifically chosen to keep the focus of the framework on “upstream” factors and to prevent the original meaning from being altered as the concept becomes more widely taught and applied.
      - This dilution of the original meaning has happened with the concept of social determinants of health, for example, which often focuses more on factors that are further “downstream” than the structural ones we’ve been describing. We will discuss this more in the next section of the training.
      - The word “competency” was chosen for the framework for three important reasons:
        1. First, it is used in part because the term is now well known as a result of cultural competency being a widely taught model of care delivery.
        2. Second, health professional education is now being framed in terms of various “competencies” in an effort to expand the focus of training beyond simple memorization of medical facts. For example, medical trainees are now expected to develop competency in patient care and communication.
        3. Finally, the term “competency” suggests that the framework should be a standard part of training for all providers. If not for three identified benefits of using the word “competency,” this framework might be called “structural attentiveness” or “structural responsiveness.”
3. **Structural Competency and the Social Determinants of Health (5 minutes)**
   1. **Social Determinants of Health (5 minutes) (Appendix J: Slide 13):** Define the key concept.
      - The structural competency framework builds upon and expands existing frameworks that are used to understand and explain health disparities.
      - In addition to cultural competency and cultural humility, structural competency expands the Social Determinants of Health (SDOH) approach.
      - The original framers of the Social Determinants of Health (SDOH) approach were analyzing, discussing, and writing about social structures – policies, economic systems, and social hierarchies – and related structural issues.
      - However, this is often not reflected in how health care staff are trained.
      - As visually depicted on this slide, Social Determinants of Health training is most often limited to describing research that links social inequities with health disparities without exploring the root causes of these identified social inequities. [*Indicate on slide*.]
      - The structural competency framework builds upon this narrower interpretation of the Social Determinants of Health.
      - Specifically, structural competency is about recognizing and understanding the policies, economic systems, and social hierarchies that underlie poverty and inequality, and therefore contribute to disparities in health and health care.
      - We can use the phrase, “structural determinants of the social determinants of health,” as a way to remind ourselves of this relationship between structural competency and the uncritical version of the Social Determinants of Health.”
      - [*Ask participants if they have any questions and then conclude section two of module two.*]

Module 2 — Section 3: Why is Structural Competency Important for Providers to Learn?

**Time: 20 minutes**

**Learning Objective:** To equip providers to leverage the structural competency approach and vocabulary to analyze, discuss, and respond to structural violence and structural vulnerability.

**Supplies:**

- Flipchart paper
- Flipchart markers
- Appendix L: Participant Workbook
- Appendix J: Slides 14-24

**Handout(s):**

- Components of Structural Competency (Appendix L, pg 9)
- Your Arrow Diagram Exercise (Appendix L, pg. 8)

**Preparation:**

- Complete the required reading for the module prior to presenting.
- Write the key structural competency concepts and definitions discussed in this section on flipchart paper prior to presenting. Display the definitions on the wall in the room.
  - Key concepts: Structural Competency
- Review all handouts for this section prior to presenting the information. Refer to them in the participant workbook, as necessary, throughout the module.

1. **Why Providers Should Participate in Structural Competency Training (5 minutes)**
   1. **Introduction (5 minutes) (Appendix J: Slides 14-17):** Present arguments for why healthcare professionals should learn about structural competency.

[**Appendix J: Slide 14**]

- - - A common question that the Structural Competency Working Group has received is, “Why is this important for providers to learn?”
    - An essential premise of structural competency is that there is no neutral position.
    - Desmond Tutu wrote, “If you are neutral in situations of injustice, you have chosen the side of the oppressor. If an elephant has its foot on the tail of a mouse and you say that you are neutral, the mouse will not appreciate your neutrality.”
    - If providers are not analyzing and responding to health disparities using the structural competency framework, then some other framework is being used.
    - This could include using frameworks that respond to health disparities as the result of individual interactions, such as cultural competency and cultural humility, or the three implicit frameworks that we discussed earlier: culture, individual behavior, and biology. It might also include using frameworks that respond to health disparities at most as the result of social inequities, such as the social determinants of health framework.
    - Structural competency takes the position of responding to health disparities as the consequence of harmful social, political, and economic structures that produce social inequities.

[**Appendix J: Slide 15**]

- - - When providers are trained in structural competency – that is to say, when they analyze and respond to health disparities as the results of harmful social structures – this can (1) improve the work experience of providers; (2) improve patient health outcomes; and (3) empower providers to advocate for systemic change.

[**Appendix J: Slide 16**]

- - - How does structural competency empower providers to advocate for systemic change?
    - Providers regularly witness and directly engage with the embodied human consequences of structural violence and structural vulnerability.
    - Advocacy grounded in the provider experience may be less likely to be dismissed as partisan and therefore may be more effective in changing harmful social structures.
    - Providers can use the structural competency framework and vocabulary when engaging in provider-driven advocacy efforts.

[**Appendix J: Slide 17**]

- - - One example of this is the advocacy work of Dr. Mona Hanna-Attisha.
    - Dr. Mona Hanna-Attisha is an Iraqi-American pediatrician based in Flint, MI. She played a key role in drawing national attention to the water crisis in Flint.
    - Inspired in part by her clinical observations, Dr. Mona Hanna-Attisha collaborated with others at the hospital where she worked to conduct epidemiological research on lead blood-screening data.
    - Her research demonstrated a doubling in elevated blood lead levels following a change in Flint’s water source. Rather than wait for peer review of her research, Dr. Mona Hanna-Attisha held an urgent press conference to let the public know about the findings.
    - The community in Flint had already tried for a year to bring attention to the issue. The doctor’s press conference marked a turning point in the work underway to address the lead levels in the water.
    - Although state lawmakers initially dismissed Dr. Mona Hanna-Attisha’s claims, the press took note and lawmakers started to pay attention. Eventually, state lawmakers took steps to address the issue.
  1. **How does structural competency training improve the work experience of providers? (10 minutes) (Appendix J: Slides 18-22):** Present arguments for why structural competency training can improve provider experiences.

[**Appendix J: Slide 18**]

- - - Finally, structural competency can benefit providers themselves. As we’ll talk about in a moment, structures not only affect the health of our patients—they also affect the way that healthcare is practiced. For instance, the 15-minute (or shorter) primary care visit is not a law of nature—it’s a contemporary reality brought into existence by various economic and political structural influences. One of the effects of these structures is very high rates of burnout among providers. A structural lens helps us to recognize—and work to address—the structural factors that are harmful to us as providers. And, as the slide says, if we as providers aren’t working to address these structures, who is going to do it for us?
    - Additionally, some people suspect that structural competency can promote empathy among providers for their patients, by promoting increased awareness of structural violence and the implicit frameworks by which inequality gets naturalized (and patients get blamed for structurally-influenced illnesses). Studies have found that increased empathy for patients is correlated with decreased burnout, so some have suggested that it is possible that learning structural competency could also directly reduce rates of provider burnout.

[**Appendix J: Slide 19**]

- The content presented thus far in the training has focused on how structures influence the provider and patient encounter, and more specifically, the health and health care of the patient.
  - - It is very important, however, that we also identify the ways that structures influence the working conditions of providers.

[**Appendix J: Slide 20**]

- - - When we assume positive intent and give the benefit of doubt, it is possible to frame the assumptions and actions of providers not as ill-willed, but as the result of a system that has molded providers to interact with and provide health care and social services to patients in a very specific manner.
    - This can be recognized as something that happens across all providers, ranging from frontline staff to clinicians and case managers to health center leadership.

[**Appendix J: Slide 21**]

- - - On this slide you will see a diagram similar to the one used in the previous patient case studies.
    - As we look at the course of this providers trajectory, what structures may have influenced this provider, and in turn, their encounter with this patient?
    - *Ask for suggestions from the audience, and then display the social structures that overylay this arrow diagram.*

[**Appendix J: Slide 22**]

- - - [*Explain the factors that shaped the person’s training and career trajectory. Elicit discussion on how each structure contributes to framing and where it might stem from.*]
  1. **Social structures in your life and training (5 minutes) (Appendix J: Slides 23-24):** Facilitate the “Your Arrow Diagram” exercise (Appendix L, pg. 8).
     - On page 8 in your participant workbook (Appendix L) you will find a space to create your arrow diagram. Spend the next 5 minutes creating this diagram based upon your own personal experience.
     - What social structures influenced your training? What social structures are present in your day to day work, and how do they influence your interactions with your colleagues, with patients, and with the community?
     - [*Ask participants if they have any questions*.]

[**Appendix J: Slide 24**]

- This slide is a teaser for a session about the structural influences on Evidence-Based Medicine, or EBM, developed by Josh Neff, one of the founders and leaders of the SCWG. The three main take-aways from this session, which we don’t have time to go into more deeply today, are as follows:
- First, “Population-level data does not tell us about the best treatment for an individual patient.” An assumption intrinsic to EBM is that whatever findings are true for the population of a given study or studies will necessarily apply to individual patients. There are various challenges to this idea that EBM does not take into account.
- Second, “According to EBM, ‘good evidence’ can only be generated for phenomena with controllable variables, which does not include complex social phenomena.” EBM is a knowledge hierarchy in which knowledge supported by randomized controlled trials (RCTs) is seen as more legitimate than other forms of knowledge – only RCTs count as truly “good evidence.” However, not everything can be studied by RCTs—for instance, complex phenomena such as the influence of social structures cannot be studied by RCT. That doesn’t mean that their significance is less “real”—it just has to be studied through other modes.
- Third and finally, “EBM does not adequately account for threats to scientific integrity including academic incentives and the vested interests of study funding sources (big pharma, etc.).” Again, EBM says that we should trust information if it is generated by RCTs. However, RCTs are quite expensive and are subject to corrupting influences including pressure in academia to have positive findings (and little incentive to run confirmation trials) and biased study design in studies funded by entities such as the pharmaceutical industry. In other words, the question to ask should not simply be whether a conclusion is backed by an RCT—we need to ask whether a given RCT, or any other type of study, is credible given its study design and the interests of those designing it. As longtime editor-in-chief of the Lancet, Richard Horton, said in 2015, “The case against science is straightforward: much of the scientific literature, perhaps half, may simply be untrue.”
- Again, without going further into the details, point of this slide is to recognize (a) that even something that seems very objective in healthcare practice, such as evidence-based-medicine, is also subject to structural influences, and (b) that learning about and being aware of those structural influences can help us to be better providers, and to fix systemic problems in healthcare.
  - - [*Ask participants if they have any questions and then conclude section three of module two.*]

1. **Module 2 Summary**

[**Appendix J: Slide 25**]

- This module focused on the history of the cultural competency, cultural humility, and structural competency movements.
- As a brief summary, we first discussed how cultural competency began as a framework for helping acknowledge and engage with different understandings of illness and health
- We also addressed how many institutions and educators have focused cultural competency trainings on a “list of traits” that often included stereotypes about marginalized groups
- Cultural humility was introduced in response to this framework and emphasized humility, self-reflection, self-critique, and lifelong learning
- Structural competency built on these movements to introduce a framework that recognized the influences of structures on patient health and healthcare practice
- We also explored how structures effect not only patient health, but also the medical system, providers, and trainees.
- This can help us acknowledge and investigate the how we are all affected by structure in different ways.
- *Ask for any questions or reflections then conclude Module 2*
