# Supplementary material for: Structural Competency: Curriculum for Medical Students, Residents, and Interprofessional Teams on the Structural Factors That Produce Health Disparities
Source: MedEdPORTAL. 2020 Mar 13;16:10888. doi: 10.15766/mep_2374-8265.10888 (PMC7182045; doi:10.15766/mep_2374-8265.10888)
Supplement: Supplementary file 1 — A. Manual Background Info.docx B. Manual Intro.docx C. Manual Module 1.docx D. Manual Module 2.docx E. Manual Module 3.docx F. Manual Conclusion and Evaluation.docx G. Supplemental Reading List.docx H. Training Slides Intro.pptx I. Training Slides Module 1.pptx J. Training Slides Module 2.pptx K. Training Slides Module 3.pptx L. Participant Workbook.pdf M. Posttraining Survey.pdf N. Facilitator Guidelines.docx O. Facilitator Preparation - Terms and Concepts.docx P. Participant Sign-in Sheet.docx [file mep-16-10888-s001.zip › E. Manual Module 3.docx]

STRUCTURAL COMPETENCY:

A Framework for Recognizing & Responding to Social, Political & Economic Structures to Improve Health


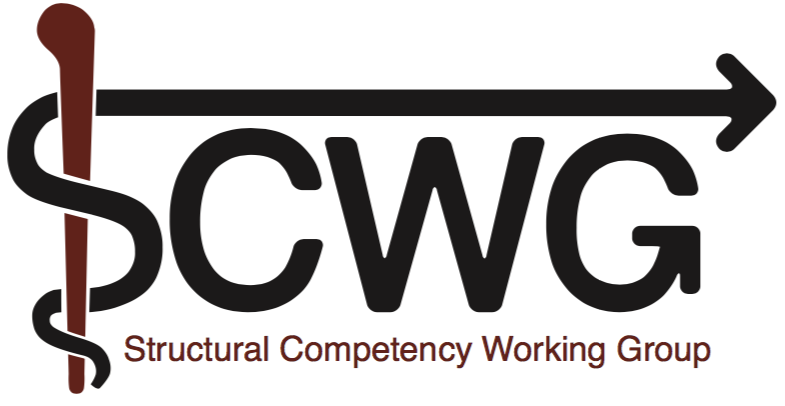


TRAINING CURRICULUM: MODULE 3

Updated September 2018

Copyright:

The Structural Competency Working Group ([www.structcomp.org](http://www.structcomp.org)), in order to promote the widest possible dissemination of health curricula, has adopted an open copyright policy for its structural competency training materials. This means that we will grant permission to translate, adapt, or borrow our materials without charging fees or royalties under the following conditions:

- that you credit the Structural Competency Working Group for any borrowed/adapted Working Group materials and inform your audience about who we are and how to learn more about our organization ([structcomp.org](http://structcomp.org)). We suggest the following attribution (and request that it appear on any copyright page):
  - “*These materials have been [borrowed][adapted] from the Structural Competency Working Group,* [*www.structuralcompetency.org*](http://www.structuralcompetency.org)*; you can contact the Group at* [*structuralcompentency@gmail.com*](mailto:structuralcompentency@gmail.com)*”;*
- that your materials are distributed at no cost (or for your production cost only), that is, not-for-profit;
- that you allow others to reproduce/ adapt your edition or adaptation with no fees, royalties, etc. so long as they also do so at no cost or for production cost only, that is, not-for-profit;
- that you provide us with digital versions of your materials (including PDF and/or Microsoft Word files) and work with us so that we can host it on our website;
- that you send us your contact information so we can post it on our website and provide it to people who want to contact you about your edition, adaptation or publication;
- that you contact and stay in touch with the Structural Competency Working Group so that we can learn about your project and make sure you are using the most up-to-date materials.

If you decide to begin translating, adapting, or borrowing our materials, please use the most recently updated versions. Please contact us at [structuralcompetency@gmail.com](mailto:structuralcompetency@gmail.com) to find out if we are currently working on updating our materials, and if anyone else is working on a project similar to your own.

This training curriculum was prepared by Josh Neff, Seth M. Holmes, Kelly R. Knight, Shirley Strong, Ariana Thompson-Lastad, Cara McGuinness, Laura Duncan, Michael J. Harvey, Nimish Saxena, Katiana L. Carey-Simms, Alice Langford, Sara Minahan, Shannon Satterwhite, Lillian Walkover, Jorge De Avila, Brett Lewis, Gregory Chin, Jenifer Matthews, and Nick Nelson of the Structural Competency Working Group ([structcomp.org](http://structcomp.org/)), in collaboration with Sonia Lee and Caitlin Ruppel of Health Outreach Partners ([outreach-partners.org](http://outreach-partners.org/)).

The Structural Competency Working Group’s efforts have been supported by the Berkeley Center for Social Medicine and Deborah Lustig as well as the University of California Humanities Research Institute. Josh Neff’s work on this project has been supported by the UCSF Resource Allocation Program for Trainees (RAPtr), the Greater Good Science Center Hornaday Fellowship, UC Berkeley-UCSF Joint Medical Program Thesis Grant, and the Helen Marguerite Schoeneman Scholarship.

Module 3: Responding to Harmful Structures in and Beyond the Clinic

| Content Time | 60 minutes |
| --- | --- |
| Learning Objective(s): | 1. To describe at least one historical or contemporary example of an intervention that addressed structural violence and vulnerability. 2. To define and understand six levels of intervention for addressing harmful social structures. 3. To identify a challenge and a strategy for at least one of the six levels of intervention that is applicable in a provider setting. 4. To define “Beloved Community” and articulate its importance for structurally competent practice. 5. To identify at least one intervention strategy to implement to address structural causes of ill health. |
| Methods of Instruction: | - Facilitator Instruction - Large Group Discussion - Individual and Group Activities |
| Sections: | 1. Structurally Competent Interventions 2. Levels of Intervention 3. Imagining Structural Interventions 4. Beloved Community 5. Putting Theory into Practice |
| Supplies: | - Flipchart - Markers - Tape - Appendix N: Facilitator Guidelines - Appendix O: Facilitator Preparation -Terms and Concepts - Appendix L: Training Workbook - Appendix K: Slides. Module 3 and Wrap Up |
| Required Reading for Facilitator: | - Baum, F., & Fisher, M. (2014). Why Behavioral Health Promotion Endures Despite Its Failure to Reduce Health Inequities. *Sociology of Health and Illness*, 36(2), 213-225. - Geiger, J. H. (1984). Community health centers: health care as an instrument of social change. In Sidel, V.W., & Sidel, R. (Eds.) *Reforming Medicine: Lessons of the Last Quarter Century* (pp. 11-32) New York, NY: Pantheon Books. - Messac, L., Ciccarone, D., Draine, J., & Bourgois, P. (2013). The Good-Enough Science-and-Politics of Anthropological Collaboration with Evidence-Based Clinical Research: Four Ethnographic Case Studies. *Social Science and Medicine,* 99, 176-186. - Nelson, A. (2016). The longue durée of Black Lives Matter. *American Journal of Public Health*, 106(10), 1734–1737. - Structural Competency Working Group (Producer). (January 2016). Structural Competency Training Video Part 3. Available from the Structural Competency Working Group, Berkeley, California. |
| Handout(s): | - Components of Structural Competency (Appendix L, pg. 9) - Levels of Intervention (Appendix L: pgs. 10-12) |

Module 3 — Section 1: Structurally Competent Interventions

**Time: 15 minutes**

**Learning Objective:** To describe at least one historical or contemporary example of an intervention that addressed structural violence and vulnerability.

**Supplies:**

- Flipchart paper
- Flipchart markers
- Appendix L: Participant Workbook
- Appendix K Slides: 2-5

**Handout(s):**

- None

**Preparation:**

- Complete the required reading for the module prior to presenting.
- Write the key structural competency concepts and definitions discussed in this section on flipchart paper prior to presenting. Display the definitions on the wall in the room.
  - Key concepts: Levels of Intervention
- Review all handouts for this section prior to presenting the information. Refer to them in the participant workbook, as necessary, throughout the module.

1. **What are Structurally Competent Interventions? (15 minutes)**
   1. **Introduction (1 minute) (Appendix K: Slides 2-3):** Introduce the section and its learning objectives.
      - We began the training by defining structural violence and structural vulnerability and discussing how these inequalities are naturalized through implicit frameworks.
      - Then we reviewed how encounters between providers and patients are affected by structural violence and structural vulnerability, and the role of structural competency in addressing these structures.
      - In the previous section, we defined structural competency and reviewed what this new framework attempts to achieve.
      - In this section of the training we will talk through the action that we can take in response to our recognition of harmful structures.
      - To begin, I will present two examples of interventions that demonstrate structural competency.
   2. **The Integrated Soft Tissue Infection Service Clinic (7 minutes) (Appendix K: Slide 4):** Explain the intervention.
      - This example is taken from the book, *Righteous Dopefiend*, written by anthropologists, Philippe Bourgois and Jeffery Schonberg.
      - During their ethnographic fieldwork with homeless heroin users in San Francisco in the early 2000s, Philippe Bourgois and Jeffery Schonberg learned that injection drug users were experiencing systemic mistreatment by surgeons at the county hospital.
      - Specifically, the two anthropologists observed that when injection drug users went to the county hospital with injection-related skin abscesses, the surgeons who treated them were removing extensive amounts of tissue from around the wound.
      - This medical procedure – called debridement – is used to prevent necrotizing fasciitis, a rare but deadly health issue; however, the anthropologists found that the surgeons were not providing appropriate anesthesia for this procedure, and that they sometime cut more widely and deeply than was medically necessary. The anthropologists concluded that this was purposefully punitive.
      - Furthermore, the anthropologists found that as a result of this treatment, many of the homeless heroin users lanced (i.e.: cut open) their own abscesses outside of the county hospital, on their own or for one another, in highly unsterile settings.
      - In response, the two anthropologists recruited a family physician from the University of California San Francisco (UCSF) to provide treatment to the homeless injection drug users. The recruited physician, Dan Ciccarone, was known for providing compassionate care to injection drug users.
      - Dr. Ciccarone began providing sterile, minimally-invasive incision-and-drainage procedures in the homeless encampments where the two anthropologists were conducting fieldwork. These treatments caused minimal pain and scarring, and effectively healed all abscesses for which he performed the procedure.
      - According to the two anthropologists, Dr. Ciccarone calculated that ‘soft-tissue infections’ were the largest single admissions category for the county hospital (more than 4,000 per year).
      - The two anthropologists and Dr. Ciccarone presented their findings and captured the attention of the surgeons at the county hospital where the invasive procedures had been done.
      - The county hospital surgeons conducted a study to investigate the effects of providing compassionate care to injection drug users, including the use of simple incision and drainage procedures rather than debridement.
      - The effects of the change were dramatic. Twelve months after the creation of an informal outpatient clinic, emergency department visits to the county hospital decreased 33.9%. Furthermore, inpatient operating room procedures at the county hospital decreased 71%. These reductions produced a net savings of approximately $8.77 million dollars for the hospital.
      - Importantly, patient satisfaction was measured at 86% and few patients were deemed “treatment failures” or “lost to follow-up”.
      - The surgeons from the county hospital published the quantitative findings of their study This practice became the established national standard of specialized outpatient care for underserved injection drug users (Messac et al., 2013).
      - as a result of the study, a formal “Integrated Soft-Tissue Infection Services” outpatient clinic was immediately founded in San Francisco.
      - This example demonstrates that observation and documentation of poor clinical practices at the individual and community level can lead to change in practice not only in one institution but across the entire U.S. healthcare system.
      - It is important to note here that while cost-ineffective practice changes should be made if they improve patient health outcomes, a change that is also cost-effective for providers or the health system will likely be easier to implement.
      - [*Ask participants if they have any questions*.]
   3. **The People’s Free Health Clinics of the Black Panther Party (7 minutes) (Appendix K: Slide 5):** Explain the intervention.
      - The content of this example comes from the book, *Body and Soul: The Black Panther Party and the Fight Against Medical Discrimination*, written by sociologist Alondra Nelson
      - The Black Panther Party (BPP) was founded in 1966 by Bobby Seale and Huey Newton. Based in Oakland, CA, the political organization was established to as a response to the economic and social oppression, or structural vulnerability, of African American communities.
      - Initially, a key focus of the BPP was offering legal armed surveillance of the police and, when needed, resistance to police brutality in African American communities in order to address racism and inequality.
      - Due to continued violent interactions with law enforcement, including incarceration, the Black Panther Party shifted the focus of its efforts towards the provision of social programs to address the fundamental needs of African American children and adults.
      - In 1970, the central committee of the Black Panther party mandated that all chapters establish People’s Free Medical Clinics
      - The People’s Free Health Clinics responded to structurally-rooted racial disparities in health and health care. Namely, the lack of access to medical care experienced by African American communities as well as the abuse and discrimination they experience in the American medical system.
      - The People’s Free Health Clinics were staffed by members of the Black Panther Party as well as volunteer health professionals, such as medical students, physicians, lab technicians and nurses. Many of the volunteers came from more privileged economic or social backgrounds and were required to attend classes where they read and discussed post-colonial writings. This helped to build solidarity between the volunteers, the BPP members and the communities accessing medical care at the clinics. In addition, the volunteers trained members of the community and the local BPP chapter to staff the PFHC.
      - The scope of services provided at the People’s Free Health Clinics were limited based on available financial support, donations, and volunteers; however, the clinics accomplished a great deal. Patients had access to basic health care services such as immunizations, screenings for Tuberculosis and lead poisoning, and screening for sickle-cell disease – a disease that disproportionately affects the African American community but was largely neglected by the American medical system at the time.
      - In addition, patient advocates at the People’s Free Health Clinics provided legal aid, housing assistance, financial support, translation services, and schooling to patients and their families, as it was possible.
      - The BPP’s People’s Free Health Clinic model is an example of structurally humble care: providers followed the lead of the community while at the same time sharing their expertise to build lasting community-based and driven change.
      - [*Ask participants if they have any questions and then conclude section one of module three*.]

Module 3 — Section 2: Levels of Intervention

**Time: 5minutes**

**Learning Objective:** To define and understand the six levels of intervention for addressing harmful social structures.

**Supplies:**

- Flipchart paper
- Flipchart markers
- Appendix L: Participant Workbook
- Appendix K: Slide 6

**Handout(s):**

- Levels of Intervention (Appendix L, pgs. 10-12)

**Preparation:**

- Complete the required reading for the module prior to presenting.
- Write the key structural competency concepts and definitions discussed in this section on flipchart paper prior to presenting. Display the definitions on the wall in the room.
  - Key concepts: Levels of Intervention
- Review all handouts for this section prior to presenting the information. Refer to them in the participant workbook, as necessary, throughout the module.

1. **Introduction to Levels of Intervention (5 minutes)**
   1. **Levels of Intervention Overview (5 minutes) (Appendix K: Slide 6):** Explain the levels of intervention.
      - In this section of the training we will talk through six levels of intervention that we can use to identify and implement action steps that respond to structural violence, structural vulnerability, and the naturalization of inequality.
      - The six levels of intervention that we will discuss today are: individual, interpersonal, clinic, community, research and policy.
      - You can find the information on this slide in your participant workbook on pages 10-12.
      - At the individual level we are referring to the ways that individuals think and talk about others and themselves; how they conceptualize society and their place in it; the assumptions, biases, and prejudices that we carry and the stories we believe about various groups of people.
      - This level influences our ability and willingness to act at all of the other levels, personally and professionally.
      - Individual level responses to structural violence include working to recognize and address one’s own implicit and explicit biases, learning about structural competency and the structural determinants of health, and recognizing implicit frameworks.
      - The interpersonal level builds on the individual level to consider the dynamics between people. For example, how you all as health care staff or students interact with one another, with patients, and with the broader community.
      - One example of an interpersonal level challenge may be the existence of a power imbalance between patients and providers, or even between providers based on training and skill set.
      - The BPP People’s Free Health Clinic provides an example of how this challenge may be addressed through patient advocates and the mutual sharing of knowledge between community and providers.
      - The clinic and institutional level of intervention addresses the organizational structures within an organization. Specifically, it is about identifying any structurally harmful issues within the institution or clinic that influence the delivery of care to patients and the broader community.
      - One challenge that may occur at this level is that the institution has not adapted available services to meet the needs of the patient population. An intervention would be to conduct a needs assessment to identify unmet health and social service needs and inform service delivery.
      - The Integrated Soft Tissue Infection Service Clinic presented earlier was an example of an interpersonal level intervention.
      - The community level can be defined as the organizations and informational networks within a community, including community leaders and members.
      - One example of a community level structural challenge may be limited access to healthy food. A clinic could work with local organizations to build community food gardens and provide nutrition and cooking classes.
      - The policy level considers how institutions develop rules and regulations in managing health in the public sphere. It pertains to local, state, national and global laws and policies, including policies regarding the allocation of resources.
      - Finally, the research level is defined as academic and organizational research that creates, organizes and integrates new knowledge.
      - [*Optional talking points below.*]
      - Why is it that in the U.S. health care and social support systems, randomized clinical trials are held as the highest level of evidence, and what factors may we be missing when we structure evidence in this manner?
      - High-quality evidence produced through rigorously-controlled randomized clinical trials may provide real, reliable solutions to certain medical problems.
      - On the other hand, rigorously-controlled randomized clinical trials may not be able to explain all factors that influence the prevalence, persistence, or burden of certain diseases.
      - For example, structural racism and other social causes of illness cannot be studied through a randomized clinical trial because they cannot be controlled for or randomized.
      - In addition, rigorously-controlled randomized clinical trials have restrictive inclusion criteria. For example, a patient with comorbidities cannot participate. This makes findings from the research less useful for application to a large portion of the general population.
      - Furthermore, exclusion or statistical analysis of data may hide or erase socially structured differences between subjects participating in the research study.
      - Finally, randomized clinical trials are expensive. These days, funding often comes from pharmaceutical companies, which influences what is studied or not studied.
      - Because evidence-based medicine is limited in the types of knowledge that it can produce, and may be subject to commercial or scientific interests, it can be a key player in the process of naturalizing inequality, and therefore may warrant intervention.
      - [*Ask participants if they have any questions and then conclude section two of module three*.]

Module 3 — Section 3: Imagining Structural Interventions

**Time: 25 minutes**

**Learning Objective:** To identify a challenge and a strategy for at least one of the six levels of intervention that is applicable at a provider setting.

**Supplies:**

- Flipchart paper
- Flipchart markers
- Appendix L: Participant Workbook
- Appendix K: Slides 7-8

**Handout(s):**

- Levels of Intervention (Appendix L, pgs. 10-12)

**Preparation:**

- Complete the required reading for the module prior to presenting.
- Write the key structural competency concepts and definitions discussed in this section on flipchart paper prior to presenting. Display the definitions on the wall in the room.
  - Key concepts: Levels of Intervention
- Review all handouts for this section prior to presenting the information. Refer to them in the participant workbook, as necessary, throughout the module.

1. **Applying the Levels of Intervention (25 minutes)**
   1. **Introduction (5 minutes) (Appendix K: Slide 7):** Introduce the section.
      - We will conclude this section of the training by discussing examples of structurally-informed interventions.
      - Specifically, we will apply the information about the six levels of intervention to the patient case study from the first section of the training.
   2. **Partner and Group Discussion (20 minutes) (Appendix K: Slide 8):** Introduce and facilitate the activity.
      - Spend the next 15 discussing the prompts on this slide with the person next to you.
      - For the level of intervention that you have been assigned:
        1. Write down at least one structurally competent intervention that is applicable in the case of the corn farmer patient that is something you have either experienced or heard about happening.
        2. In addition, write down at least one structurally competent intervention that is applicable in the case of the corn farmer patient that is something that you would do if you had a “magic wand” to address issues at your assigned level of intervention.
      - [*Assign levels of intervention to the participants*.]
      - Let’s spend the next 10 minutes talking together about the structurally competent interventions that you all discussed.
      - [*Ask participants to share 1-2 structural interventions that they have identified for their assigned level of intervention. Facilitate conversation and questions and answers, as needed.]*
      - [*Optional talking points below.*]
      - Let’s review examples of interventions created by the people who developed the structural competency training.
      - At the individual level, providers can educate themselves and work against internalized implicit and explicit biases, such as anti-fat bias or judgements about “noncompliance.” In addition, healthcare professionals can engage in training and continued learning about social determinants of health.
      - At the interpersonal level, providers can approach patients without blame or judgement.
      - At the clinical level, providers can connect patients to locally available social services, such as free or reduced cost medication programs or housing services.
      - At the community level, providers can collaborate with their local partners and community members to organize and advocate for improved access to affordable food options, such as through community gardens.
      - At the policy level, providers can participate in collective efforts to promote healthy food policy, such as through reform of the farm bill. At present, the farm bill subsidizes agribusiness corn; however, it could instead subsidize the production of healthy, diverse crops.
      - In addition, at the policy level, providers can join in existing advocacy and organizing efforts around the establishment of universal child care and/or universal basic income. This slide has two articles exploring the potential for universal basic income to address health disparities.
      - Finally, at the research level, providers can change needs assessments to include questions that seek to identify structural forces on health disparities.
      - [*Ask participants if they have any questions and then conclude section three of module three.*]

Module 3 – Section 4: Beloved Community

**Time: 5 minutes**

**Learning Objective:** To define “Beloved Community” and articulate its importance for structurally competent practice.

**Supplies:**

- Flipchart paper
- Flipchart markers
- Appendix L: Participant Workbook
- Appendix K: Slides 9-14

**Handout(s):**

- None

**Preparation:**

- Complete the required reading for the module prior to presenting.
- Write the key structural competency concepts and definitions discussed in this section on flipchart paper prior to presenting. Display the definitions on the wall in the room.
  - Key concepts: Beloved Community
- Review all handouts for this section prior to presenting the information. Refer to them in the participant workbook, as necessary, throughout the module.

1. **Beloved Community (5 minutes)**
   1. **Introduction (1 minute) (Appendix K: Slide 9):** Introduce the section.
      - To conclude today’s training, we will focus on a vision of the future that we would like to create, and on actions that you can take, individually and collectively, to enact structural competency in in healthcare.
      - First, we will discuss the concept of Beloved Community. Then, we will walk through three principles of action in order to conclude the day with the creation of an individual action plan.
      - We all have different frameworks that help us to understand the world, and to situate ourselves within communities working on social justice issues. One framework that Shirley Strong, the Chief Diversity Officer at Samuel Merritt University, introduced to the Structural Competency Working Group, is the concept of beloved community. This concept has inspired many of us working on structural competency and may be helpful to you. We hope this will also help you think about other frameworks that you find supportive and inspiring.
   2. **Beloved Community (4 min) (Appendix K: Slides 10-14):** Define the key concept and present examples.

[**Appendix K: Slide 10**]

- - - [*Review working definition of beloved community.]* The working definition of beloved community we are using is [read from slide] an inclusive, interconnected consciousness, based on love, justice, compassion, responsibility, power, and a deep respect for all people, places, and things that radically transforms individuals and restructures institutions.

[**Appendix K: Slide 11**]

- - - [*Introduce Josiah Royce and his definition of beloved community*.] While many people connect the idea of beloved community to Martin Luther King Jr. Josiah Royce actually described it in the 1800’s. [*Read quote from slide.]*
    - This quote is especially useful because of the end “act so as to hasten its coming.” It says that inherent to descriptions of beloved community is the need for those involved to take action.

[**Appendix K: Slide 12**]

- - - [*Review MLK’s vision of beloved community and related quotes*.]
    - Martin Luther King Jr. also powerfully took up the idea of the beloved community. *[Read MLK quotes from slide.]*

[**Appendix K: Slide 13**]

- - - Grace Lee Boggs asked, “I wonder how things would have been different, both intraracially and interracially, had we been able to combine Malcolm’s militancy with Martin’s Beloved Community?”

[**Appendix K: Slide 14**]

- - - Consider what would be different in our social movements today if we had been able to work together from our different positions in terms of how change occurs
    - Grace Lee Boggs was a Detroit-based philosopher, civil rights activist, and writer who dedicated her life to the fight for racial justice

[**Appendix K: Slide 15**]

- We encourage you to learn about social movement leaders whose visions speak to you, and to connect to active work going on around you. Collaboration and community building must be a crucial tenant of any structurally competent intervention.
- [*Ask participants if they have any questions and then conclude section four of module three.*]

1. **Summary Slide: Module 3 Manual**

[**Appendix K: Slide 16**]

- In this Module we explored how a structural framework can lead to new ways of thinking about interventions. We also discussed how historical and contemporary social movements inspire our current work.
- The exercises in this module can remind us that there are many levels at which one can intervene using a structural lens.
- Finally, it’s important to remember that we do not have to, nor should we, do this work alone. Community building is a powerful force for change and can also help prevent burn-out and isolation.
- *Ask for any questions or reflections then conclude Module 3*
