# Supplementary material for: Structural Competency: Curriculum for Medical Students, Residents, and Interprofessional Teams on the Structural Factors That Produce Health Disparities
Source: MedEdPORTAL. 2020 Mar 13;16:10888. doi: 10.15766/mep_2374-8265.10888 (PMC7182045; doi:10.15766/mep_2374-8265.10888)
Supplement: Supplementary file 1 — A. Manual Background Info.docx B. Manual Intro.docx C. Manual Module 1.docx D. Manual Module 2.docx E. Manual Module 3.docx F. Manual Conclusion and Evaluation.docx G. Supplemental Reading List.docx H. Training Slides Intro.pptx I. Training Slides Module 1.pptx J. Training Slides Module 2.pptx K. Training Slides Module 3.pptx L. Participant Workbook.pdf M. Posttraining Survey.pdf N. Facilitator Guidelines.docx O. Facilitator Preparation - Terms and Concepts.docx P. Participant Sign-in Sheet.docx [file mep-16-10888-s001.zip › F. Manual Conclusion and Evaluation.docx]

STRUCTURAL COMPETENCY:

A Framework for Recognizing & Responding to Social, Political & Economic Structures to Improve Health


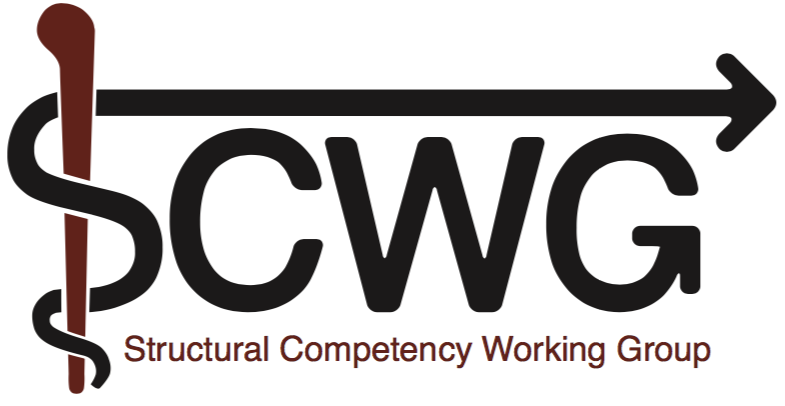


TRAINING CURRICULUM: CONCLUSION & EVALUATION

Updated September 2018

Copyright:

The Structural Competency Working Group ([www.structcomp.org](http://www.structcomp.org)), in order to promote the widest possible dissemination of health curricula, has adopted an open copyright policy for its structural competency training materials. This means that we will grant permission to translate, adapt, or borrow our materials without charging fees or royalties under the following conditions:

- that you credit the Structural Competency Working Group for any borrowed/adapted Working Group materials and inform your audience about who we are and how to learn more about our organization ([structcomp.org](http://structcomp.org)). We suggest the following attribution (and request that it appear on any copyright page):
  - “*These materials have been [borrowed][adapted] from the Structural Competency Working Group,* [*www.structuralcompetency.org*](http://www.structuralcompetency.org)*; you can contact the Group at* [*structuralcompentency@gmail.com*](mailto:structuralcompentency@gmail.com)*”;*
- that your materials are distributed at no cost (or for your production cost only), that is, not-for-profit;
- that you allow others to reproduce/ adapt your edition or adaptation with no fees, royalties, etc. so long as they also do so at no cost or for production cost only, that is, not-for-profit;
- that you provide us with digital versions of your materials (including PDF and/or Microsoft Word files) and work with us so that we can host it on our website;
- that you send us your contact information so we can post it on our website and provide it to people who want to contact you about your edition, adaptation or publication;
- that you contact and stay in touch with the Structural Competency Working Group so that we can learn about your project and make sure you are using the most up-to-date materials.

If you decide to begin translating, adapting, or borrowing our materials, please use the most recently updated versions. Please contact us at [structuralcompetency@gmail.com](mailto:structuralcompetency@gmail.com) to find out if we are currently working on updating our materials, and if anyone else is working on a project similar to your own.

This training curriculum was prepared by Josh Neff, Seth M. Holmes, Kelly R. Knight, Shirley Strong, Ariana Thompson-Lastad, Cara McGuinness, Laura Duncan, Michael J. Harvey, Nimish Saxena, Katiana L. Carey-Simms, Alice Langford, Sara Minahan, Shannon Satterwhite, Lillian Walkover, Jorge De Avila, Brett Lewis, Gregory Chin, Jenifer Matthews, and Nick Nelson of the Structural Competency Working Group ([structcomp.org](http://structcomp.org/)), in collaboration with Sonia Lee and Caitlin Ruppel of Health Outreach Partners ([outreach-partners.org](http://outreach-partners.org/)).

The Structural Competency Working Group’s efforts have been supported by the Berkeley Center for Social Medicine and Deborah Lustig as well as the University of California Humanities Research Institute. Josh Neff’s work on this project has been supported by the UCSF Resource Allocation Program for Trainees (RAPtr), the Greater Good Science Center Hornaday Fellowship, UC Berkeley-UCSF Joint Medical Program Thesis Grant, and the Helen Marguerite Schoeneman Scholarship.

Conclusion and Evaluation

| Content Time | 10 minutes |
| --- | --- |
| Learning Objective(s): | 1. To solicit participant feedback to inform the delivery of future trainings. |
| Methods of Instruction: | - Facilitator Instruction - Large Group Discussion |
| Sections: | 1. Closing Discussion and Training Evaluation |
| Supplies: | - Appendix N: Facilitator Guidelines - Appendix O: Facilitator Preparation -Terms and Concepts - Appendix K: Slides 17-20 |
| Required Reading for Facilitator: | - N/A |
| Handout(s): | - Appendix M: Post-Training Survey |

Conclusion: Section 1: Closing Discussion & Evaluations

**Time: 10 minutes**

**Learning Objective:** To provide an opportunity for participants to ask questions and to offer feedback on the training experience.

**Supplies:**

- Appendix K: Slides 17-20

**Handout(s):**

- Appendix M: Post-Training Survey

**Preparation:**

- Review all handouts for this section prior to presenting the information.

1. **Closing Discussion (6 minutes)**
   1. **Group Discussion (5 minutes) (Appendix K: Slide 17):** Facilitate a reflection exercise to close out the training.
      - I would like to conclude the training with a discussion and reflection on how, with the knowledge gained today, you all can work together to cultivate structural humility and build a beloved community through structural competency.
      - Take 5 minutes and write down your thoughts on the day. What were the most important points you’d like to take away?
      - [*Ask participants if they are willing to share 1-2 key takeaways with the group*.]
   2. **Wrap-Up and Q&A Session (1 minute) (Appendix K: Slides 18-19):** Conclude the training.

[Appendix K: Slide 18]

- - - Thank you for participating in this Structural Competency training.
    - We appreciate your willingness to participate in a dynamic learning environment and the contributions that you offered throughout the session.
    - *[Optional: briefly remind participants what content was covered during the training.]*

[Appendix K: Slide 19]

- - - On this slide you can find our contact information if you would like to contact us to request additional information or to continue the conversation that we began today.
    - *[Ask participants if they have any outstanding questions.]*

1. **Training Evaluation (4 minutes)**
   1. **Evaluation (4 minute) (Appendix K: Slide 20):** Administer the training evaluations (Appendix M: Post-Training Survey).
      - You all ought to have received a post-training survey. Please take the next few minutes to complete the form. You can return this to our facilitators as you leave the training. Thank you!
