# Supplementary material for: Structural Competency: Curriculum for Medical Students, Residents, and Interprofessional Teams on the Structural Factors That Produce Health Disparities
Source: MedEdPORTAL. 2020 Mar 13;16:10888. doi: 10.15766/mep_2374-8265.10888 (PMC7182045; doi:10.15766/mep_2374-8265.10888)
Supplement: Supplementary file 1 — A. Manual Background Info.docx B. Manual Intro.docx C. Manual Module 1.docx D. Manual Module 2.docx E. Manual Module 3.docx F. Manual Conclusion and Evaluation.docx G. Supplemental Reading List.docx H. Training Slides Intro.pptx I. Training Slides Module 1.pptx J. Training Slides Module 2.pptx K. Training Slides Module 3.pptx L. Participant Workbook.pdf M. Posttraining Survey.pdf N. Facilitator Guidelines.docx O. Facilitator Preparation - Terms and Concepts.docx P. Participant Sign-in Sheet.docx [file mep-16-10888-s001.zip › G. Supplemental Reading List.docx]

Appendix G: Supplemental Readings

Baum, F., & Fisher, M. (2014). Why Behavioral Health Promotion Endures Despite Its Failure to Reduce Health

Inequities. *Sociology of Health and Illness*, 36(2), 213-225.

Bourgois, P., Holmes, S. M., Sue, K., & Quesada, J. (2017). Structural Vulnerability: Operationalizing the Concept to

Address Health Disparities in Clinical Care. *Academic Medicine*, 92(3), 299-307.

Coates, T. N. (2014, June). The Case for Reparations. *The Atlantic.* Retrieved from

<http://www.theatlantic.com/magazine/archive/2014/06/the-case-for->reparations/361631/

Farmer, P. E., Nizeye, B., Stulac, S., & Keshavjee, S. (2006). Structural violence and clinical medicine. *Public Library*

*of Science Medicine*, *3*(10), e449.

Gregg, J., & Saha, S. (2006). Losing Culture on the Way to Competence: The Use and Misuse of Culture in Medical

Education. *Academic Medicine*, 81(6), 542-546.

Martin, N., & Montagne, R. (2017, December 7)*.* Black Mothers Keep Dying After Giving Birth. Shalon Irving’s Story

Explains Why. *National Public Radio*. Retrieved from <https://www.npr.org/2017/12/07/568948782/black-mothers-keep-dying-after->giving-birth-shalon-irvings-story-explains-why.

Messac, L., Ciccarone, D., Draine, J., & Bourgois, P. (2013). The Good-Enough Science-and-Politics of

Anthropological Collaboration with Evidence-Based Clinical Research: Four Ethnographic Case Studies. *Social Science and Medicine,* 99, 176-186.

Metzl, J. M., & Roberts, D. E. (2014). Structural competency meets structural racism: race, politics, and the

structure of medical knowledge. *Virtual Mentor,* 16(9), 674-690.

Neff, J., Knight, K. R., Satterwhite, S., Nelson, N., Matthews, J., & Holmes, S. M. (2017). Teaching Structure: A

Qualitative Evaluation of a Structural Competency Training for Resident Physicians. *Journal of General Internal Medicine*, 32(4), 430-443.

Nelson, A. (2016). The longue durée of Black Lives Matter. *American Journal of Public Health*, 106(10), 1734–1737.

Rivkin-Fish, M. (2011). Learning the Moral Economy of Commodified Health Care: ‘Community Education,’ Failed

Consumers, and the Shaping of Ethical Clinician-Citizens. *Culture, Medicine and Psychiatry,* 35(2), 183-205.

Suarez-Orozco, C., Casanova, S., Martin, M., Katsiaficas, D., Cuellar, V., Smith, N. A., & Dias, S. A. (2015). Toxic Rain

in Class: Classroom Interpersonal Microaggressions. *Educational Researcher,* 44(3), 151-160.

Sue, D., Lin, A., Torino, G., Capodilupo, C., & Rivera, D. (2009). Racial Microaggressions and Difficult Dialogues on

Race in the Classroom. *Cultural Diversity and Ethnic Minority Psychology,* 15(2), 183-190.

Thackrah, R. D., & Thompson, S. (2013). Refining the concept of cultural competence: building on decades of

progress. *The Medical Journal of Australia,* 199 (1), 35-38.
