# Supplementary material for: Structural Competency: Curriculum for Medical Students, Residents, and Interprofessional Teams on the Structural Factors That Produce Health Disparities
Source: MedEdPORTAL. 2020 Mar 13;16:10888. doi: 10.15766/mep_2374-8265.10888 (PMC7182045; doi:10.15766/mep_2374-8265.10888)
Supplement: Supplementary file 1 — A. Manual Background Info.docx B. Manual Intro.docx C. Manual Module 1.docx D. Manual Module 2.docx E. Manual Module 3.docx F. Manual Conclusion and Evaluation.docx G. Supplemental Reading List.docx H. Training Slides Intro.pptx I. Training Slides Module 1.pptx J. Training Slides Module 2.pptx K. Training Slides Module 3.pptx L. Participant Workbook.pdf M. Posttraining Survey.pdf N. Facilitator Guidelines.docx O. Facilitator Preparation - Terms and Concepts.docx P. Participant Sign-in Sheet.docx [file mep-16-10888-s001.zip › H. Training Slides Intro.pptx]

## Slide 1
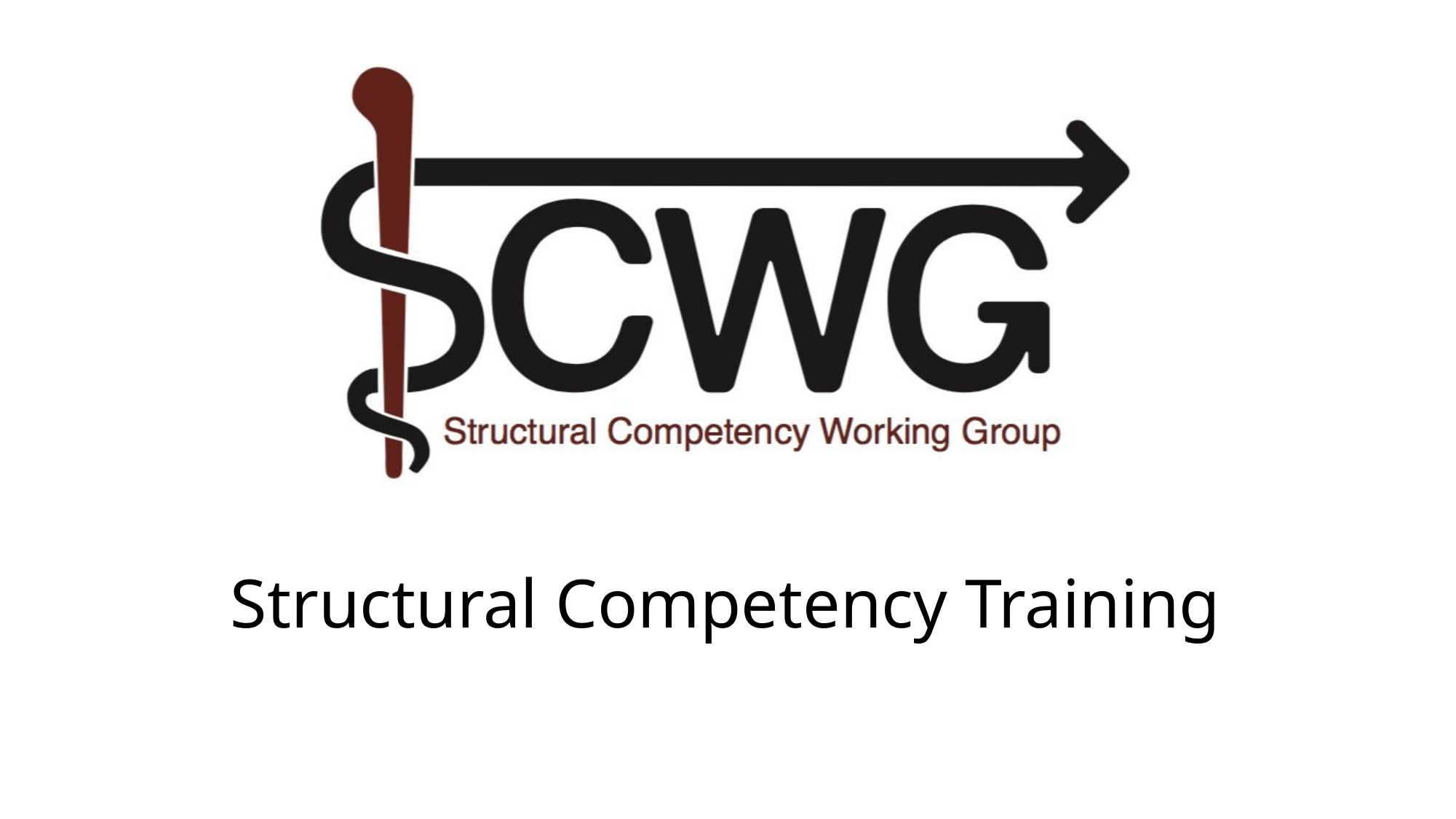

Structural Competency Training

## Slide 2
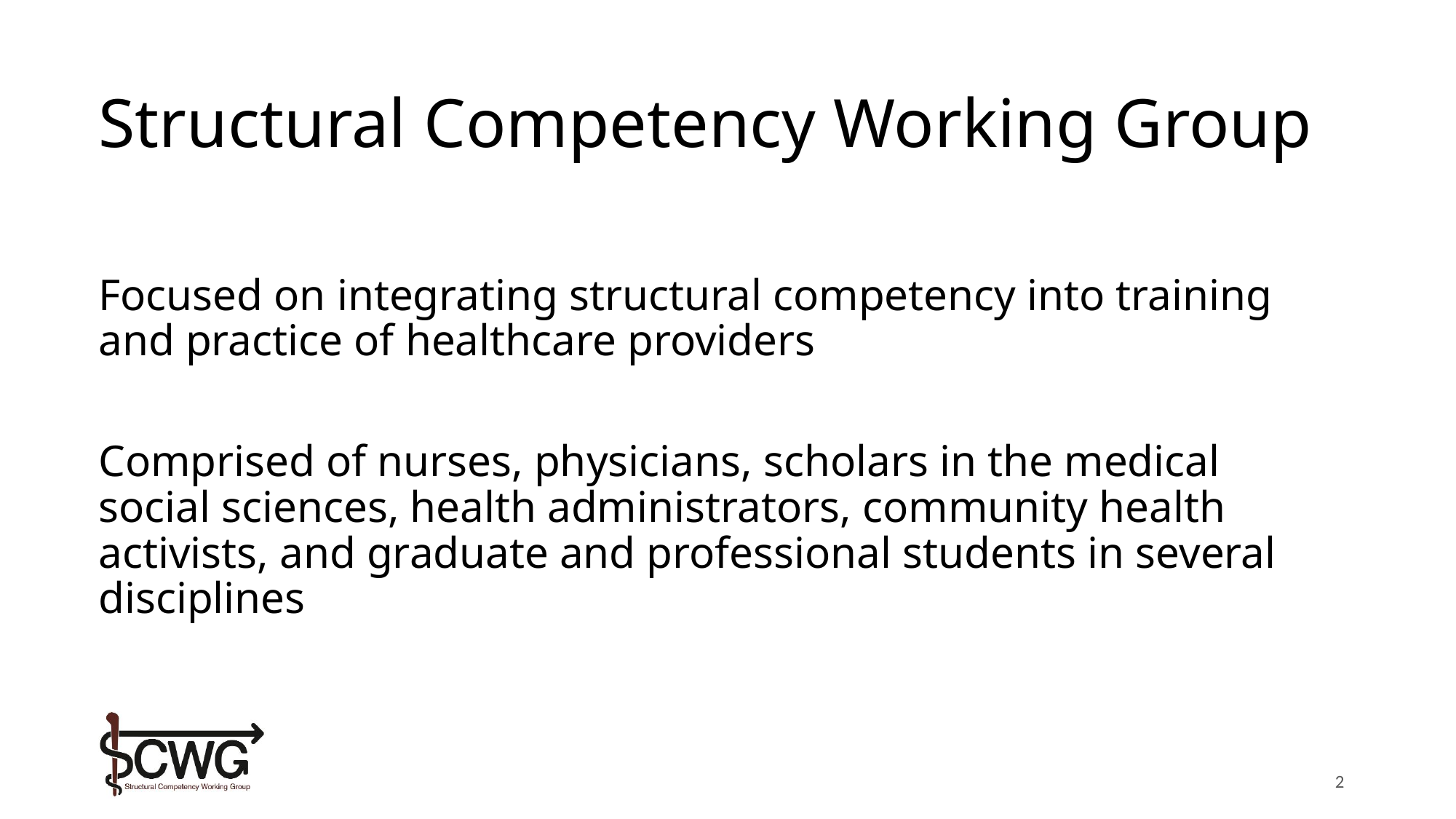

# Structural Competency Working Group
Focused on integrating structural competency into training and practice of healthcare providers
Comprised of nurses, physicians, scholars in the medical social sciences, health administrators, community health activists, and graduate and professional students in several disciplines
2

## Slide 3
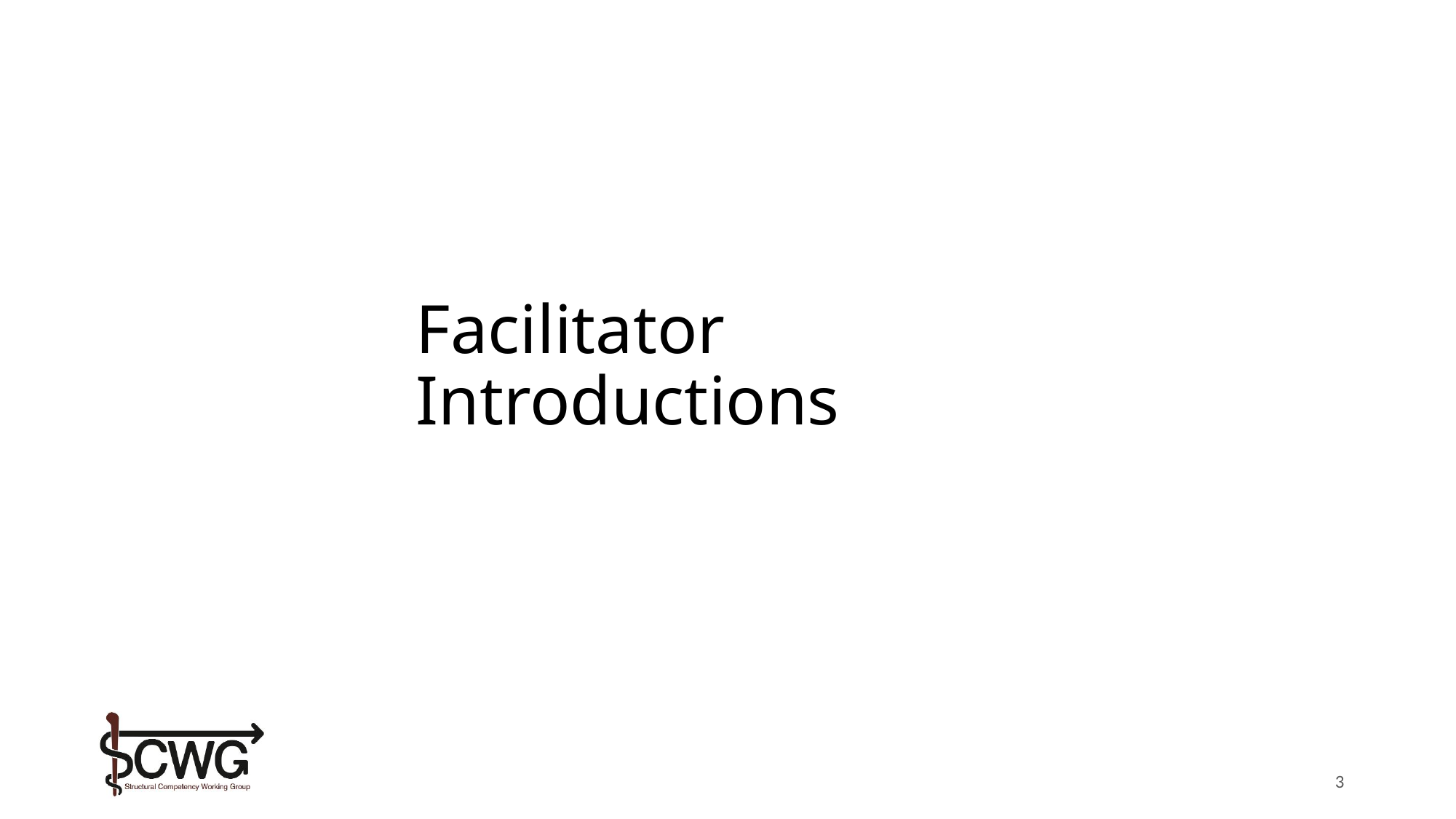

# Facilitator Introductions
3

## Slide 4
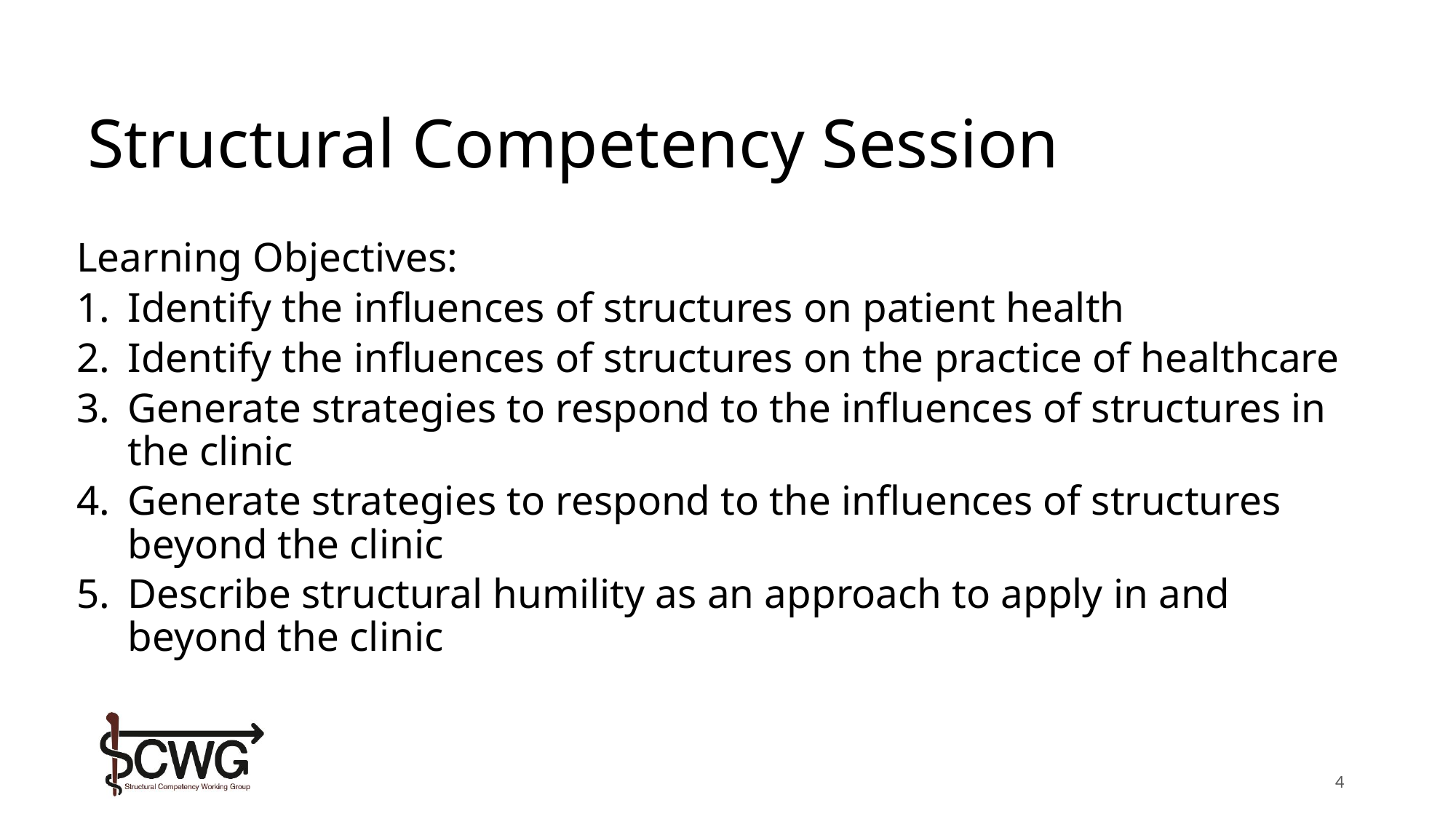

# Structural Competency Session
Learning Objectives:
Identify the influences of structures on patient health
Identify the influences of structures on the practice of healthcare
Generate strategies to respond to the influences of structures in the clinic
Generate strategies to respond to the influences of structures beyond the clinic
Describe structural humility as an approach to apply in and beyond the clinic
4

## Slide 5
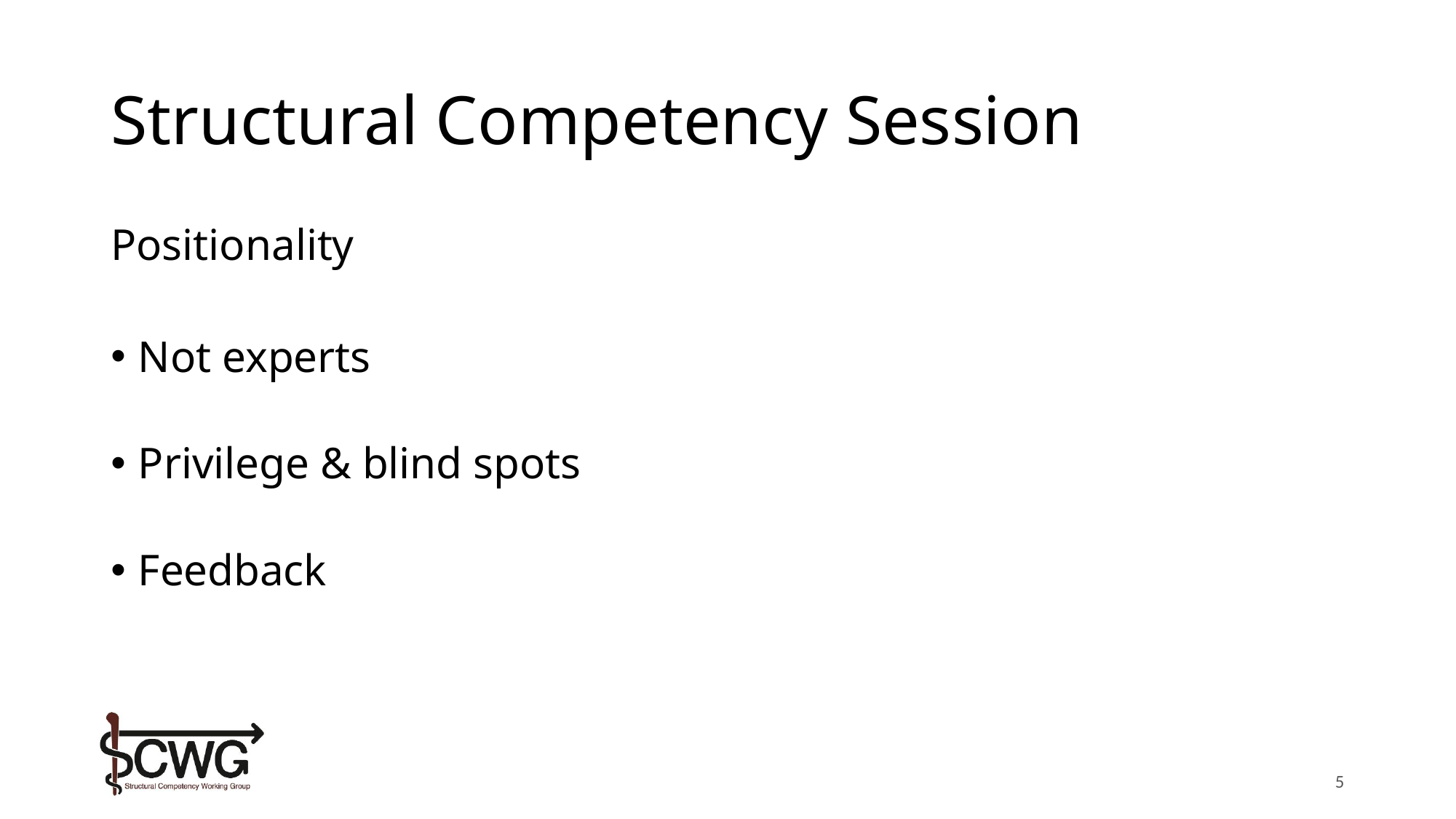

# Structural Competency Session
Positionality
Not experts
Privilege & blind spots
Feedback
5

## Slide 6
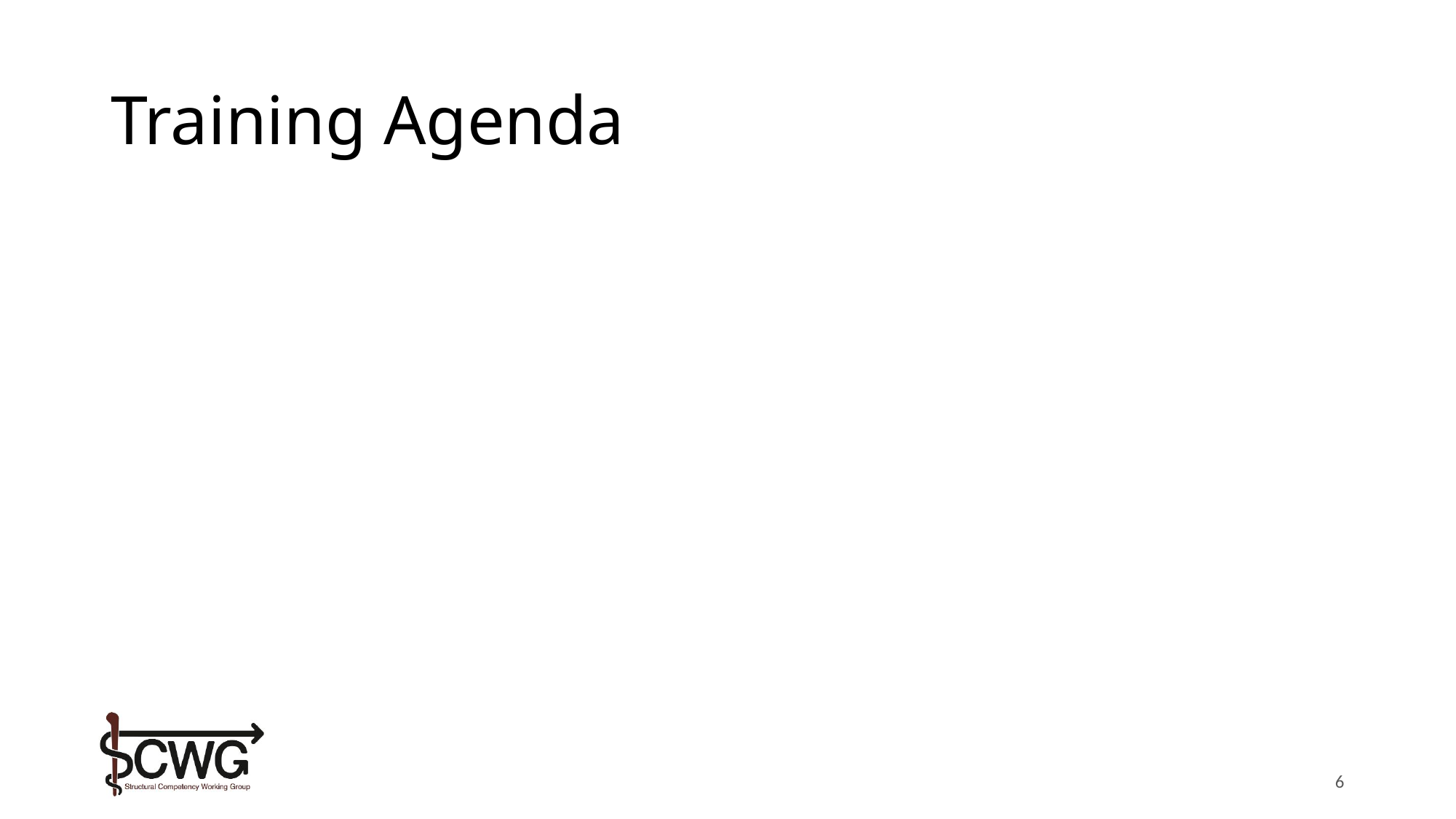

# Training Agenda
6

## Slide 7
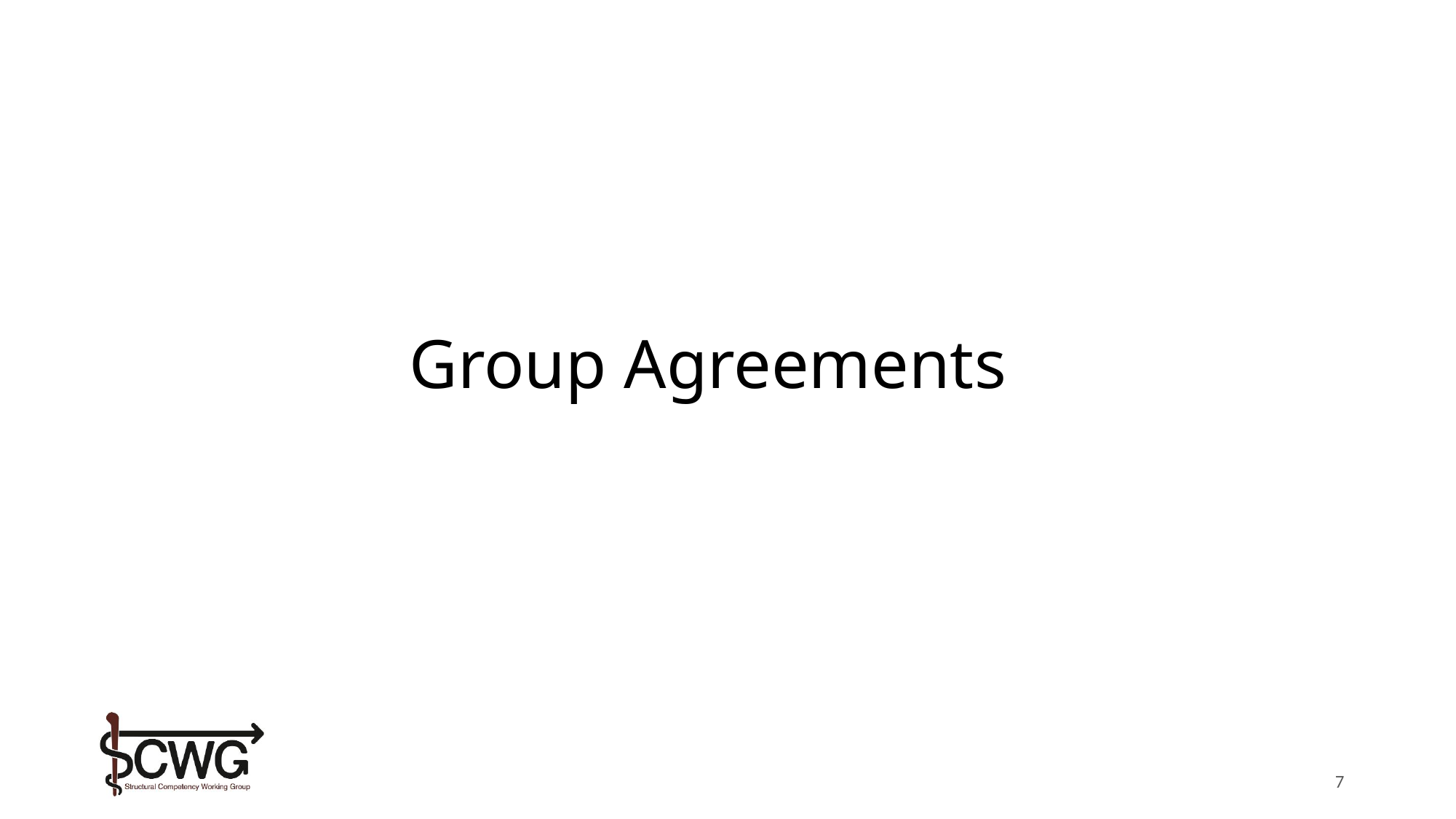

# Group Agreements
7
