# Supplementary material for: Structural Competency: Curriculum for Medical Students, Residents, and Interprofessional Teams on the Structural Factors That Produce Health Disparities
Source: MedEdPORTAL. 2020 Mar 13;16:10888. doi: 10.15766/mep_2374-8265.10888 (PMC7182045; doi:10.15766/mep_2374-8265.10888)
Supplement: Supplementary file 1 — A. Manual Background Info.docx B. Manual Intro.docx C. Manual Module 1.docx D. Manual Module 2.docx E. Manual Module 3.docx F. Manual Conclusion and Evaluation.docx G. Supplemental Reading List.docx H. Training Slides Intro.pptx I. Training Slides Module 1.pptx J. Training Slides Module 2.pptx K. Training Slides Module 3.pptx L. Participant Workbook.pdf M. Posttraining Survey.pdf N. Facilitator Guidelines.docx O. Facilitator Preparation - Terms and Concepts.docx P. Participant Sign-in Sheet.docx [file mep-16-10888-s001.zip › I. Training Slides Module 1.pptx]

## Slide 1
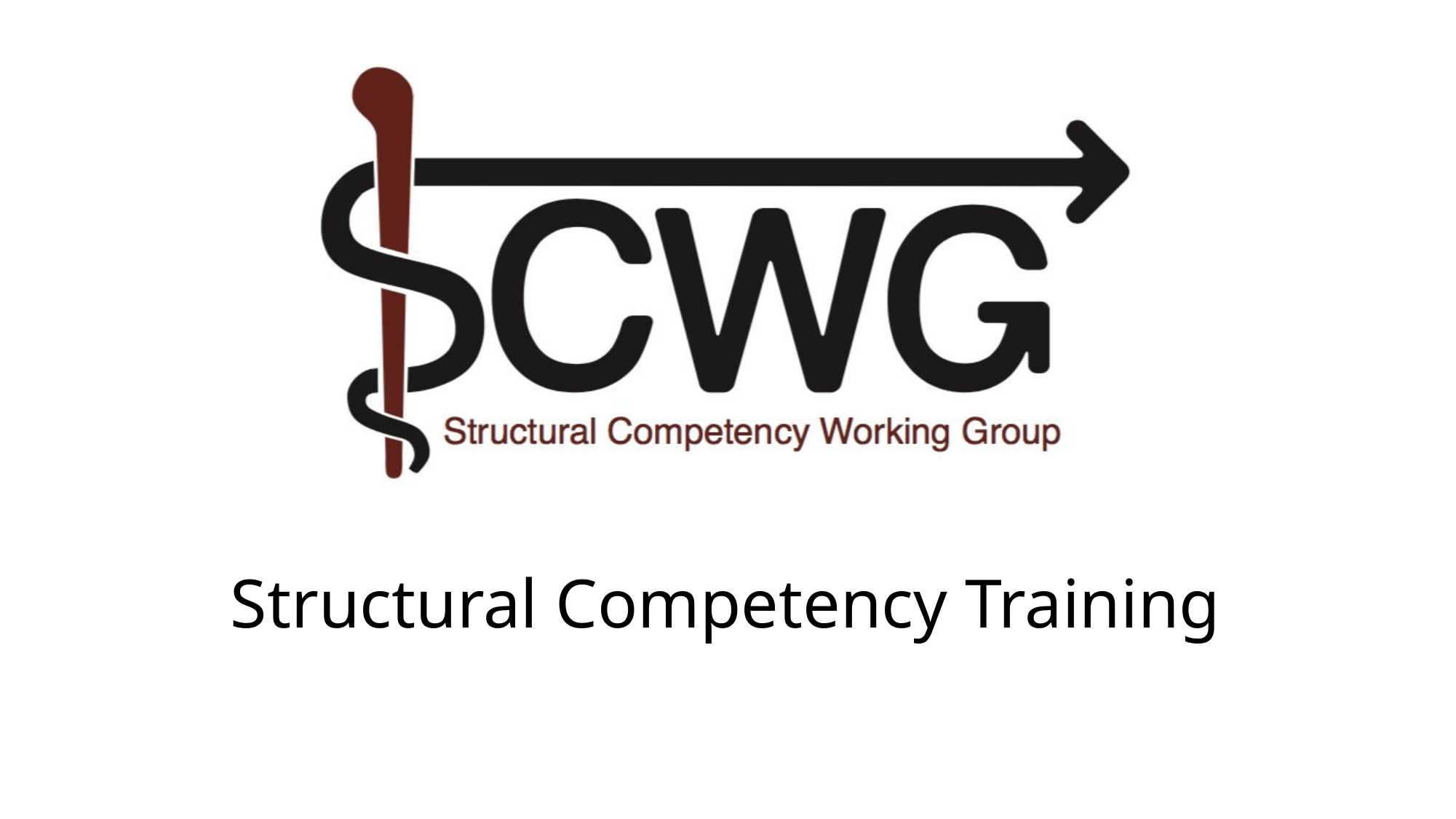

Structural Competency Training

## Slide 2
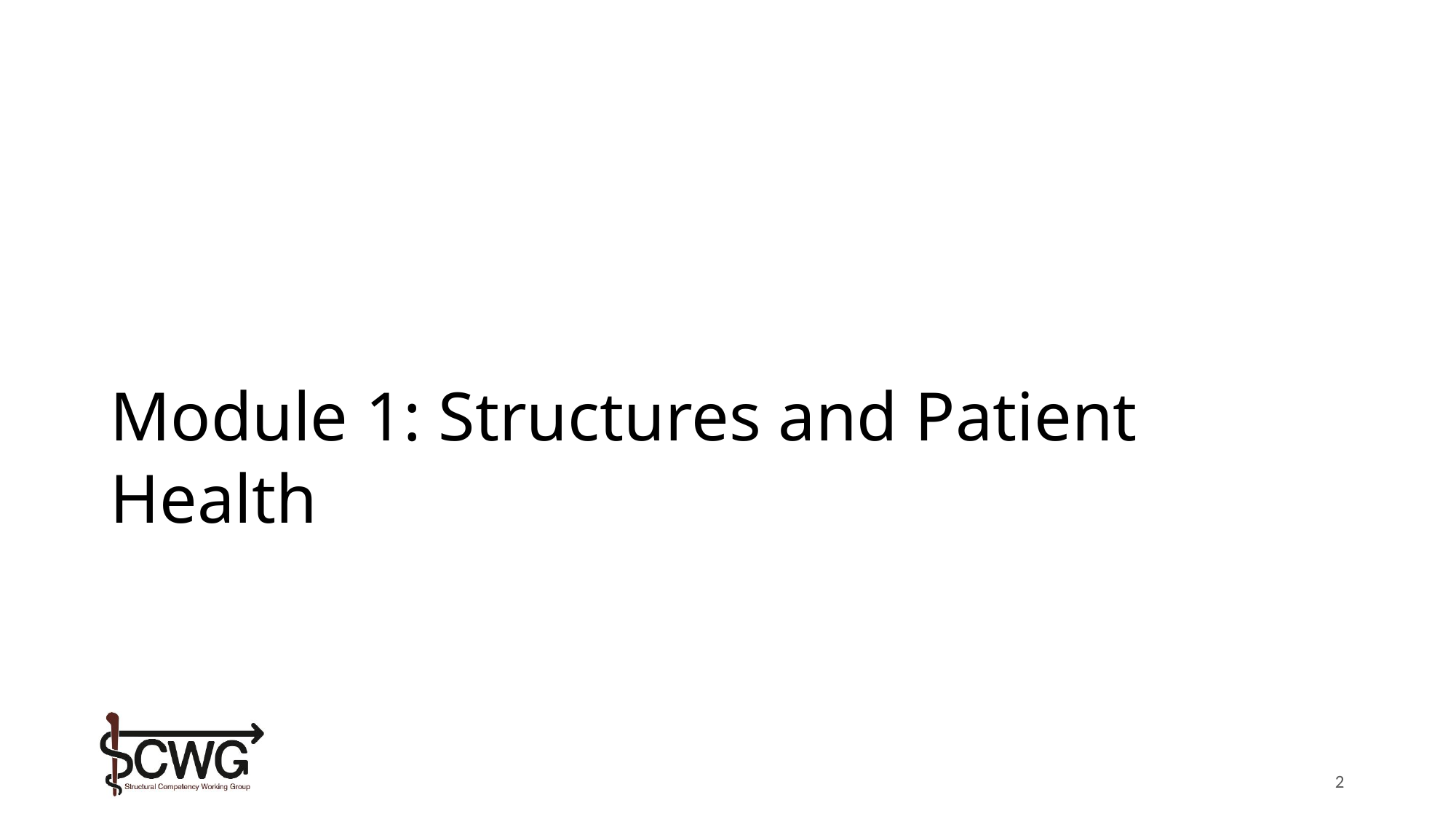

# Module 1: Structures and Patient Health
2

## Slide 3
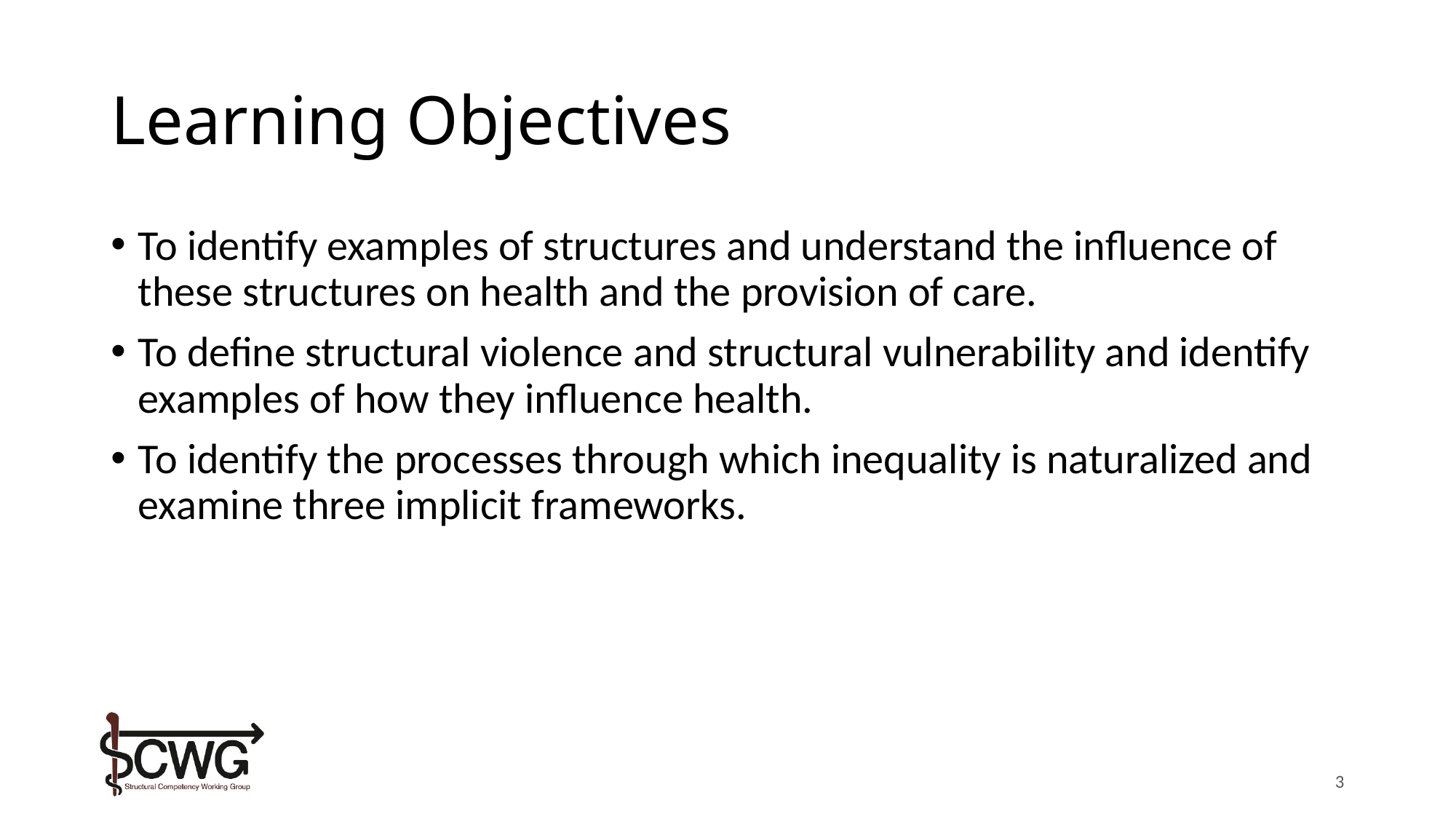

# Learning Objectives
To identify examples of structures and understand the influence of these structures on health and the provision of care.
To define structural violence and structural vulnerability and identify examples of how they influence health.
To identify the processes through which inequality is naturalized and examine three implicit frameworks.
3

## Slide 4
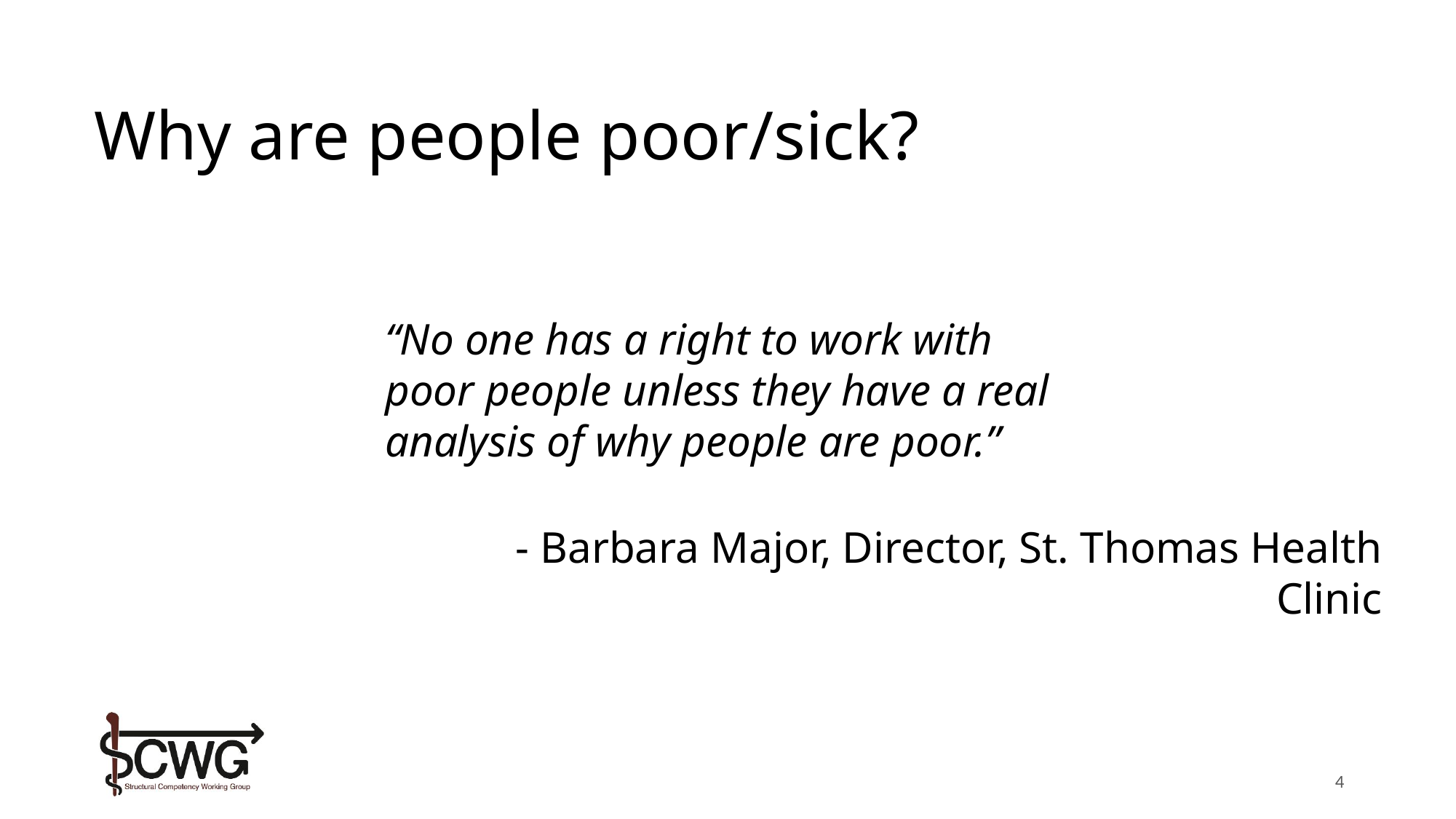

# Why are people poor/sick?
“No one has a right to work with poor people unless they have a real analysis of why people are poor.”
- Barbara Major, Director, St. Thomas Health Clinic
4

## Slide 5
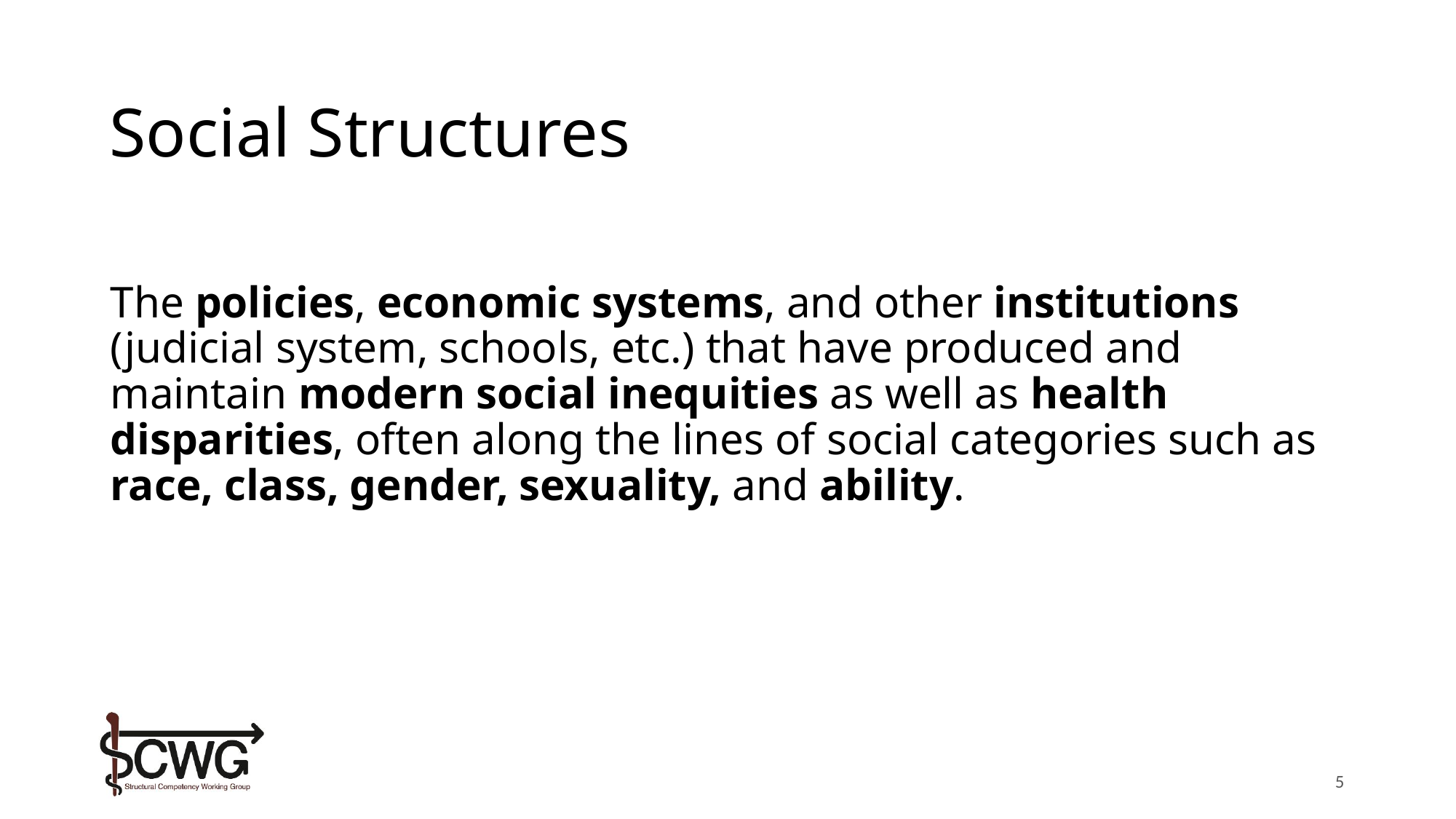

# Social Structures
The policies, economic systems, and other institutions (judicial system, schools, etc.) that have produced and maintain modern social inequities as well as health disparities, often along the lines of social categories such as race, class, gender, sexuality, and ability.
5

## Slide 6
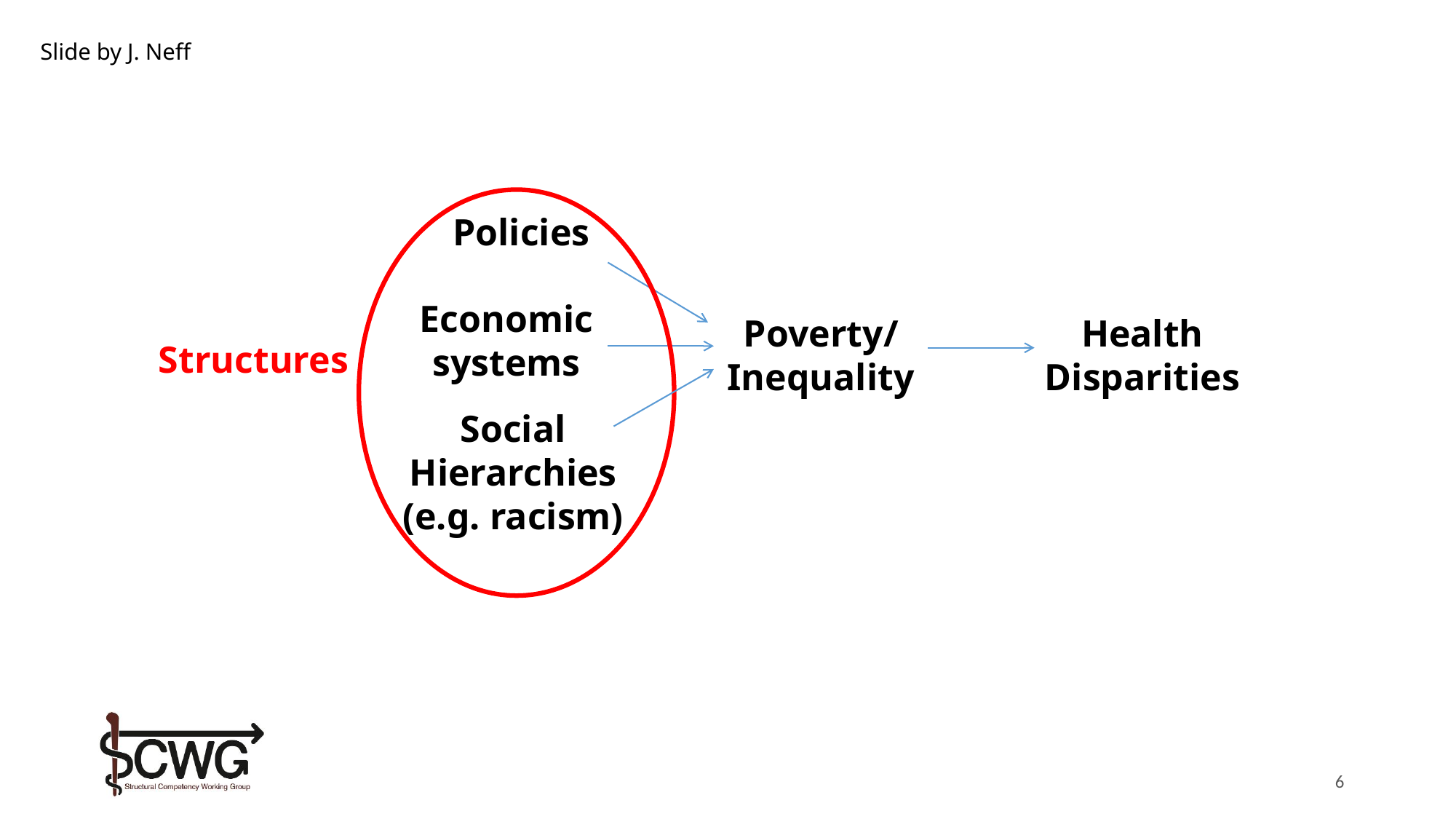

Slide by J. Neff
#
Structures
Policies
Economic systems
Poverty/ Inequality
Health Disparities
Social Hierarchies
(e.g. racism)
6

## Slide 7
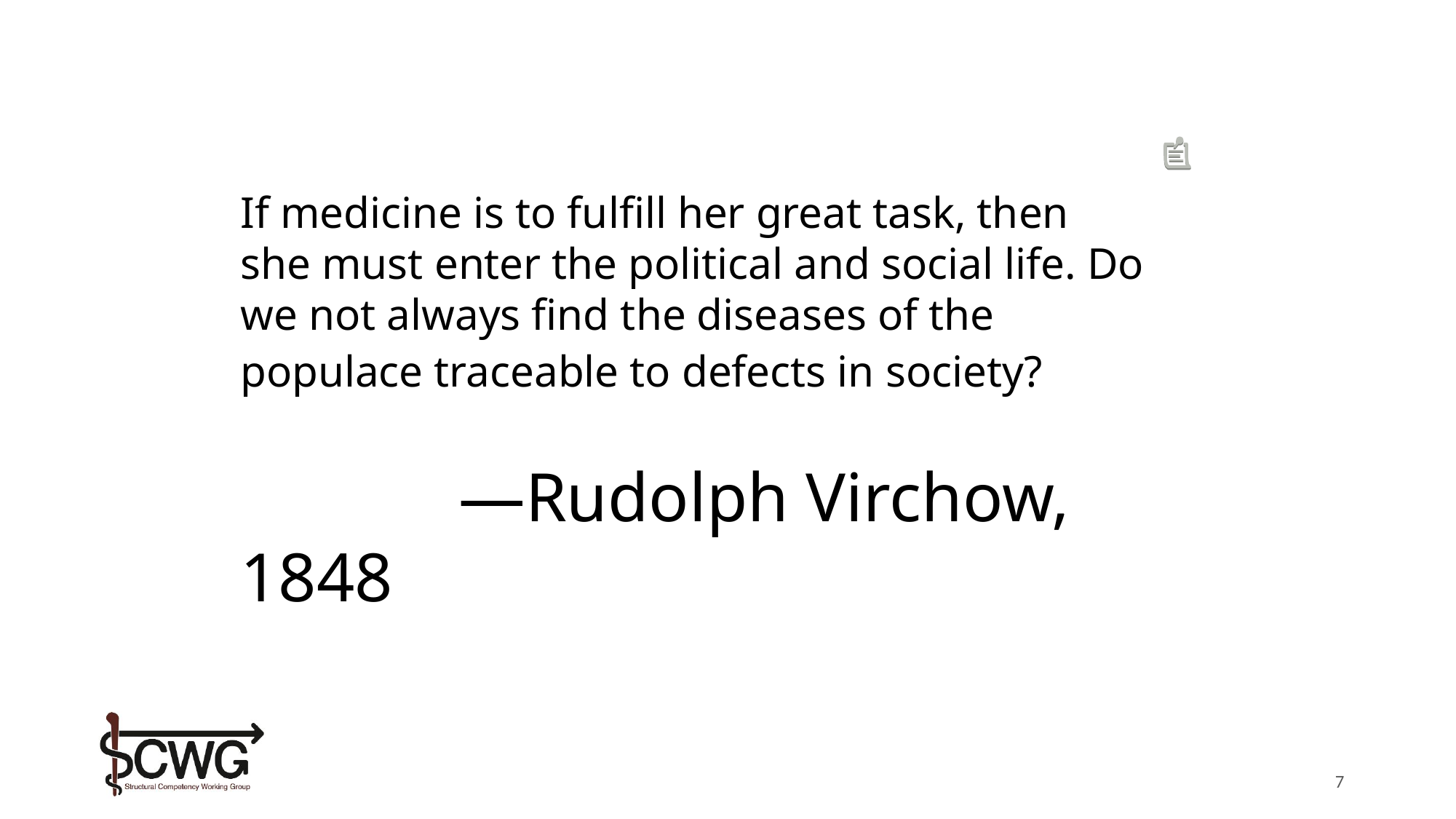

If medicine is to fulfill her great task, then she must enter the political and social life. Do we not always find the diseases of the populace traceable to defects in society?
		—Rudolph Virchow, 1848
7

## Slide 8
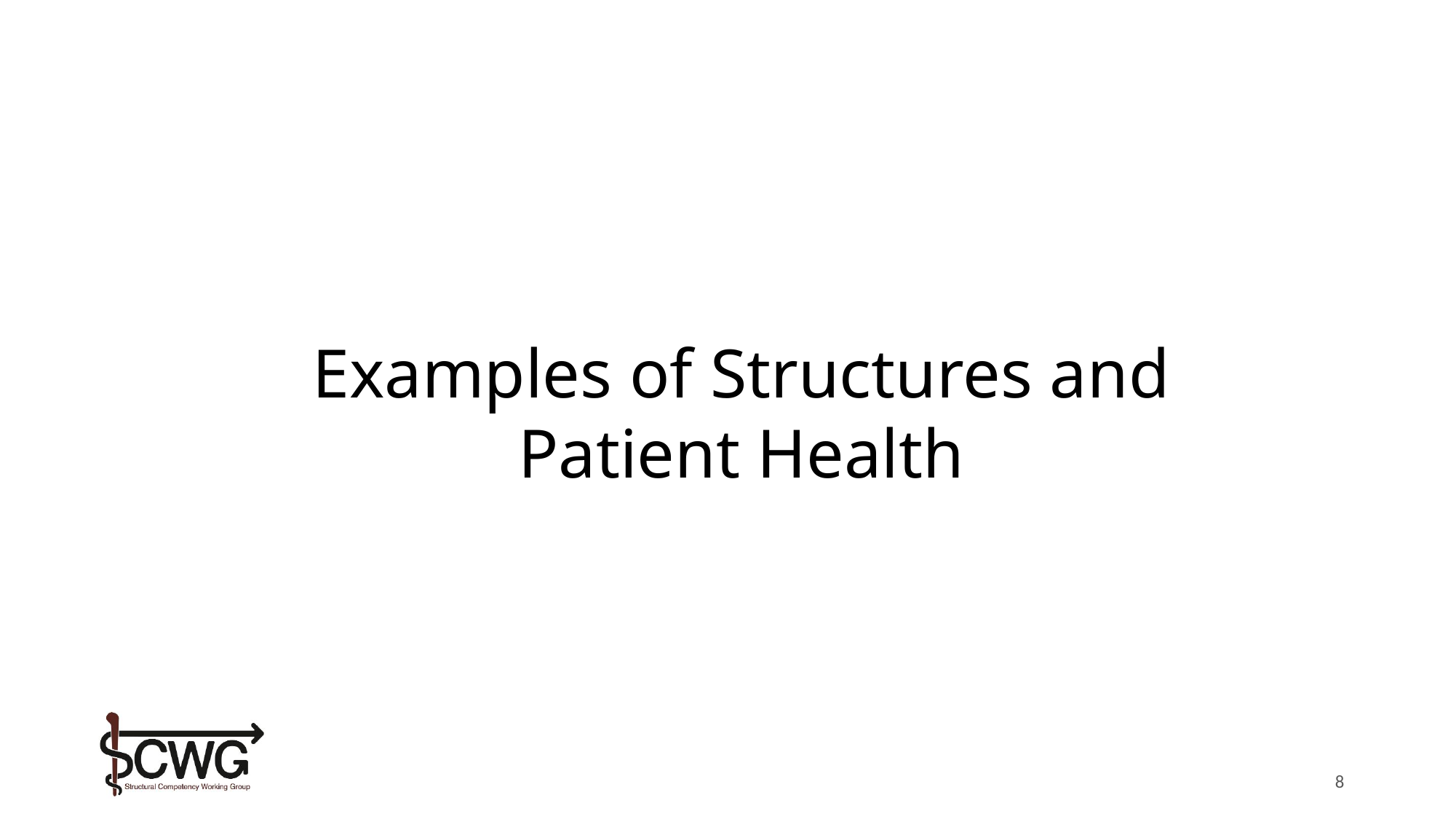

Examples of Structures and Patient Health
8

## Slide 9
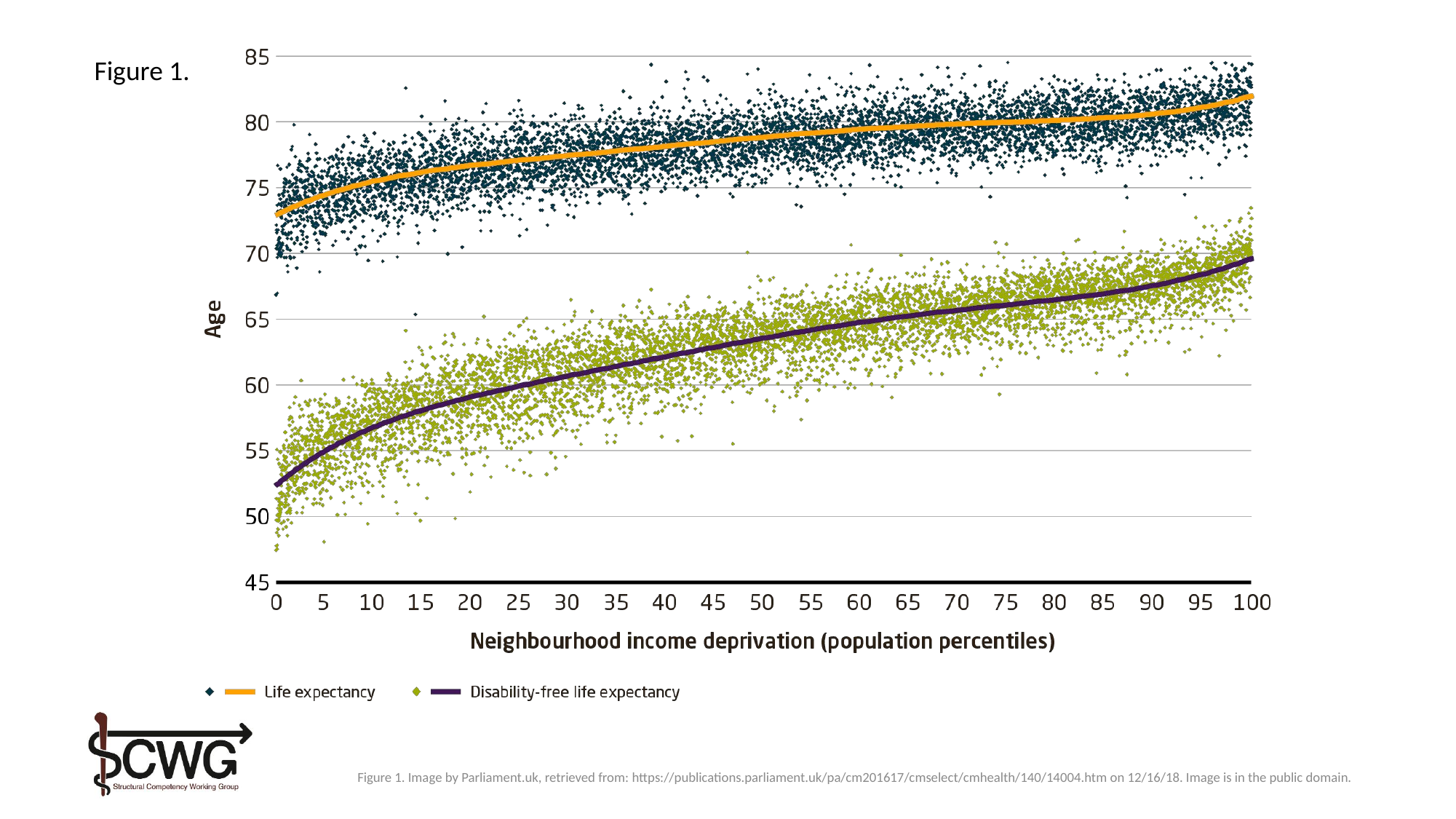

Figure 1.
Figure 1. Image by Parliament.uk, retrieved from: https://publications.parliament.uk/pa/cm201617/cmselect/cmhealth/140/14004.htm on 12/16/18. Image is in the public domain.

## Slide 10
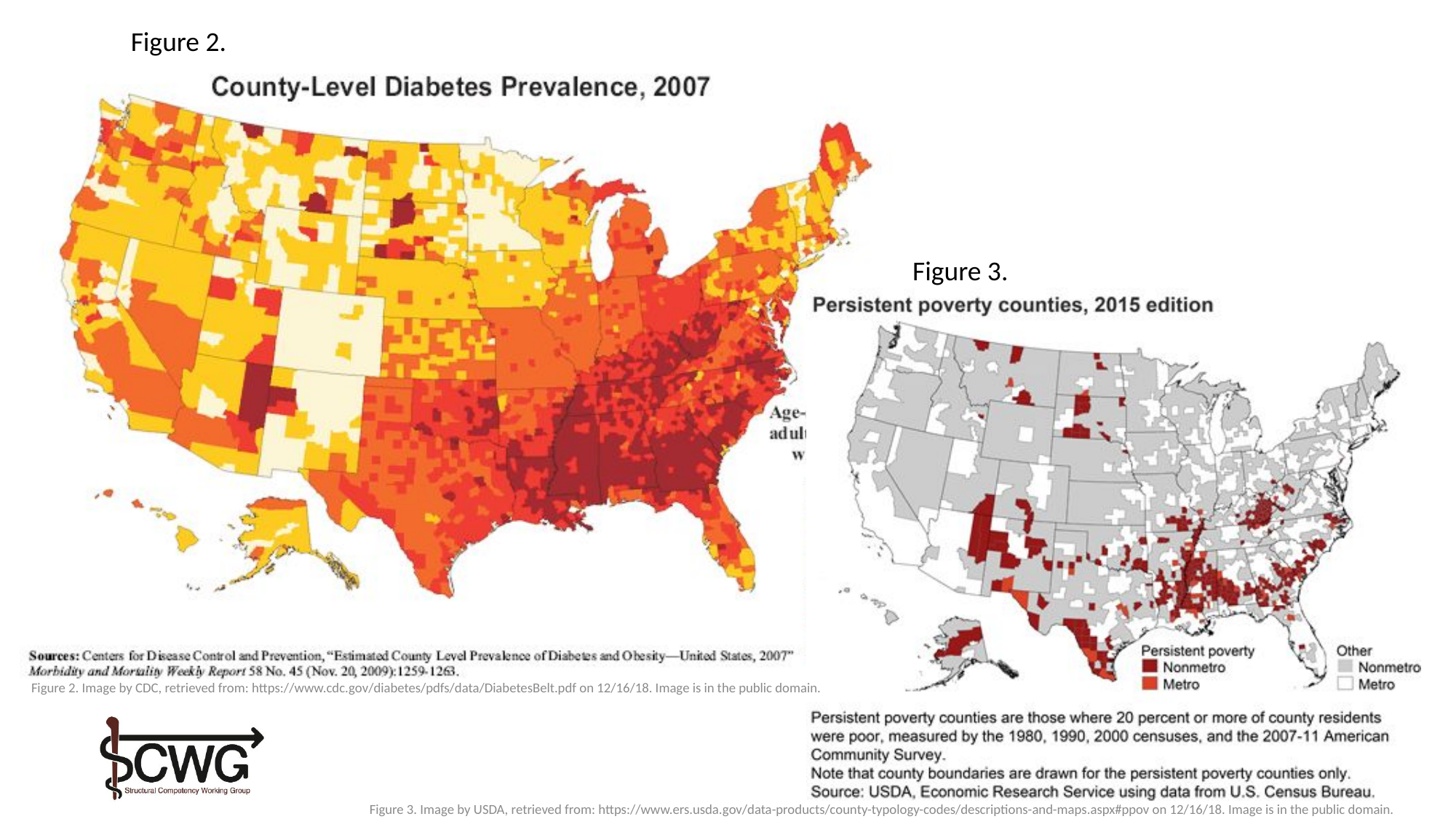

Figure 2.
Figure 3.
Figure 2. Image by CDC, retrieved from: https://www.cdc.gov/diabetes/pdfs/data/DiabetesBelt.pdf on 12/16/18. Image is in the public domain.
Figure 3. Image by USDA, retrieved from: https://www.ers.usda.gov/data-products/county-typology-codes/descriptions-and-maps.aspx#ppov on 12/16/18. Image is in the public domain.

## Slide 11
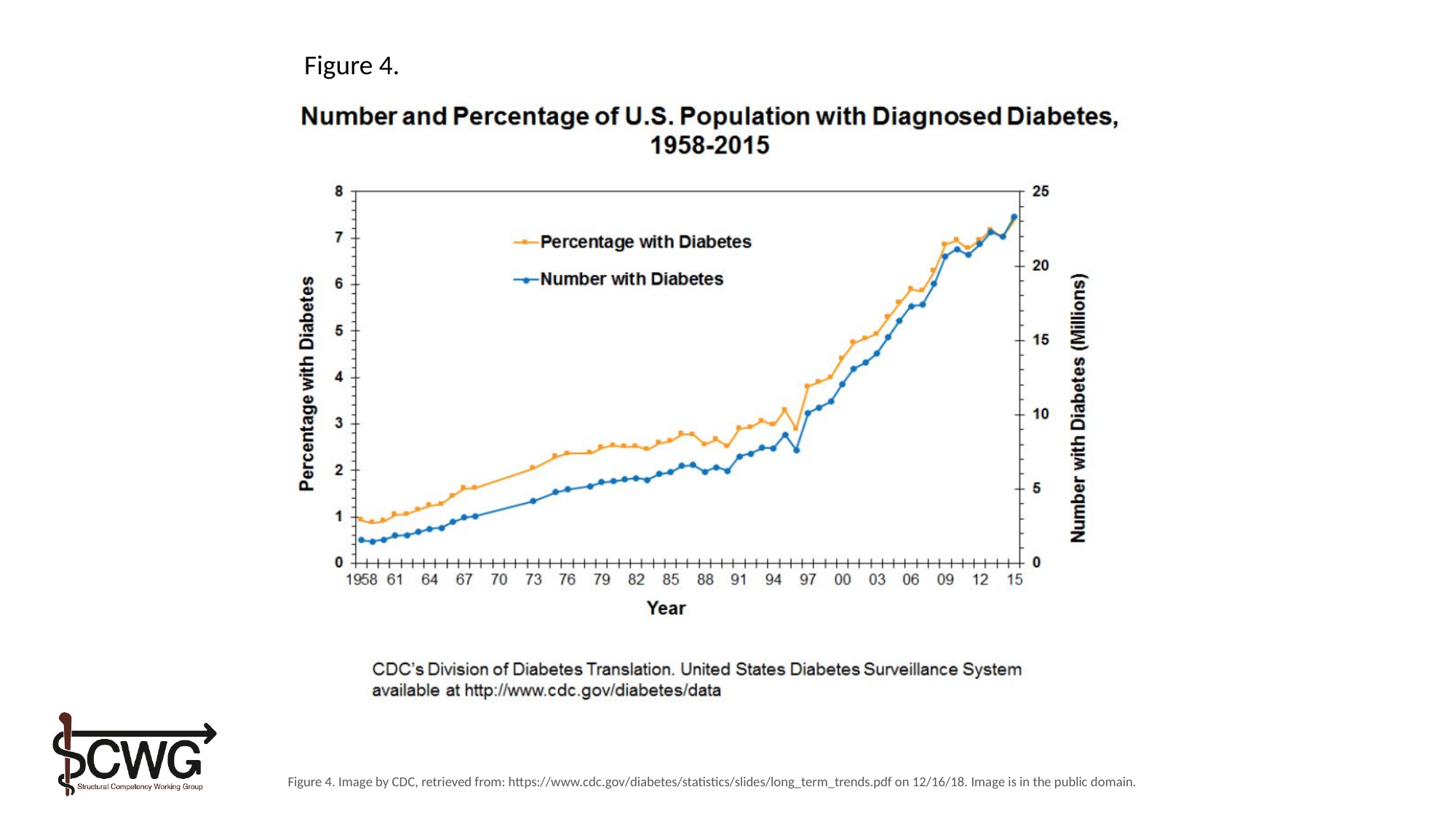

Figure 4.
Figure 4. Image by CDC, retrieved from: https://www.cdc.gov/diabetes/statistics/slides/long_term_trends.pdf on 12/16/18. Image is in the public domain.

## Slide 12
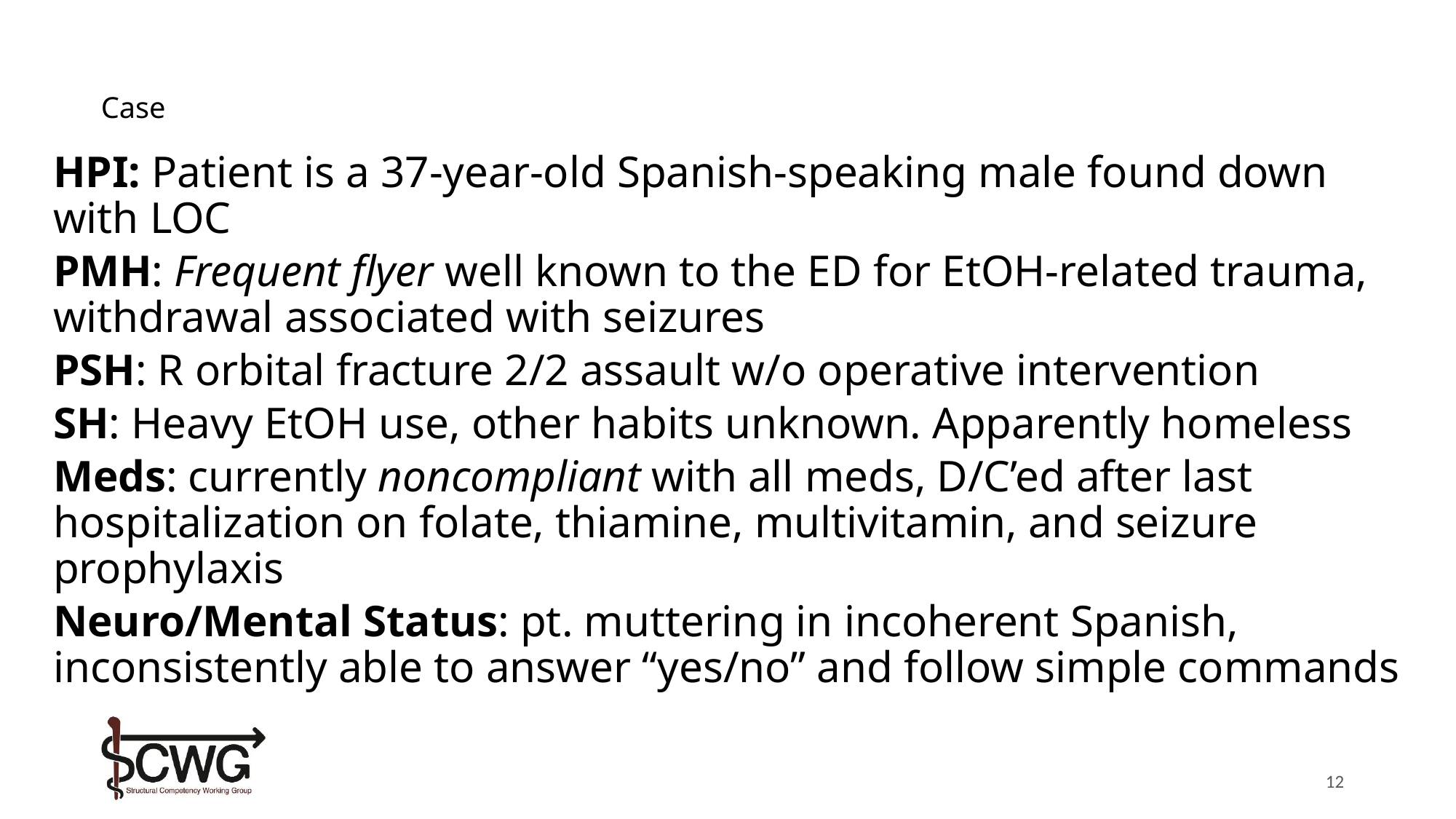

# Case
HPI: Patient is a 37-year-old Spanish-speaking male found down with LOC
PMH: Frequent flyer well known to the ED for EtOH-related trauma, withdrawal associated with seizures
PSH: R orbital fracture 2/2 assault w/o operative intervention
SH: Heavy EtOH use, other habits unknown. Apparently homeless
Meds: currently noncompliant with all meds, D/C’ed after last hospitalization on folate, thiamine, multivitamin, and seizure prophylaxis
Neuro/Mental Status: pt. muttering in incoherent Spanish, inconsistently able to answer “yes/no” and follow simple commands
12

## Slide 13
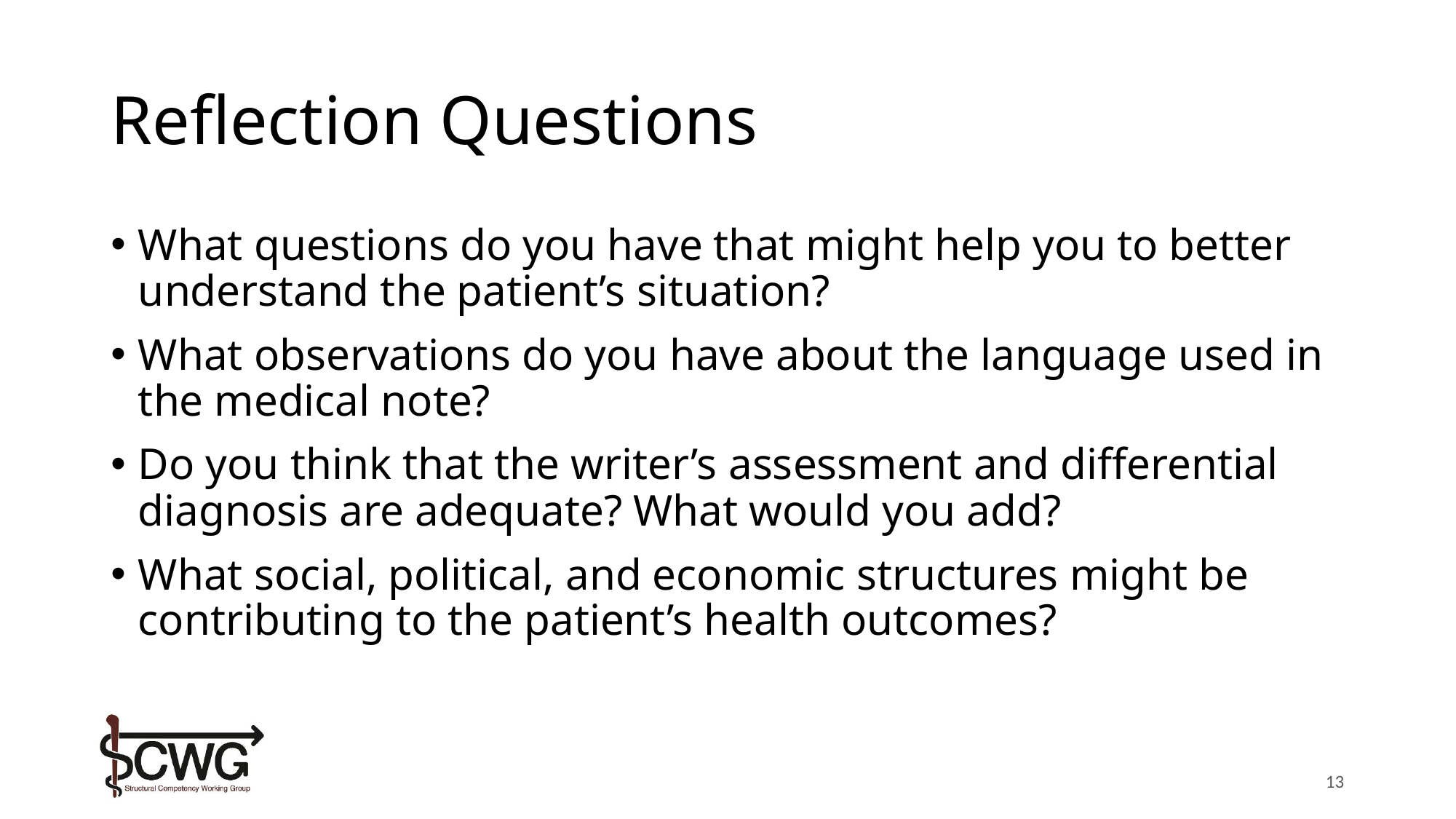

# Reflection Questions
What questions do you have that might help you to better understand the patient’s situation?
What observations do you have about the language used in the medical note?
Do you think that the writer’s assessment and differential diagnosis are adequate? What would you add?
What social, political, and economic structures might be contributing to the patient’s health outcomes?
13

## Slide 14
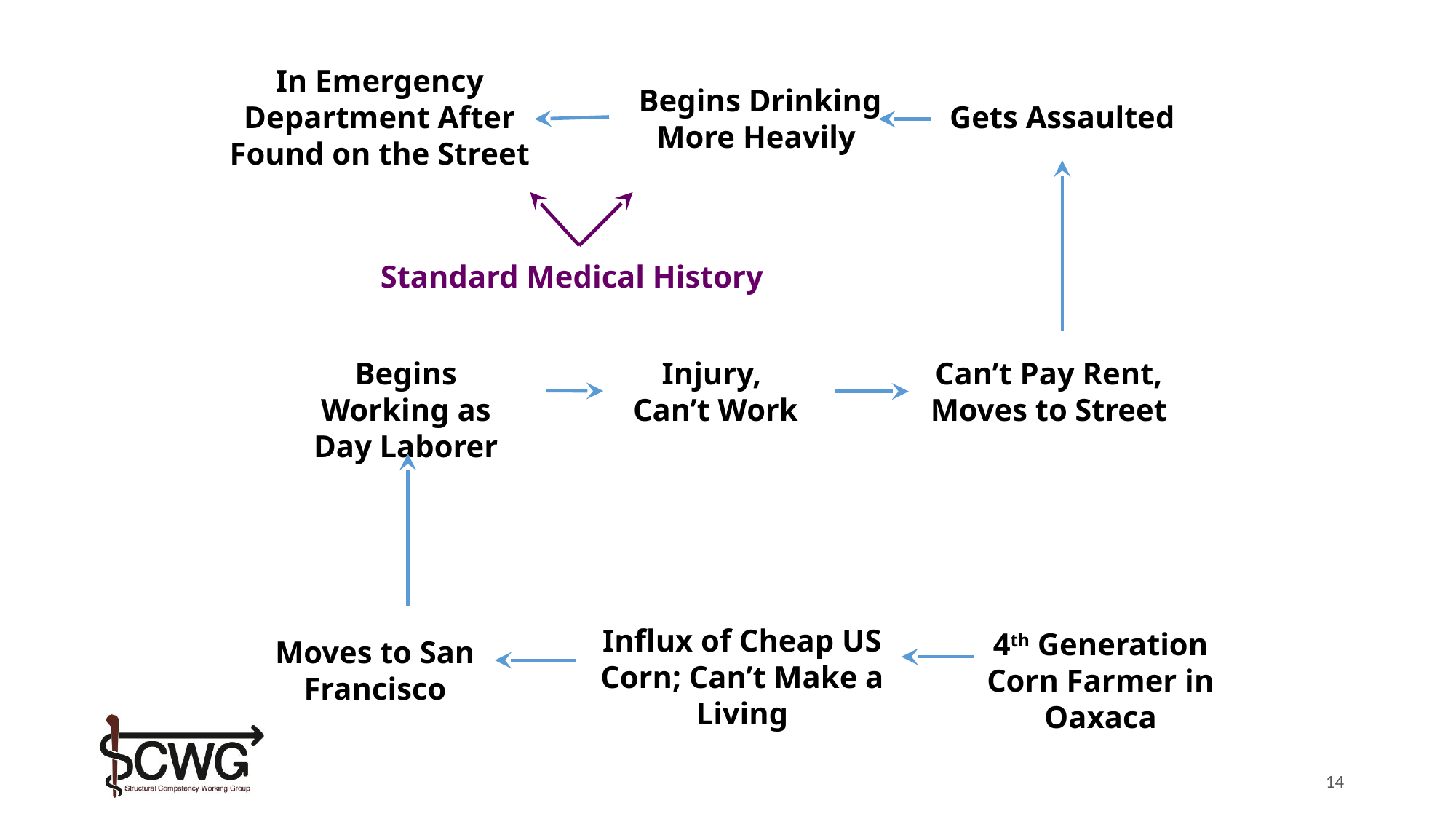

In Emergency Department After Found on the Street
Begins Drinking More Heavily
Gets Assaulted
Standard Medical History
Injury,
 Can’t Work
Can’t Pay Rent, Moves to Street
Begins Working as Day Laborer
Influx of Cheap US Corn; Can’t Make a Living
4th Generation Corn Farmer in Oaxaca
Moves to San Francisco
14

## Slide 15
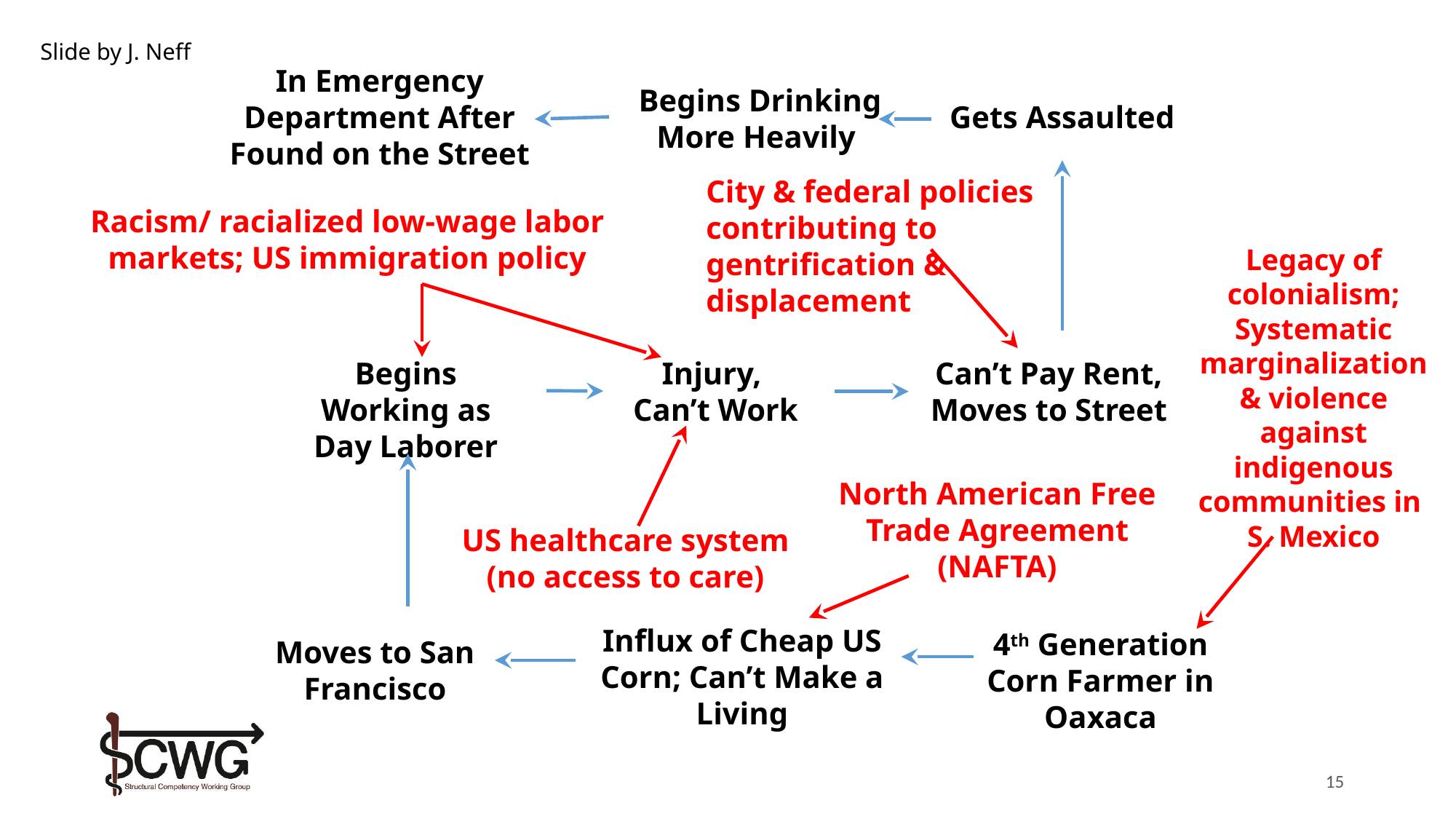

Slide by J. Neff
In Emergency Department After Found on the Street
Begins Drinking More Heavily
Gets Assaulted
City & federal policies contributing to gentrification & displacement
Racism/ racialized low-wage labor markets; US immigration policy
Legacy of colonialism; Systematic marginalization & violence against indigenous communities in
S. Mexico
Injury,
 Can’t Work
Can’t Pay Rent, Moves to Street
Begins Working as Day Laborer
US healthcare system (no access to care)
North American Free Trade Agreement (NAFTA)
Influx of Cheap US Corn; Can’t Make a Living
4th Generation Corn Farmer in Oaxaca
Moves to San Francisco
15

## Slide 16
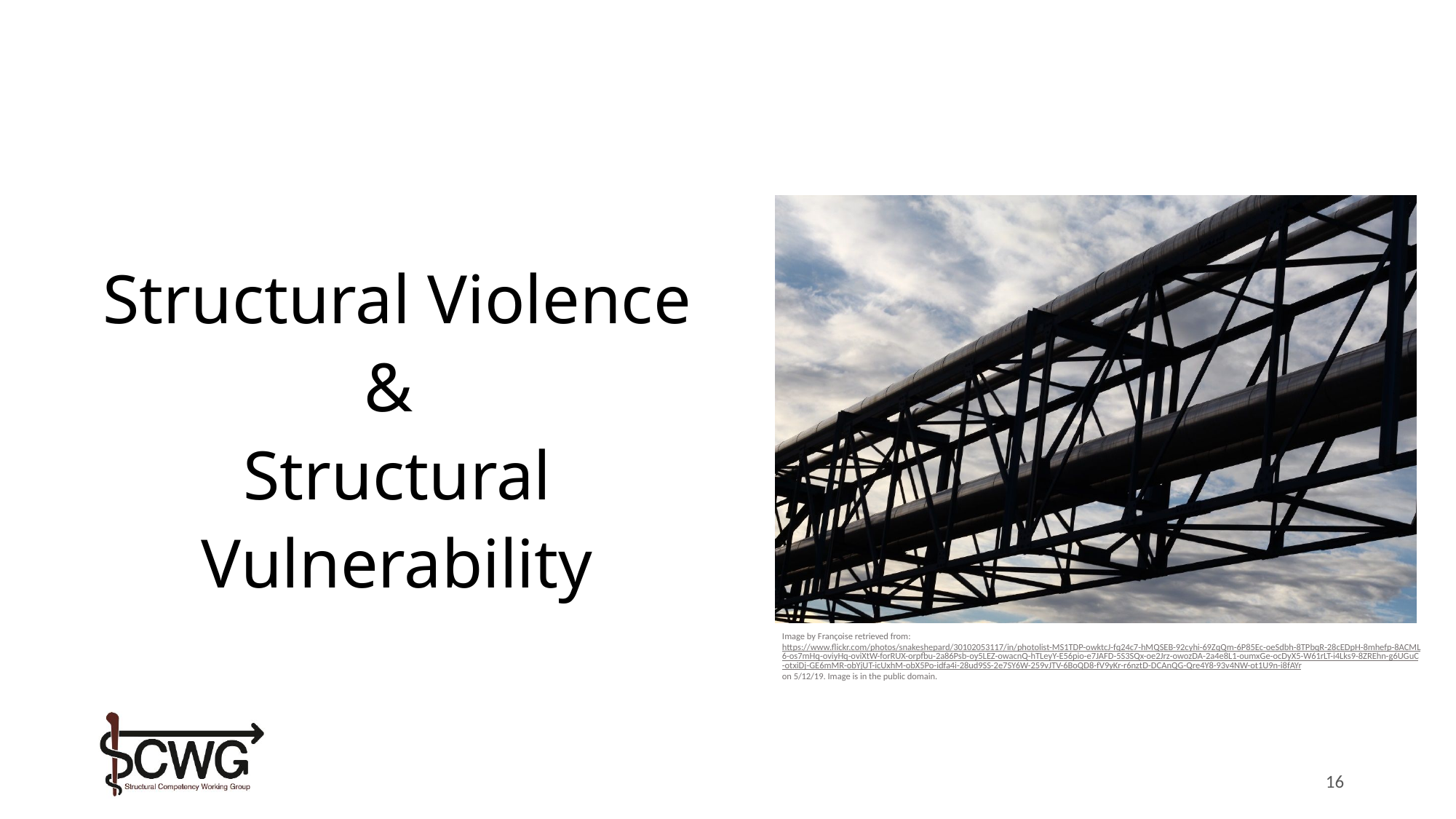

Structural Violence
&
Structural Vulnerability
Image by Françoise retrieved from: https://www.flickr.com/photos/snakeshepard/30102053117/in/photolist-MS1TDP-owktcJ-fq24c7-hMQSEB-92cyhi-69ZqQm-6P85Ec-oeSdbh-8TPbqR-28cEDpH-8mhefp-8ACML6-os7mHq-oviyHq-oviXtW-forRUX-orpfbu-2a86Psb-oy5LEZ-owacnQ-hTLeyY-E56pio-e7JAFD-5S3SQx-oe2Jrz-owozDA-2a4e8L1-oumxGe-ocDyX5-W61rLT-i4Lks9-8ZREhn-g6UGuC-otxiDj-GE6mMR-obYjUT-icUxhM-obX5Po-idfa4i-28ud9SS-2e7SY6W-259vJTV-6BoQD8-fV9yKr-r6nztD-DCAnQG-Qre4Y8-93v4NW-ot1U9n-i8fAYron 5/12/19. Image is in the public domain.
16

## Slide 17
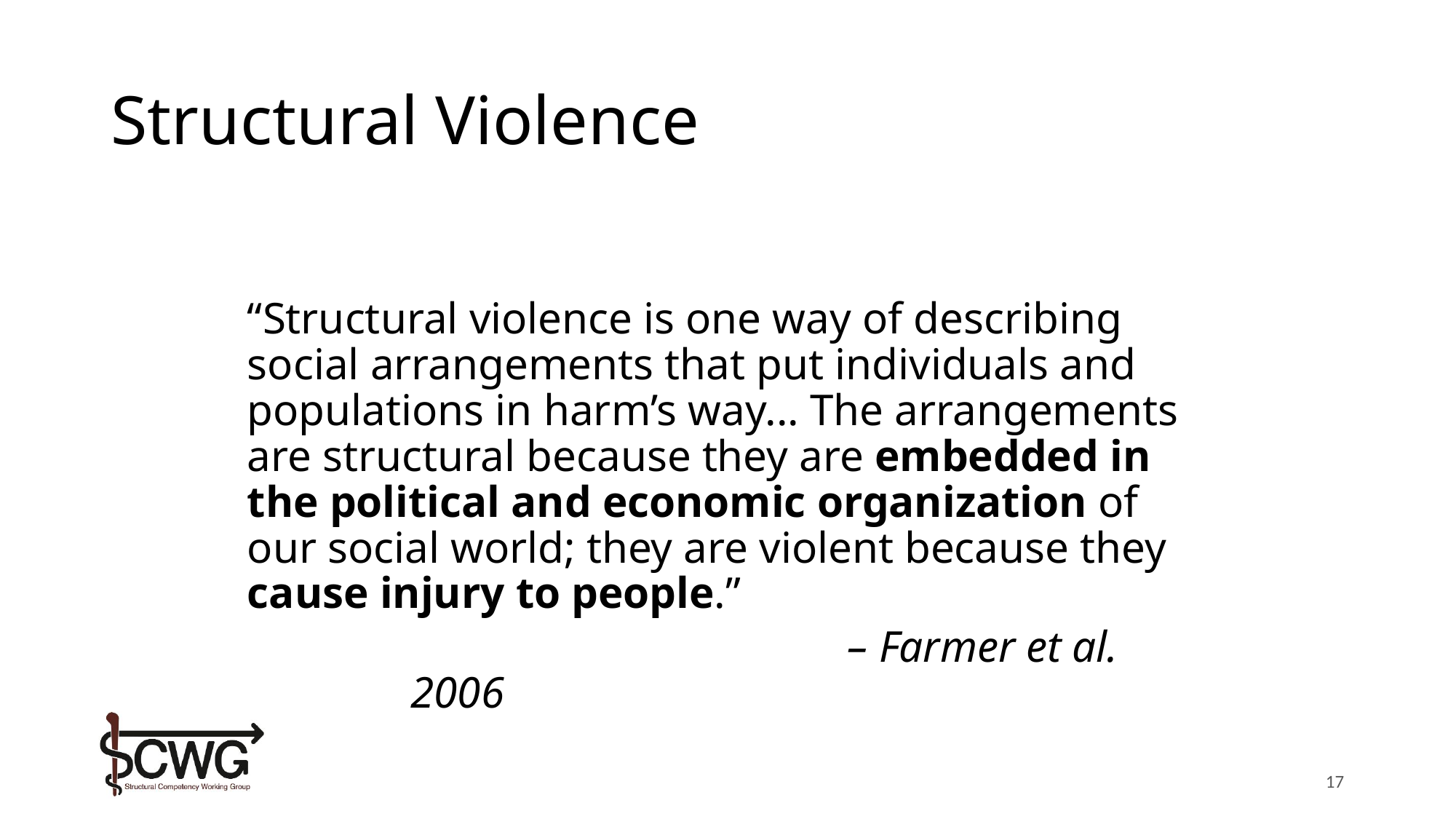

# Structural Violence
“Structural violence is one way of describing social arrangements that put individuals and populations in harm’s way... The arrangements are structural because they are embedded in the political and economic organization of our social world; they are violent because they cause injury to people.”
				– Farmer et al. 2006
17

## Slide 18
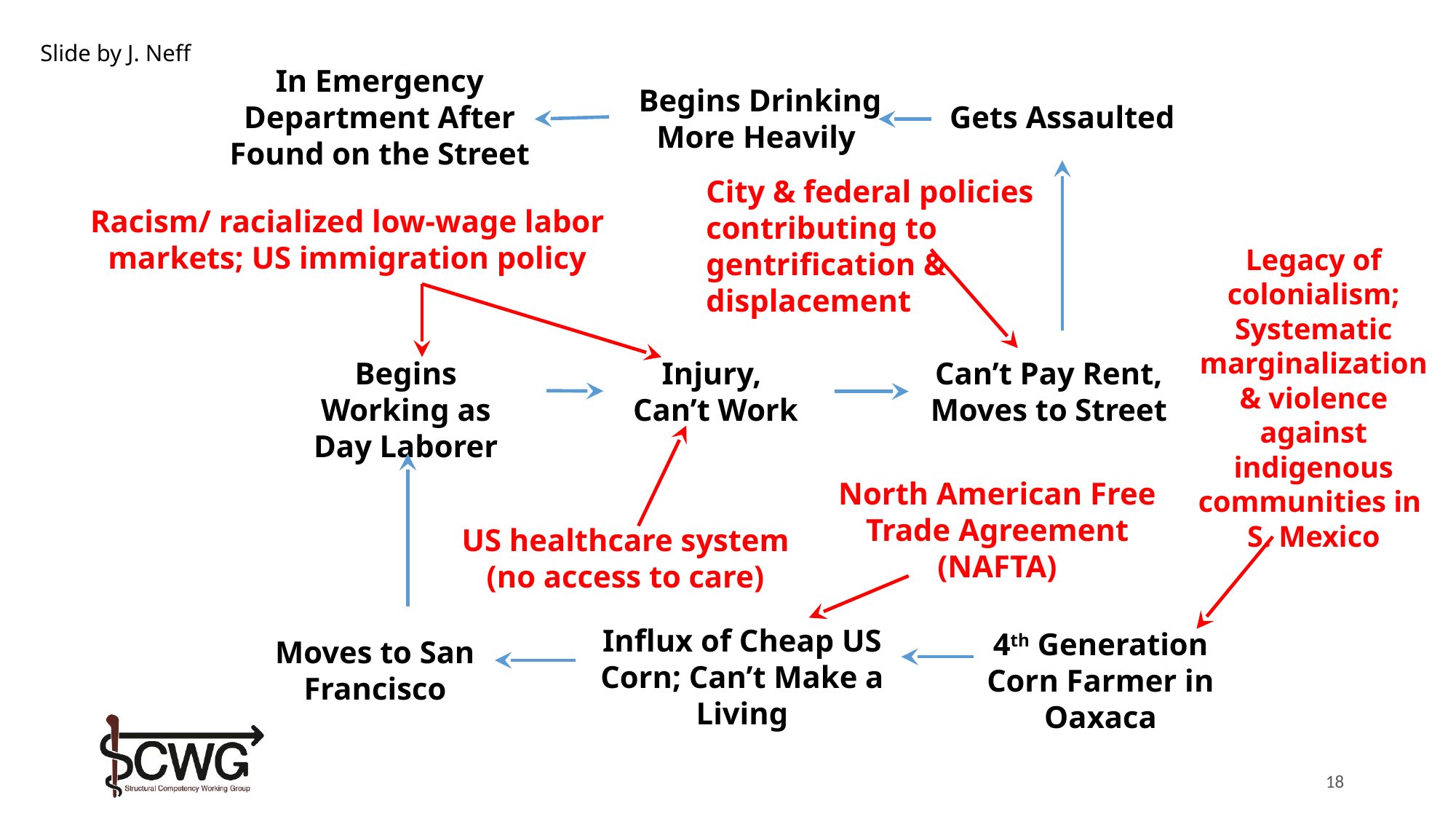

Slide by J. Neff
In Emergency Department After Found on the Street
Begins Drinking More Heavily
Gets Assaulted
City & federal policies contributing to gentrification & displacement
Racism/ racialized low-wage labor markets; US immigration policy
Legacy of colonialism; Systematic marginalization & violence against indigenous communities in
S. Mexico
Injury,
 Can’t Work
Can’t Pay Rent, Moves to Street
Begins Working as Day Laborer
US healthcare system (no access to care)
North American Free Trade Agreement (NAFTA)
Influx of Cheap US Corn; Can’t Make a Living
4th Generation Corn Farmer in Oaxaca
Moves to San Francisco
18

## Slide 19
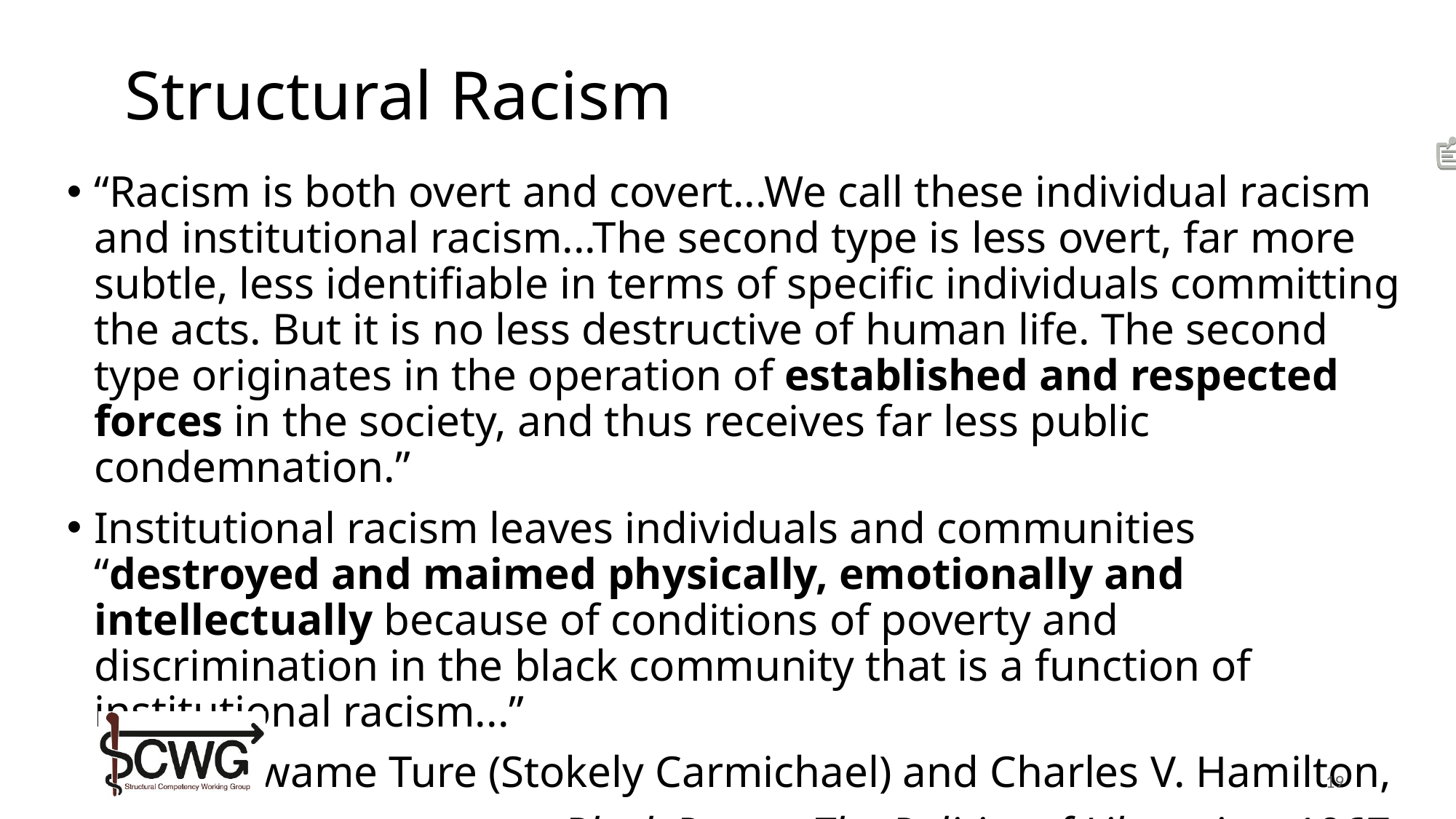

# Structural Racism
“Racism is both overt and covert...We call these individual racism and institutional racism...The second type is less overt, far more subtle, less identifiable in terms of specific individuals committing the acts. But it is no less destructive of human life. The second type originates in the operation of established and respected forces in the society, and thus receives far less public condemnation.”
Institutional racism leaves individuals and communities “destroyed and maimed physically, emotionally and intellectually because of conditions of poverty and discrimination in the black community that is a function of institutional racism...”
Kwame Ture (Stokely Carmichael) and Charles V. Hamilton,
Black Power: The Politics of Liberation. 1967.
19

## Slide 20
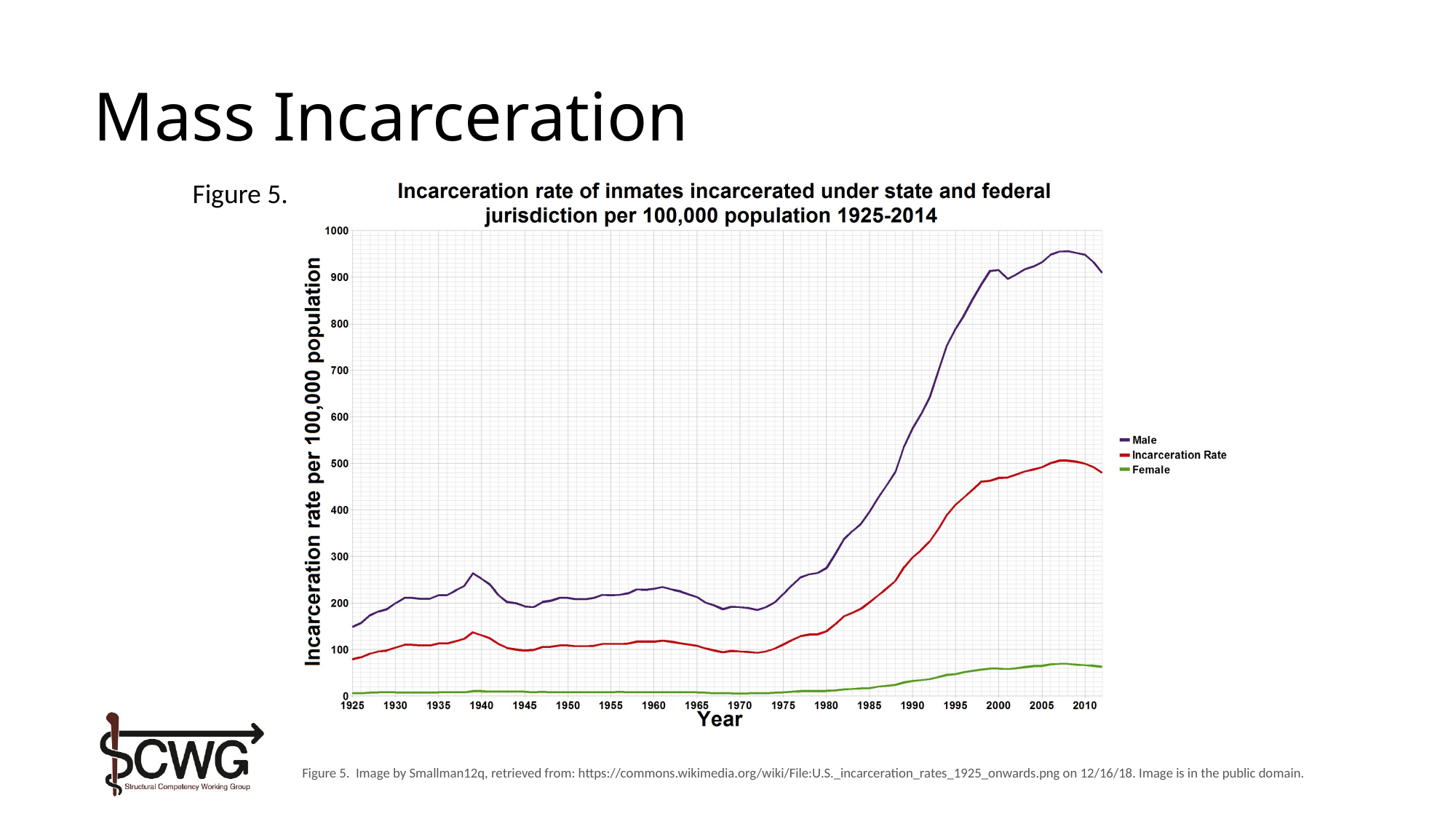

# Mass Incarceration
Figure 5.
Figure 5. Image by Smallman12q, retrieved from: https://commons.wikimedia.org/wiki/File:U.S._incarceration_rates_1925_onwards.png on 12/16/18. Image is in the public domain.

## Slide 21
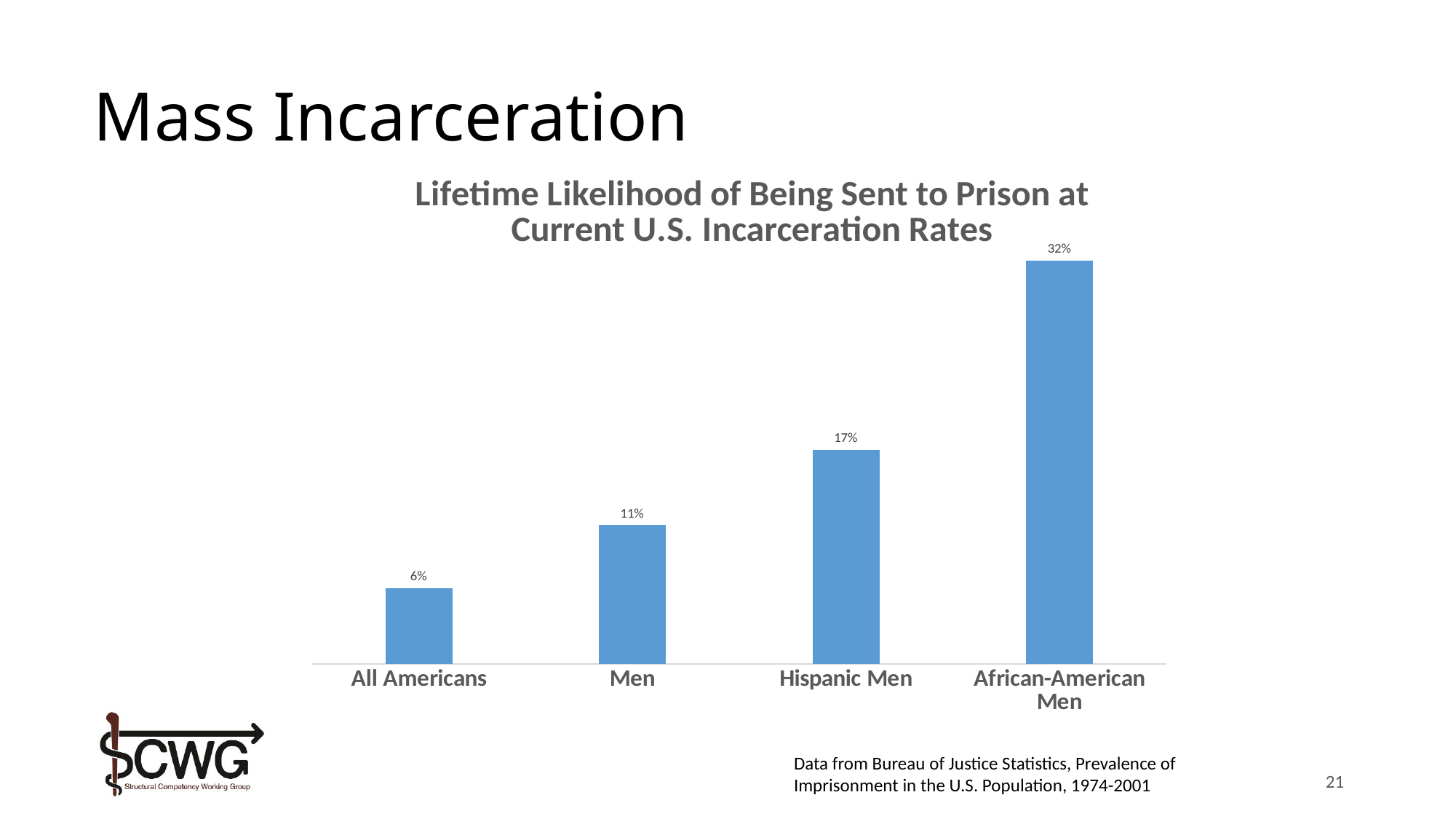

Mass Incarceration
### Chart: Lifetime Likelihood of Being Sent to Prison at Current U.S. Incarceration Rates
| Category | |
|---|---|
| All Americans | 0.06 |
| Men | 0.11 |
| Hispanic Men | 0.17 |
| African-American Men | 0.32 |
Data from Bureau of Justice Statistics, Prevalence of Imprisonment in the U.S. Population, 1974-2001
21

## Slide 22
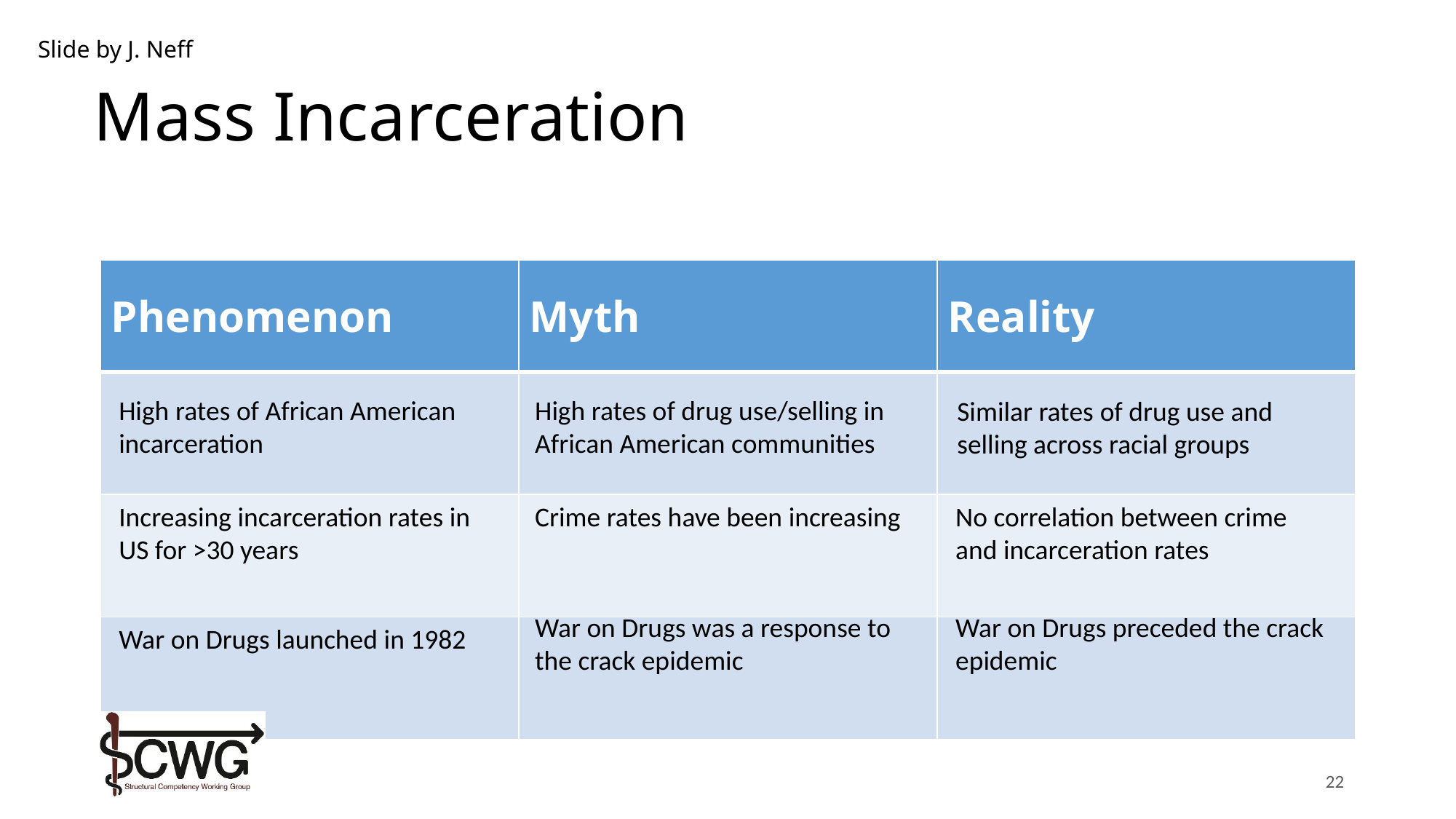

Slide by J. Neff
Mass Incarceration
| Phenomenon | Myth | Reality |
| --- | --- | --- |
| | | |
| | | |
| | | |
High rates of African American incarceration
High rates of drug use/selling in African American communities
Similar rates of drug use and selling across racial groups
Increasing incarceration rates in US for >30 years
Crime rates have been increasing
No correlation between crime and incarceration rates
War on Drugs was a response to the crack epidemic
War on Drugs preceded the crack epidemic
War on Drugs launched in 1982
22

## Slide 23
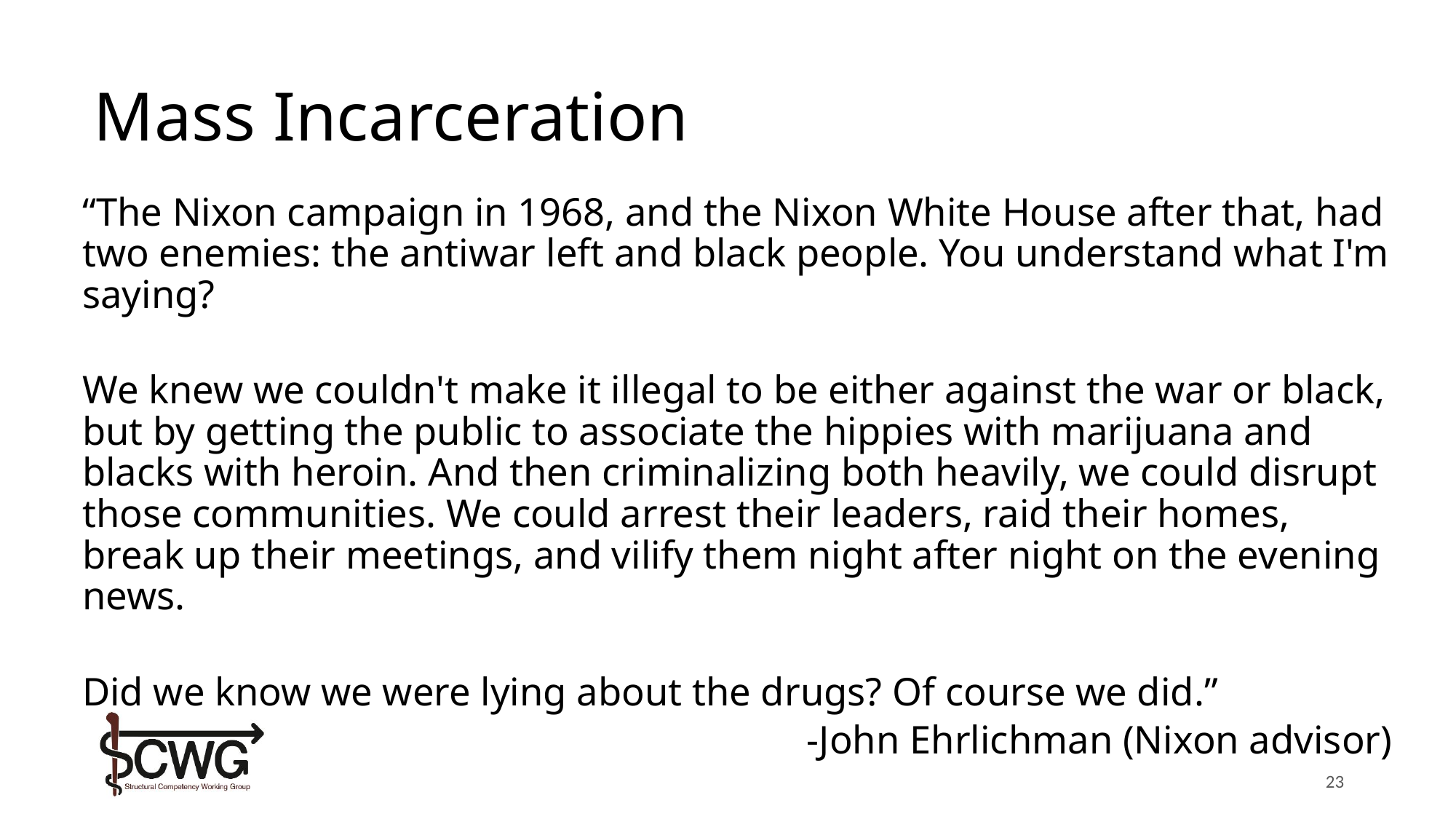

Mass Incarceration
“The Nixon campaign in 1968, and the Nixon White House after that, had two enemies: the antiwar left and black people. You understand what I'm saying?
We knew we couldn't make it illegal to be either against the war or black, but by getting the public to associate the hippies with marijuana and blacks with heroin. And then criminalizing both heavily, we could disrupt those communities. We could arrest their leaders, raid their homes, break up their meetings, and vilify them night after night on the evening news.
Did we know we were lying about the drugs? Of course we did.”
-John Ehrlichman (Nixon advisor)
23

## Slide 24
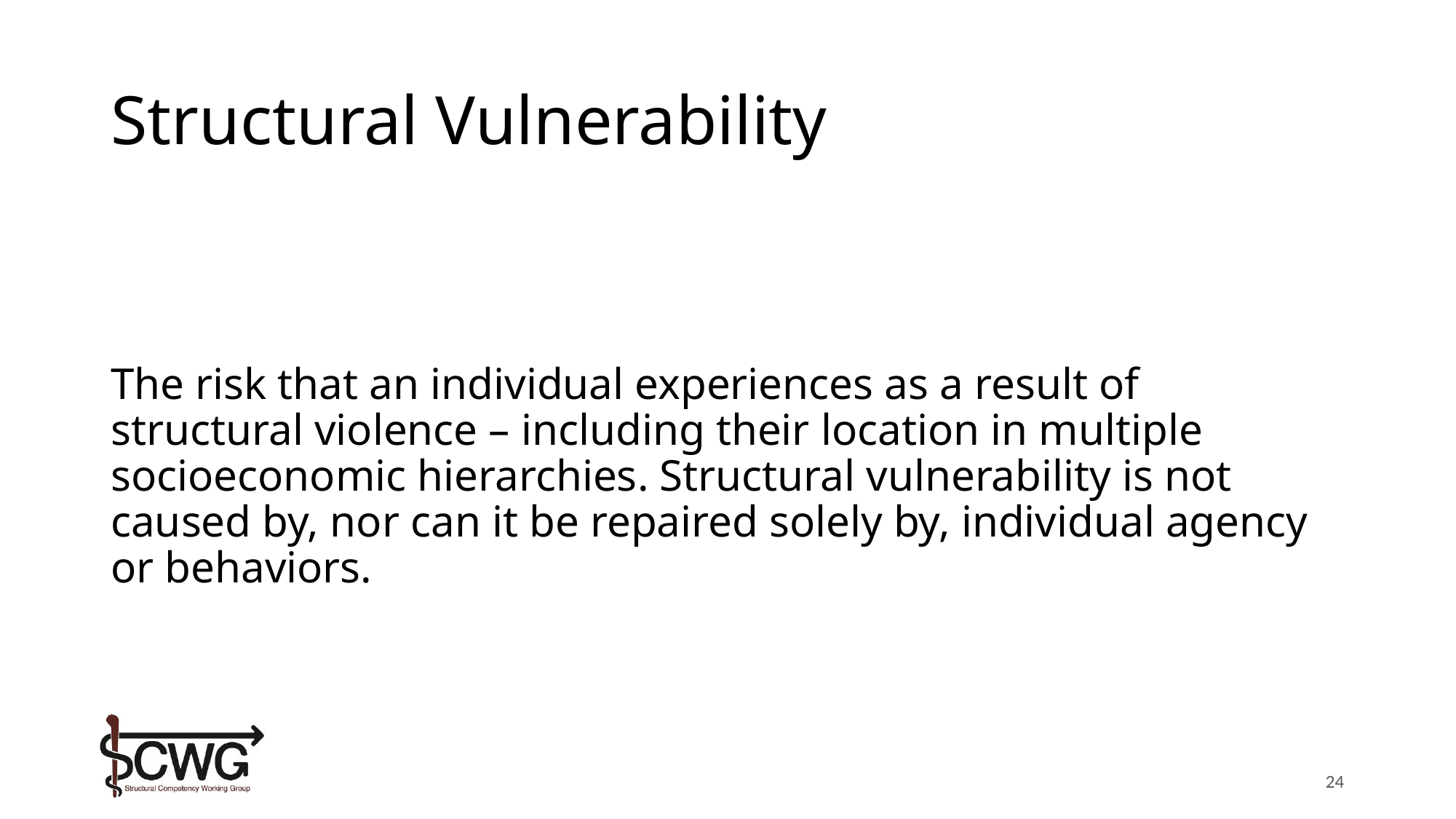

# Structural Vulnerability
The risk that an individual experiences as a result of structural violence – including their location in multiple socioeconomic hierarchies. Structural vulnerability is not caused by, nor can it be repaired solely by, individual agency or behaviors.
24

## Slide 25
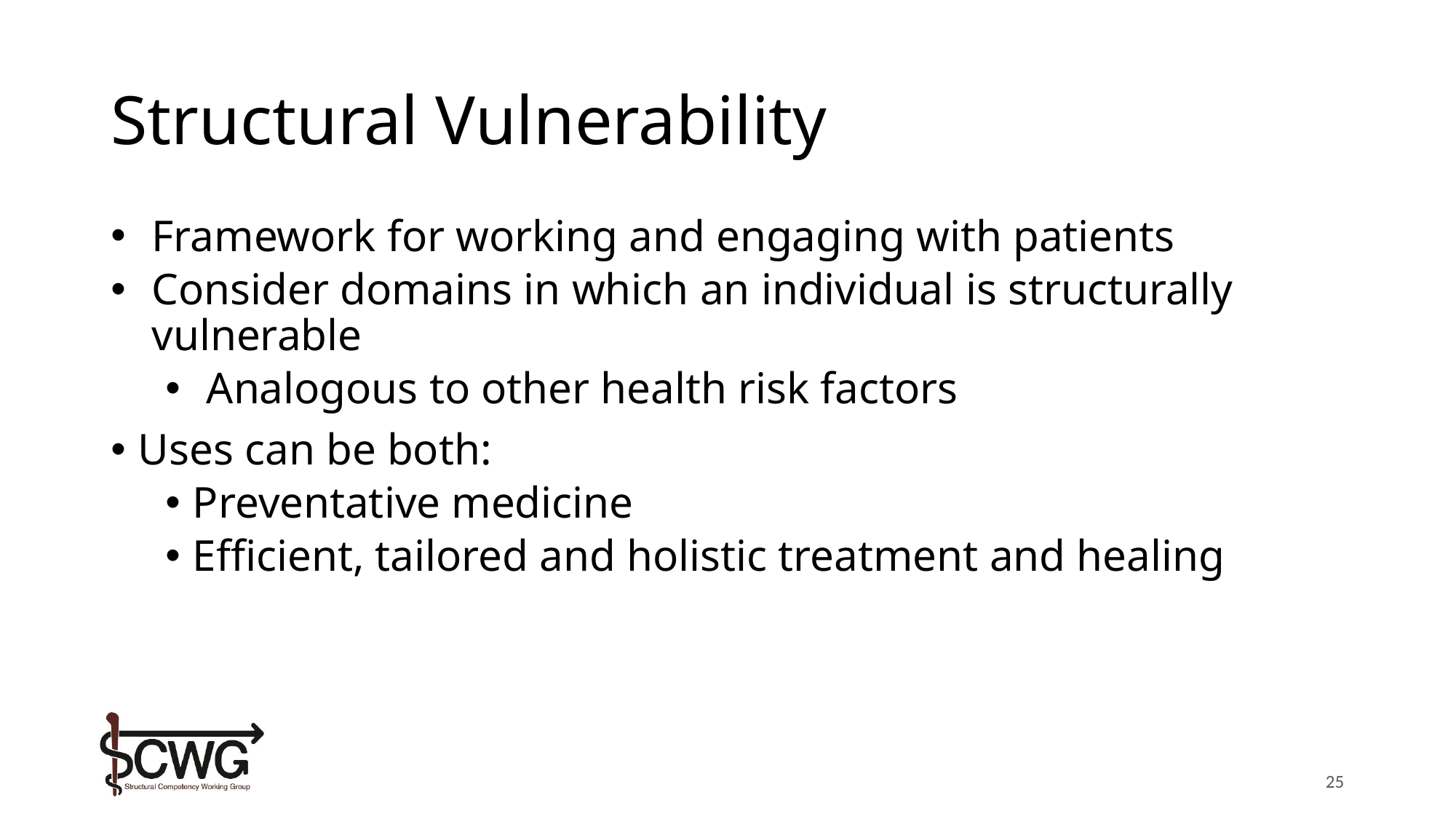

# Structural Vulnerability
Framework for working and engaging with patients
Consider domains in which an individual is structurally vulnerable
Analogous to other health risk factors
Uses can be both:
Preventative medicine
Efficient, tailored and holistic treatment and healing
25

## Slide 26
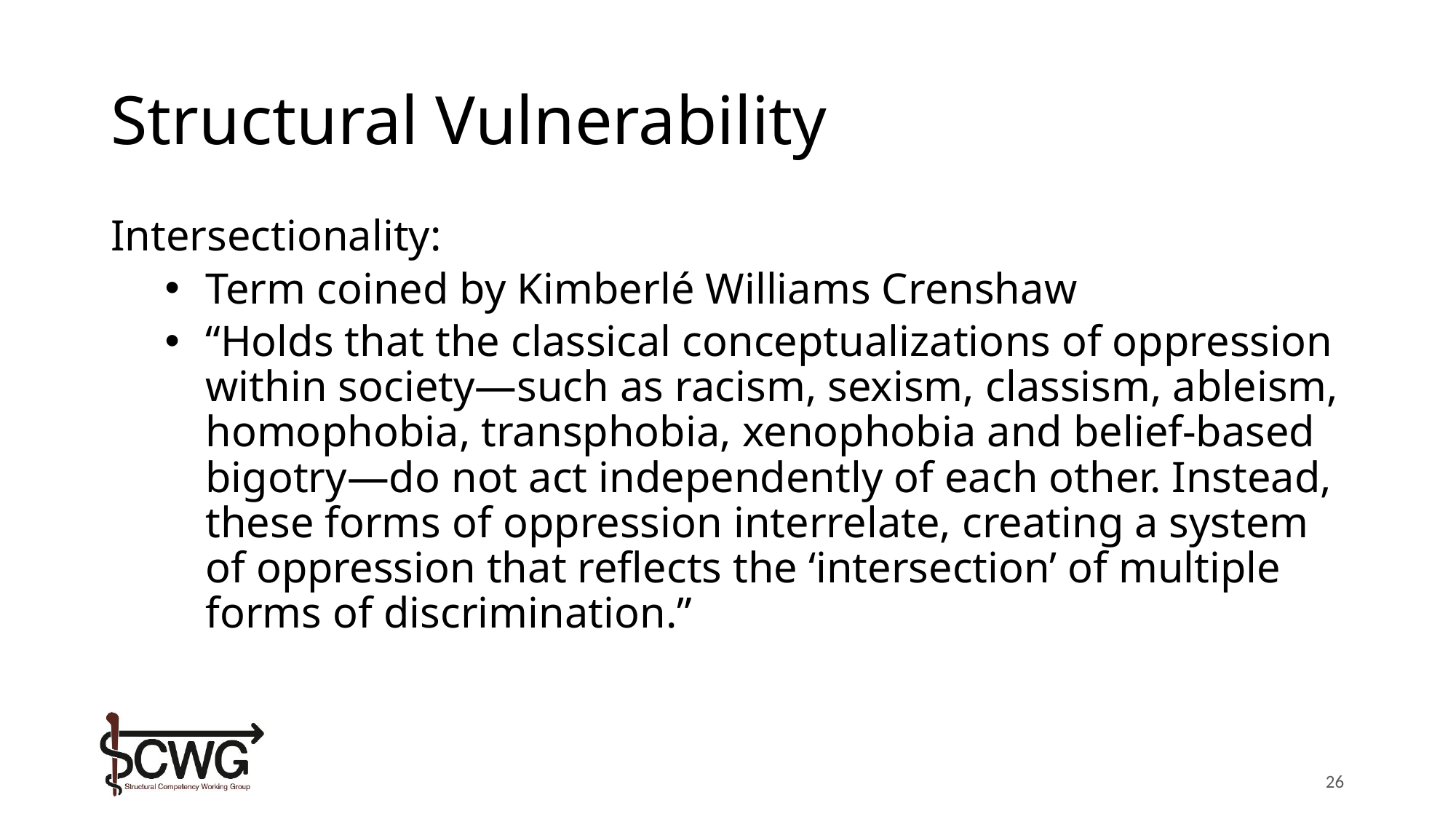

# Structural Vulnerability
Intersectionality:
Term coined by Kimberlé Williams Crenshaw
“Holds that the classical conceptualizations of oppression within society—such as racism, sexism, classism, ableism, homophobia, transphobia, xenophobia and belief-based bigotry—do not act independently of each other. Instead, these forms of oppression interrelate, creating a system of oppression that reflects the ‘intersection’ of multiple forms of discrimination.”
26

## Slide 27
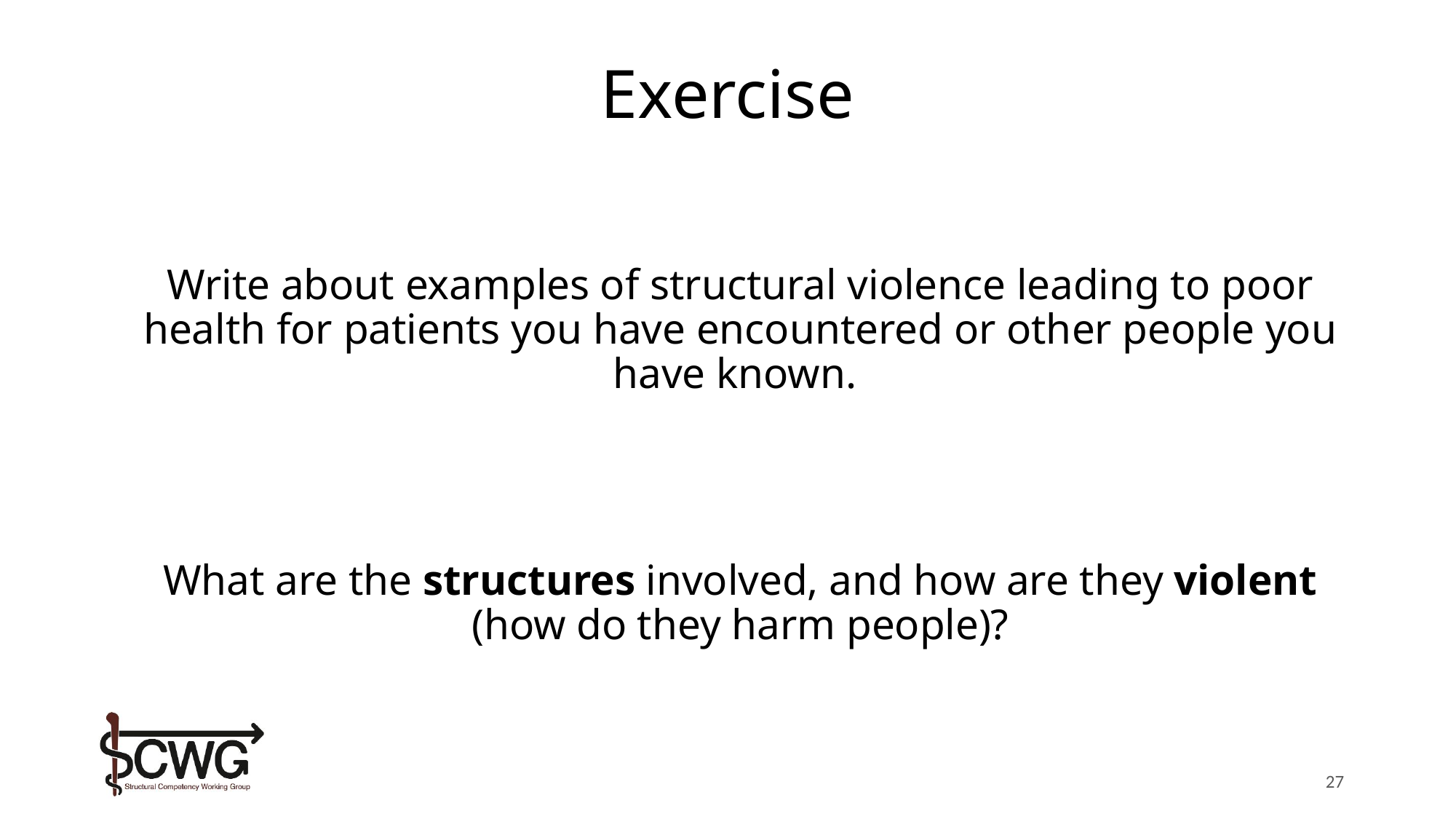

# Exercise
Write about examples of structural violence leading to poor health for patients you have encountered or other people you have known.
What are the structures involved, and how are they violent (how do they harm people)?
27

## Slide 28
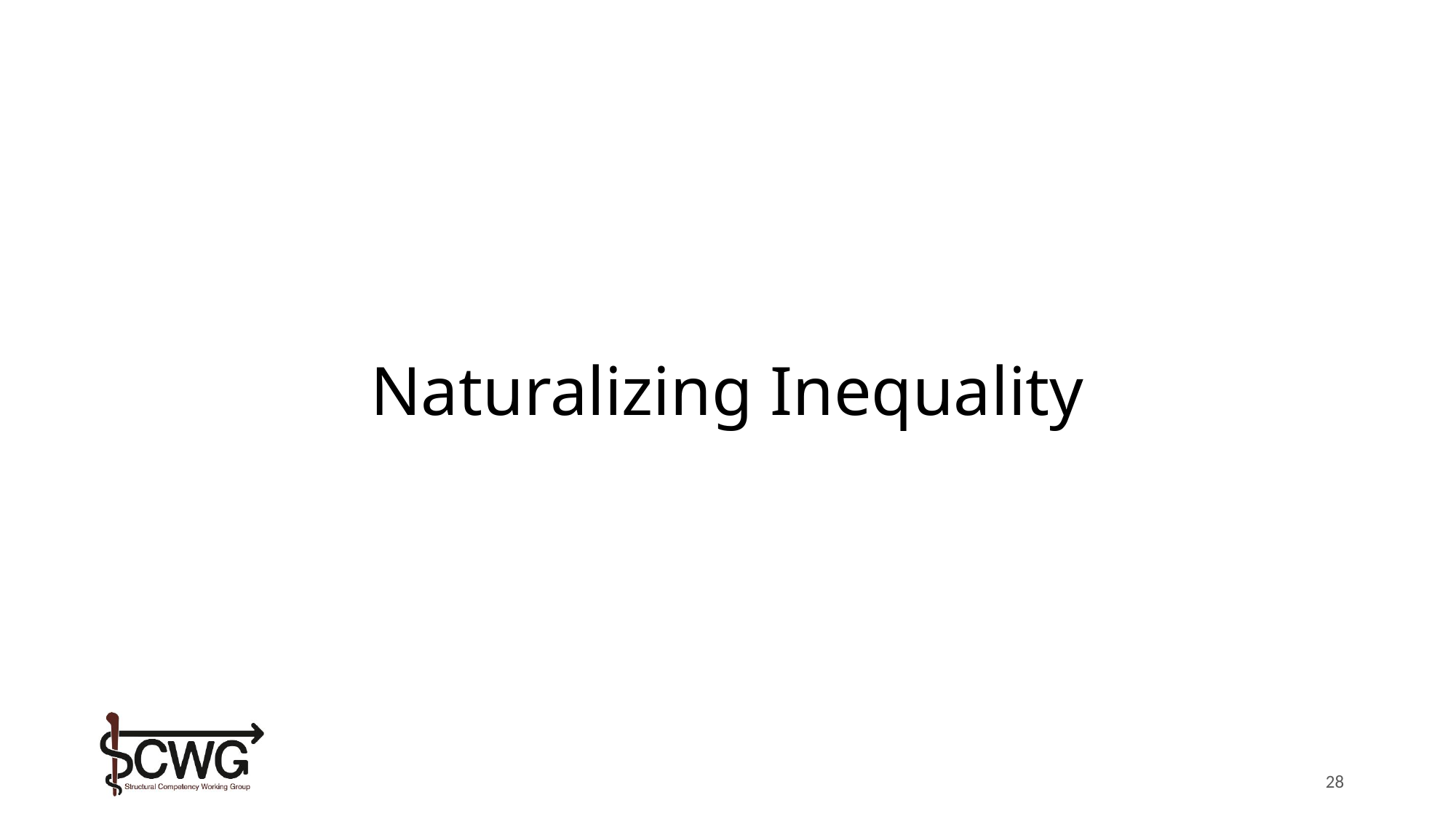

Naturalizing Inequality
28

## Slide 29
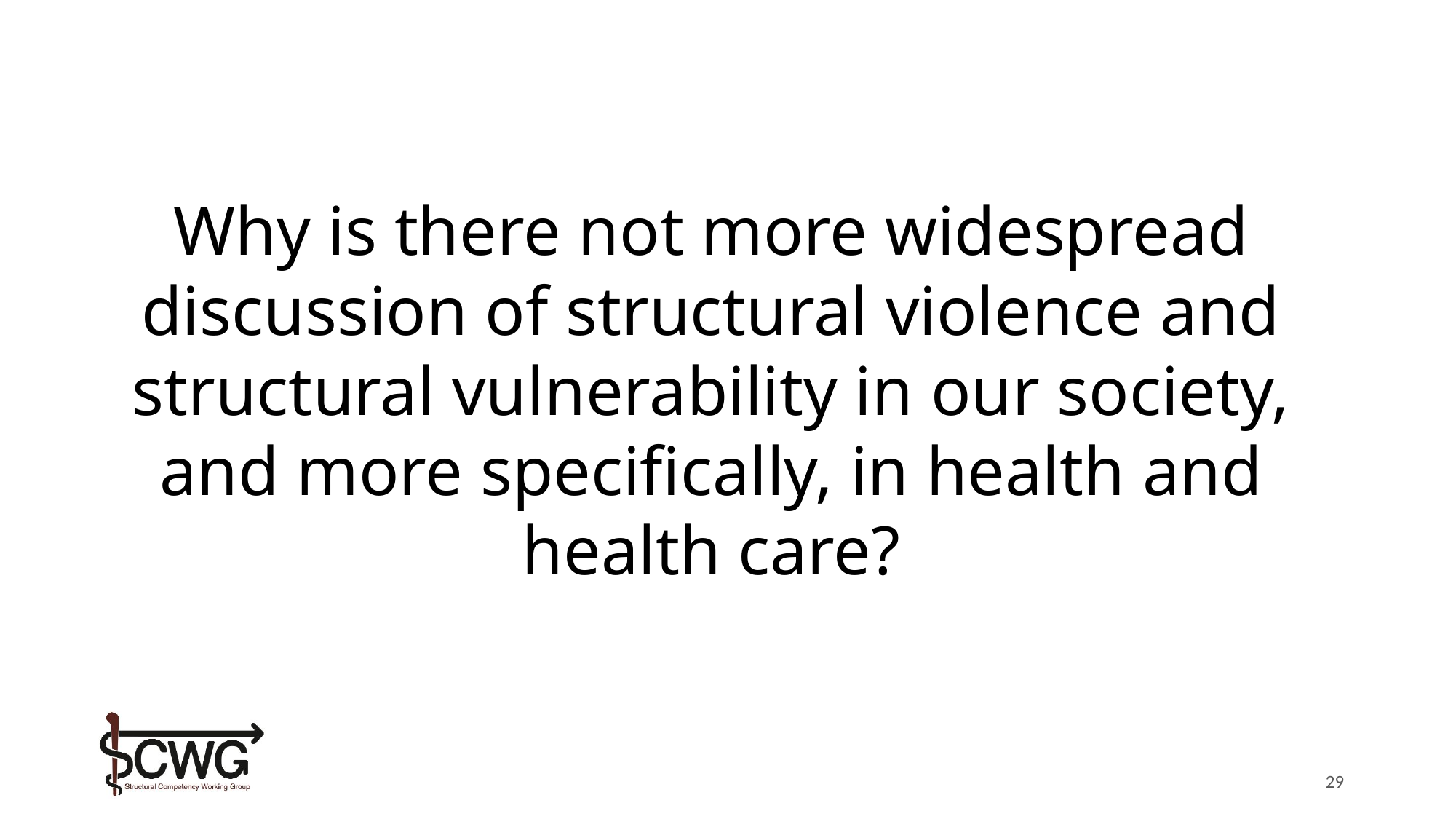

Why is there not more widespread
discussion of structural violence and structural vulnerability in our society, and more specifically, in health and health care?
29

## Slide 30
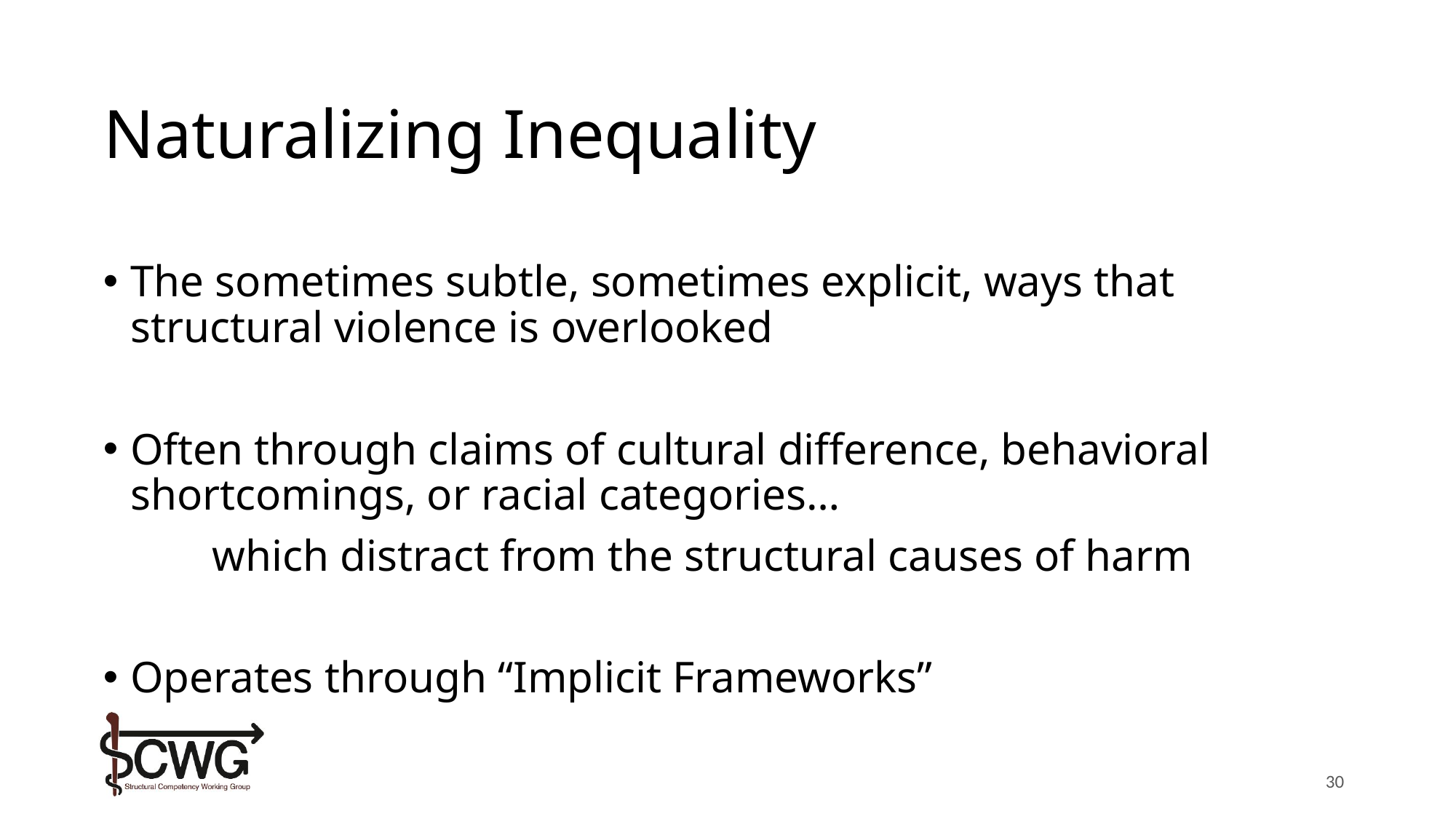

# Naturalizing Inequality
The sometimes subtle, sometimes explicit, ways that structural violence is overlooked
Often through claims of cultural difference, behavioral shortcomings, or racial categories…
	which distract from the structural causes of harm
Operates through “Implicit Frameworks”
30

## Slide 31
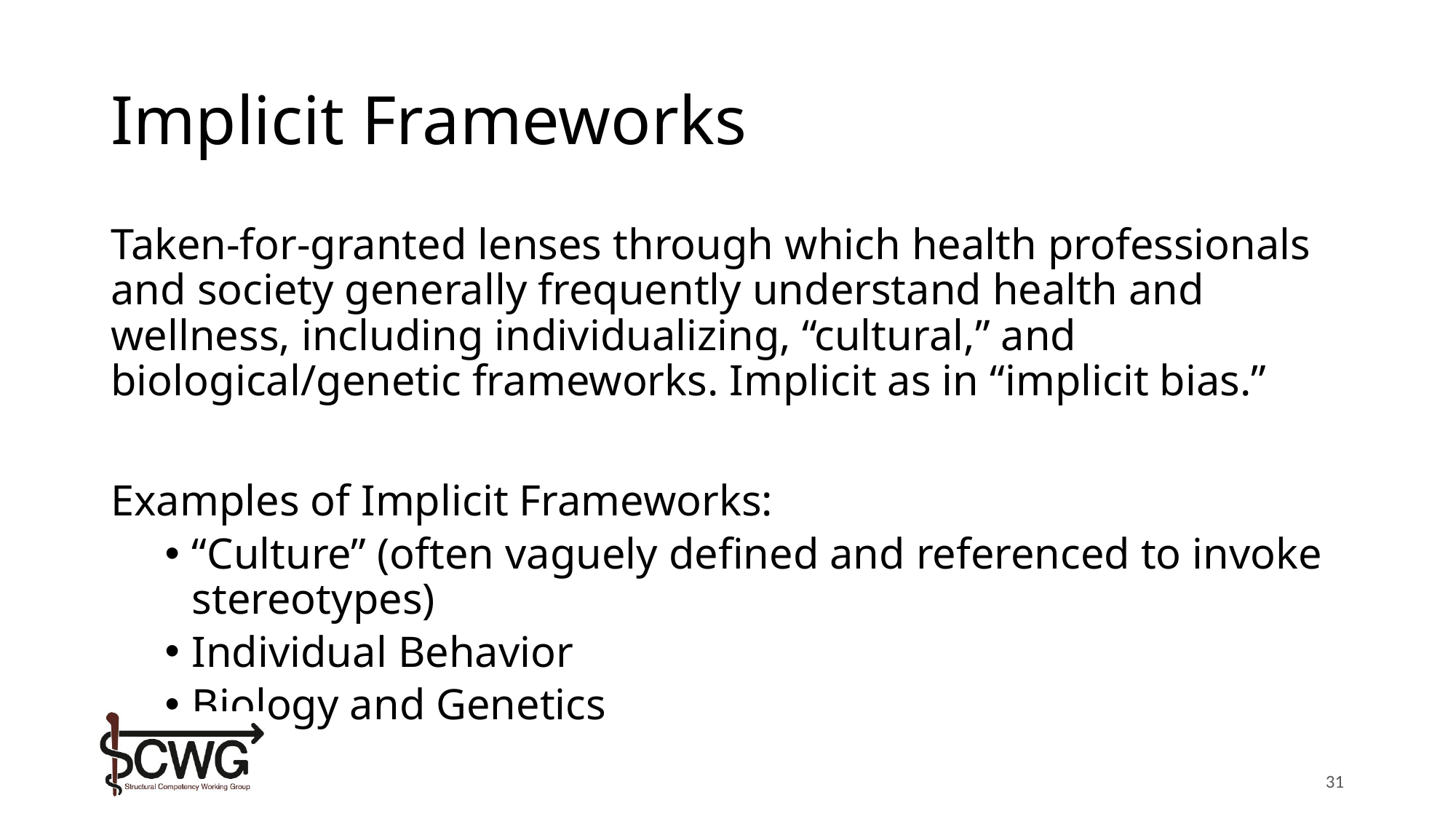

# Implicit Frameworks
Taken-for-granted lenses through which health professionals and society generally frequently understand health and wellness, including individualizing, “cultural,” and biological/genetic frameworks. Implicit as in “implicit bias.”
Examples of Implicit Frameworks:
“Culture” (often vaguely defined and referenced to invoke stereotypes)
Individual Behavior
Biology and Genetics
31

## Slide 32
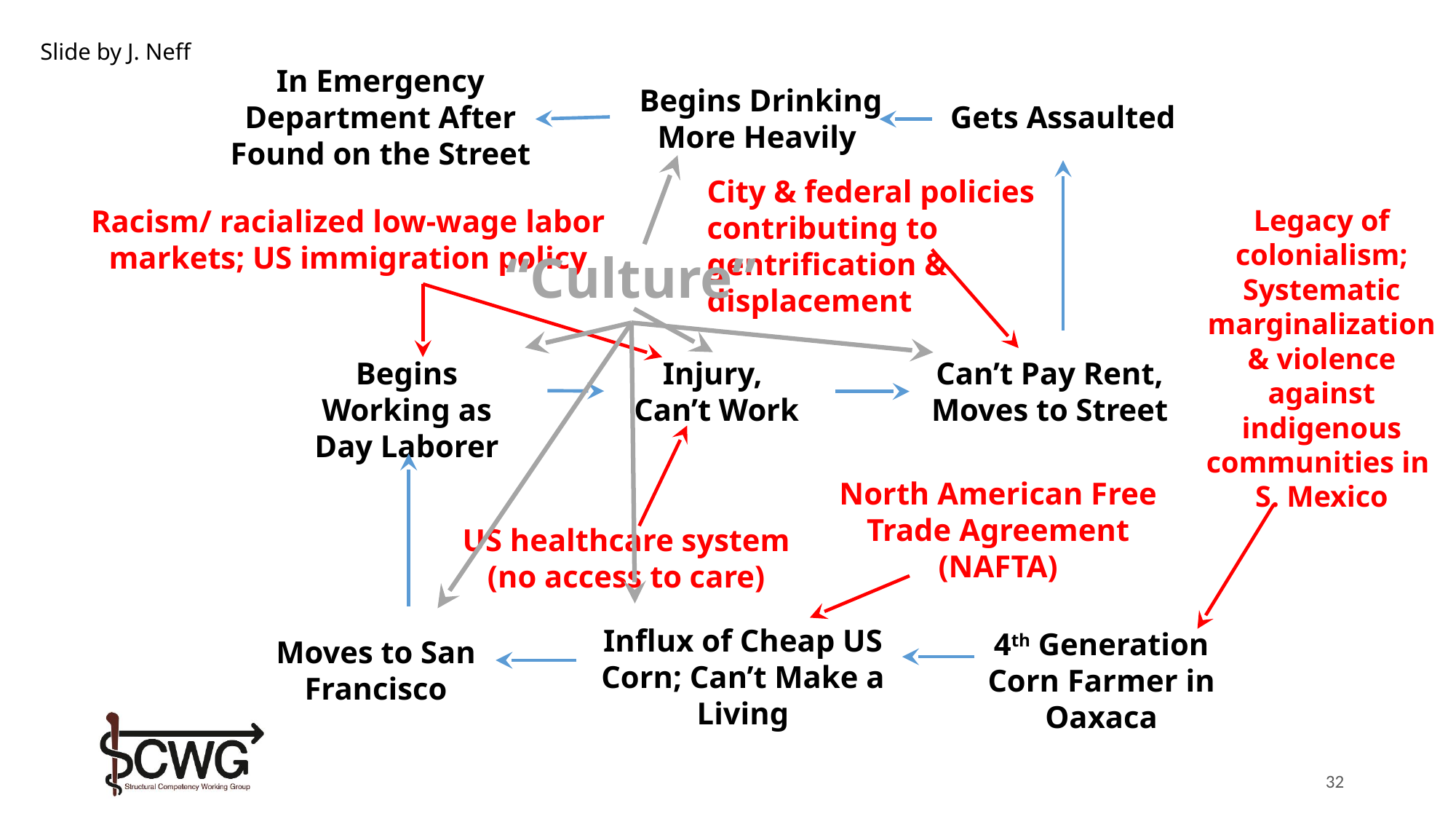

Slide by J. Neff
In Emergency Department After Found on the Street
Begins Drinking More Heavily
Gets Assaulted
City & federal policies contributing to gentrification & displacement
Racism/ racialized low-wage labor markets; US immigration policy
Legacy of colonialism; Systematic marginalization & violence against indigenous communities in
S. Mexico
“Culture”
Injury,
 Can’t Work
Can’t Pay Rent, Moves to Street
Begins Working as Day Laborer
US healthcare system (no access to care)
North American Free Trade Agreement (NAFTA)
Influx of Cheap US Corn; Can’t Make a Living
4th Generation Corn Farmer in Oaxaca
Moves to San Francisco
32

## Slide 33
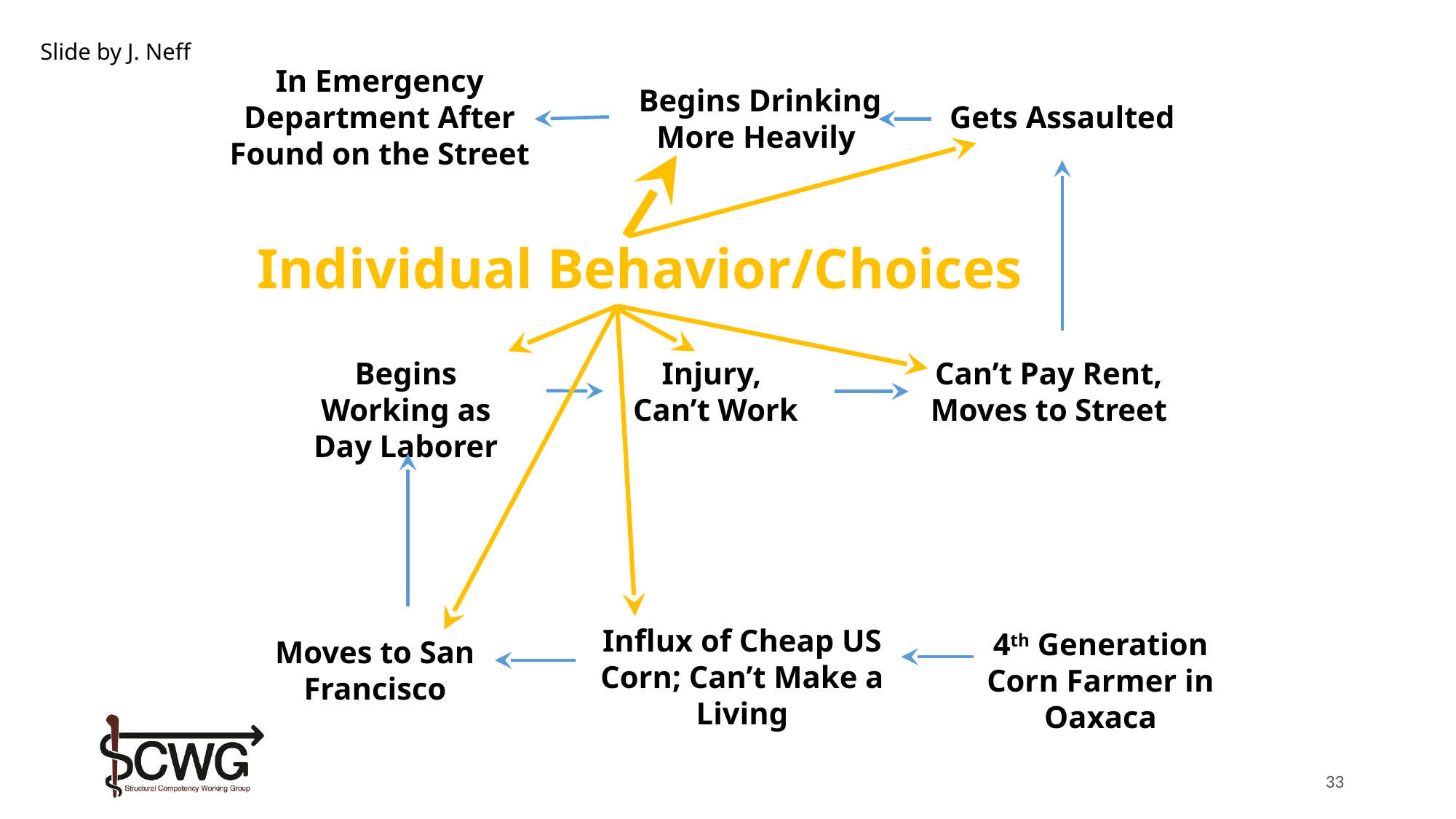

Slide by J. Neff
In Emergency Department After Found on the Street
Begins Drinking More Heavily
Gets Assaulted
Individual Behavior/Choices
Injury,
 Can’t Work
Can’t Pay Rent, Moves to Street
Begins Working as Day Laborer
Influx of Cheap US Corn; Can’t Make a Living
4th Generation Corn Farmer in Oaxaca
Moves to San Francisco
33

## Slide 34
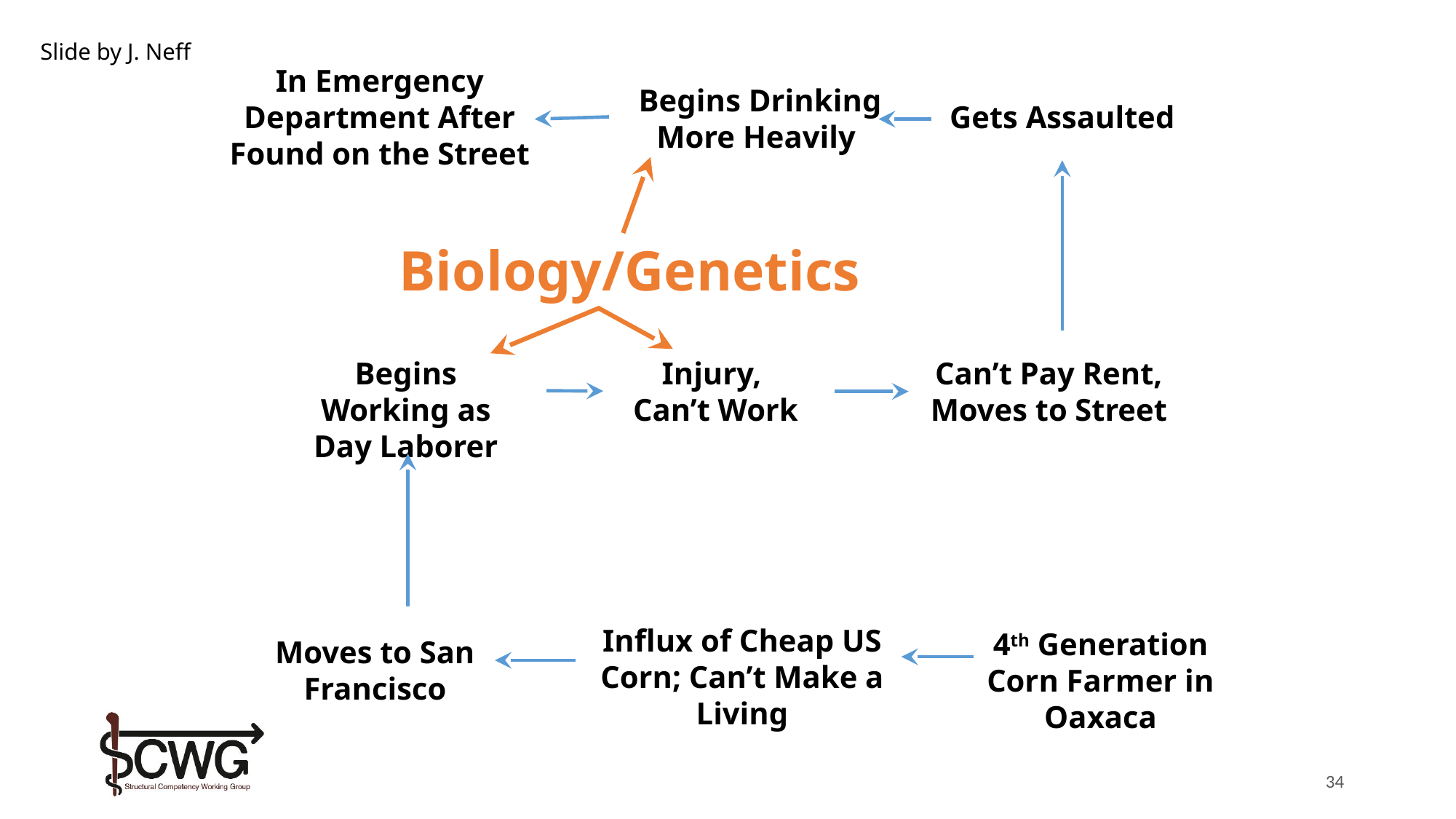

Slide by J. Neff
In Emergency Department After Found on the Street
Begins Drinking More Heavily
Gets Assaulted
Biology/Genetics
Injury,
 Can’t Work
Can’t Pay Rent, Moves to Street
Begins Working as Day Laborer
Influx of Cheap US Corn; Can’t Make a Living
4th Generation Corn Farmer in Oaxaca
Moves to San Francisco
34

## Slide 35
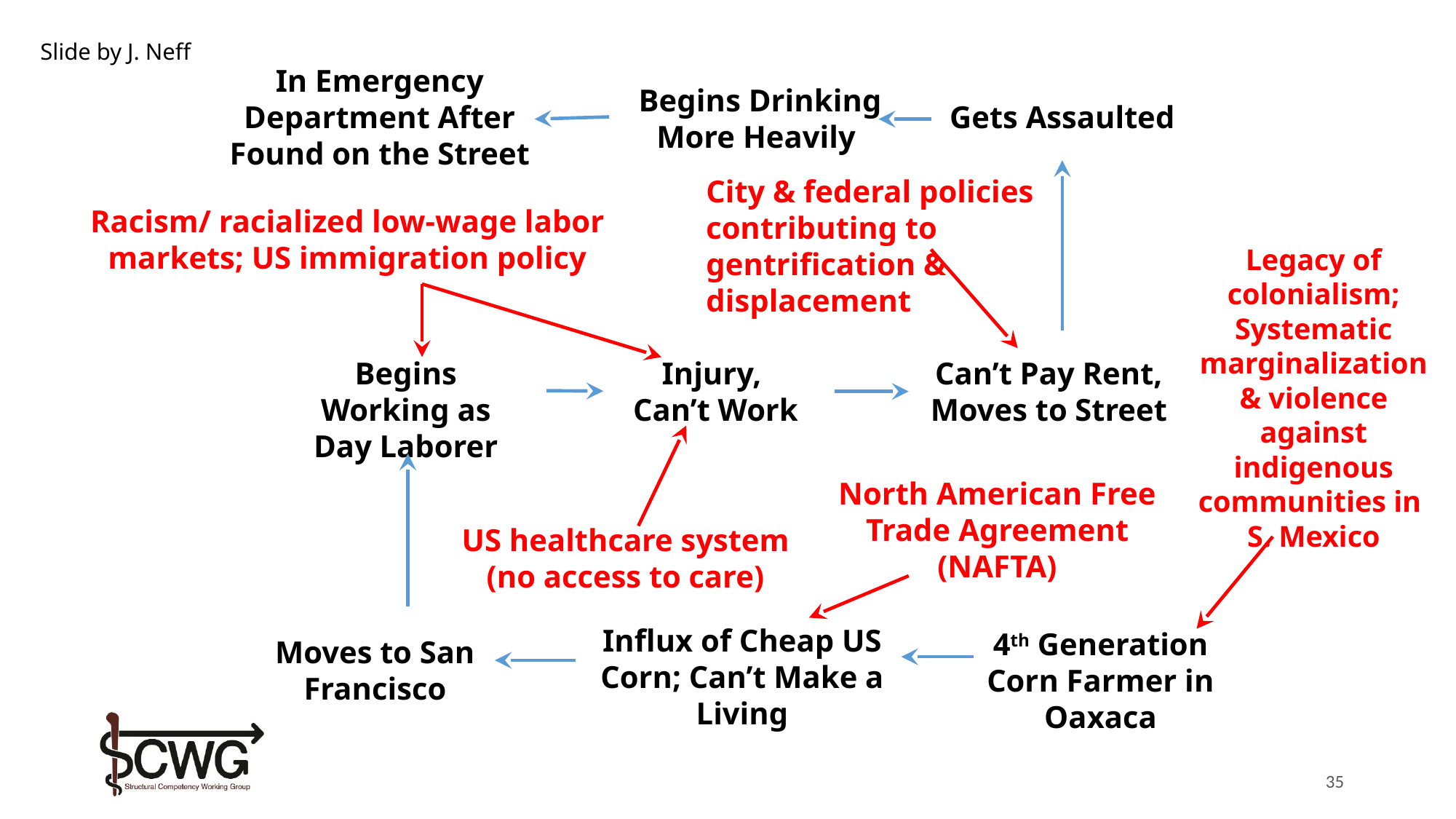

Slide by J. Neff
In Emergency Department After Found on the Street
Begins Drinking More Heavily
Gets Assaulted
City & federal policies contributing to gentrification & displacement
Racism/ racialized low-wage labor markets; US immigration policy
Legacy of colonialism; Systematic marginalization & violence against indigenous communities in
S. Mexico
Injury,
 Can’t Work
Can’t Pay Rent, Moves to Street
Begins Working as Day Laborer
US healthcare system (no access to care)
North American Free Trade Agreement (NAFTA)
Influx of Cheap US Corn; Can’t Make a Living
4th Generation Corn Farmer in Oaxaca
Moves to San Francisco
35

## Slide 36
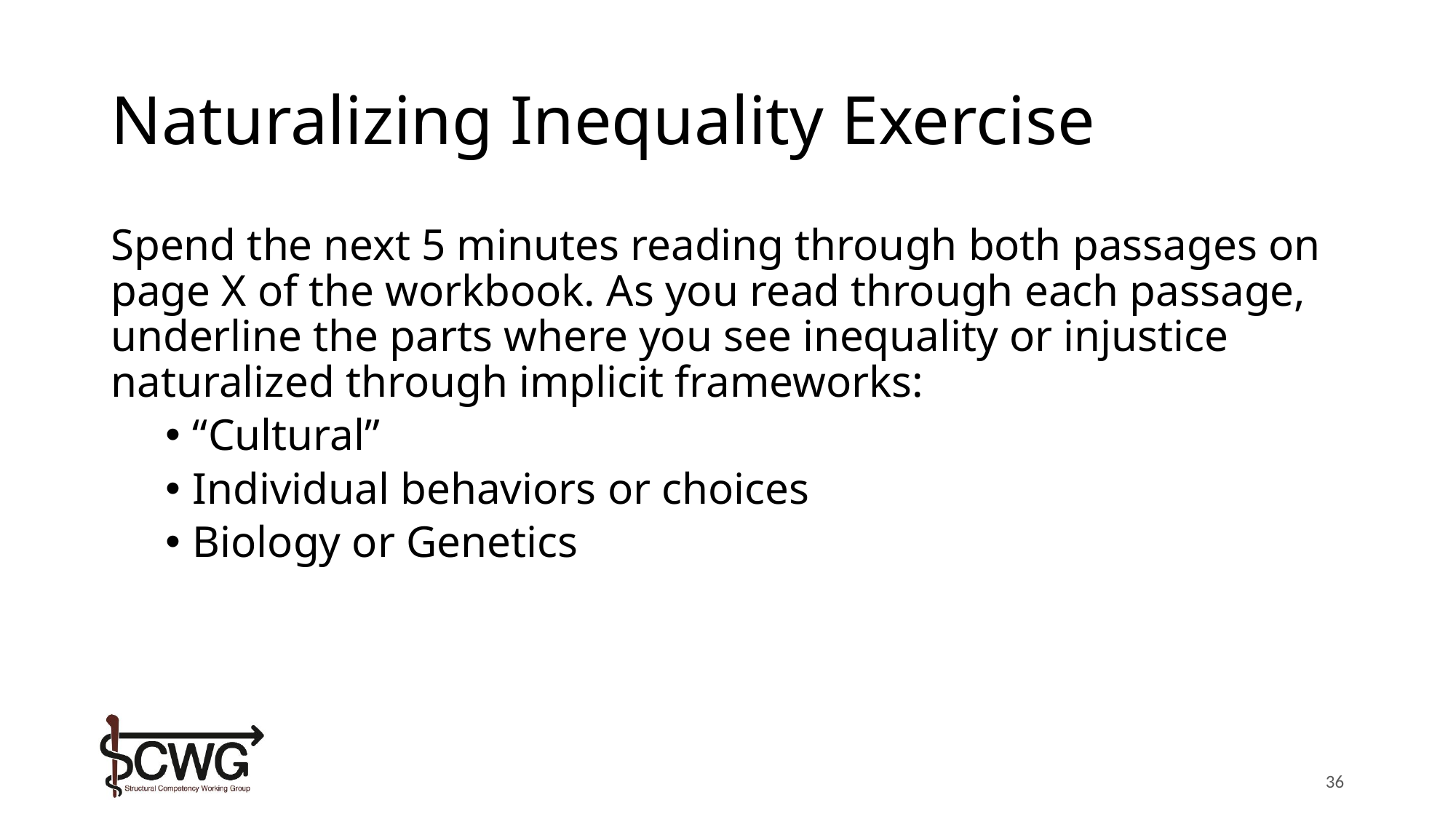

# Naturalizing Inequality Exercise
Spend the next 5 minutes reading through both passages on page X of the workbook. As you read through each passage, underline the parts where you see inequality or injustice naturalized through implicit frameworks:
“Cultural”
Individual behaviors or choices
Biology or Genetics
36

## Slide 37
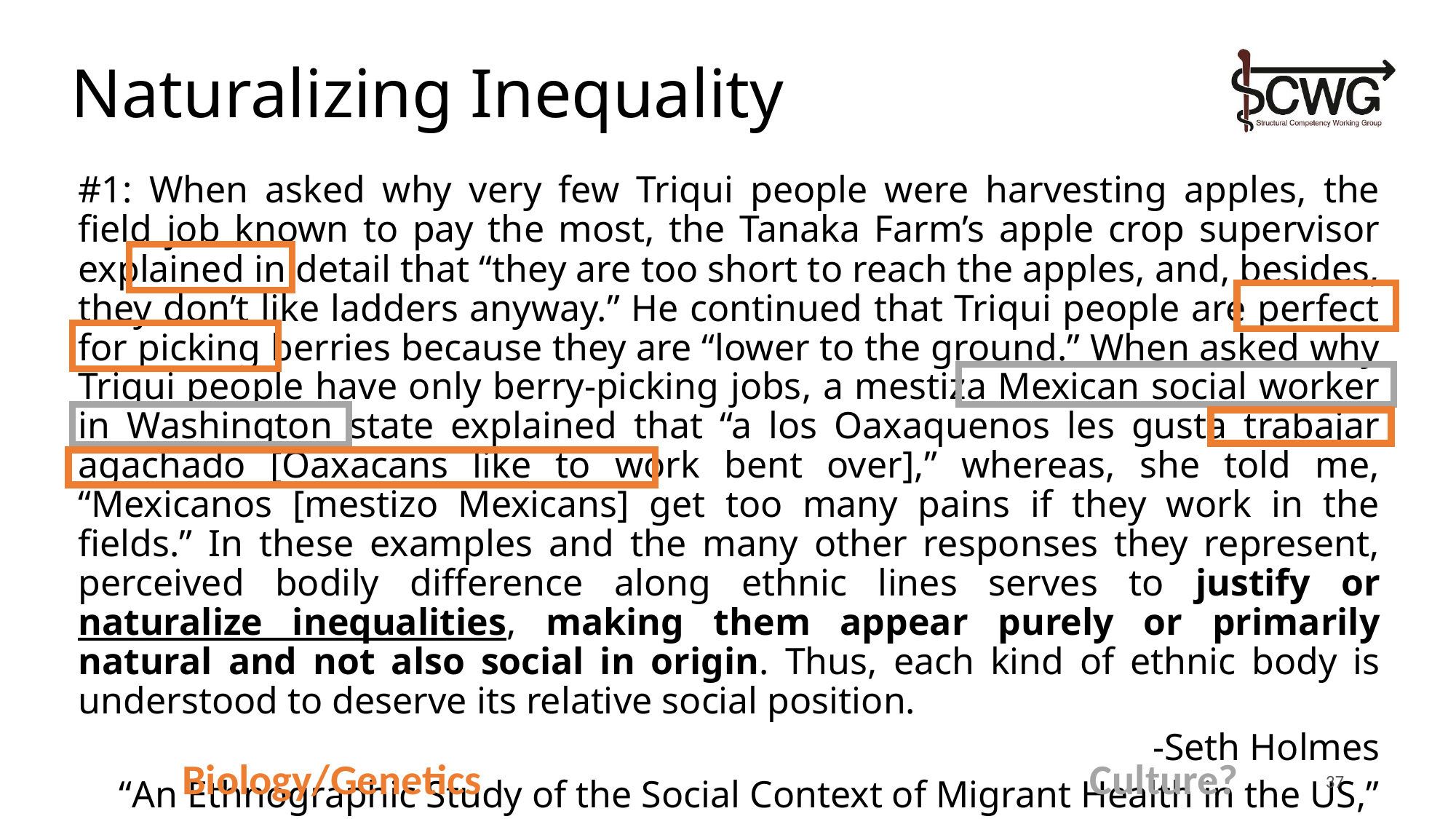

# Naturalizing Inequality
#1: When asked why very few Triqui people were harvesting apples, the field job known to pay the most, the Tanaka Farm’s apple crop supervisor explained in detail that “they are too short to reach the apples, and, besides, they don’t like ladders anyway.” He continued that Triqui people are perfect for picking berries because they are “lower to the ground.” When asked why Triqui people have only berry-picking jobs, a mestiza Mexican social worker in Washington state explained that “a los Oaxaquenos les gusta trabajar agachado [Oaxacans like to work bent over],” whereas, she told me, “Mexicanos [mestizo Mexicans] get too many pains if they work in the fields.” In these examples and the many other responses they represent, perceived bodily difference along ethnic lines serves to justify or naturalize inequalities, making them appear purely or primarily natural and not also social in origin. Thus, each kind of ethnic body is understood to deserve its relative social position.
-Seth Holmes
“An Ethnographic Study of the Social Context of Migrant Health in the US,” 2006
Culture?
Biology/Genetics
37

## Slide 38
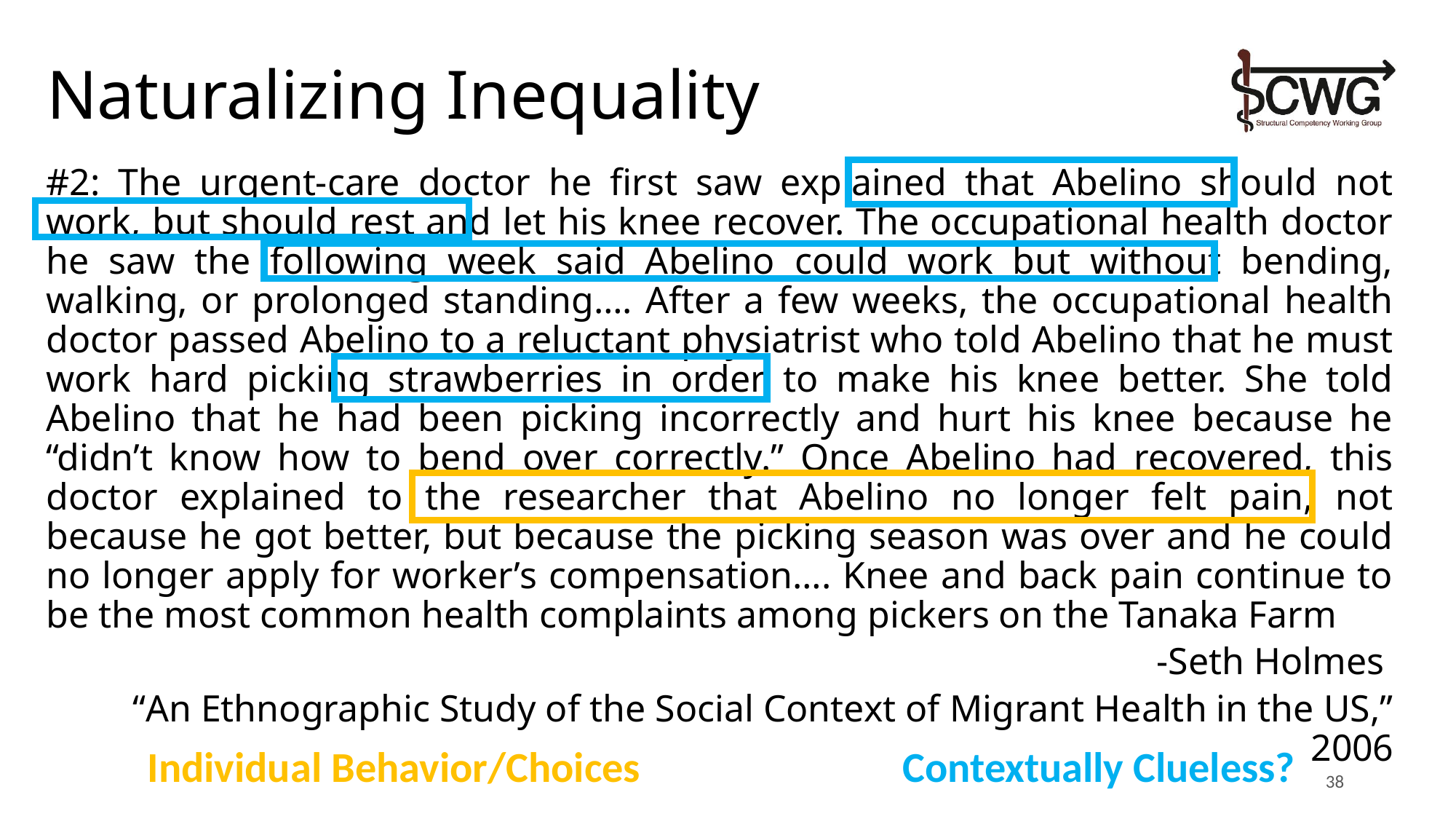

# Naturalizing Inequality
#2: The urgent-care doctor he first saw explained that Abelino should not work, but should rest and let his knee recover. The occupational health doctor he saw the following week said Abelino could work but without bending, walking, or prolonged standing…. After a few weeks, the occupational health doctor passed Abelino to a reluctant physiatrist who told Abelino that he must work hard picking strawberries in order to make his knee better. She told Abelino that he had been picking incorrectly and hurt his knee because he “didn’t know how to bend over correctly.” Once Abelino had recovered, this doctor explained to the researcher that Abelino no longer felt pain, not because he got better, but because the picking season was over and he could no longer apply for worker’s compensation…. Knee and back pain continue to be the most common health complaints among pickers on the Tanaka Farm
-Seth Holmes
“An Ethnographic Study of the Social Context of Migrant Health in the US,” 2006
Contextually Clueless?
Individual Behavior/Choices
38

## Slide 39
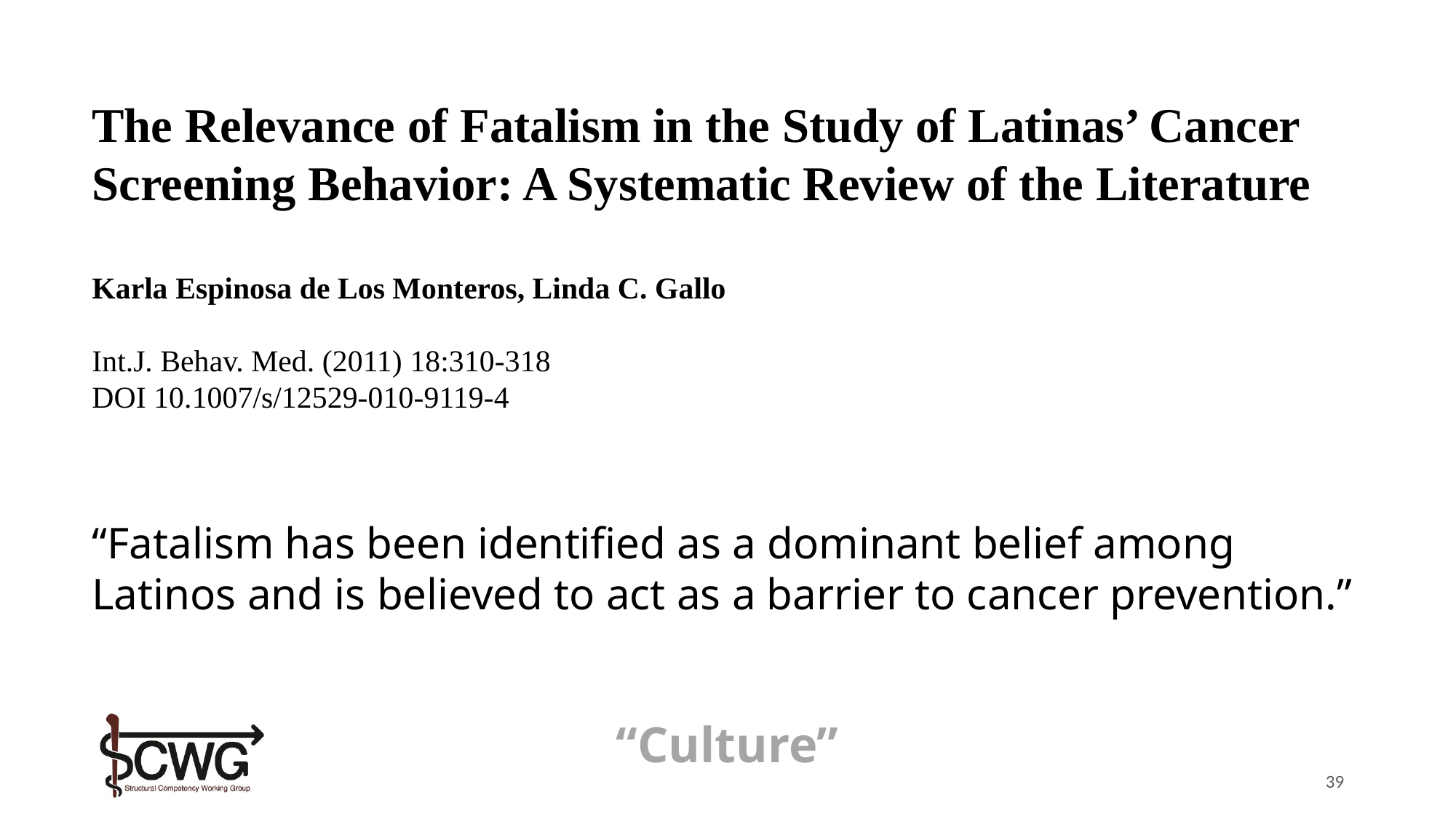

The Relevance of Fatalism in the Study of Latinas’ Cancer Screening Behavior: A Systematic Review of the Literature
Karla Espinosa de Los Monteros, Linda C. Gallo
Int.J. Behav. Med. (2011) 18:310-318
DOI 10.1007/s/12529-010-9119-4
“Fatalism has been identified as a dominant belief among Latinos and is believed to act as a barrier to cancer prevention.”
“Culture”
39

## Slide 40
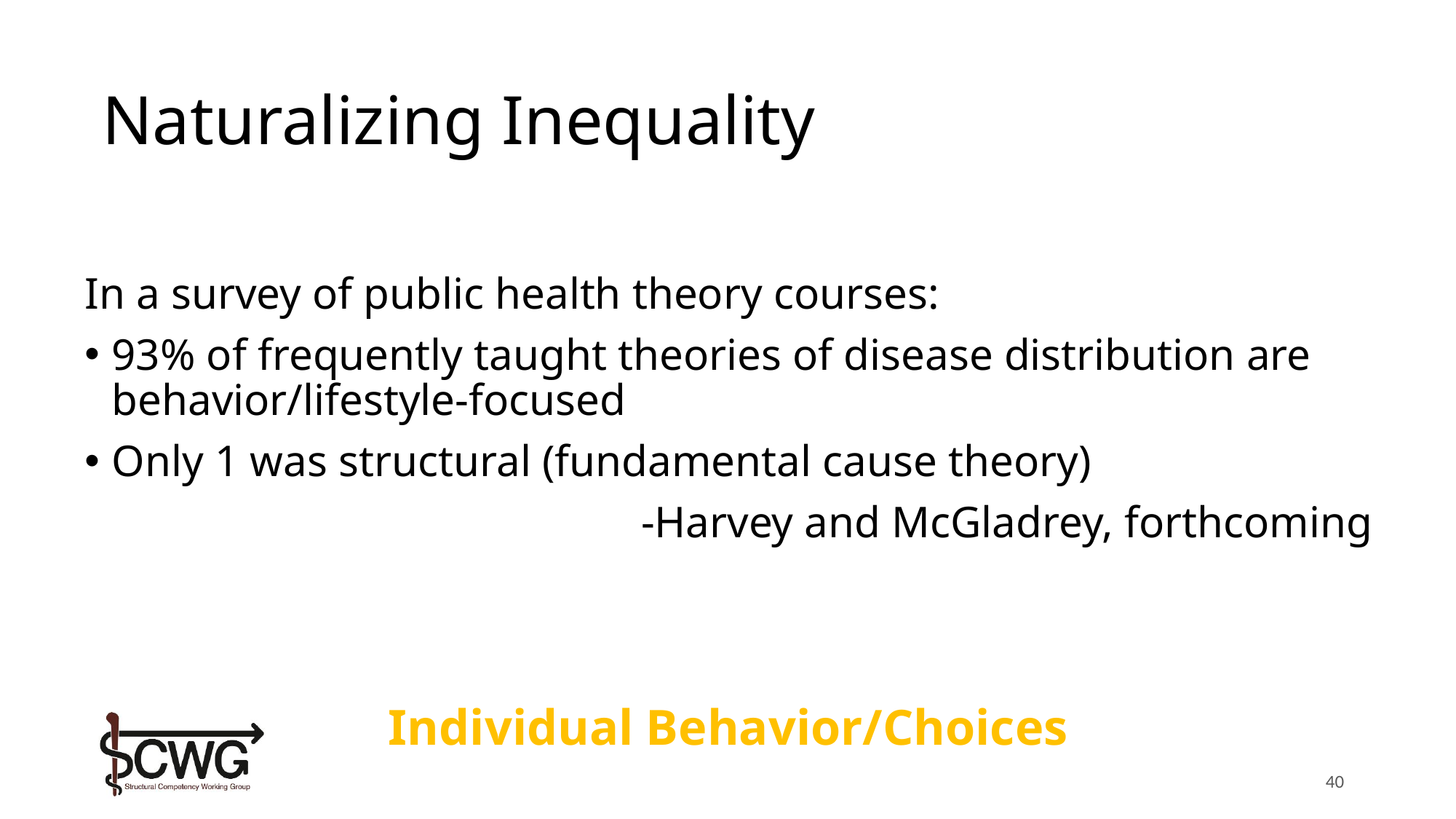

# Naturalizing Inequality
In a survey of public health theory courses:
93% of frequently taught theories of disease distribution are behavior/lifestyle-focused
Only 1 was structural (fundamental cause theory)
-Harvey and McGladrey, forthcoming
Individual Behavior/Choices
40

## Slide 41
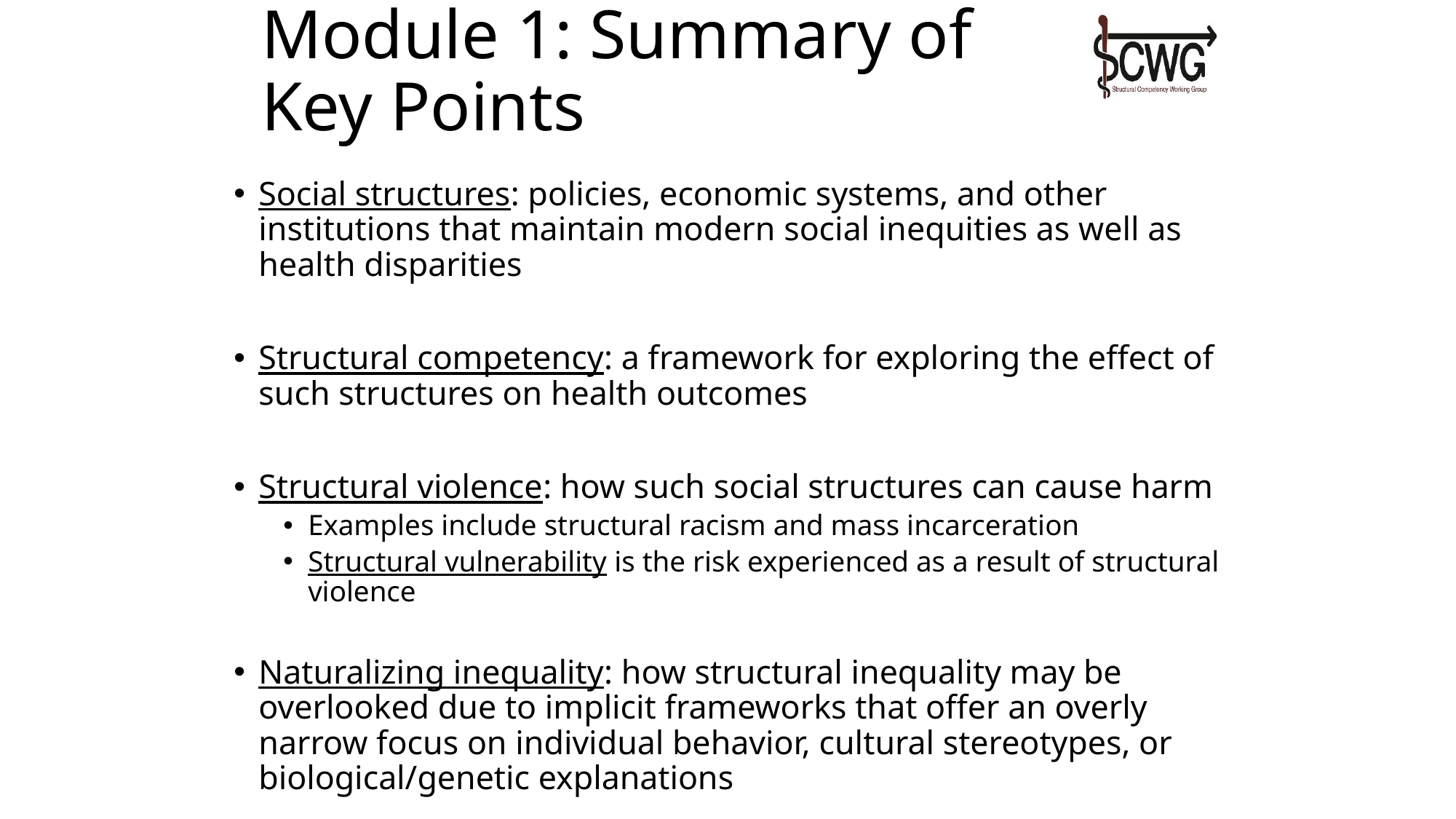

# Module 1: Summary of Key Points
Social structures: policies, economic systems, and other institutions that maintain modern social inequities as well as health disparities
Structural competency: a framework for exploring the effect of such structures on health outcomes
Structural violence: how such social structures can cause harm
Examples include structural racism and mass incarceration
Structural vulnerability is the risk experienced as a result of structural violence
Naturalizing inequality: how structural inequality may be overlooked due to implicit frameworks that offer an overly narrow focus on individual behavior, cultural stereotypes, or biological/genetic explanations
