# Supplementary material for: Structural Competency: Curriculum for Medical Students, Residents, and Interprofessional Teams on the Structural Factors That Produce Health Disparities
Source: MedEdPORTAL. 2020 Mar 13;16:10888. doi: 10.15766/mep_2374-8265.10888 (PMC7182045; doi:10.15766/mep_2374-8265.10888)
Supplement: Supplementary file 1 — A. Manual Background Info.docx B. Manual Intro.docx C. Manual Module 1.docx D. Manual Module 2.docx E. Manual Module 3.docx F. Manual Conclusion and Evaluation.docx G. Supplemental Reading List.docx H. Training Slides Intro.pptx I. Training Slides Module 1.pptx J. Training Slides Module 2.pptx K. Training Slides Module 3.pptx L. Participant Workbook.pdf M. Posttraining Survey.pdf N. Facilitator Guidelines.docx O. Facilitator Preparation - Terms and Concepts.docx P. Participant Sign-in Sheet.docx [file mep-16-10888-s001.zip › J. Training Slides Module 2.pptx]

## Slide 1
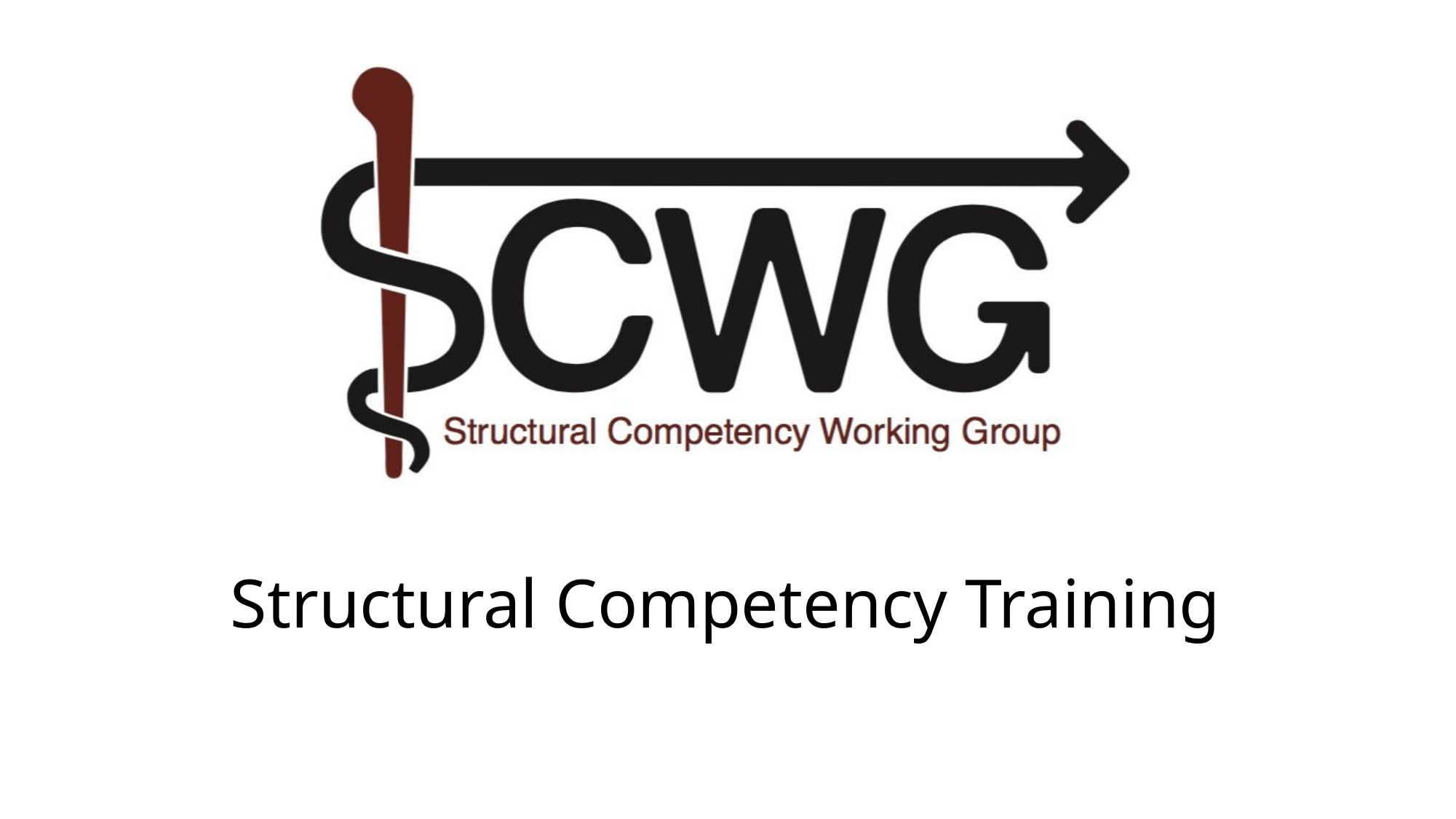

Structural Competency Training

## Slide 2
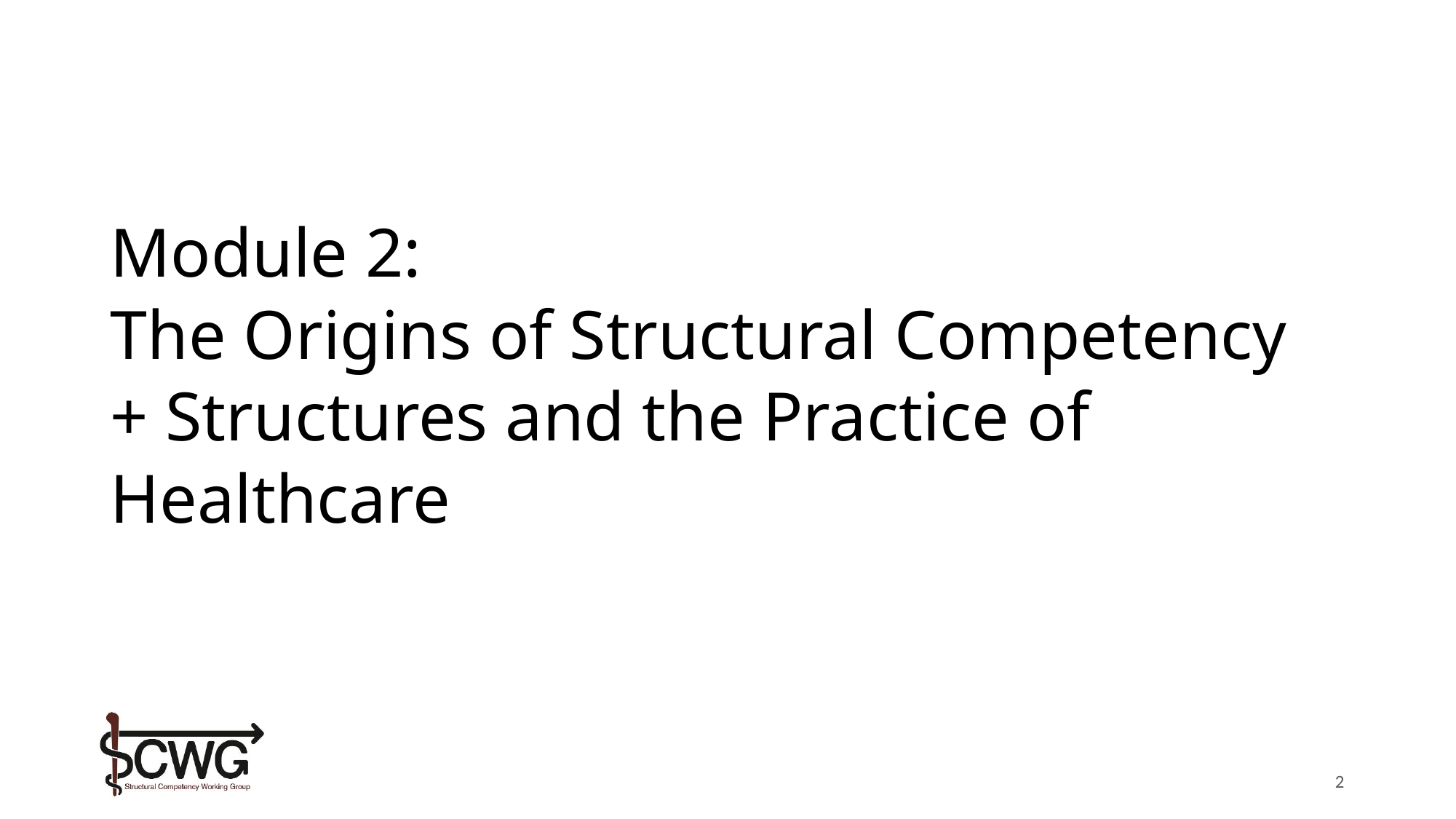

# Module 2: The Origins of Structural Competency + Structures and the Practice of Healthcare
2

## Slide 3
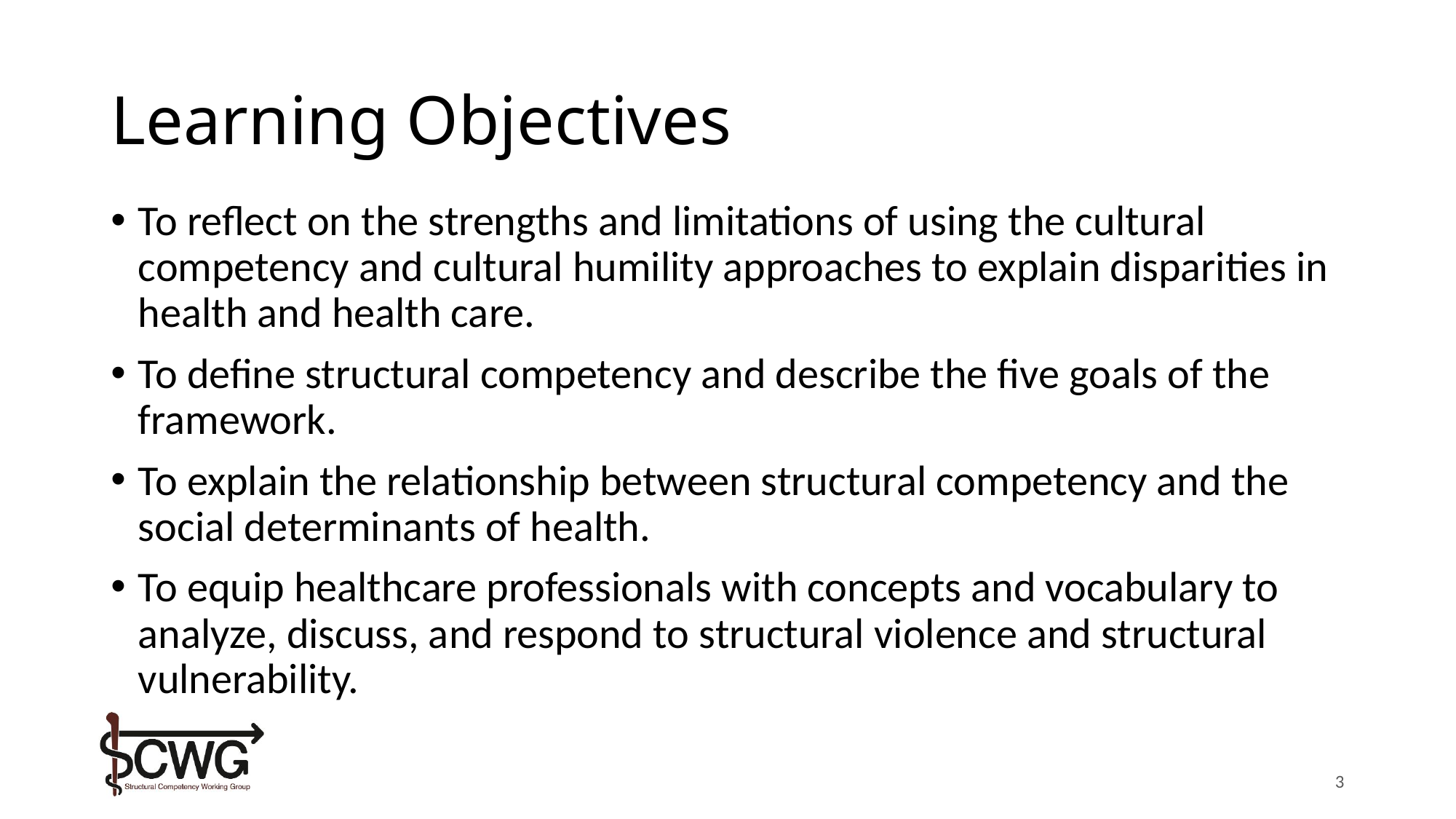

# Learning Objectives
To reflect on the strengths and limitations of using the cultural competency and cultural humility approaches to explain disparities in health and health care.
To define structural competency and describe the five goals of the framework.
To explain the relationship between structural competency and the social determinants of health.
To equip healthcare professionals with concepts and vocabulary to analyze, discuss, and respond to structural violence and structural vulnerability.
3

## Slide 4
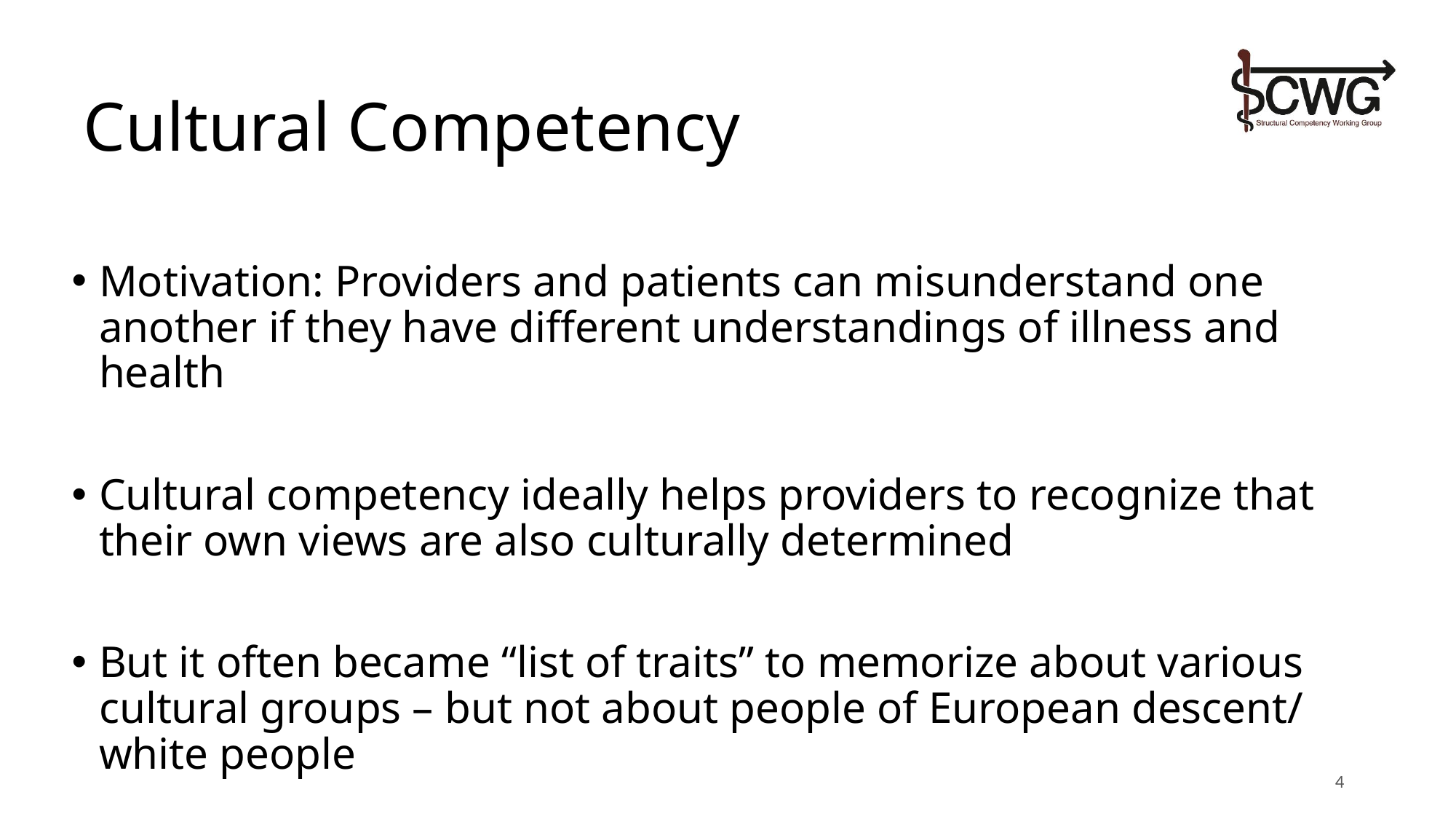

# Cultural Competency
Motivation: Providers and patients can misunderstand one another if they have different understandings of illness and health
Cultural competency ideally helps providers to recognize that their own views are also culturally determined
But it often became “list of traits” to memorize about various cultural groups – but not about people of European descent/ white people
4

## Slide 5
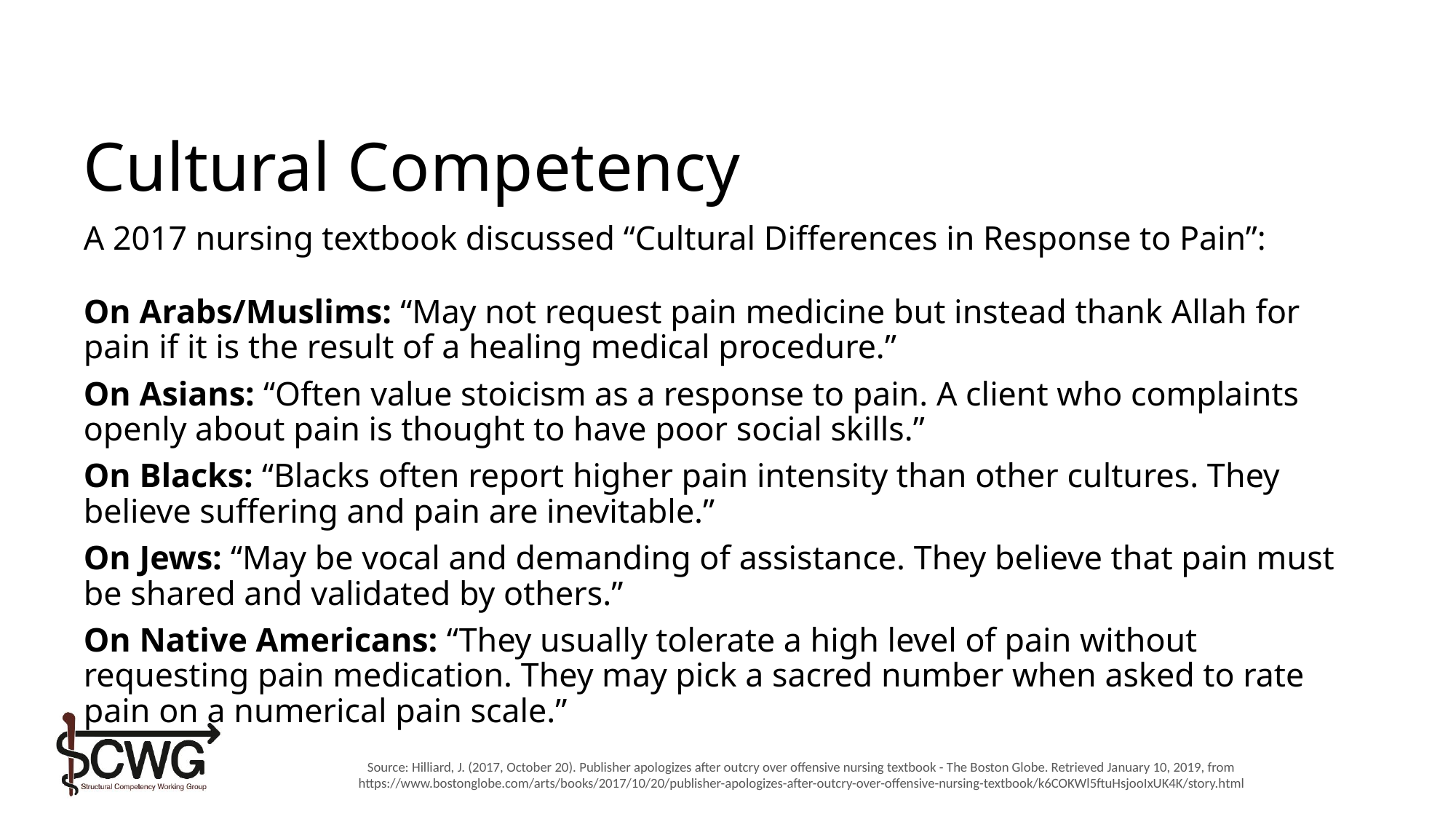

# Cultural Competency
A 2017 nursing textbook discussed “Cultural Differences in Response to Pain”:
On Arabs/Muslims: “May not request pain medicine but instead thank Allah for pain if it is the result of a healing medical procedure.”
On Asians: “Often value stoicism as a response to pain. A client who complaints openly about pain is thought to have poor social skills.”
On Blacks: “Blacks often report higher pain intensity than other cultures. They believe suffering and pain are inevitable.”
On Jews: “May be vocal and demanding of assistance. They believe that pain must be shared and validated by others.”
On Native Americans: “They usually tolerate a high level of pain without requesting pain medication. They may pick a sacred number when asked to rate pain on a numerical pain scale.”
Source: Hilliard, J. (2017, October 20). Publisher apologizes after outcry over offensive nursing textbook - The Boston Globe. Retrieved January 10, 2019, from https://www.bostonglobe.com/arts/books/2017/10/20/publisher-apologizes-after-outcry-over-offensive-nursing-textbook/k6COKWl5ftuHsjooIxUK4K/story.html

## Slide 6
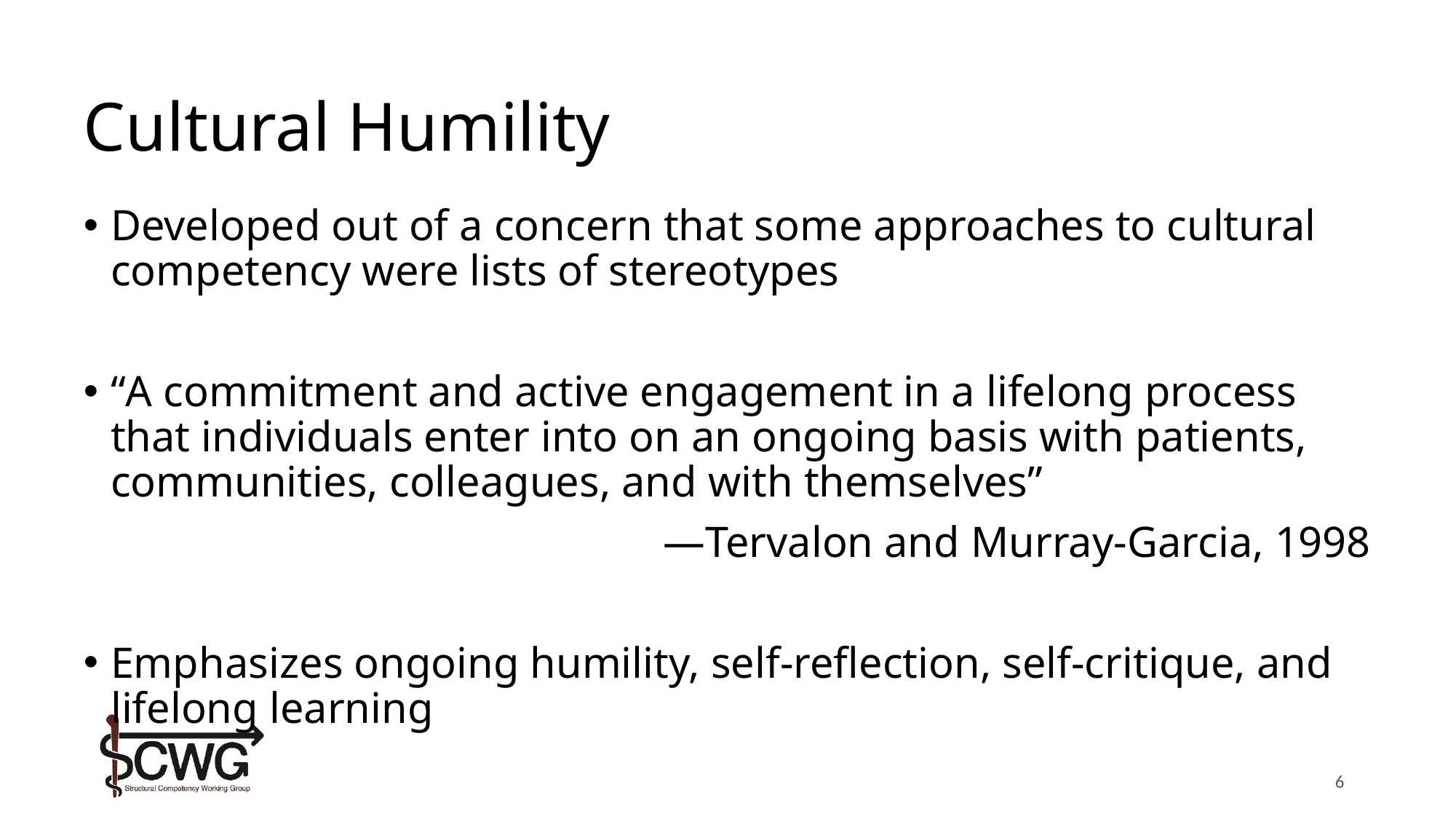

# Cultural Humility
Developed out of a concern that some approaches to cultural competency were lists of stereotypes
“A commitment and active engagement in a lifelong process that individuals enter into on an ongoing basis with patients, communities, colleagues, and with themselves”
—Tervalon and Murray-Garcia, 1998
Emphasizes ongoing humility, self-reflection, self-critique, and lifelong learning
6

## Slide 7
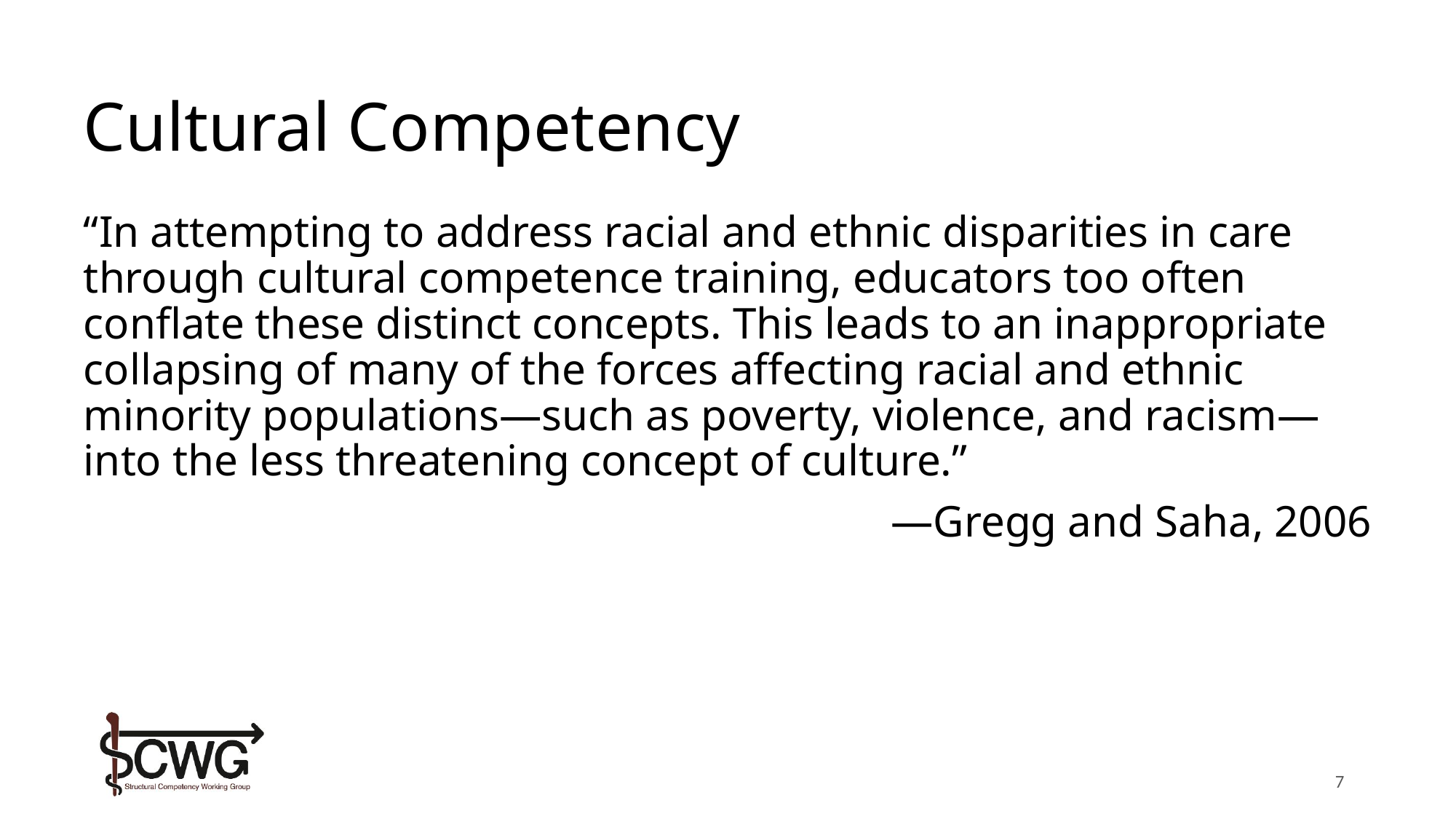

# Cultural Competency
“In attempting to address racial and ethnic disparities in care through cultural competence training, educators too often conflate these distinct concepts. This leads to an inappropriate collapsing of many of the forces affecting racial and ethnic minority populations—such as poverty, violence, and racism—into the less threatening concept of culture.”
—Gregg and Saha, 2006
7

## Slide 8
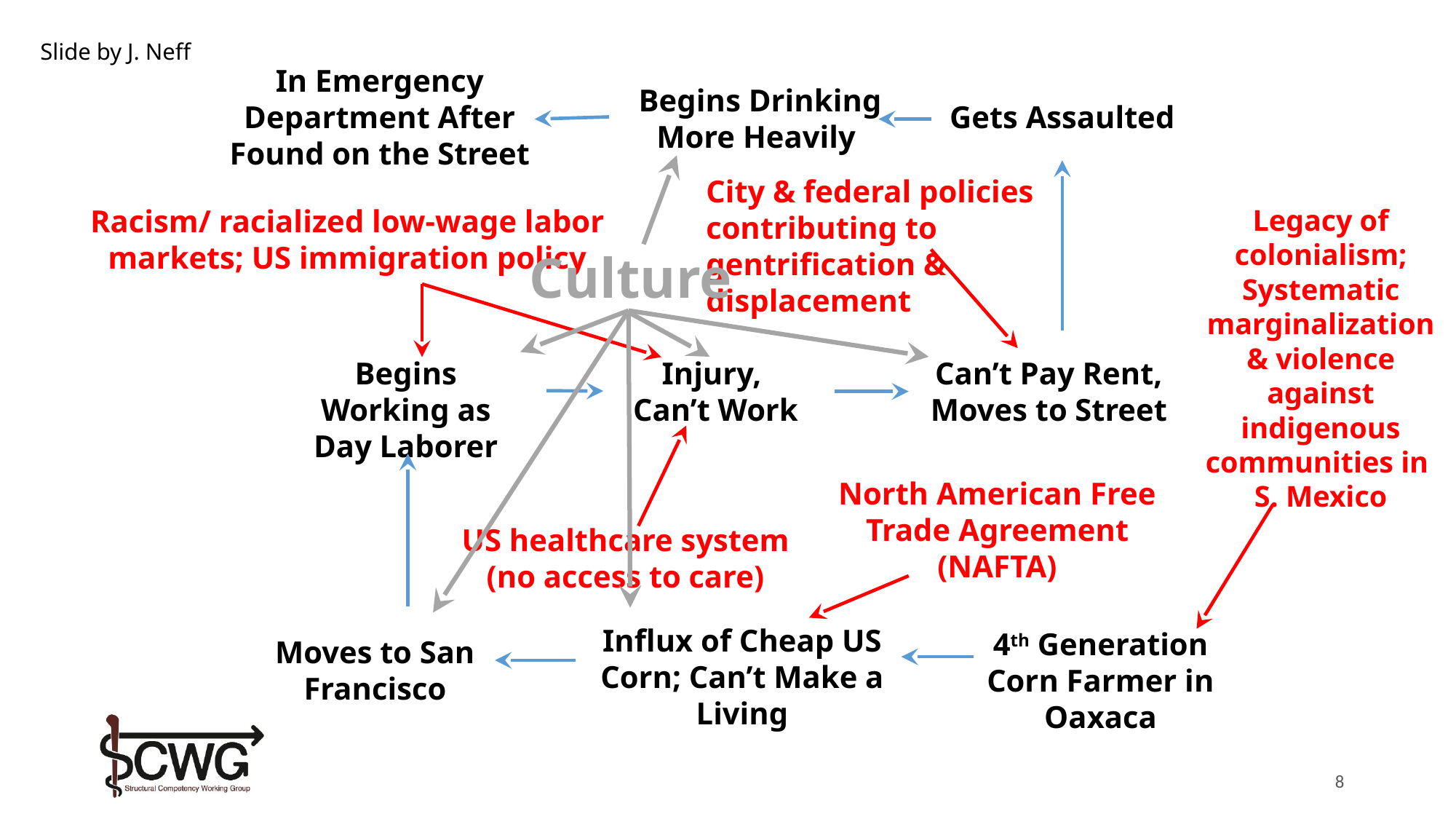

Slide by J. Neff
In Emergency Department After Found on the Street
Begins Drinking More Heavily
Gets Assaulted
Culture
City & federal policies contributing to gentrification & displacement
Racism/ racialized low-wage labor markets; US immigration policy
Legacy of colonialism; Systematic marginalization & violence against indigenous communities in
S. Mexico
Injury,
 Can’t Work
Can’t Pay Rent, Moves to Street
Begins Working as Day Laborer
US healthcare system (no access to care)
North American Free Trade Agreement (NAFTA)
Influx of Cheap US Corn; Can’t Make a Living
4th Generation Corn Farmer in Oaxaca
Moves to San Francisco
8

## Slide 9
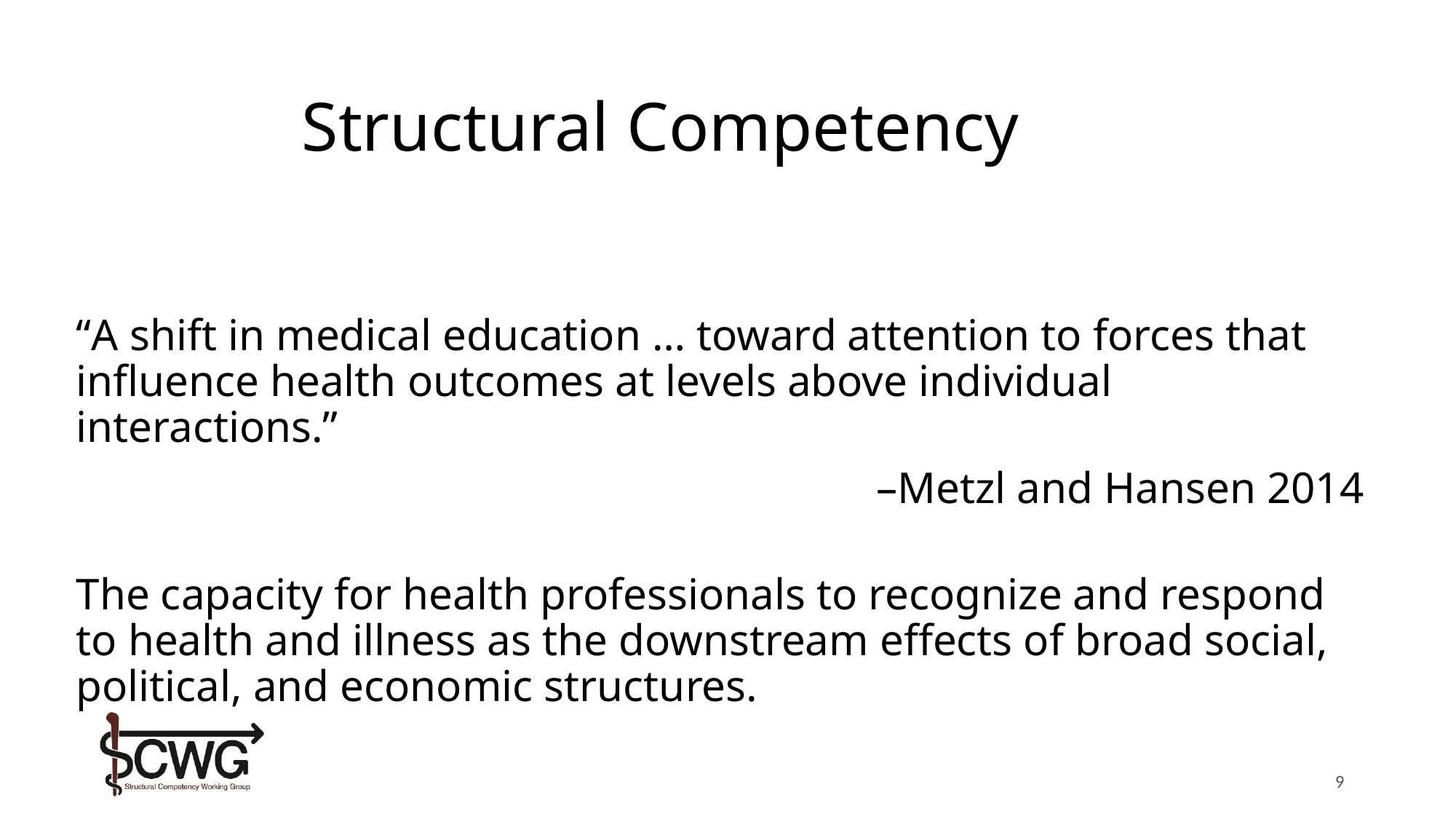

# Structural Competency
“A shift in medical education … toward attention to forces that influence health outcomes at levels above individual interactions.”
–Metzl and Hansen 2014
The capacity for health professionals to recognize and respond to health and illness as the downstream effects of broad social, political, and economic structures.
9

## Slide 10
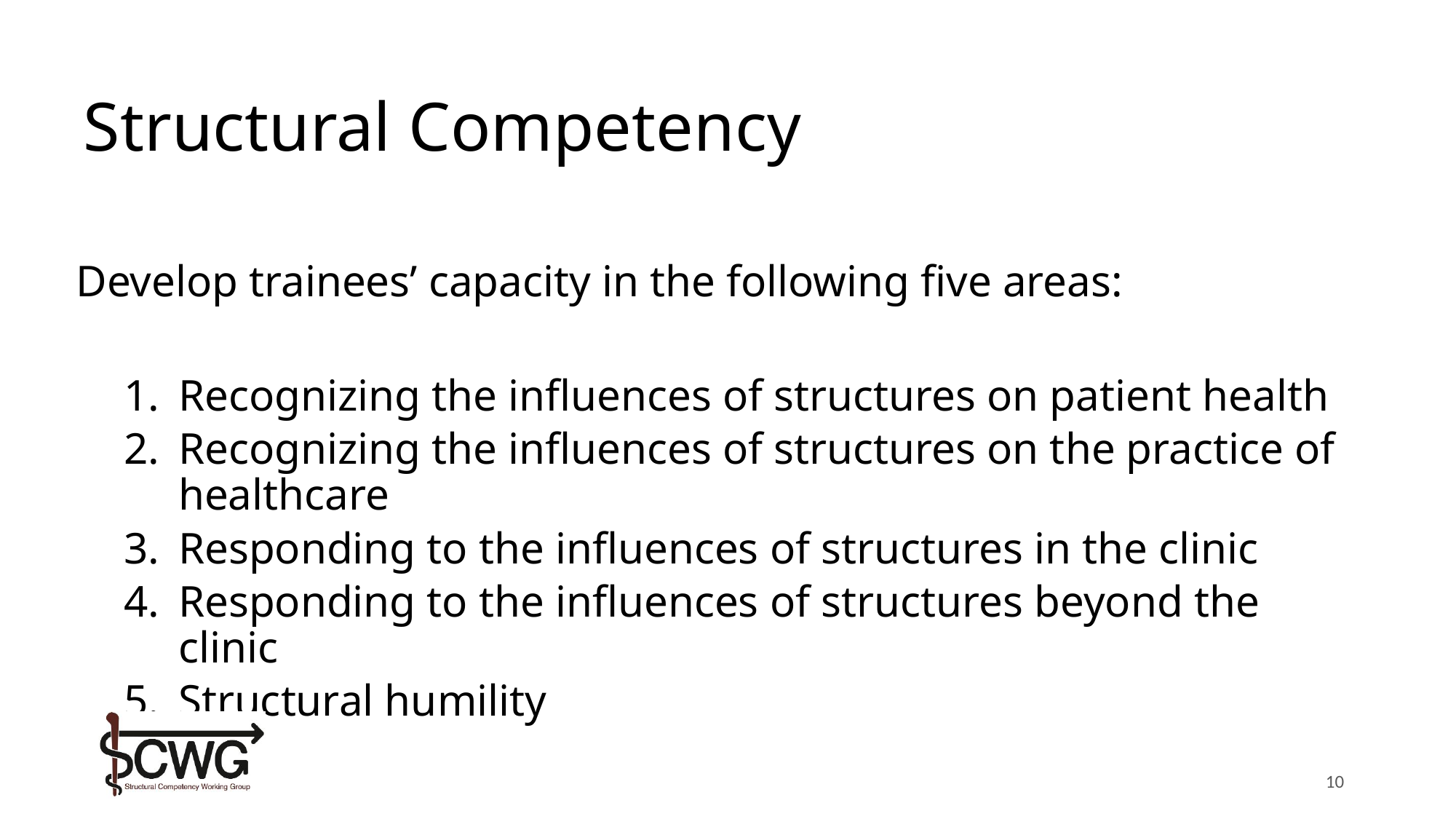

# Structural Competency
Develop trainees’ capacity in the following five areas:
Recognizing the influences of structures on patient health
Recognizing the influences of structures on the practice of healthcare
Responding to the influences of structures in the clinic
Responding to the influences of structures beyond the clinic
Structural humility
10

## Slide 11
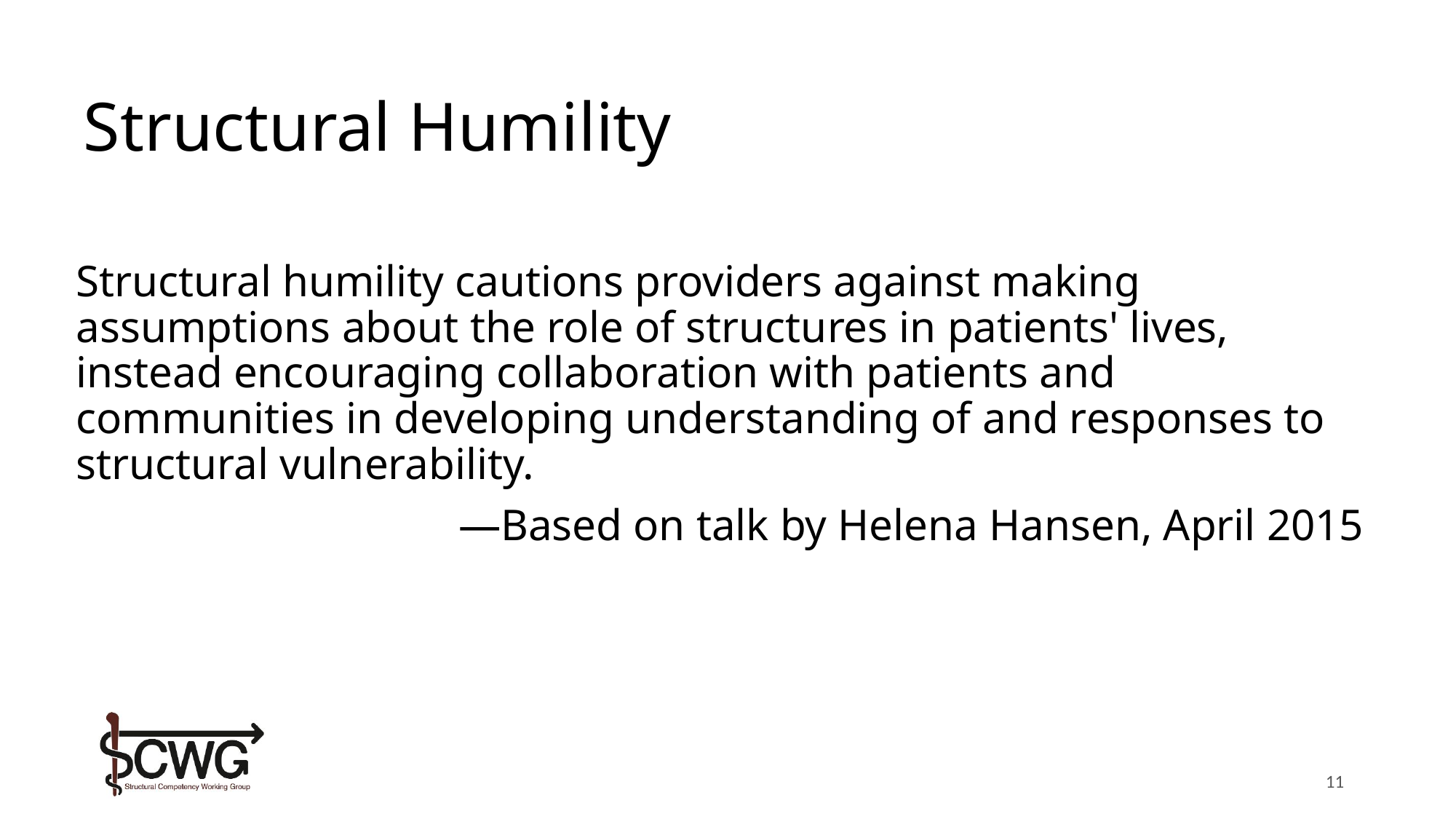

# Structural Humility
Structural humility cautions providers against making assumptions about the role of structures in patients' lives, instead encouraging collaboration with patients and communities in developing understanding of and responses to structural vulnerability.
—Based on talk by Helena Hansen, April 2015
11

## Slide 12
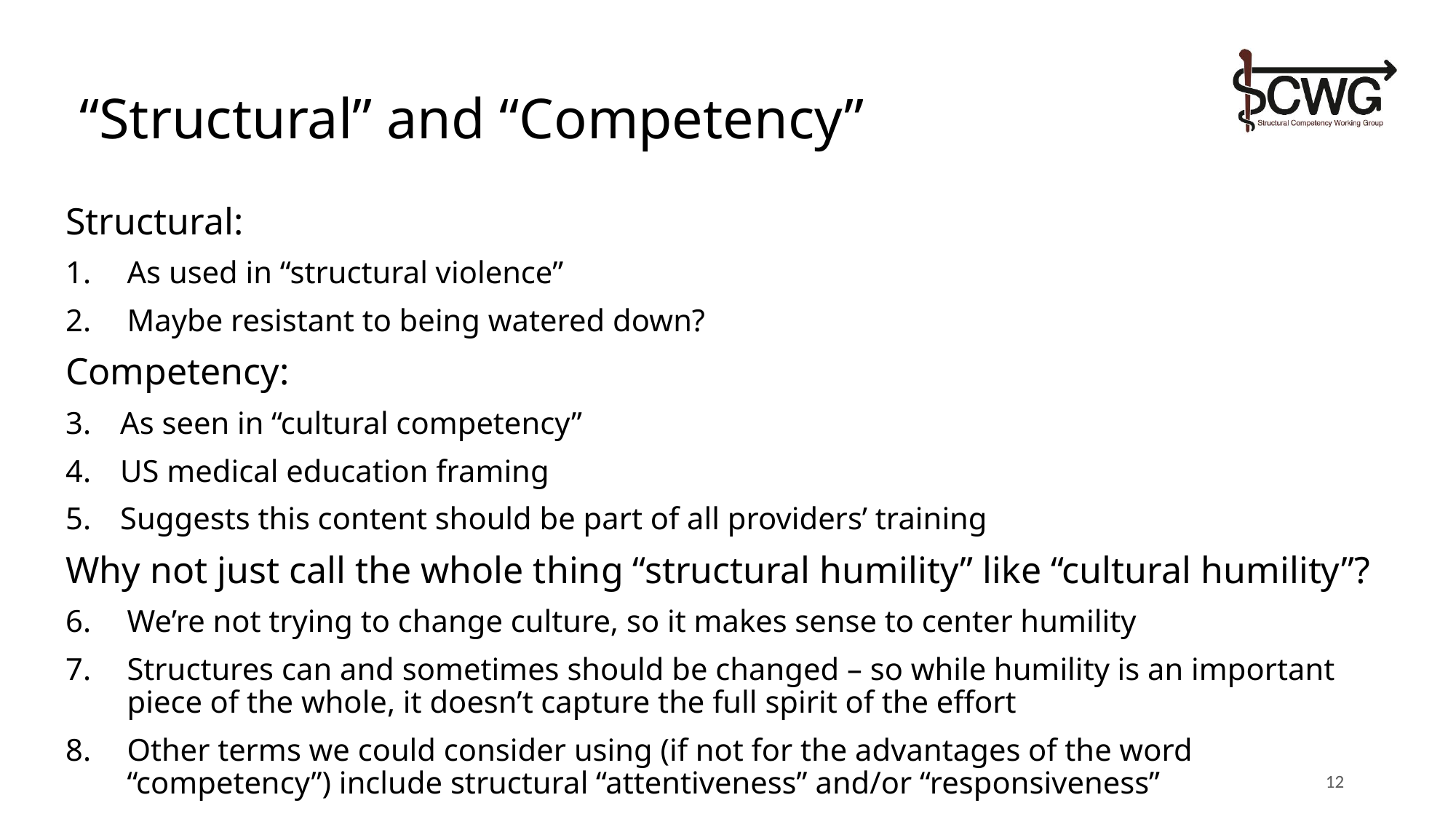

# “Structural” and “Competency”
Structural:
As used in “structural violence”
Maybe resistant to being watered down?
Competency:
As seen in “cultural competency”
US medical education framing
Suggests this content should be part of all providers’ training
Why not just call the whole thing “structural humility” like “cultural humility”?
We’re not trying to change culture, so it makes sense to center humility
Structures can and sometimes should be changed – so while humility is an important piece of the whole, it doesn’t capture the full spirit of the effort
Other terms we could consider using (if not for the advantages of the word “competency”) include structural “attentiveness” and/or “responsiveness”
12

## Slide 13
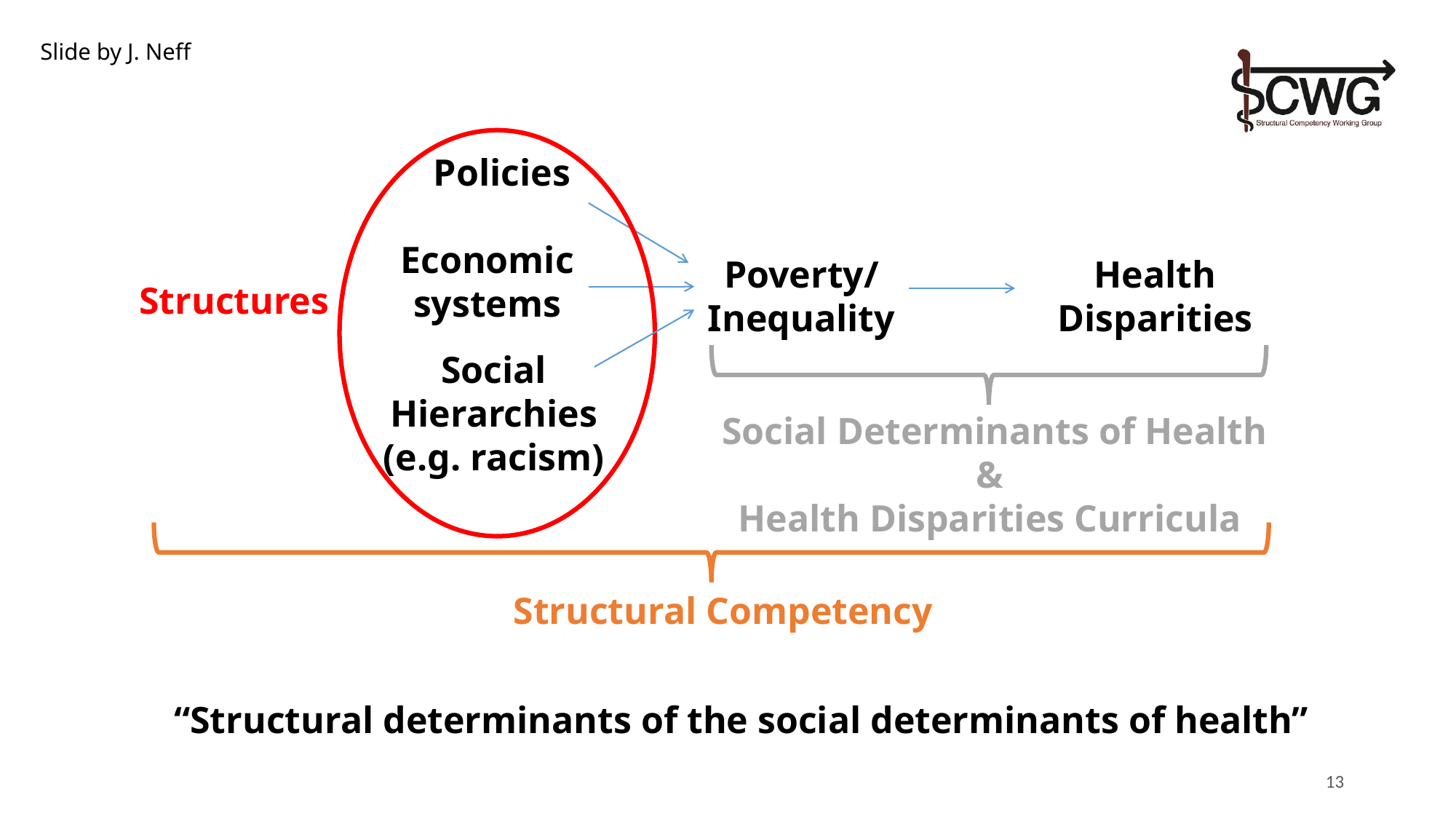

Slide by J. Neff
#
Structures
Policies
Economic systems
Poverty/ Inequality
Health Disparities
Social Hierarchies
(e.g. racism)
Social Determinants of Health &
Health Disparities Curricula
Structural Competency
“Structural determinants of the social determinants of health”
13

## Slide 14
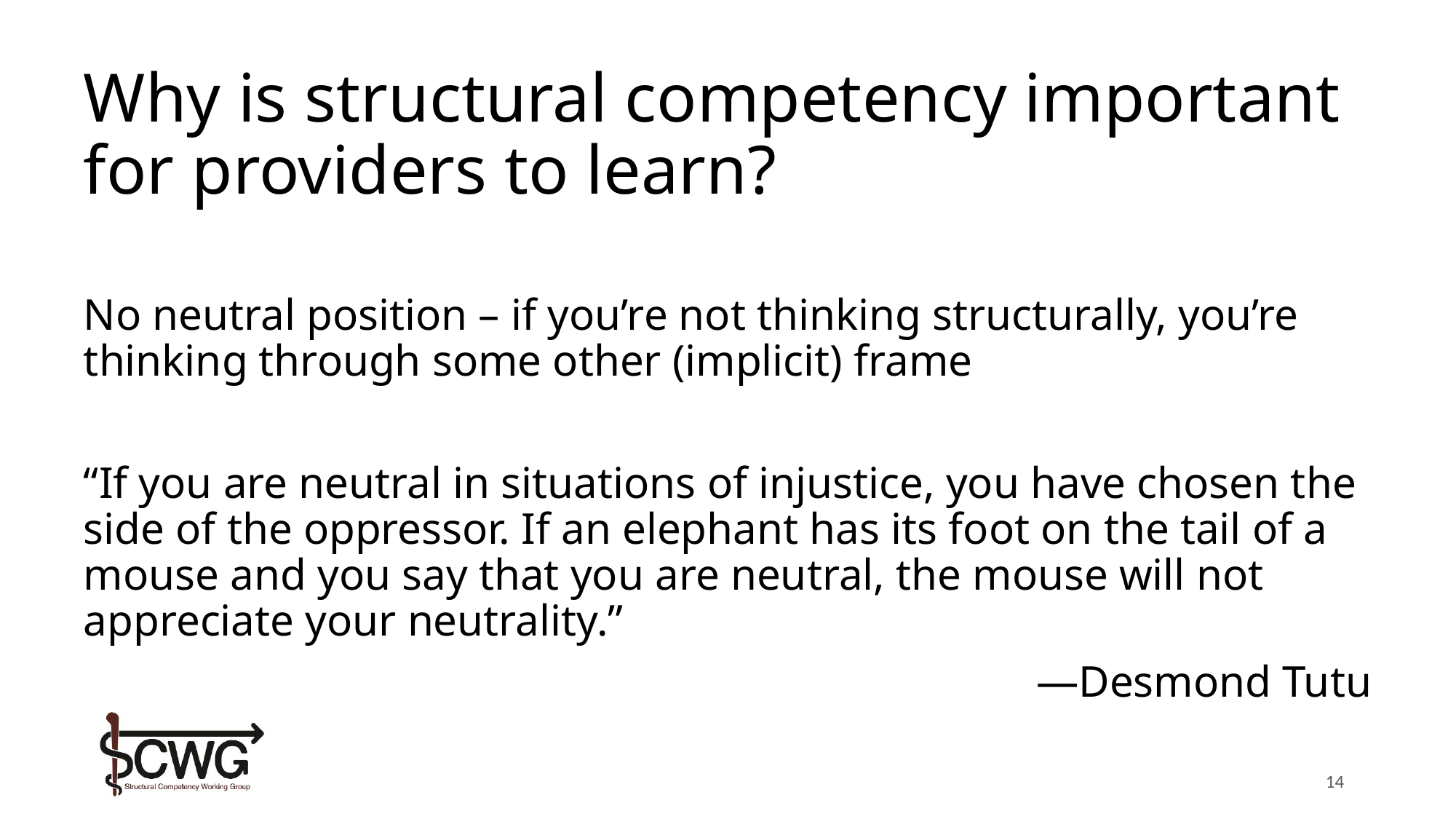

# Why is structural competency important for providers to learn?
No neutral position – if you’re not thinking structurally, you’re thinking through some other (implicit) frame
“If you are neutral in situations of injustice, you have chosen the side of the oppressor. If an elephant has its foot on the tail of a mouse and you say that you are neutral, the mouse will not appreciate your neutrality.”
—Desmond Tutu
14

## Slide 15
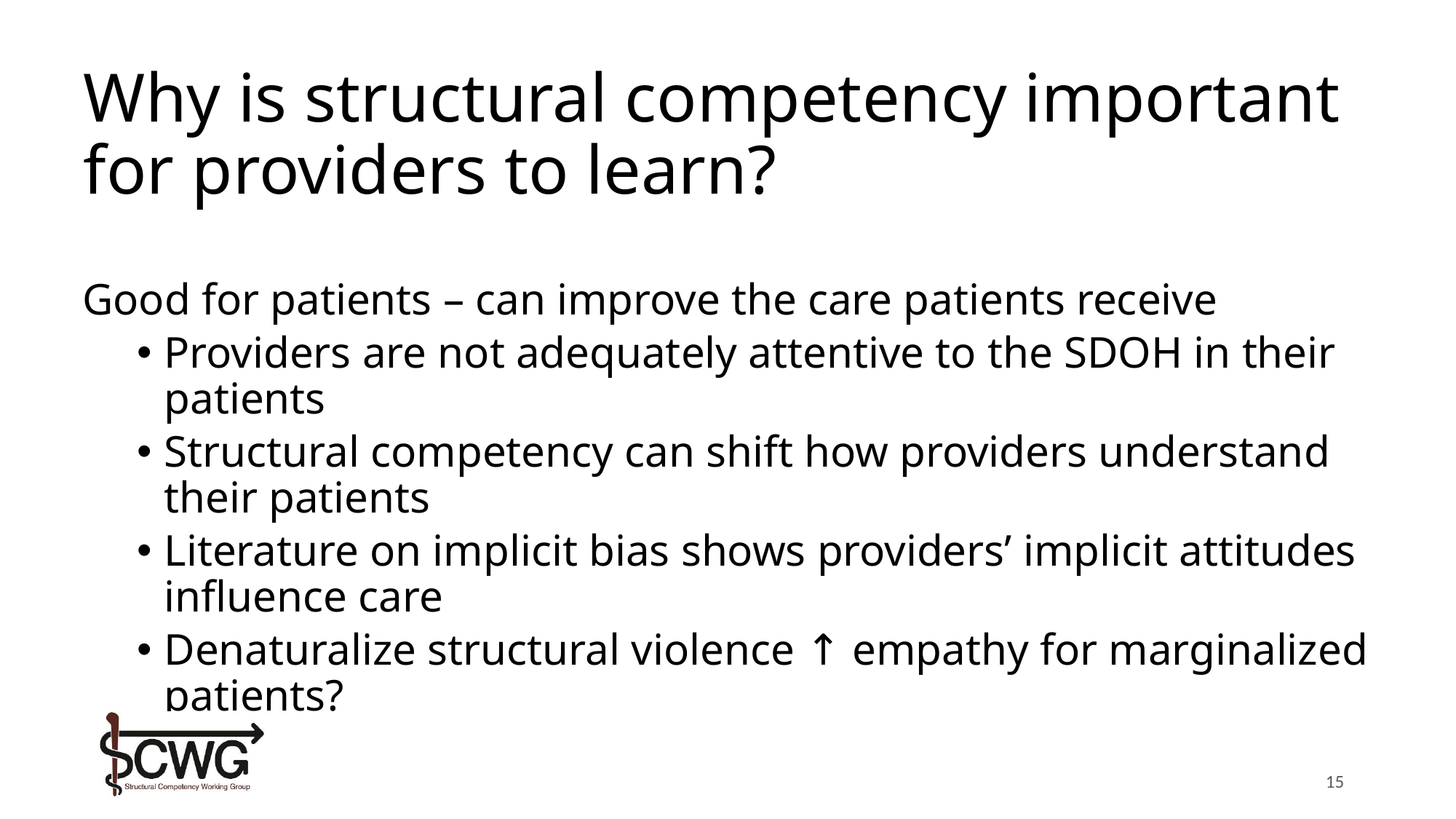

# Why is structural competency important for providers to learn?
Good for patients – can improve the care patients receive
Providers are not adequately attentive to the SDOH in their patients
Structural competency can shift how providers understand their patients
Literature on implicit bias shows providers’ implicit attitudes influence care
Denaturalize structural violence ↑ empathy for marginalized patients?
15

## Slide 16
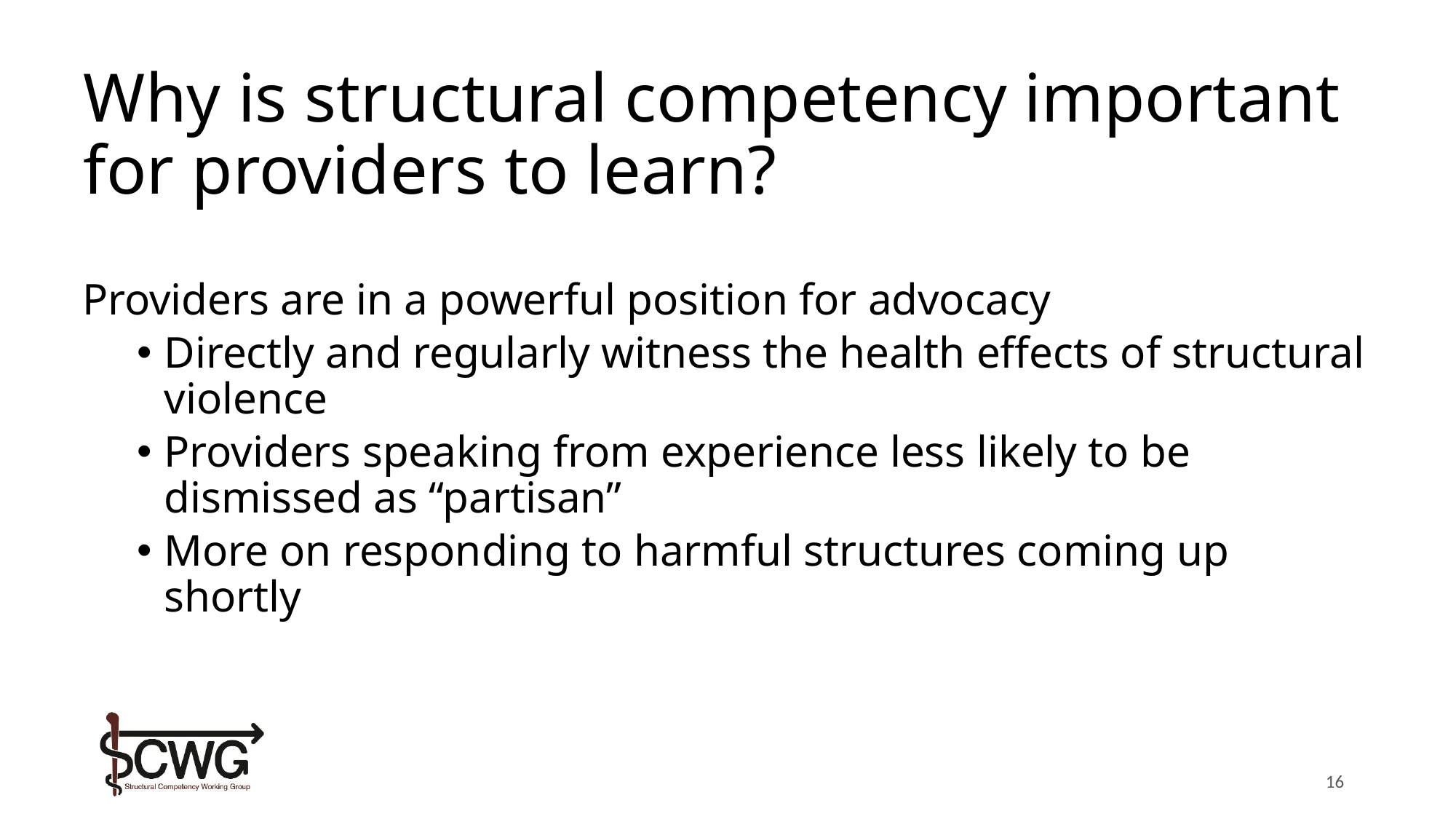

# Why is structural competency important for providers to learn?
Providers are in a powerful position for advocacy
Directly and regularly witness the health effects of structural violence
Providers speaking from experience less likely to be dismissed as “partisan”
More on responding to harmful structures coming up shortly
16

## Slide 17
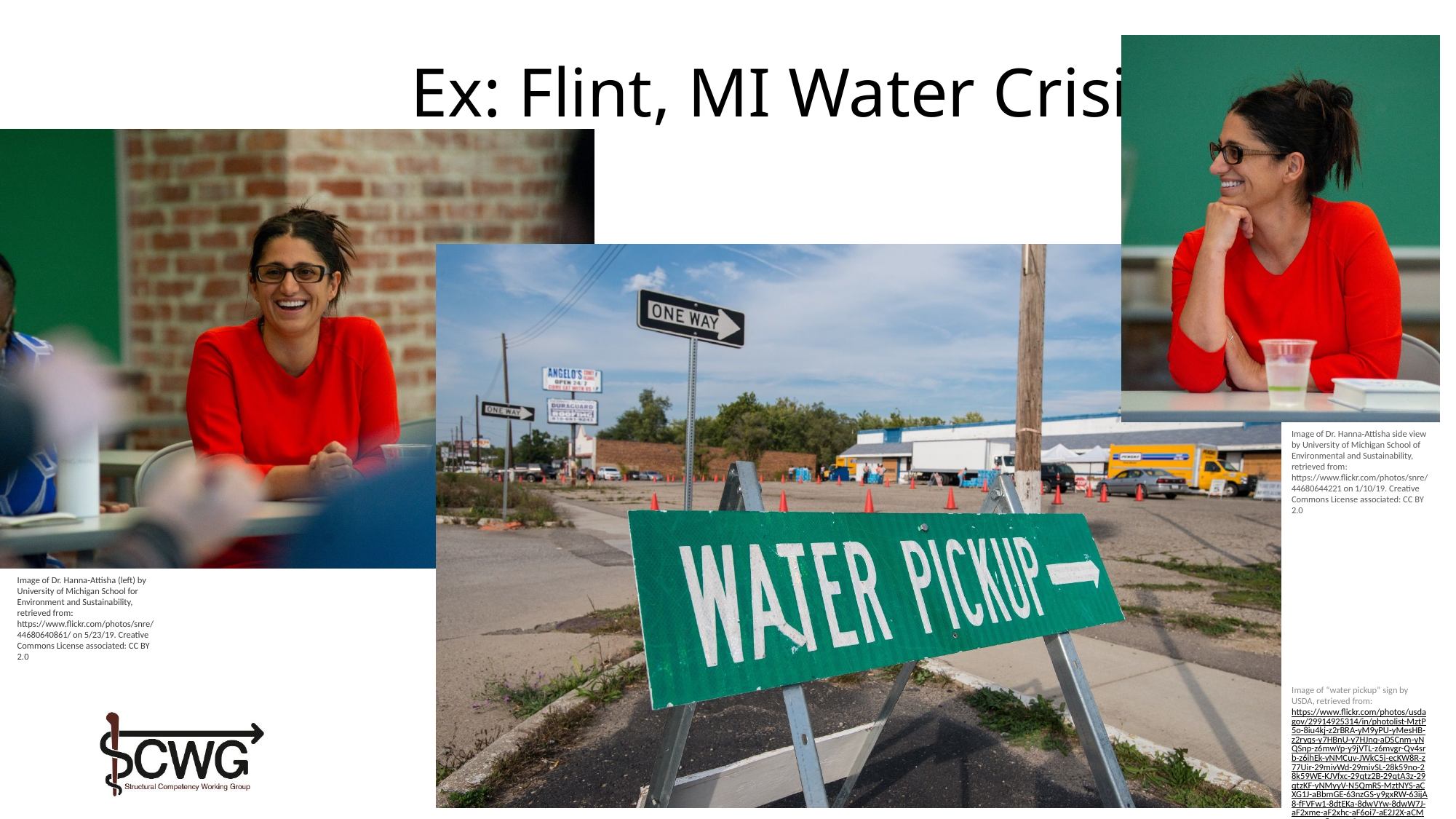

# Ex: Flint, MI Water Crisis
Image of Dr. Hanna-Attisha side view by University of Michigan School of Environmental and Sustainability, retrieved from: https://www.flickr.com/photos/snre/44680644221 on 1/10/19. Creative Commons License associated: CC BY 2.0
Image of Dr. Hanna-Attisha (left) by University of Michigan School for Environment and Sustainability, retrieved from: https://www.flickr.com/photos/snre/44680640861/ on 5/23/19. Creative Commons License associated: CC BY 2.0
Image of “water pickup” sign by USDA, retrieved from: https://www.flickr.com/photos/usdagov/29914925314/in/photolist-MztP5o-8iu4kj-z2rBRA-yM9yPU-yMesHB-z2ryqs-y7HBnU-y7HJnq-aDSCnm-yNQSnp-z6mwYp-y9jVTL-z6mvgr-Qv4srb-z6ihEk-yNMCuv-JWkC5j-ecKW8R-z77Uir-29mivWd-29mivSL-28k59no-28k59WE-KJVfxc-29qtz2B-29qtA3z-29qtzKF-yNMyyV-N5QmRS-MztNYS-aCXG1J-aBbmGE-63nzGS-y9gxRW-63ijA8-fFVFw1-8dtEKa-8dwVYw-8dwW7J-aF2xme-aF2xhc-aF6oi7-aE2J2X-aCMQre-LEzKfb-aD2J8n-La7WRD-La7X4H-La7VCM-dNDPsN on 5/12/19. Image is in the public domain.

## Slide 18
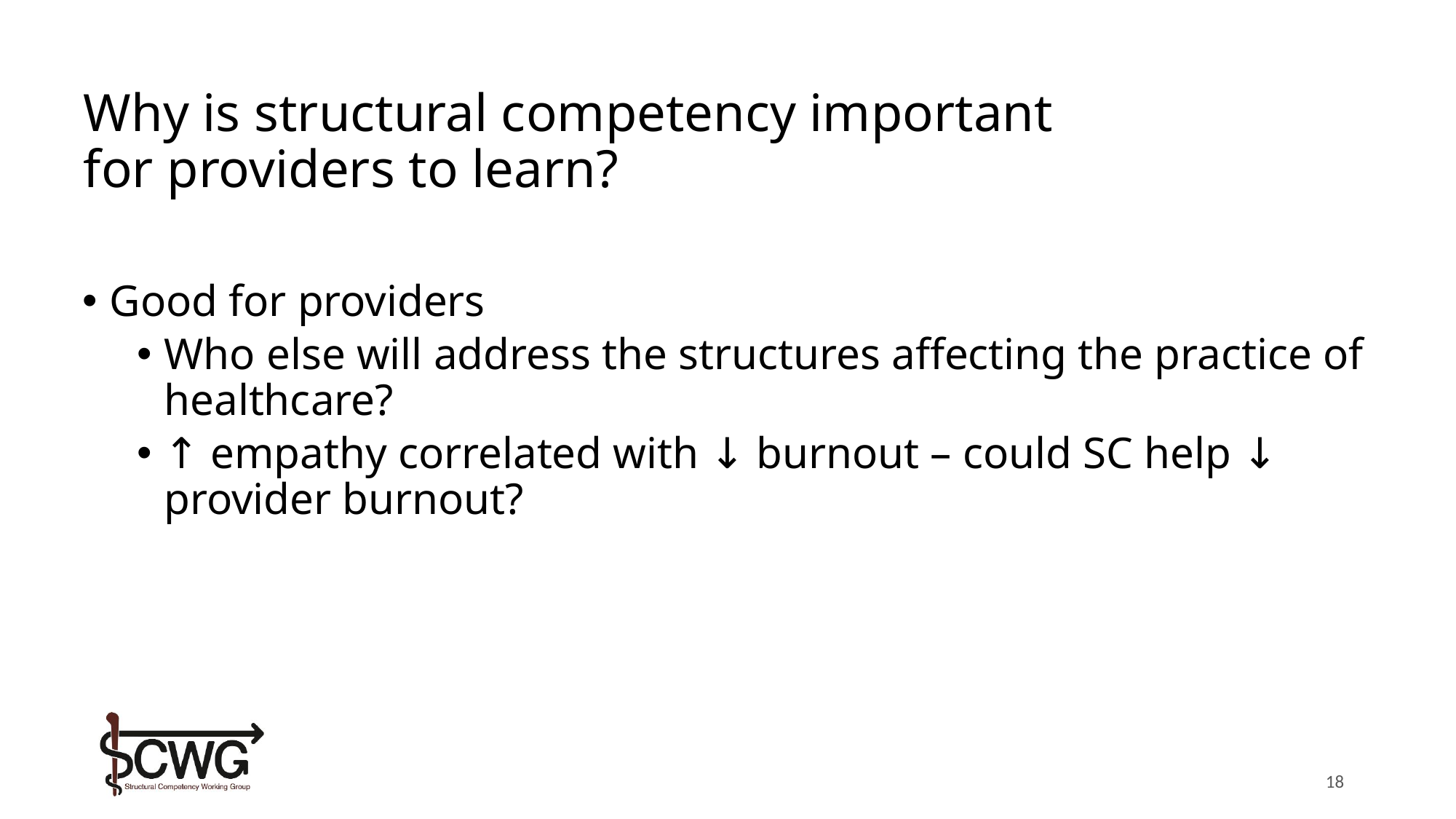

# Why is structural competency important for providers to learn?
Good for providers
Who else will address the structures affecting the practice of healthcare?
↑ empathy correlated with ↓ burnout – could SC help ↓ provider burnout?
18

## Slide 19
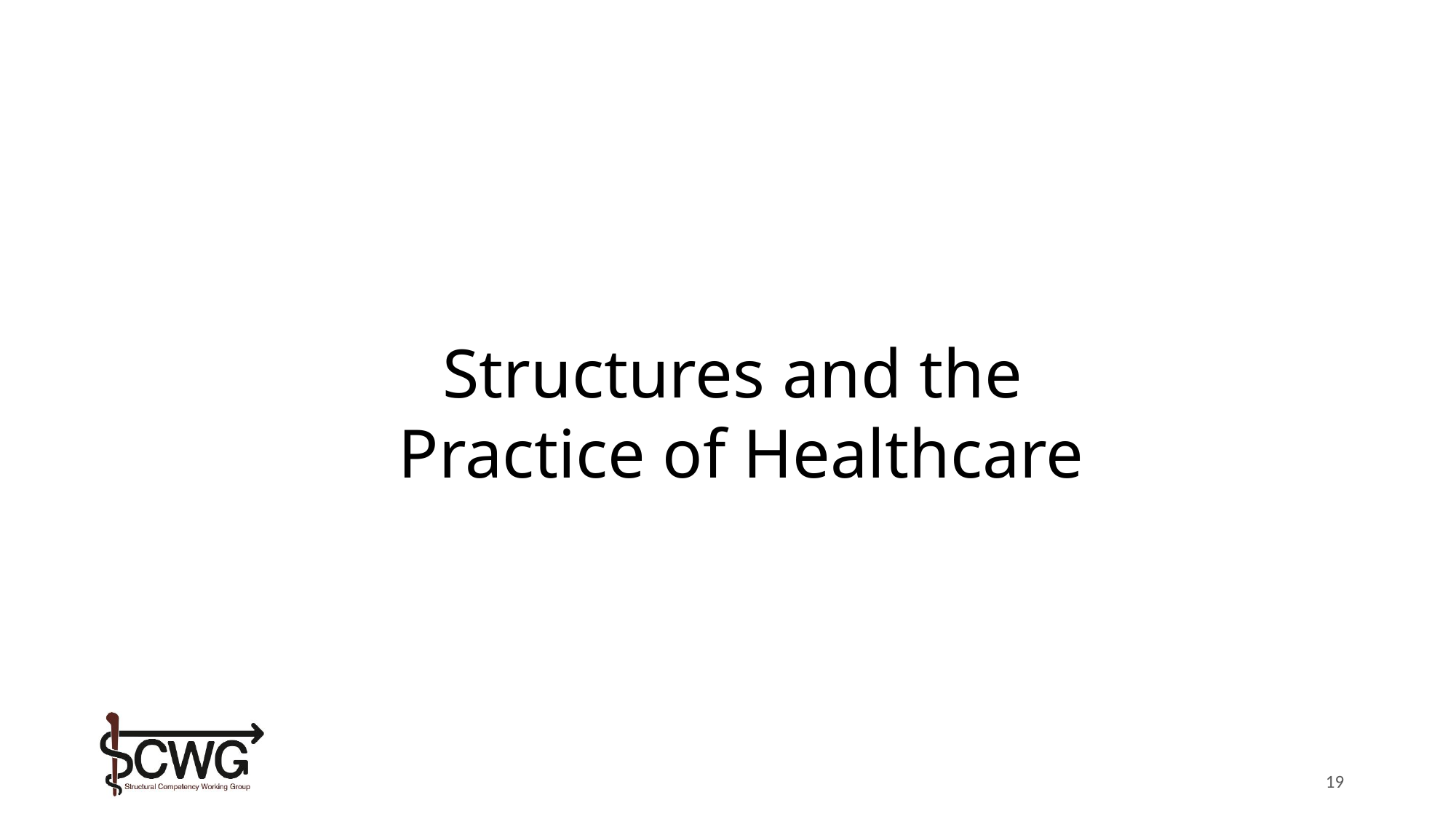

Structures and the
Practice of Healthcare
19

## Slide 20
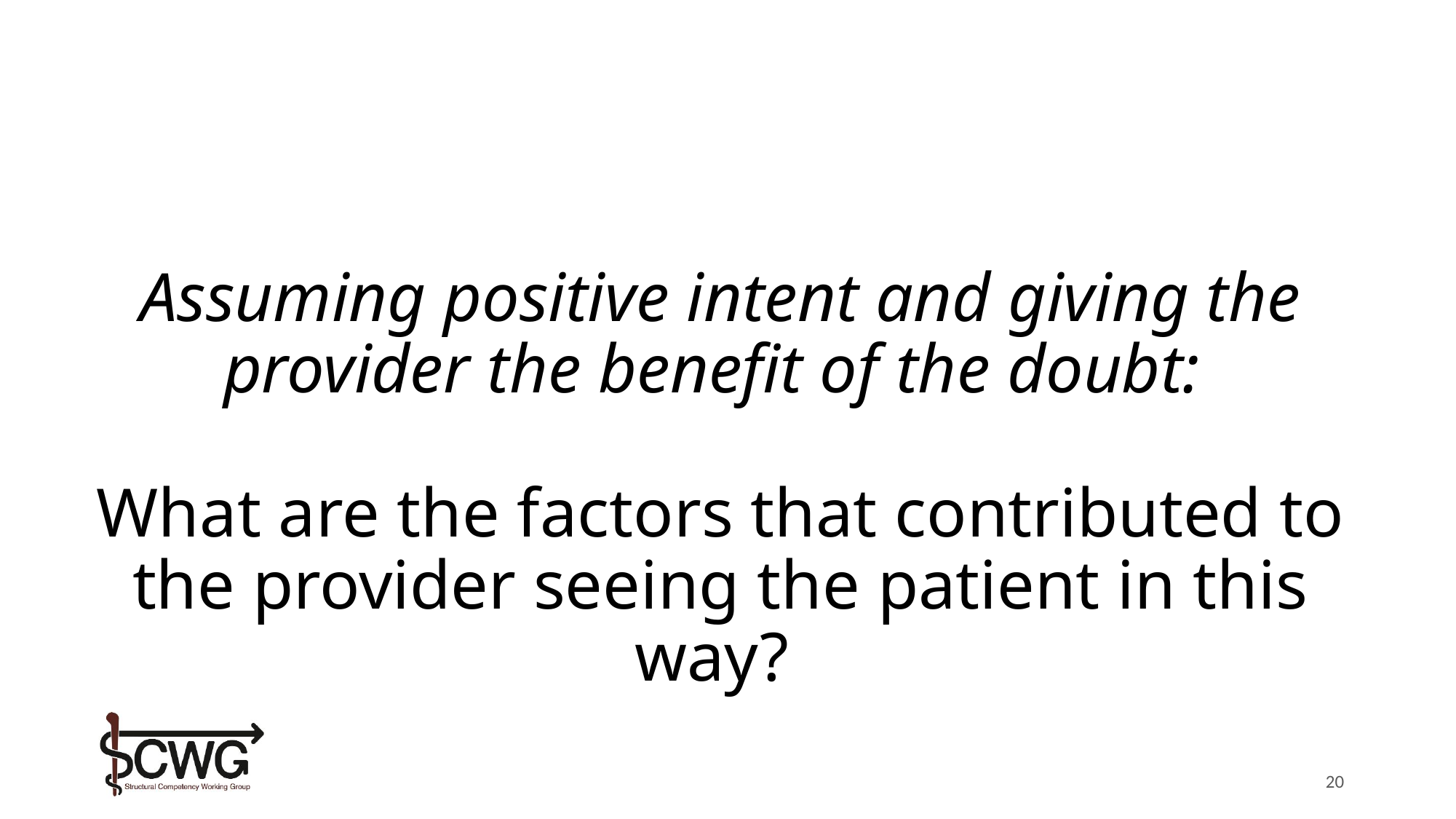

Assuming positive intent and giving the provider the benefit of the doubt:
What are the factors that contributed to the provider seeing the patient in this way?
20

## Slide 21
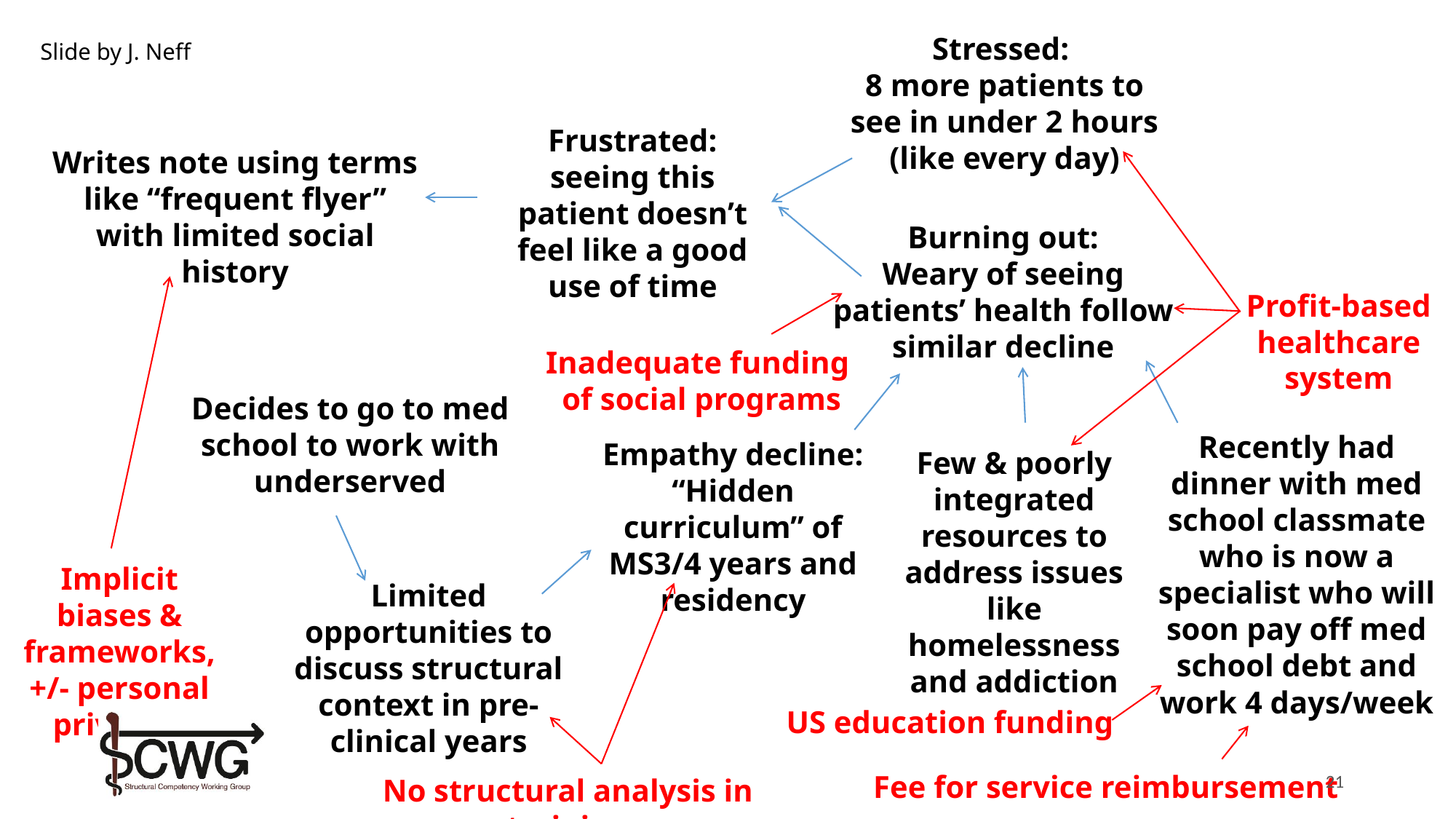

Slide by J. Neff
Stressed:
8 more patients to see in under 2 hours (like every day)
Frustrated: seeing this patient doesn’t feel like a good use of time
Writes note using terms like “frequent flyer” with limited social history
Profit-based healthcare system
Burning out:
Weary of seeing patients’ health follow similar decline
Implicit biases & frameworks, +/- personal privilege
Inadequate funding
of social programs
Recently had dinner with med school classmate who is now a specialist who will soon pay off med school debt and work 4 days/week
Few & poorly integrated resources to address issues like homelessness and addiction
Decides to go to med school to work with underserved
Empathy decline: “Hidden curriculum” of MS3/4 years and residency
Limited opportunities to discuss structural context in pre-clinical years
No structural analysis in training
 US education funding
Fee for service reimbursement
21

## Slide 22
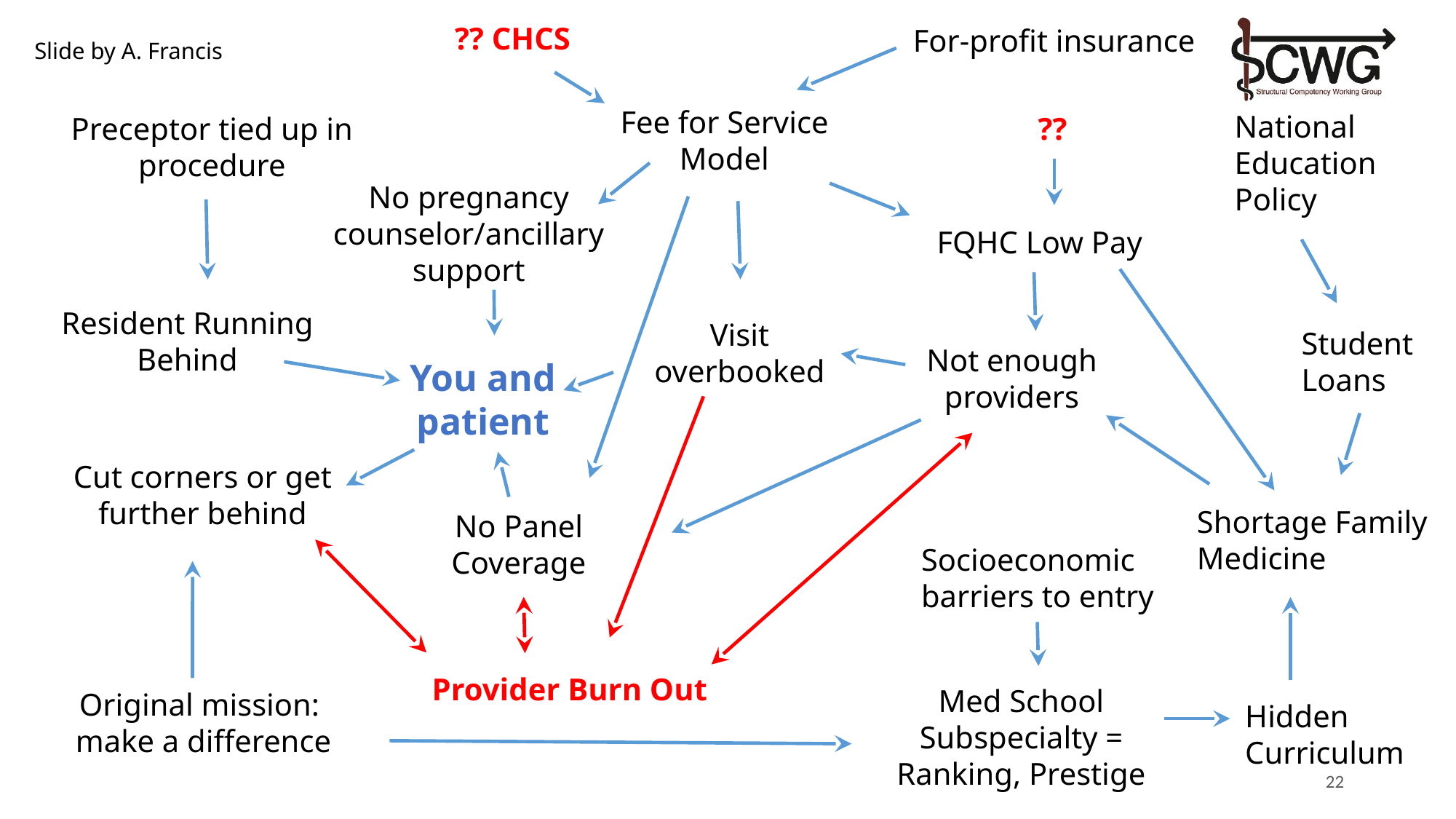

Slide by A. Francis
?? CHCS
For-profit insurance
Fee for Service Model
National Education Policy
Preceptor tied up in procedure
??
No pregnancy counselor/ancillary support
FQHC Low Pay
Shortage Family Medicine
Resident Running Behind
Visit overbooked
Student Loans
Not enough providers
You and patient
Provider Burn Out
Cut corners or get further behind
No Panel Coverage
Socioeconomic barriers to entry
Original mission:
make a difference
Hidden Curriculum
Med School Subspecialty = Ranking, Prestige
22

## Slide 23
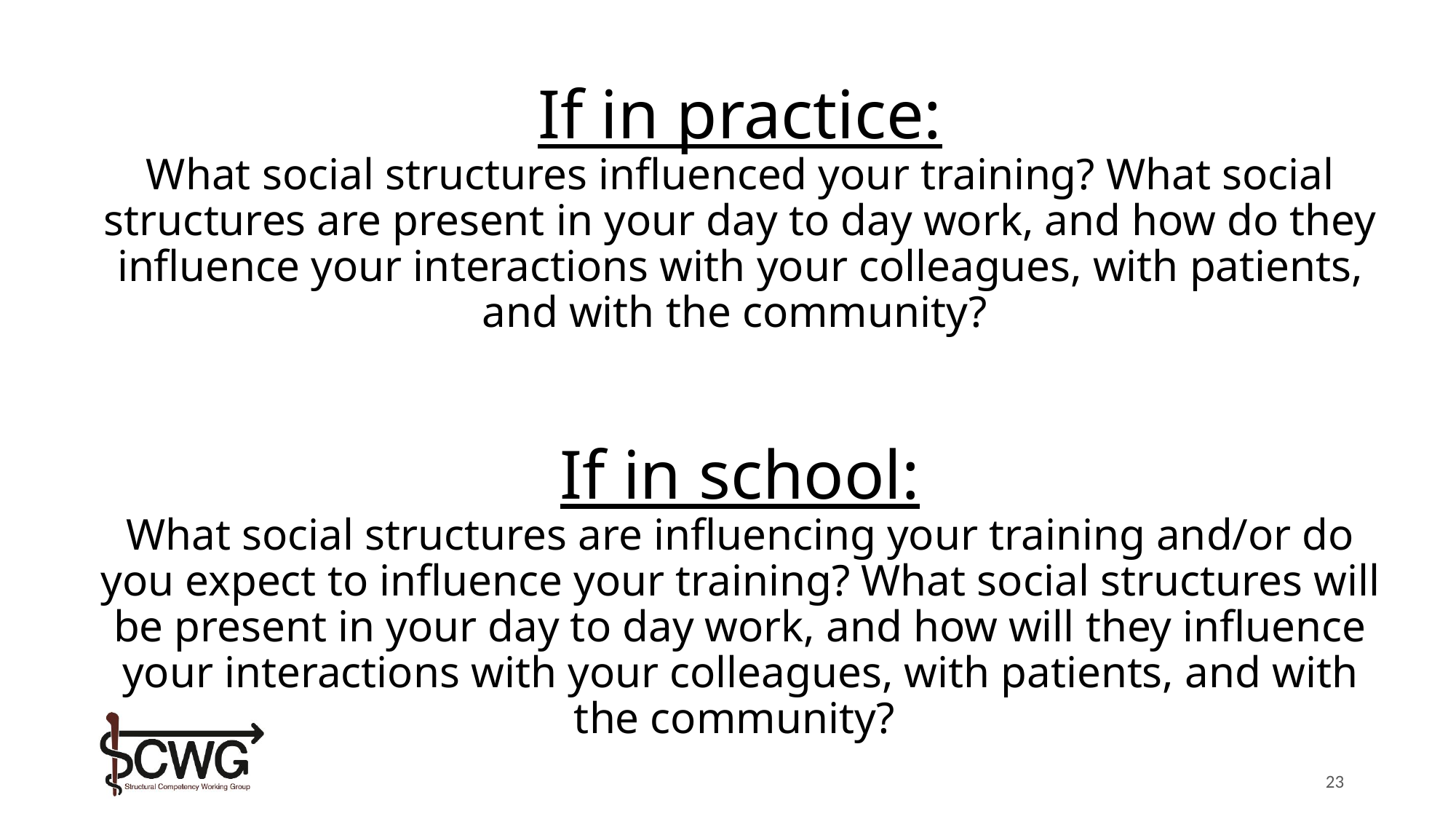

If in practice:
What social structures influenced your training? What social structures are present in your day to day work, and how do they influence your interactions with your colleagues, with patients, and with the community?
If in school:
What social structures are influencing your training and/or do you expect to influence your training? What social structures will be present in your day to day work, and how will they influence your interactions with your colleagues, with patients, and with the community?
23

## Slide 24
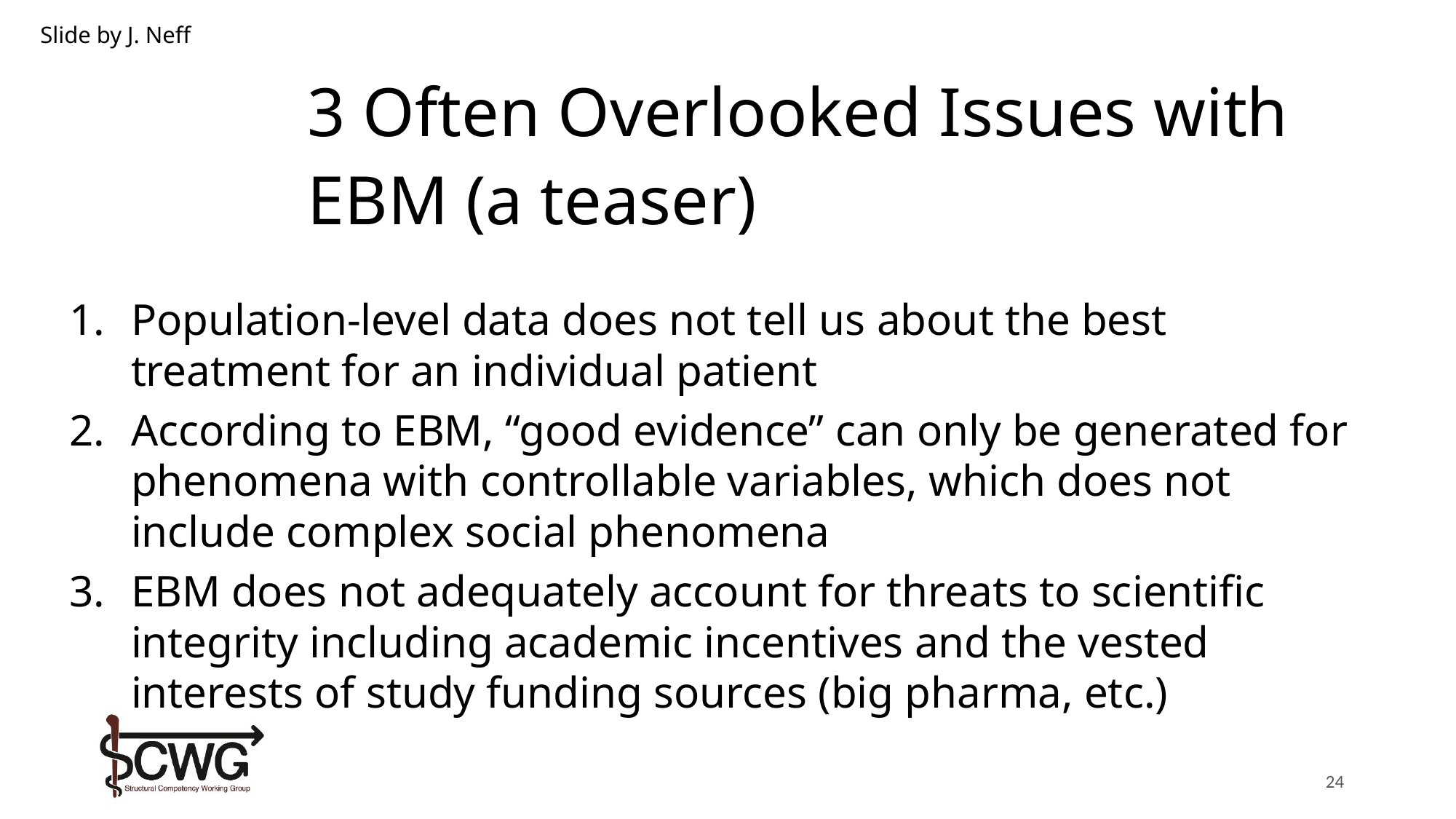

Slide by J. Neff
3 Often Overlooked Issues with EBM (a teaser)
Population-level data does not tell us about the best treatment for an individual patient
According to EBM, “good evidence” can only be generated for phenomena with controllable variables, which does not include complex social phenomena
EBM does not adequately account for threats to scientific integrity including academic incentives and the vested interests of study funding sources (big pharma, etc.)
24

## Slide 25
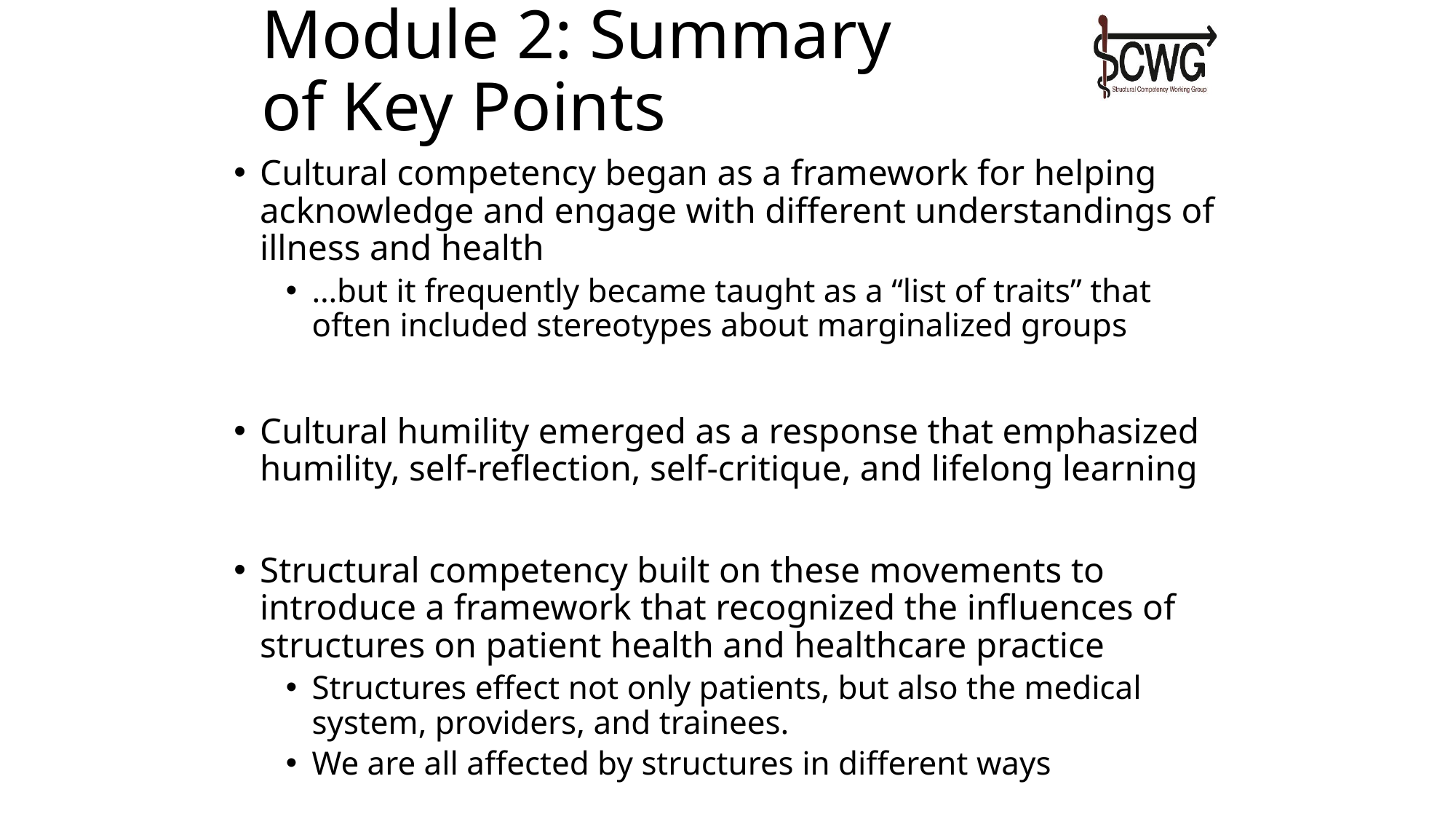

# Module 2: Summary of Key Points
Cultural competency began as a framework for helping acknowledge and engage with different understandings of illness and health
…but it frequently became taught as a “list of traits” that often included stereotypes about marginalized groups
Cultural humility emerged as a response that emphasized humility, self-reflection, self-critique, and lifelong learning
Structural competency built on these movements to introduce a framework that recognized the influences of structures on patient health and healthcare practice
Structures effect not only patients, but also the medical system, providers, and trainees.
We are all affected by structures in different ways
