# Supplementary material for: Structural Competency: Curriculum for Medical Students, Residents, and Interprofessional Teams on the Structural Factors That Produce Health Disparities
Source: MedEdPORTAL. 2020 Mar 13;16:10888. doi: 10.15766/mep_2374-8265.10888 (PMC7182045; doi:10.15766/mep_2374-8265.10888)
Supplement: Supplementary file 1 — A. Manual Background Info.docx B. Manual Intro.docx C. Manual Module 1.docx D. Manual Module 2.docx E. Manual Module 3.docx F. Manual Conclusion and Evaluation.docx G. Supplemental Reading List.docx H. Training Slides Intro.pptx I. Training Slides Module 1.pptx J. Training Slides Module 2.pptx K. Training Slides Module 3.pptx L. Participant Workbook.pdf M. Posttraining Survey.pdf N. Facilitator Guidelines.docx O. Facilitator Preparation - Terms and Concepts.docx P. Participant Sign-in Sheet.docx [file mep-16-10888-s001.zip › K. Training Slides Module 3.pptx]

## Slide 1
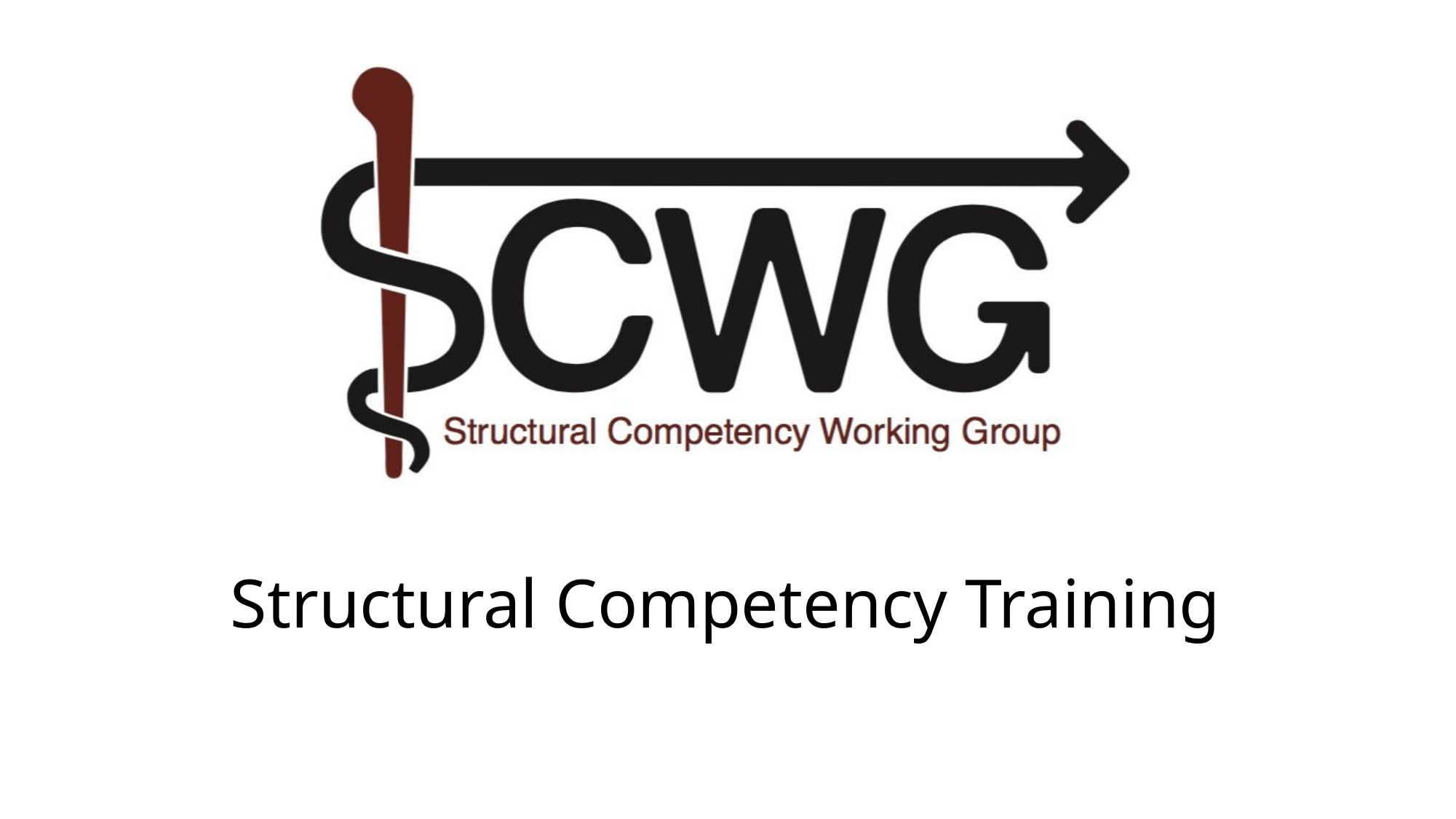

Structural Competency Training

## Slide 2
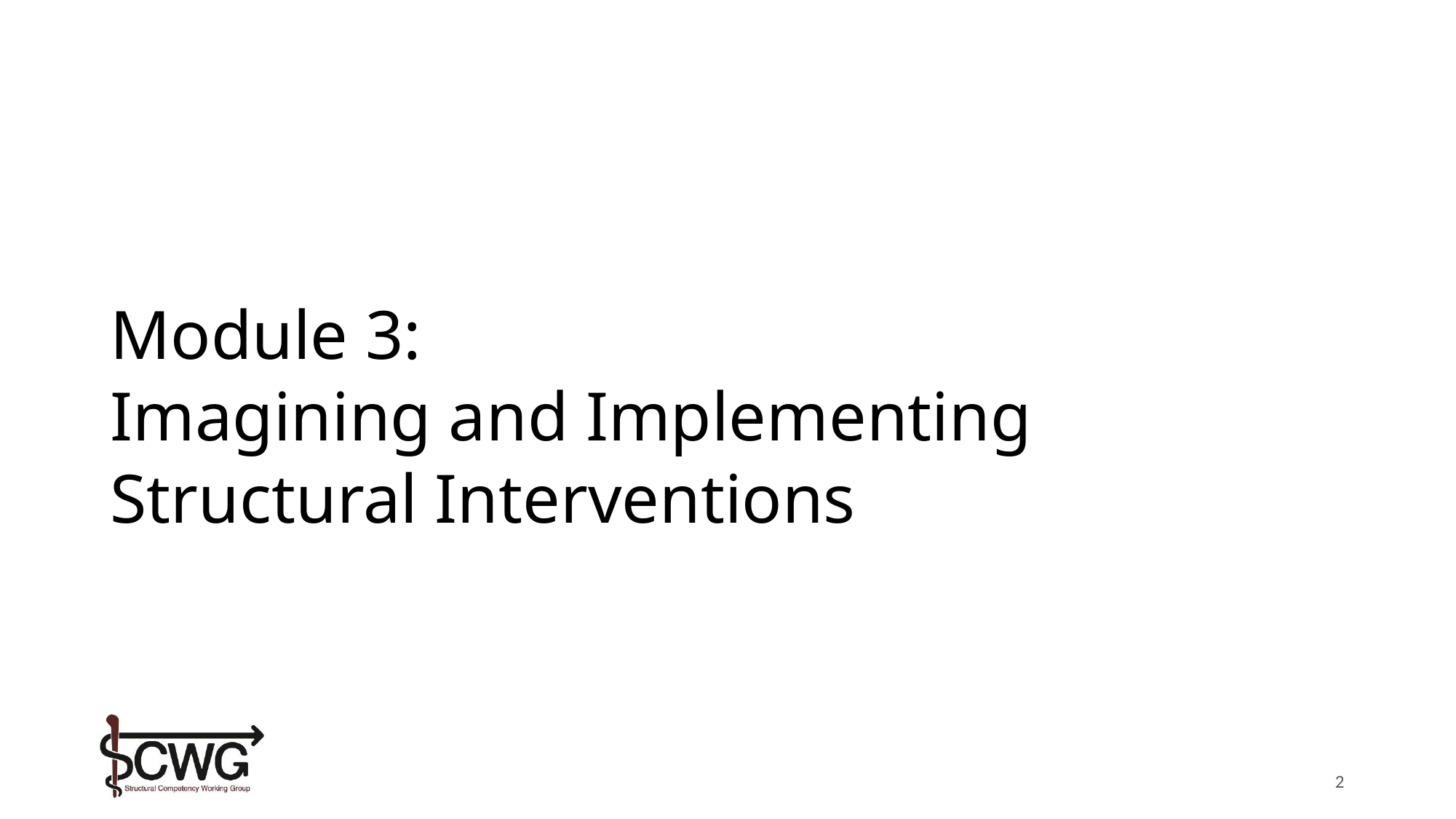

# Module 3: Imagining and Implementing Structural Interventions
2

## Slide 3
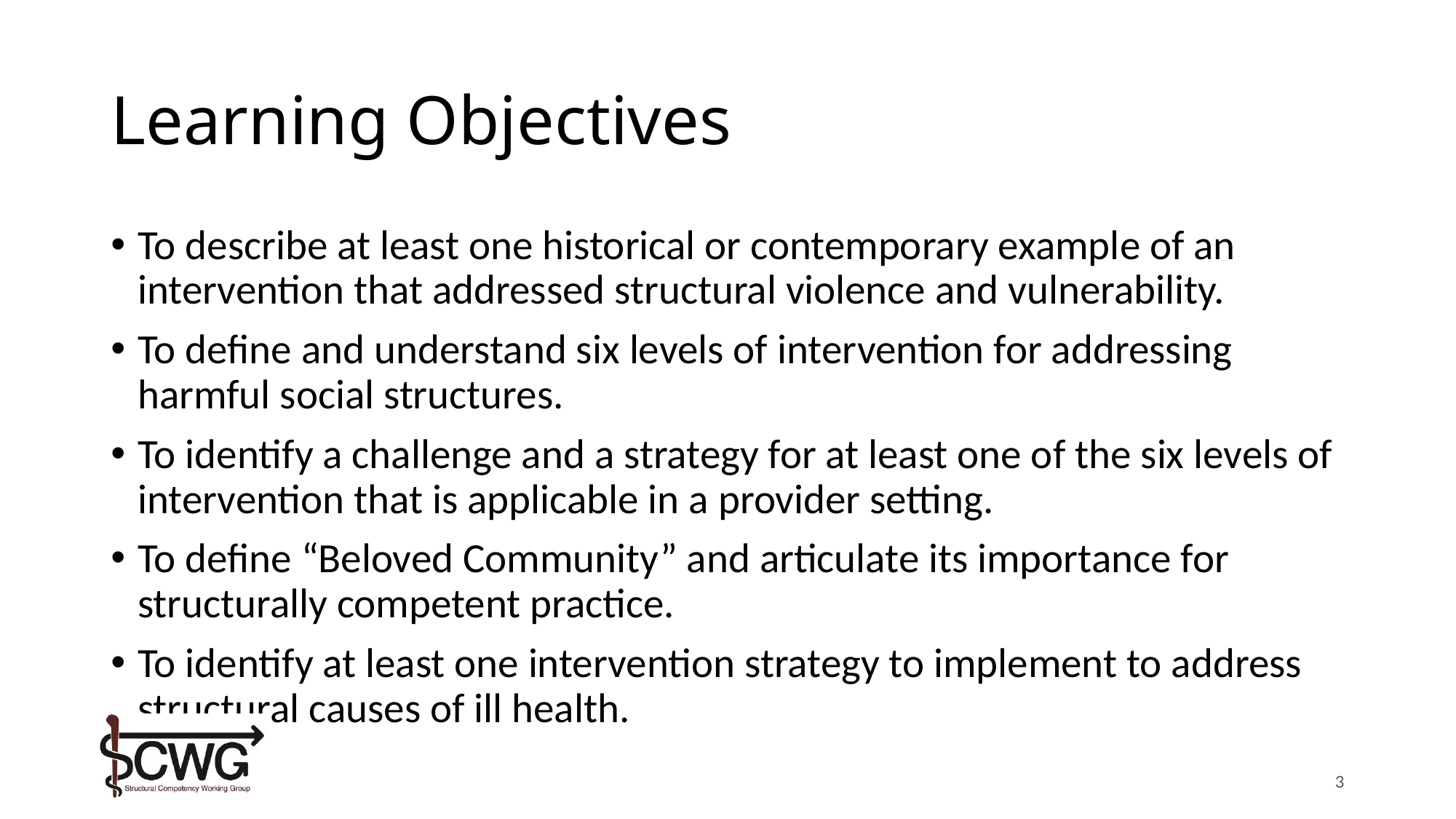

# Learning Objectives
To describe at least one historical or contemporary example of an intervention that addressed structural violence and vulnerability.
To define and understand six levels of intervention for addressing harmful social structures.
To identify a challenge and a strategy for at least one of the six levels of intervention that is applicable in a provider setting.
To define “Beloved Community” and articulate its importance for structurally competent practice.
To identify at least one intervention strategy to implement to address structural causes of ill health.
3

## Slide 4
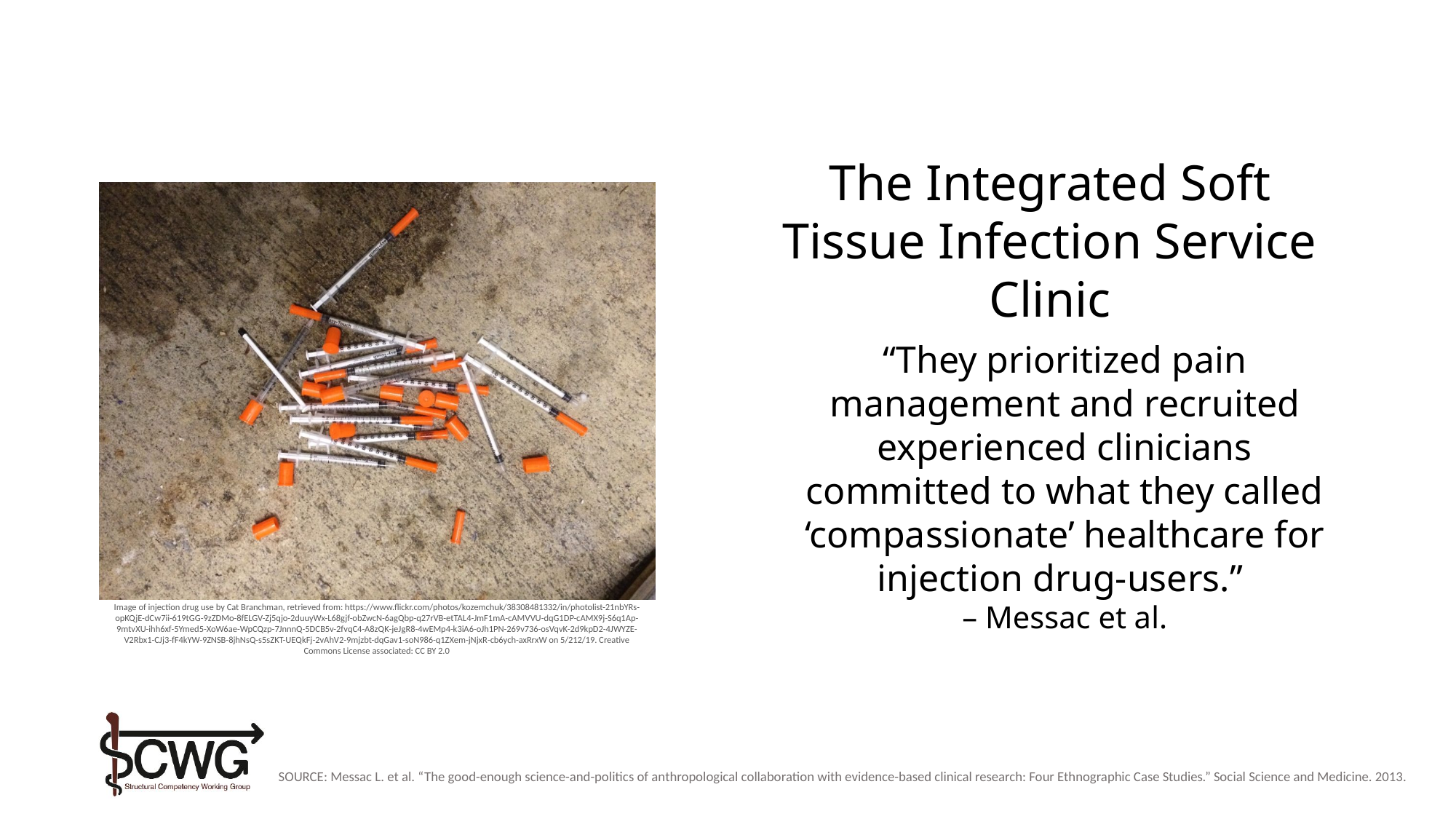

The Integrated Soft Tissue Infection Service Clinic
“They prioritized pain management and recruited experienced clinicians committed to what they called ‘compassionate’ healthcare for injection drug-users.”
– Messac et al.
Image of injection drug use by Cat Branchman, retrieved from: https://www.flickr.com/photos/kozemchuk/38308481332/in/photolist-21nbYRs-opKQjE-dCw7ii-619tGG-9zZDMo-8fELGV-Zj5qjo-2duuyWx-L68gjf-obZwcN-6agQbp-q27rVB-etTAL4-JmF1mA-cAMVVU-dqG1DP-cAMX9j-S6q1Ap-9mtvXU-ihh6xf-5Ymed5-XoW6ae-WpCQzp-7JnnnQ-5DCB5v-2fvqC4-A8zQK-jeJgR8-4wEMp4-k3iA6-oJh1PN-269v736-osVqvK-2d9kpD2-4JWYZE-V2Rbx1-CJj3-fF4kYW-9ZNSB-8jhNsQ-s5sZKT-UEQkFj-2vAhV2-9mjzbt-dqGav1-soN986-q1ZXem-jNjxR-cb6ych-axRrxW on 5/212/19. Creative Commons License associated: CC BY 2.0
SOURCE: Messac L. et al. “The good-enough science-and-politics of anthropological collaboration with evidence-based clinical research: Four Ethnographic Case Studies.” Social Science and Medicine. 2013.

## Slide 5
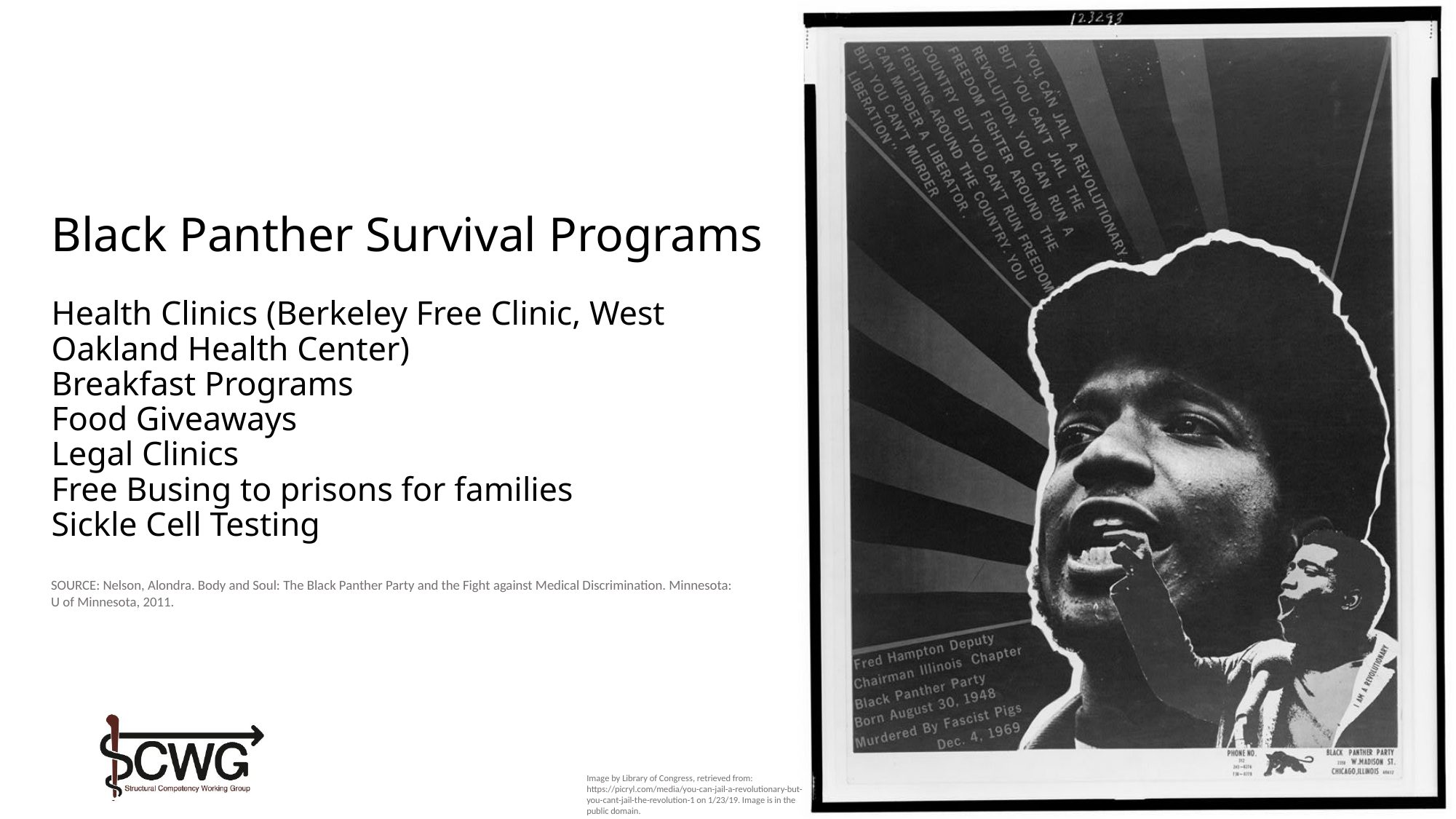

# Black Panther Survival Programs Health Clinics (Berkeley Free Clinic, West Oakland Health Center) Breakfast Programs Food Giveaways Legal Clinics Free Busing to prisons for families Sickle Cell Testing
SOURCE: Nelson, Alondra. Body and Soul: The Black Panther Party and the Fight against Medical Discrimination. Minnesota: U of Minnesota, 2011.
Image by Library of Congress, retrieved from: https://picryl.com/media/you-can-jail-a-revolutionary-but-you-cant-jail-the-revolution-1 on 1/23/19. Image is in the public domain.

## Slide 6
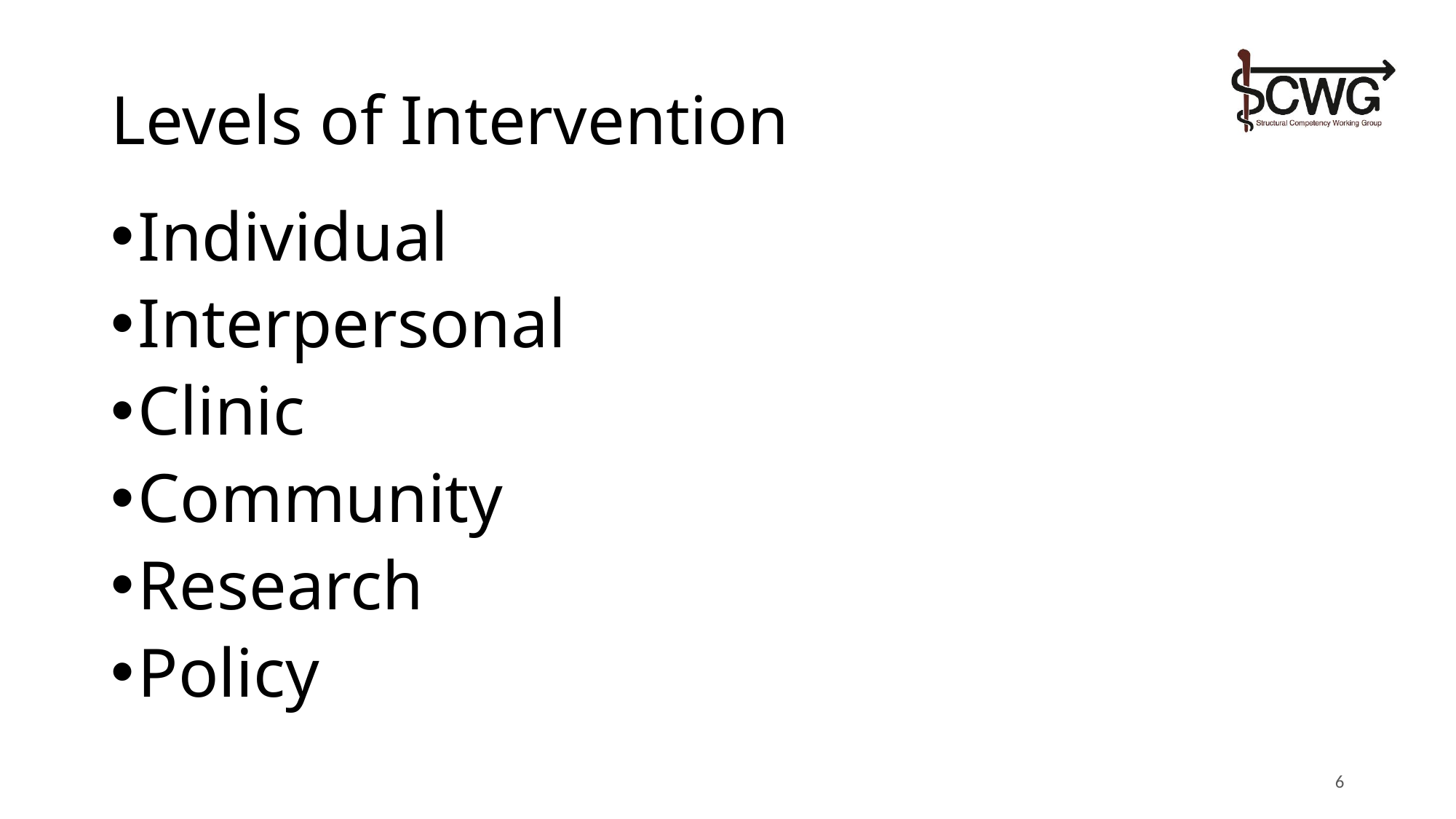

# Levels of Intervention
Individual
Interpersonal
Clinic
Community
Research
Policy
6

## Slide 7
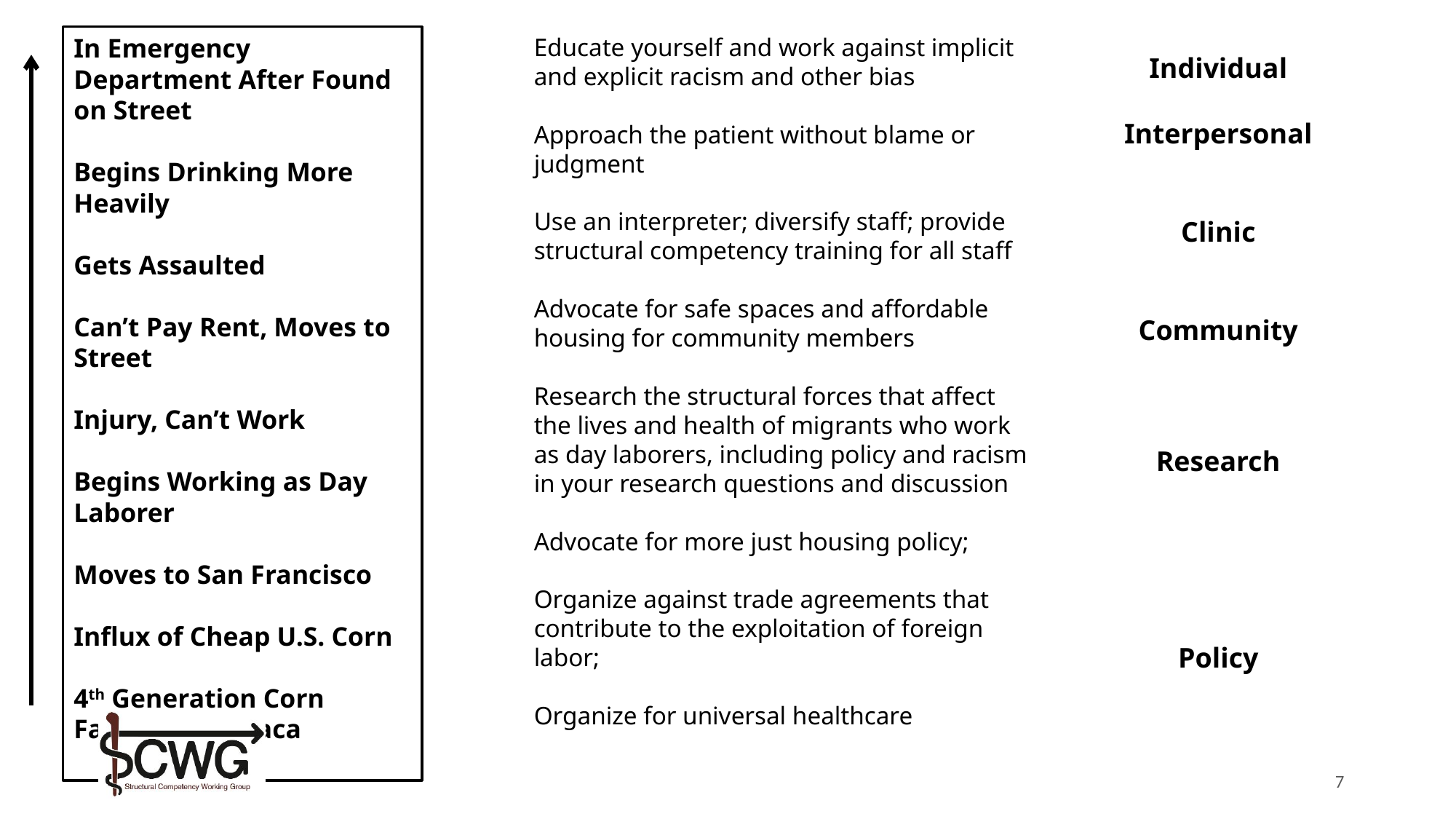

Educate yourself and work against implicit and explicit racism and other bias
Approach the patient without blame or judgment
Use an interpreter; diversify staff; provide structural competency training for all staff
Advocate for safe spaces and affordable housing for community members
Research the structural forces that affect the lives and health of migrants who work as day laborers, including policy and racism in your research questions and discussion
Advocate for more just housing policy;
Organize against trade agreements that contribute to the exploitation of foreign labor;
Organize for universal healthcare
In Emergency Department After Found on Street
Begins Drinking More Heavily
Gets Assaulted
Can’t Pay Rent, Moves to Street
Injury, Can’t Work
Begins Working as Day Laborer
Moves to San Francisco
Influx of Cheap U.S. Corn
4th Generation Corn Farmer in Oaxaca
Individual
Interpersonal
Clinic
Community
Research
Policy
7

## Slide 8
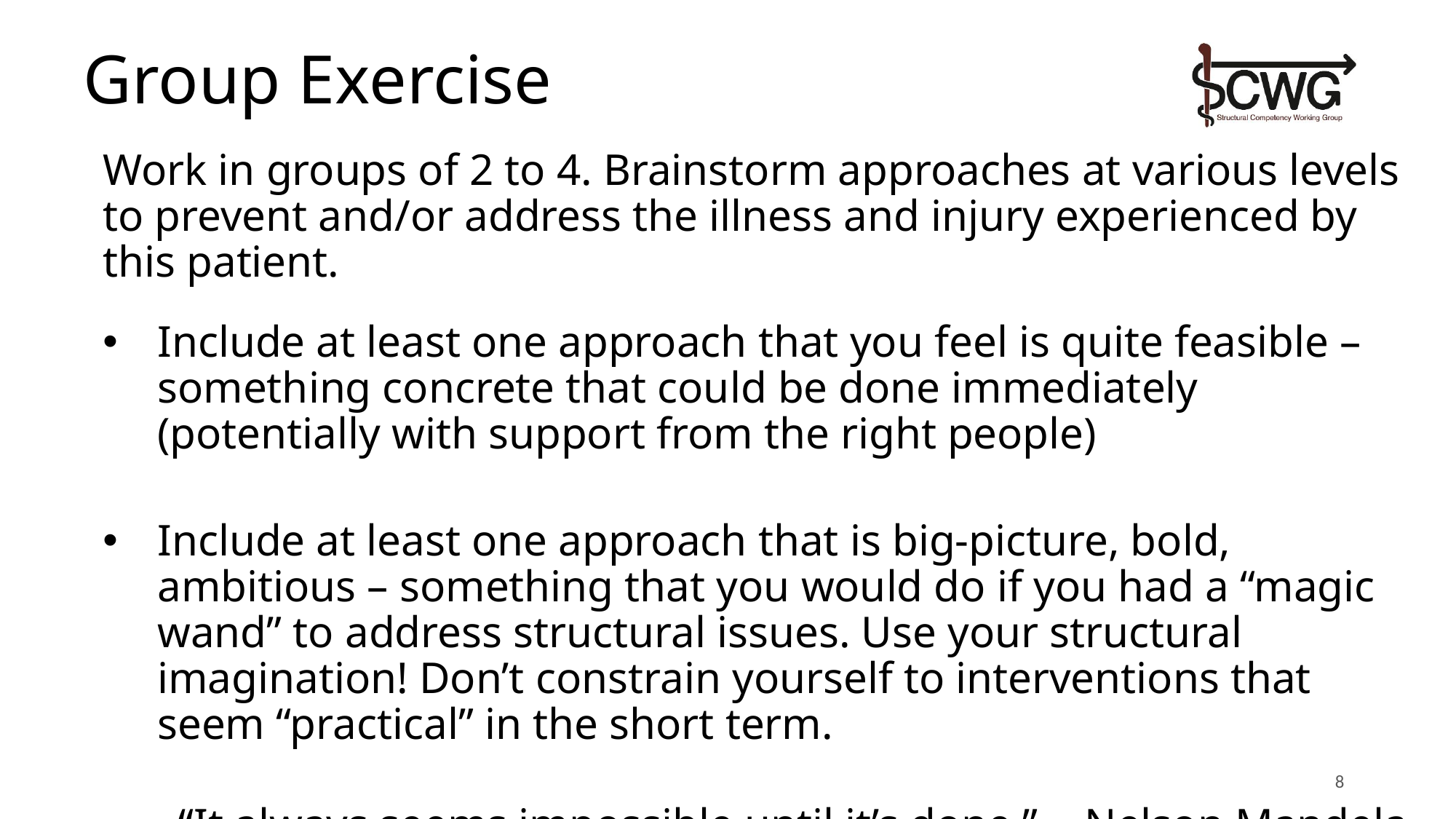

# Group Exercise
Work in groups of 2 to 4. Brainstorm approaches at various levels to prevent and/or address the illness and injury experienced by this patient.
Include at least one approach that you feel is quite feasible – something concrete that could be done immediately (potentially with support from the right people)
Include at least one approach that is big-picture, bold, ambitious – something that you would do if you had a “magic wand” to address structural issues. Use your structural imagination! Don’t constrain yourself to interventions that seem “practical” in the short term.
“It always seems impossible until it’s done.”   -Nelson Mandela
8

## Slide 9
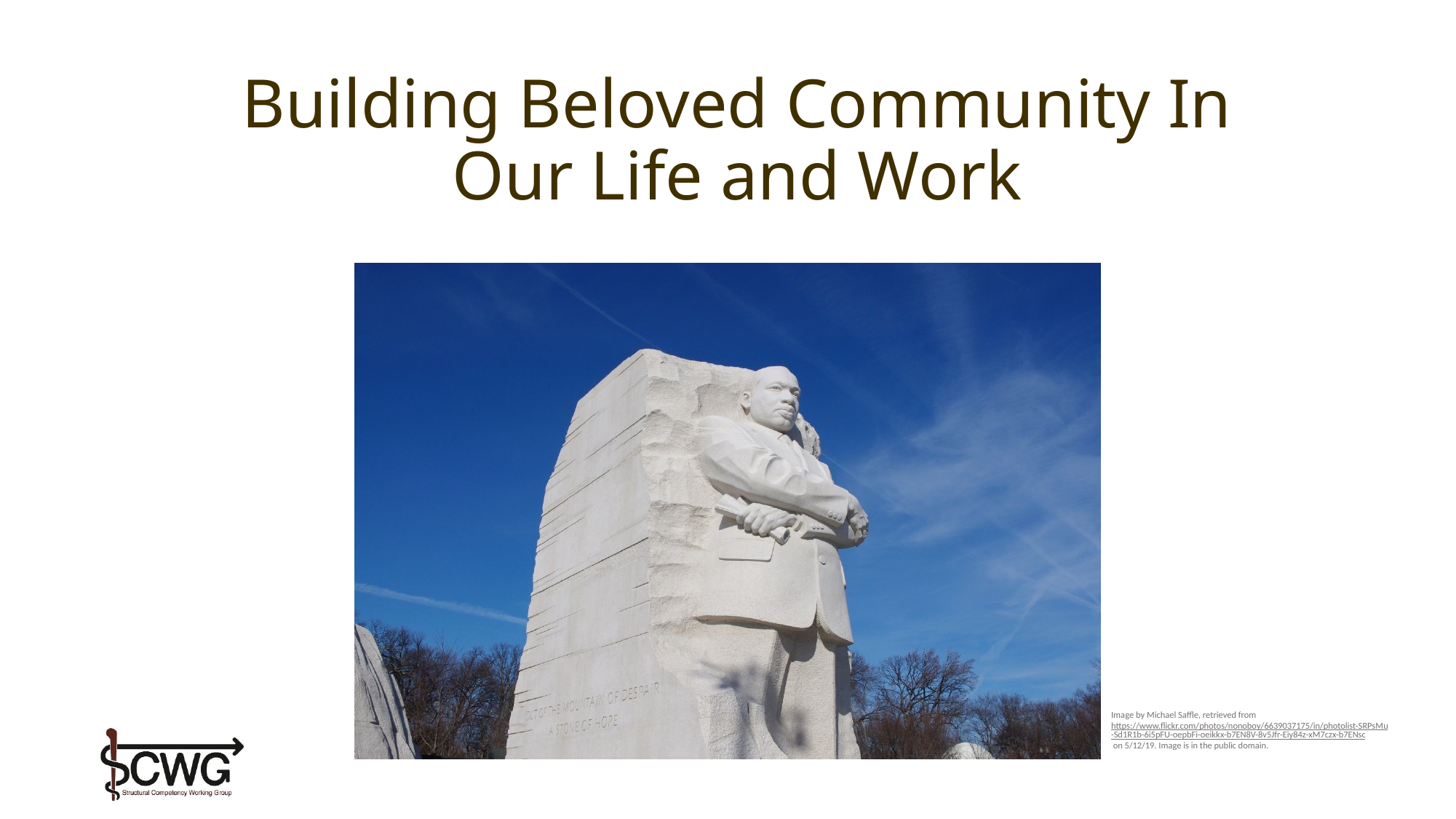

# Building Beloved Community In Our Life and Work
Image by Michael Saffle, retrieved from https://www.flickr.com/photos/nonoboy/6639037175/in/photolist-SRPsMu-Sd1R1b-6i5pFU-oepbFi-oeikkx-b7EN8V-8v5Jfr-Eiy84z-xM7czx-b7ENsc on 5/12/19. Image is in the public domain.

## Slide 10
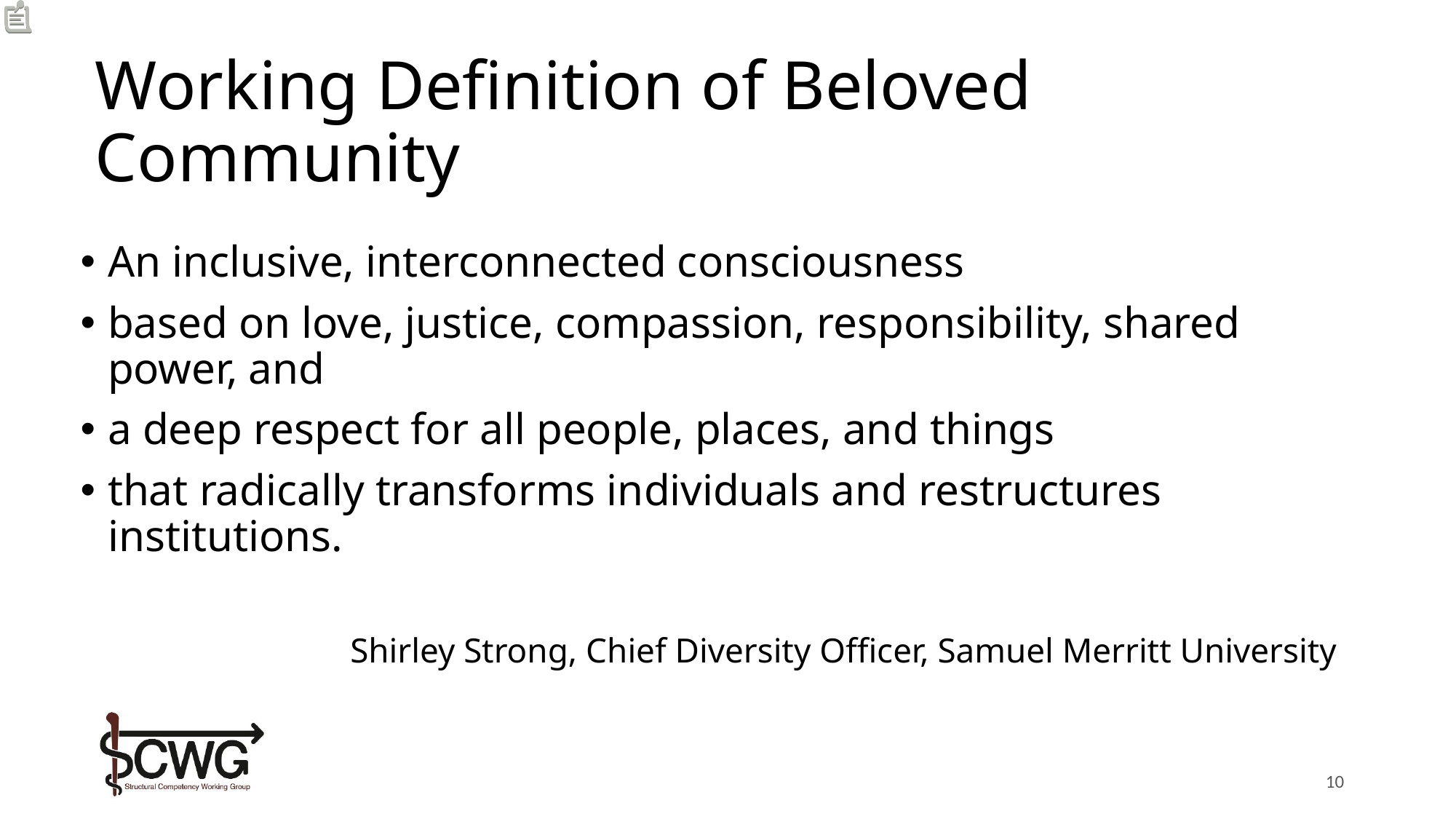

Working Definition of Beloved Community
An inclusive, interconnected consciousness
based on love, justice, compassion, responsibility, shared power, and
a deep respect for all people, places, and things
that radically transforms individuals and restructures institutions.
Shirley Strong, Chief Diversity Officer, Samuel Merritt University
10

## Slide 11
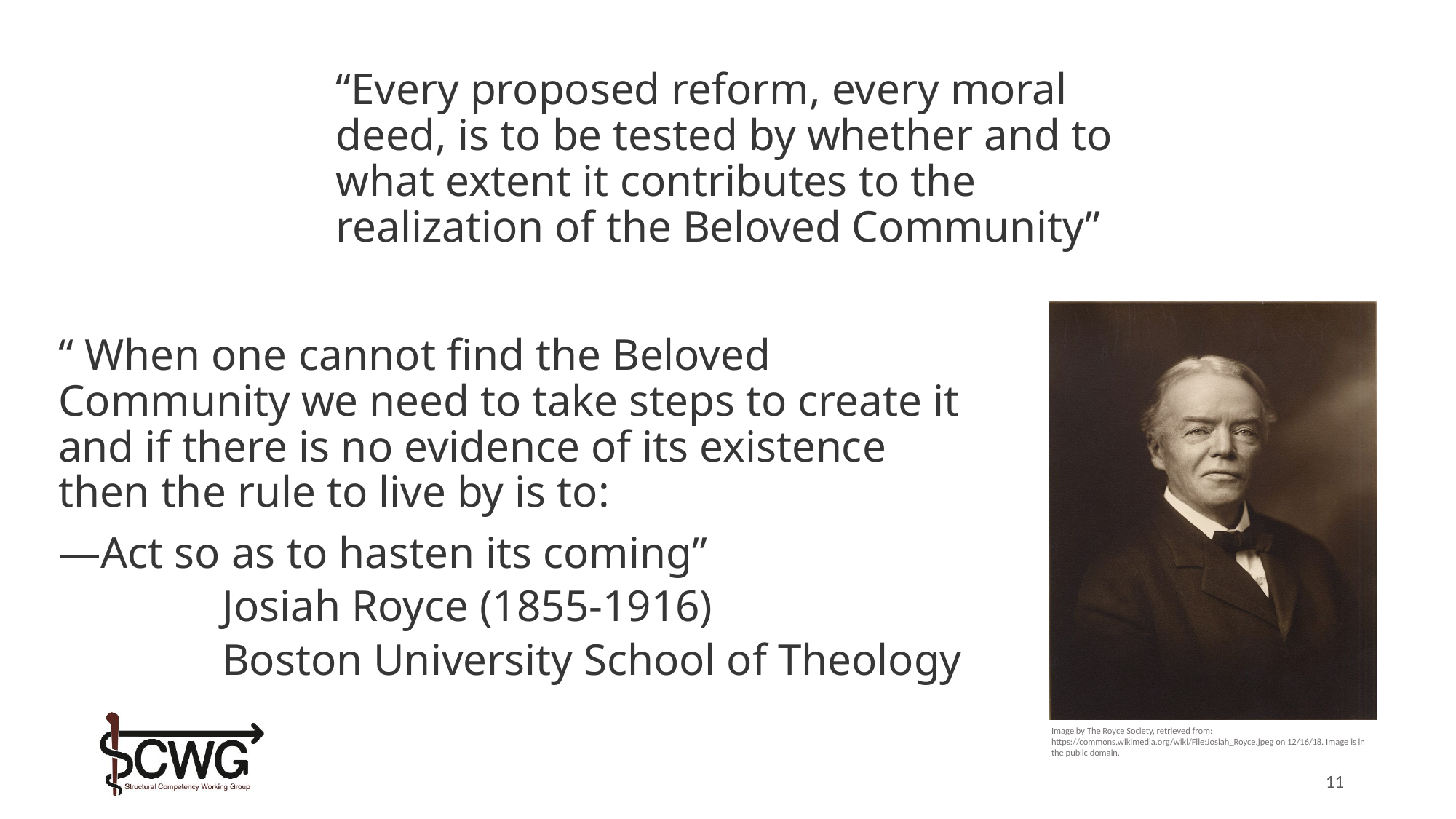

“Every proposed reform, every moral deed, is to be tested by whether and to what extent it contributes to the realization of the Beloved Community”
“ When one cannot find the Beloved Community we need to take steps to create it and if there is no evidence of its existence then the rule to live by is to:
—Act so as to hasten its coming”
Josiah Royce (1855-1916)
Boston University School of Theology
Image by The Royce Society, retrieved from: https://commons.wikimedia.org/wiki/File:Josiah_Royce.jpeg on 12/16/18. Image is in the public domain.
11

## Slide 12
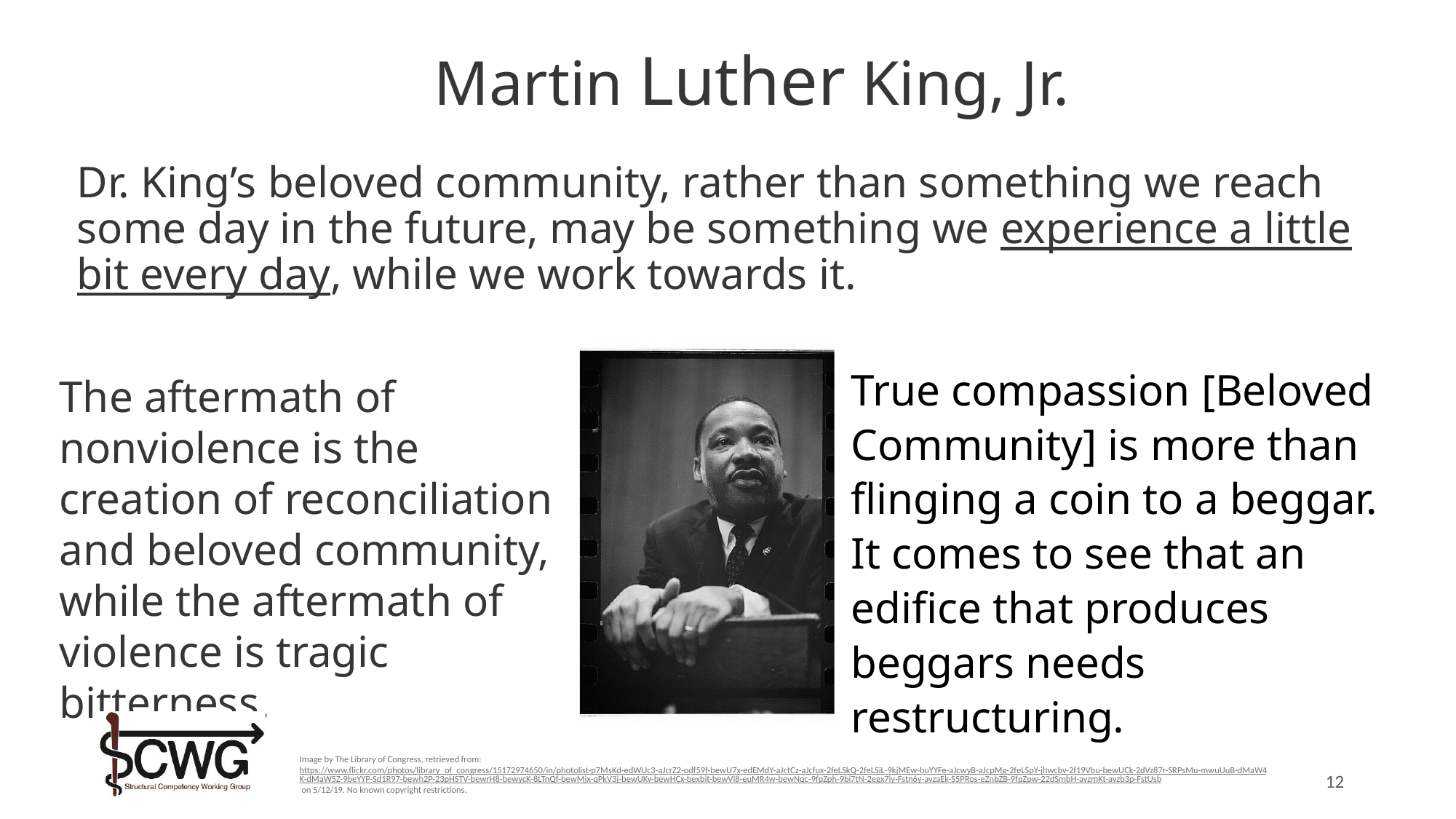

Martin Luther King, Jr.
Dr. King’s beloved community, rather than something we reach some day in the future, may be something we experience a little bit every day, while we work towards it.
True compassion [Beloved Community] is more than flinging a coin to a beggar. It comes to see that an edifice that produces beggars needs restructuring.
The aftermath of nonviolence is the creation of reconciliation and beloved community, while the aftermath of violence is tragic bitterness.
Image by The Library of Congress, retrieved from: https://www.flickr.com/photos/library_of_congress/15172974650/in/photolist-p7MsKd-edWUc3-aJcrZ2-odf59f-bewU7x-edEMdY-aJctCz-aJcfux-2feLSkQ-2feLSiL-9kjMEw-buYYFe-aJcwyB-aJcpMg-2feLSpY-jhwcbv-2f19Vbu-bewUCk-2dVz87r-SRPsMu-mwuUuB-dMaW4K-dMaW5Z-9beYYP-Sd1R97-bewh2P-23pHSTV-bewrH8-bewycK-8LTnQf-bewMjx-qPkV3j-bewUKv-bewHCx-bexbit-bewVi8-euMR4w-bewNqc-9fpZph-9bi7tN-2egx7iy-Fstn6y-avzaEk-S5PRos-eZnbZB-9fpZpw-22dSmbH-avzmKt-avzb3p-FstUsb on 5/12/19. No known copyright restrictions.
12

## Slide 13
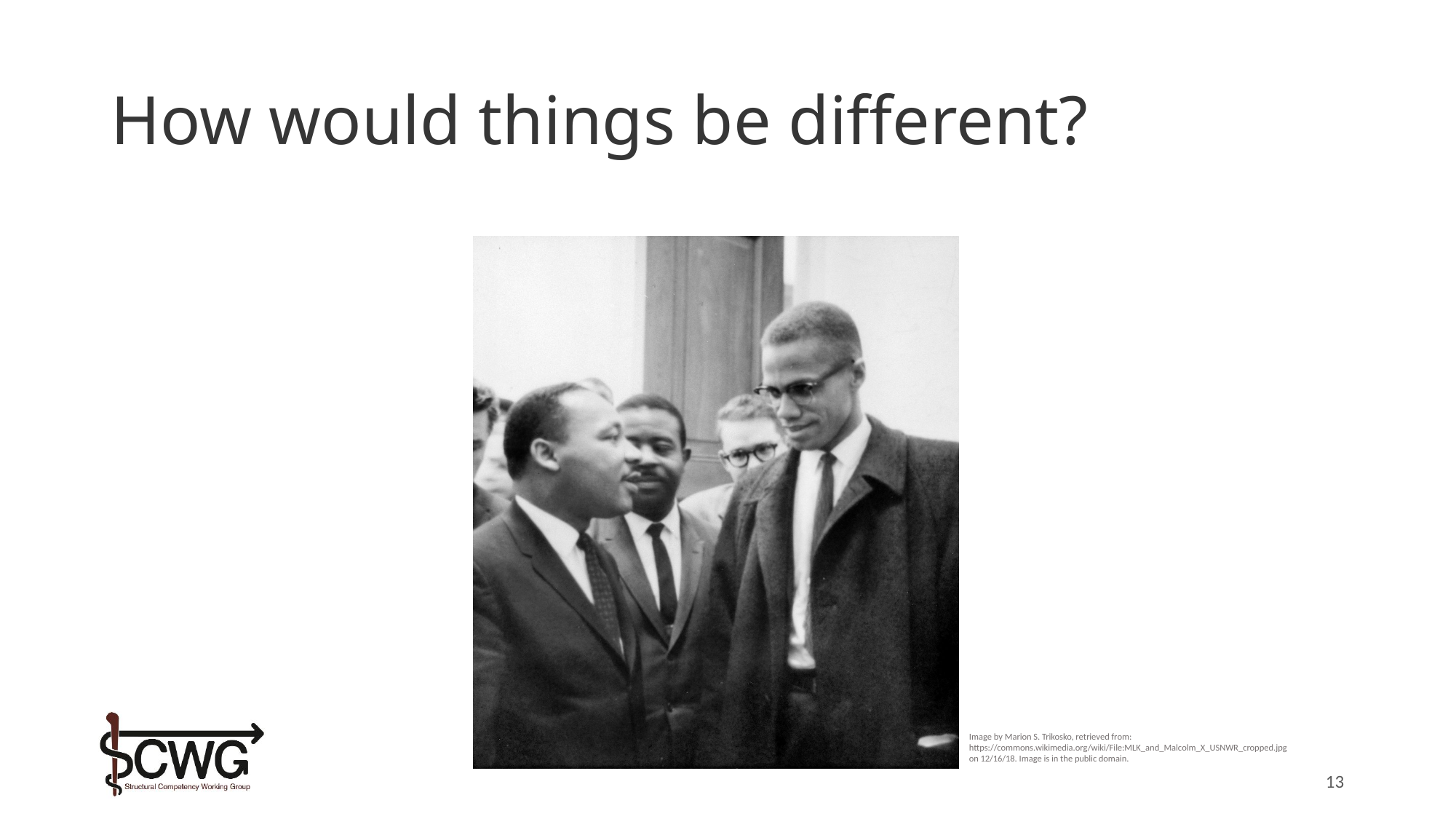

# How would things be different?
Image by Marion S. Trikosko, retrieved from: https://commons.wikimedia.org/wiki/File:MLK_and_Malcolm_X_USNWR_cropped.jpg on 12/16/18. Image is in the public domain.
13

## Slide 14
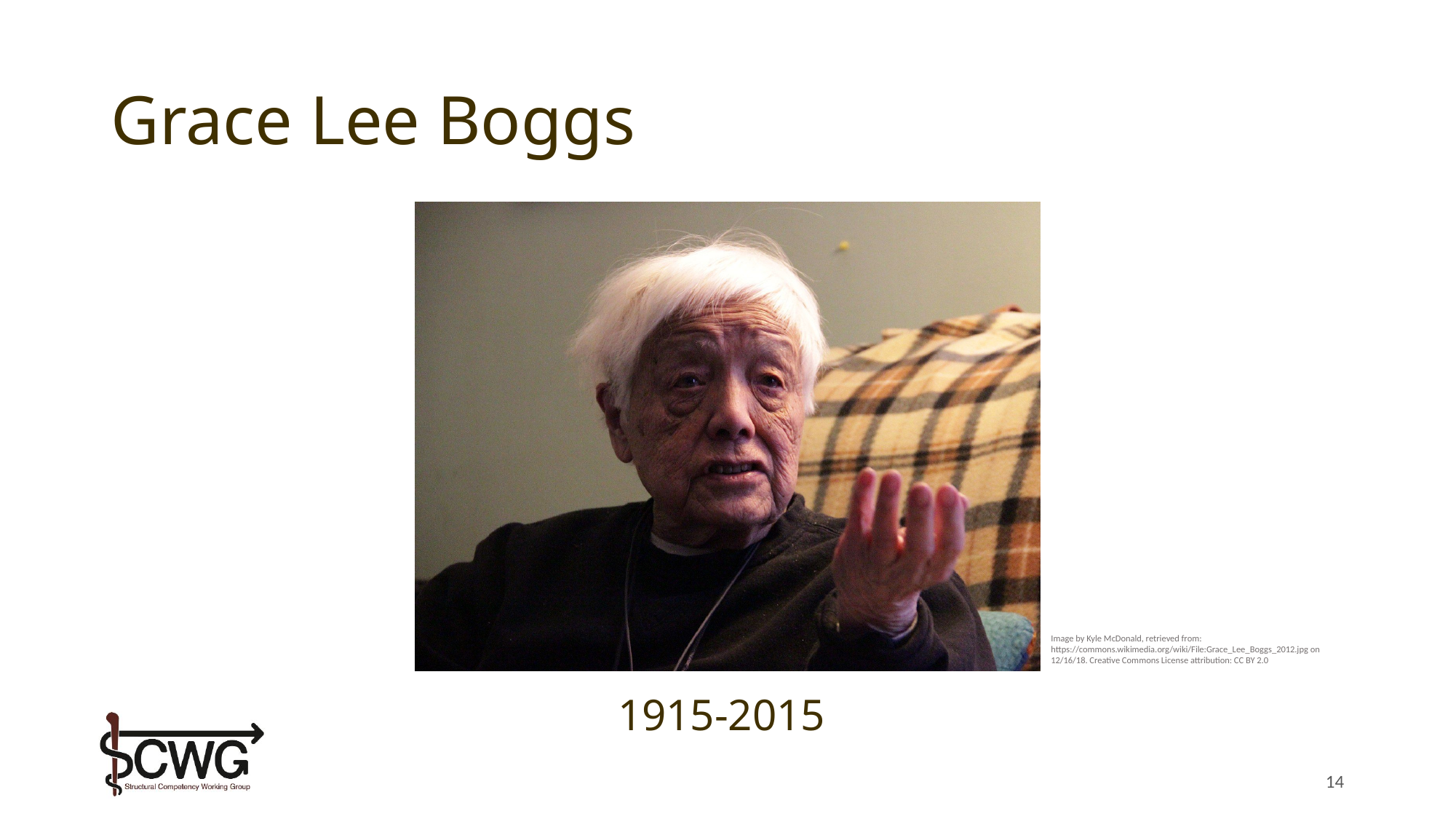

# Grace Lee Boggs
Image by Kyle McDonald, retrieved from: https://commons.wikimedia.org/wiki/File:Grace_Lee_Boggs_2012.jpg on 12/16/18. Creative Commons License attribution: CC BY 2.0
1915-2015
14

## Slide 15
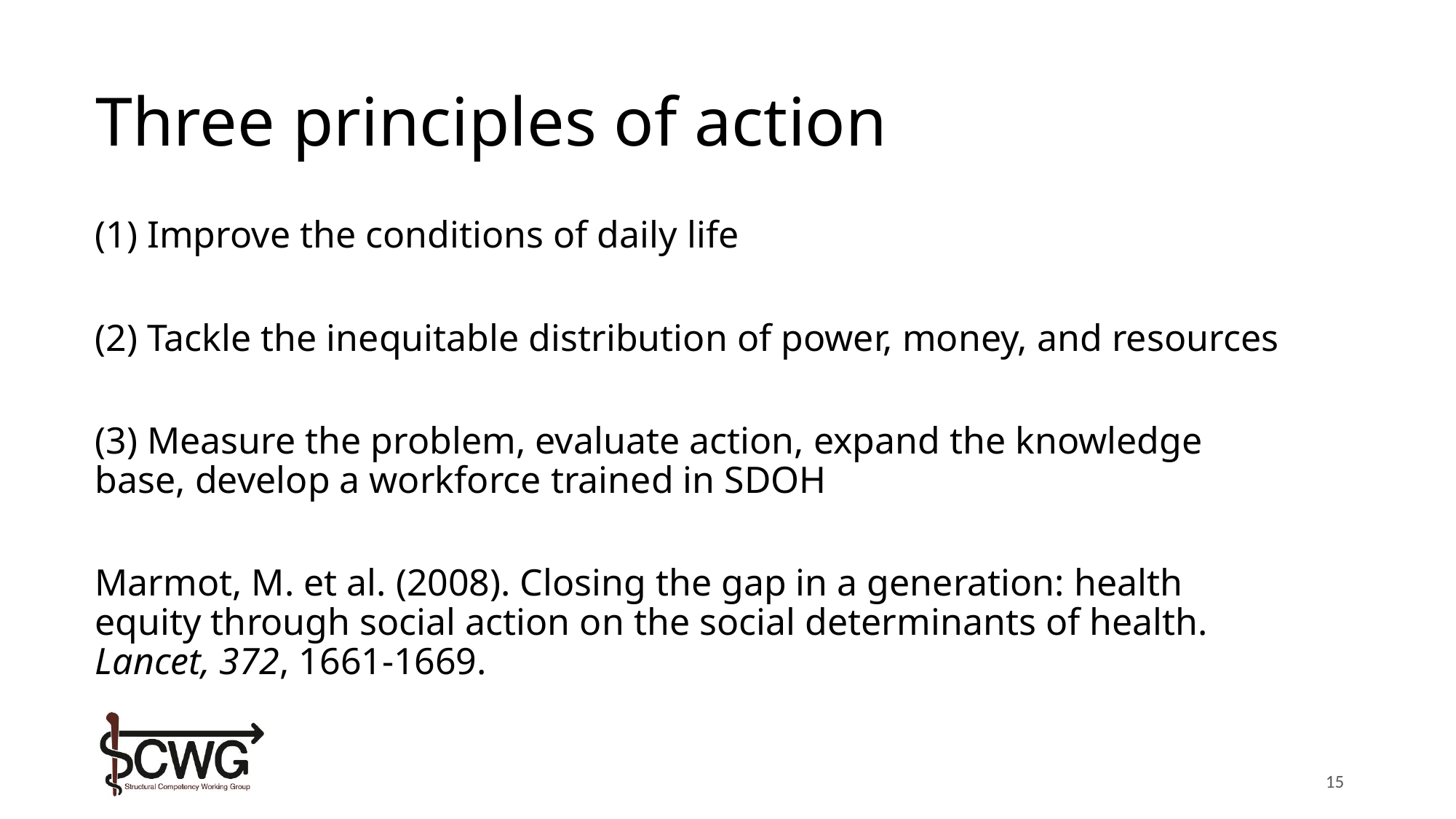

# Three principles of action
(1) Improve the conditions of daily life
(2) Tackle the inequitable distribution of power, money, and resources
(3) Measure the problem, evaluate action, expand the knowledge base, develop a workforce trained in SDOH
Marmot, M. et al. (2008). Closing the gap in a generation: health equity through social action on the social determinants of health. Lancet, 372, 1661-1669.
15

## Slide 16
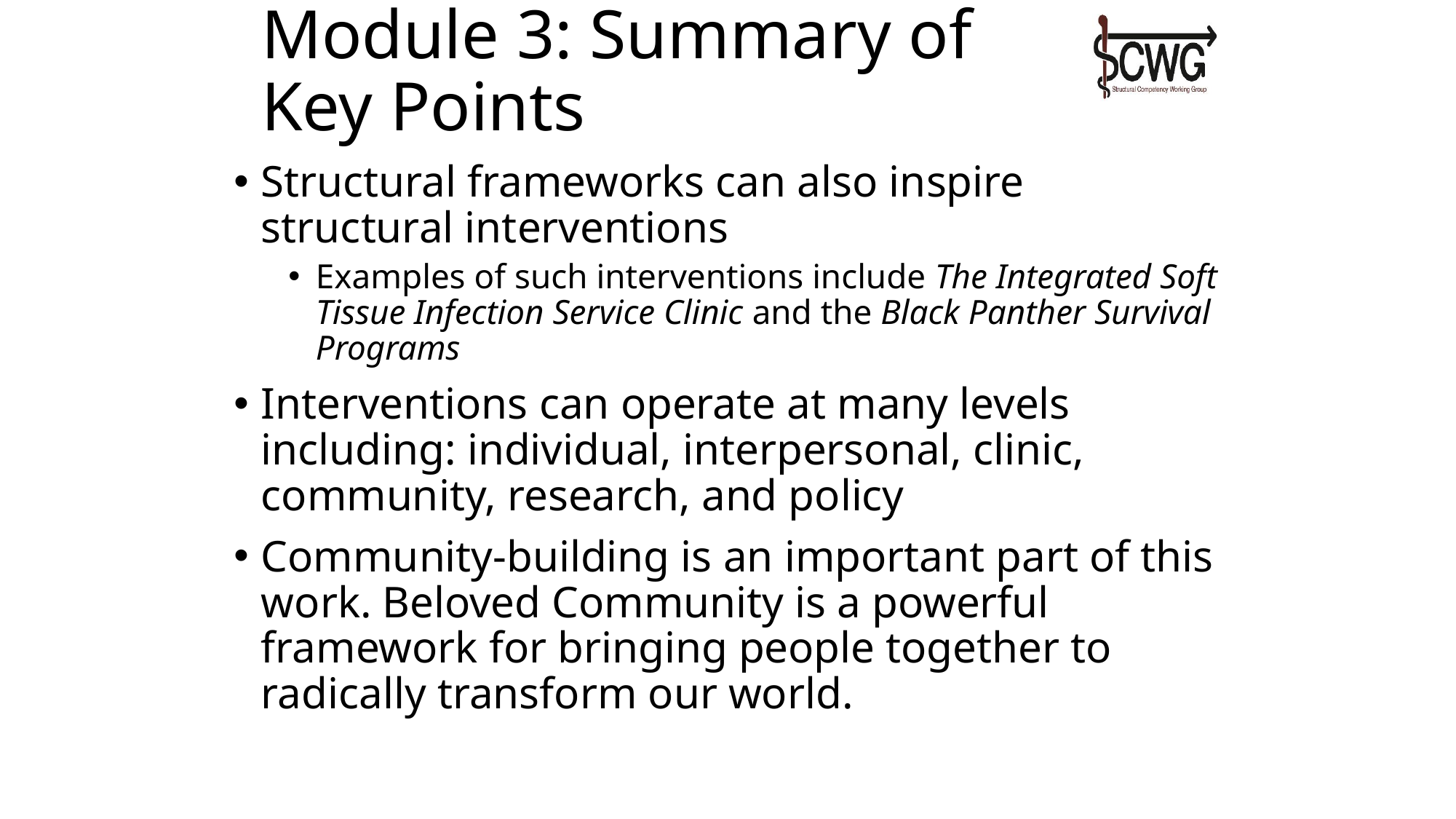

# Module 3: Summary of Key Points
Structural frameworks can also inspire structural interventions
Examples of such interventions include The Integrated Soft Tissue Infection Service Clinic and the Black Panther Survival Programs
Interventions can operate at many levels including: individual, interpersonal, clinic, community, research, and policy
Community-building is an important part of this work. Beloved Community is a powerful framework for bringing people together to radically transform our world.

## Slide 17
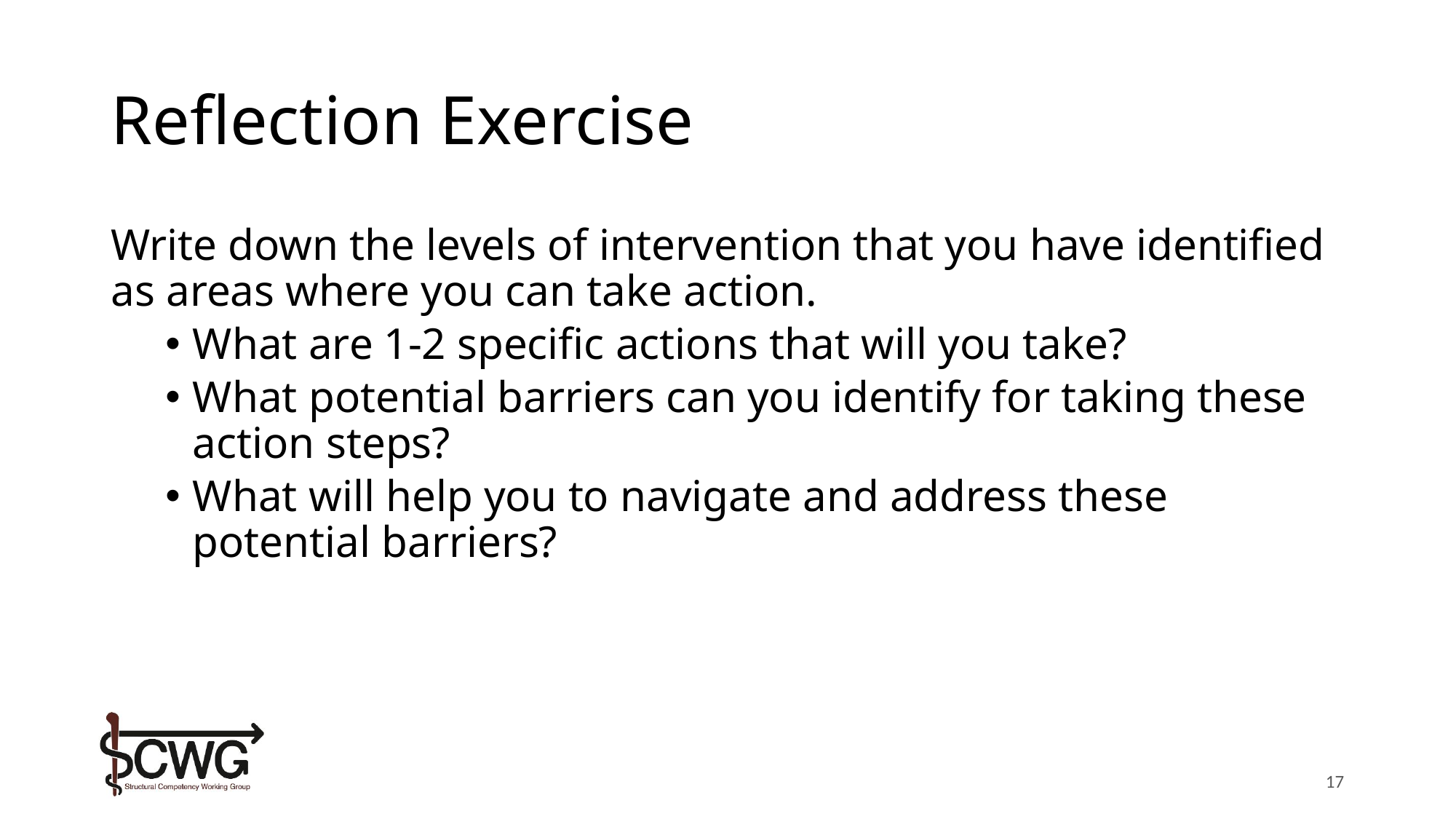

# Reflection Exercise
Write down the levels of intervention that you have identified as areas where you can take action.
What are 1-2 specific actions that will you take?
What potential barriers can you identify for taking these action steps?
What will help you to navigate and address these potential barriers?
17

## Slide 18
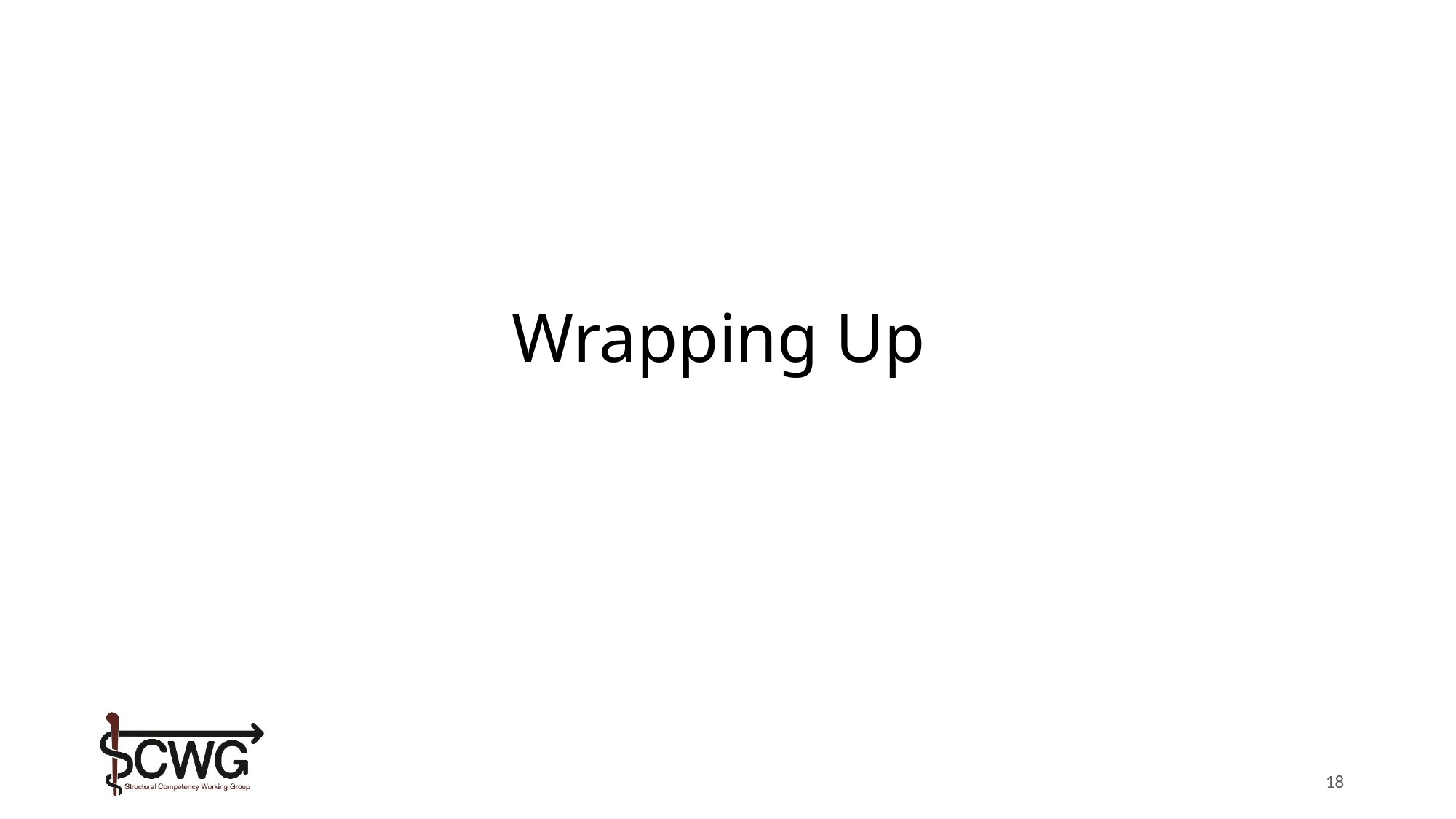

Wrapping Up
18

## Slide 19
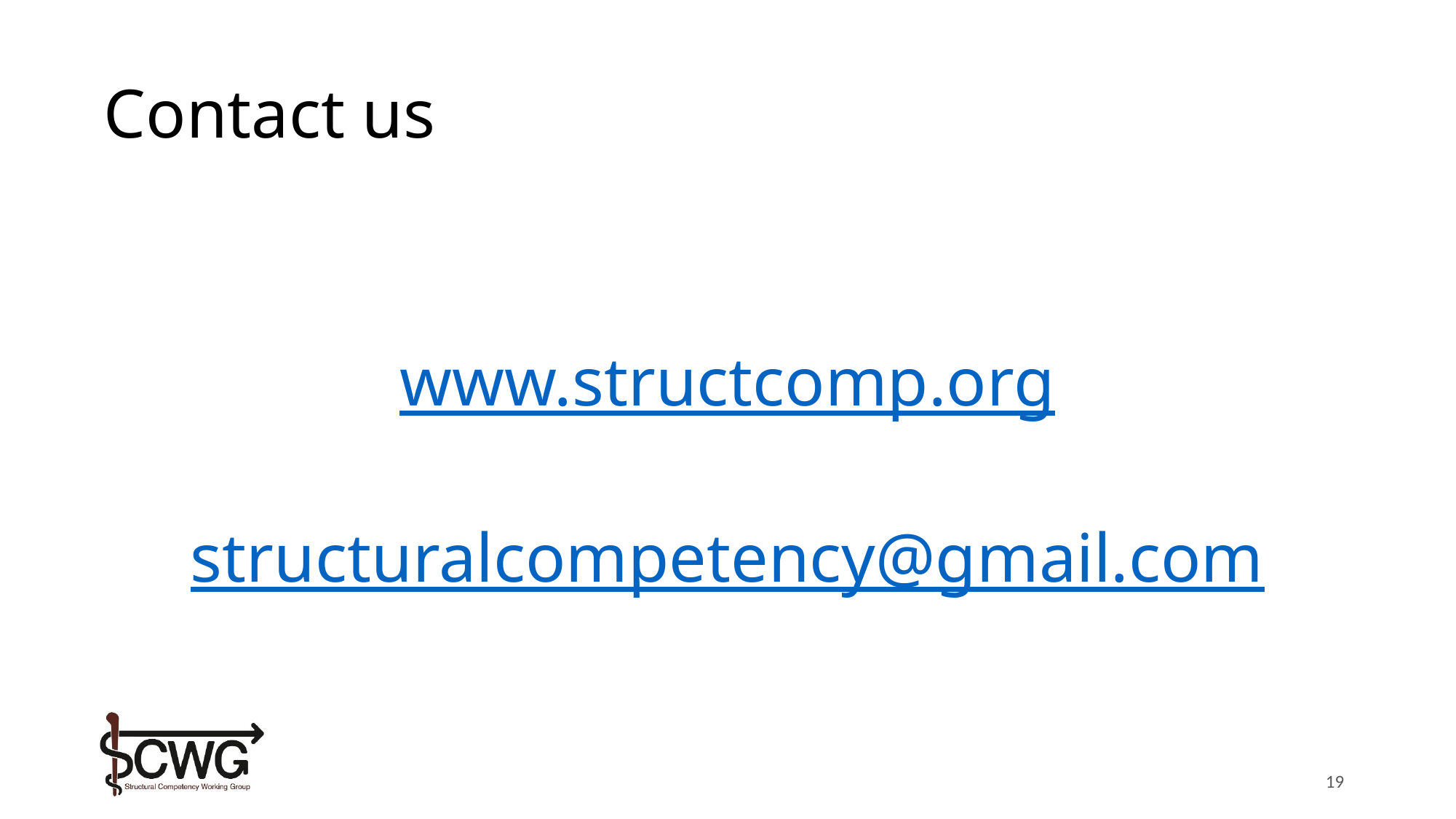

Contact us
www.structcomp.org
structuralcompetency@gmail.com
19

## Slide 20
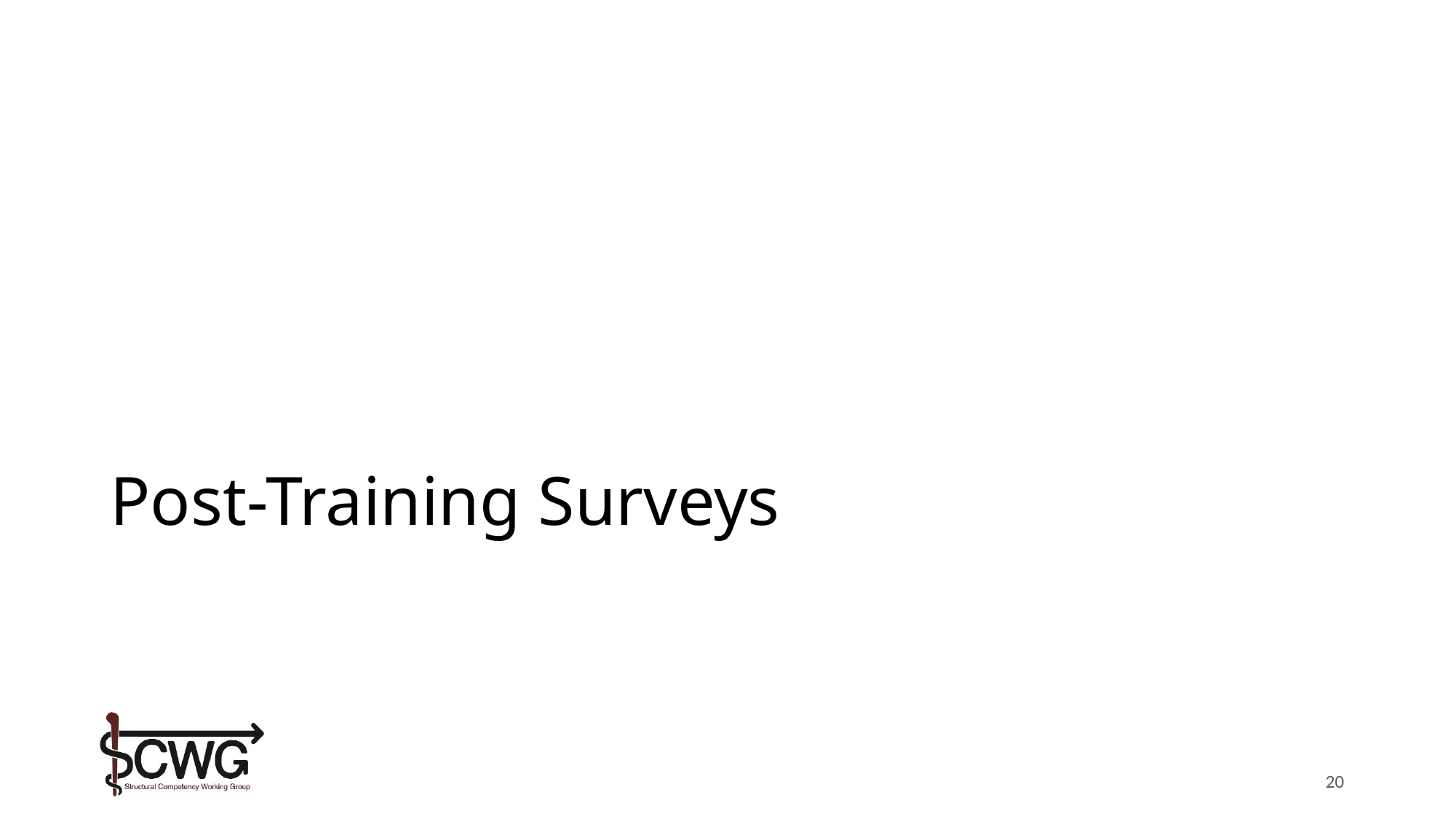

# Post-Training Surveys
20
