# Supplementary material for: Structural Competency: Curriculum for Medical Students, Residents, and Interprofessional Teams on the Structural Factors That Produce Health Disparities
Source: MedEdPORTAL. 2020 Mar 13;16:10888. doi: 10.15766/mep_2374-8265.10888 (PMC7182045; doi:10.15766/mep_2374-8265.10888)
Supplement: Supplementary file 1 — A. Manual Background Info.docx B. Manual Intro.docx C. Manual Module 1.docx D. Manual Module 2.docx E. Manual Module 3.docx F. Manual Conclusion and Evaluation.docx G. Supplemental Reading List.docx H. Training Slides Intro.pptx I. Training Slides Module 1.pptx J. Training Slides Module 2.pptx K. Training Slides Module 3.pptx L. Participant Workbook.pdf M. Posttraining Survey.pdf N. Facilitator Guidelines.docx O. Facilitator Preparation - Terms and Concepts.docx P. Participant Sign-in Sheet.docx [file mep-16-10888-s001.zip › L. Participant Workbook.pdf]

TRAINING PARTICIPANT WORKBOOK

Structural Competency Working Group

CONTENTS PAGE

1. Case Study 1
2. Key Concepts 3
3. Structural Vulnerability Checklist 5
4. Structural Violence Exercise 6
5. Naturalizing Inequality Exercise 7
6. Your Arrow Diagram Exercise 8
7. Components of Structural Competency 9
8. Levels of Intervention 10
9. Recommended Reading 13

CASE STUDY

Please read the following note describing an admission to the inpatient medicine service. When you have finished, discuss with your colleagues, imagining that you're the attending hearing this case. What questions do you have that might help you better understand the situation? What social, political, and economic structures might be contributing to this patient's problems?

Presenting Complaint: Acute loss of consciousness

History of Present Illness: Patient is a 37-year-old Spanish-speaking male found down with acute loss of consciousness. Was minimally responsive in ambulance, no response to naloxone, smell of alcohol on breath noted by first responders, pt. found on park bench w/empty cans of malt liquor. In Emergency Department the patient received fluids, initially somnolent but now tremulous and anxious despite IV lorazepam. Medicine consulted for admission for inpatient detox given risk of withdrawal.

Past Medical History: Frequent flyer well known to Emergency Department for alcohol-related trauma, assaults, withdrawal with associated seizures, and clearance for jail. Previous diagnosis of hypertension, treated for seizure disorder with anticonvulsants but lost to follow up.

Past Surgical History: Right orbital fracture secondary to assault w/o operative intervention, open reduction and internal fixation (ORIF) Right wrist secondary to alcohol-related fall, ORIF Left tibia/fibula for alcohol- related auto vs. pedestrian motor-vehicle accident.

Meds: currently noncompliant with all meds. Discharged after last hospitalization on folate, thiamine, multivitamin, and phenytoin 100mg orally 3x a day for seizure prophylaxis.

All: No Known Drug Allergies.
Family History: Not obtainable.
Social History: Heavy alcohol use, other habits unknown. Apparently homeless.
Review of Systems: Not obtainable.
Physical Exam:
Blood Pressure 165/89, Pulse 135, Respiration Rate 22, Temperature 37, 100% on Room Air.
General: malnourished, Hispanic male, disheveled, appears older than stated age.
Head, Eyes, Ears, Nose, Throat: Decent dentition.

Respiratory: Reduced breath sounds right base.
Cardiovascular: Regular rate and rhythm, no murmurs, rubs, or gallops.
Abdomen: 3cm tender hepatomegaly.
Ext: no edema, surgical scars noted.
Neurologic/Muscular Skeletal: patient muttering incoherently in Spanish. Alert and oriented to person and place, directable, able to answer "yes/no" consistently and follow simple commands. Denies pain. Tremulous, neuro nonfocal.

Labs: Alcohol level on presentation 0.35, CBC shows Hb 11.2 with MCV 105, AST 100 ALT 75, otherwise chemistry normal. EKG shows sinus tach.

Assessment: 37-year-old male noncompliant with meds with persistent Alcohol abuse and history of seizures presents with high alcohol level, now with signs of alcohol withdrawal.

1) Altered mental status: likely alcohol withdrawal, given history priors admission for similar. Do not suspect CNS or metabolic pathology. CIWA protocol instituted, patient admitted to floor with sitter. Fall precautions.
2) Hepatomegaly and elevated LFTS: likely alcohol hepatitis. Discriminant function does not indicate likely benefit from steroids, treat supportively.
3) Reduced breath sound right base: concerning for aspiration PNA given acute loss of consciousness – CXR PA and lateral.
4) Seizure disorder: unclear if primary or related to recurrent alcohol withdrawal; continue phenytoin in house.
5) Malnourishment: folate, thiamine, MVI
6) Homelessness: Medical Social Worker consulted for shelter/board and care given recurrent Emergency Department presentations.
7) Code: Full
8) Disposition: (Hospital) floor

What social, political, and economic structures might be contributing to this patient's problems?

KEY CONCEPTS

"Language is never neutral." — Paulo Freire.

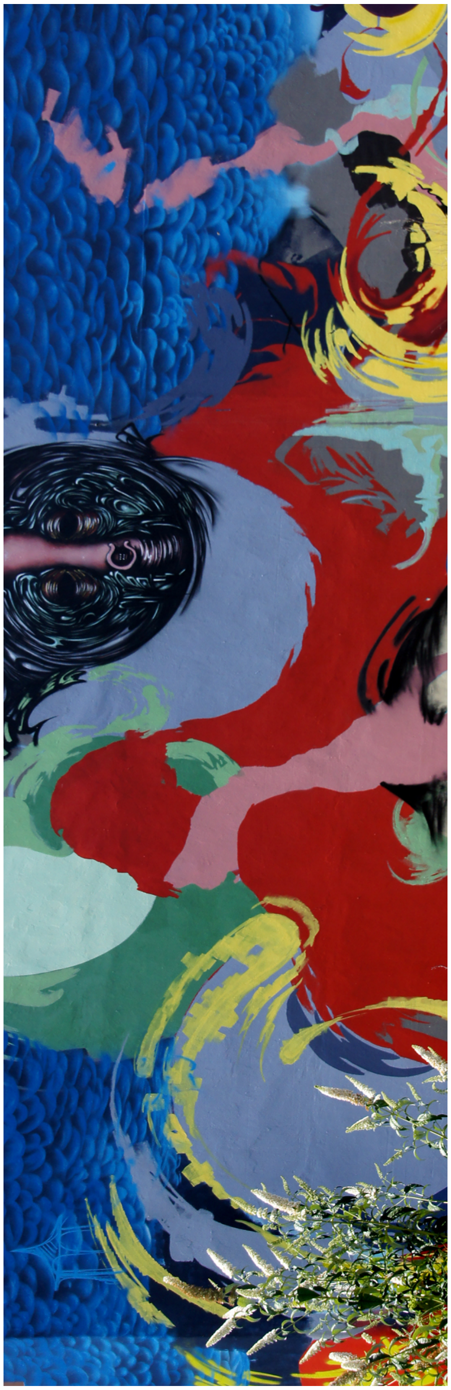

Image by Bernard Spragg. NZ, retrieved from https://www.flickr.com/photos/volvob12b/11462275126/in/photolist-isTa4d-XHeoFF-21CFRa6-XEuuuo-TcsToJ-Lo8fPQ-Kq5puX-XEuyPA-28Fggt1-Zxvu4Q-27sAe1q-YGfxJa-HuaHJK-29qcrBc-24yKpjT-oXvqDX-Yik6u9-oaNpxr-kZNAro-FNc1ko-iwS8bT-isTgj8-kZM9Ai-pJaTfH-8rhmE9-itZy9a-iwGmf7-puTY82-oqf669-69Afaq-ix8LyE-owtgFG-oeSXcE-jY5Hk2-oeVAjP-23ULiM8-fo45Zk-6VwuPD-bFdY8G-odAhUT-os4UJo-6VwrEZ-2dBPUMp-d2kQwf-oeURs6-drfYMX-edEDHs-fnvkde-isSTSD-iwV1U8 on 5/12/19. Image is in the public domain.

Social Structure: The policies, economic systems, and other institutions (policing & judicial systems, schools, etc.) that have produced and maintain social inequities and health disparities, often along the lines of social categories (race, class, gender, etc.)

Structural Violence: "Structural violence is one way of describing social arrangements that put individuals and populations in harm's way… The arrangements are structural because they are embedded in the political and economic organization of our social world; they are violent because they cause injury to people."

–Paul Farmer et al. 2006

Structural Vulnerability: The risk that an individual experiences as a result of structural violence – including their location in socioeconomic hierarchies. It is not caused by, nor can it be repaired solely by, individual agency or behaviors.

Structural Racism (aka "Institutional Racism")**:** "When white terrorists bomb a black church and kill five black children, that is an act of individual racism, widely deplored by most segments of the society. But when in that same city – Birmingham, Alabama – five hundred black babies die each year because of the lack of power, food, shelter and medical facilities, and thousands more are destroyed and maimed physically, emotionally and intellectually because of conditions of poverty and discrimination in the black community, that is a function of institutional racism. When a black family moves into a home in a white neighborhood and is stoned, burned or routed out, they are victims of an overt

act of individual racism which most people will condemn. But it is institutional racism that keeps black people locked in dilapidated slum tenements, subject to the

daily prey of exploitative slumlords, merchants, loan sharks and discriminatory real estate agents. The society either pretends it does not know of this latter situation, or is in fact incapable of doing anything meaningful about it."

-Kwame Ture (Stokely Carmichael) and Charles V. Hamilton

Black Power: The Politics of Liberation, 1967

Naturalizing Inequality: When inequality and structural violence are justified by—or go unacknowledged due to—ways of thinking that focus on individual behaviors, "cultural" characteristics, or biologized racial categories (see "implicit frameworks" below). This helps preserve social inequities by giving the impression that the current, inequitable status quo is "natural," in the sense of not being primarily social or structural in origin.

Implicit Frameworks: The common, taken-for-granted (implicit) ways of understanding health and wellness – among health professionals and in society more broadly. Examples include interpreting health disparities in terms of individual behavior, "culture," and biology/genetics, without also adequately considering underlying social and structural factors. Discussing implicit frameworks does not suggest that individual behaviors, culture, and genetics do not matter for health. Instead, this highlights ways we and others might inadvertently fail to recognize, acknowledge, and address the structural factors that are primary drivers of health disparities (see "naturalizing inequality" above).

STRUCTURAL VULNERABILITY CHECKLIST

From: Bourgois P, Holmes SM, Sue K, & Quesada J. (2017). "Structural Vulnerability: Operationalizing the Concept to Address Health Disparities in Clinical Care." *Academic Medicine, 92*(3): 299-307.

Chart 1

Structural Vulnerability Assessment Tool[a]

Domain	Screening questions and assessment probes[b]
Financial security	**Do you have enough money to live comfortably—pay rent, get food, pay utilities/telephone?**<br>• How do you make money? Do you have a hard time doing this work?<br>• Do you run out of money at the end of the month/week?<br>• Do you receive any forms of government assistance?<br>• Are there other ways you make money?<br>• Do you depend on anyone else for income?<br>• Have you ever been unable to pay for medical care or for medicines at the pharmacy?
Residence	**Do you have a safe, stable place to sleep and store your possessions?**<br>• How long have you lived/stayed there?<br>• Is the place where you live/stay clean/private/quiet/protected by a lease?
Risk environments	**Do the places where you spend your time each day feel safe and healthy?**<br>• Are you worried about being injured while working/trying to earn money?<br>• Are you exposed to any toxins or chemicals in your day-to-day environment?<br>• Are you exposed to violence? Are you exposed regularly to drug use and criminal activity?<br>• Are you scared to walk around your neighborhood at night/day?<br>• Have you been attacked/mugged/beaten/chased?
Food access	**Do you have adequate nutrition and access to healthy food?**<br>• What do you eat on most days?<br>• What did you eat yesterday?<br>• What are your favorite foods?<br>• Do you have cooking facilities?
Social network	**Do you have friends, family, or other people who help you when you need it?**<br>• Who are the members of your social network, family and friends? Do you feel this network is helpful or unhelpful to you? In what ways?<br>• Is anyone trying to hurt you?<br>• Do you have a primary care provider/other health professionals?
Legal status	**Do you have any legal problems?**<br>• Are you scared of getting in trouble because of your legal status?<br>• Are you scared the police might find you?<br>• Are you eligible for public services? Do you need help accessing these services?<br>• Have you ever been arrested and/or incarcerated?
Education	**Can you read?**<br>• In what language(s)? What level of education have you reached?<br>• Do you understand the documents and papers you must read and submit to obtain the services and resources you need?
Discrimination	***[Ask the patient]* Have you experienced discrimination?**<br>• Have you experienced discrimination based on your skin color, your accent, or where you are from?<br>• Have you experienced discrimination based on your gender or sexual orientation?<br>• Have you experienced discrimination for any other reason?<br>***[Ask yourself silently]* May some service providers (including me) find it difficult to work with this patient?**<br>• Could the interactional style of this patient alienate some service providers, eliciting potential stigma, stereotypical biases, or negative moral judgments?<br>• Could aspects of this patient's appearance, ethnicity, accent, etiquette, addiction status, personality, or behaviors cause some service providers to think this patient does not deserve/want or care about receiving top quality care?<br>• Is this patient likely to elicit distrust because of his/her behavior or appearance?<br>• May some service providers assume this patient deserves his/her plight in life because of his/her lifestyle or aspects of appearance?

SCWG Structural Competency Working Group

STRUCTURAL VIOLENCE EXERCISE

Please reflect on an example of structural violence you have witnessed, as a provider/ trainee or otherwise. Before you start writing, be sure to reflect: what were the structures involved, and how were they violent (i.e. what bodily and/or emotional harm did they cause

NATURALIZING INEQUALITY EXERCISE

Underline the parts of the passages below where you see inequality/injustice being naturalized through "Implicit Frameworks." Implicit Frameworks discussed include focusing on any of the following instead of the influence of structures: "culture," individual level behaviors/choices, and/or biology/genetics.

Excerpts from: Holmes SM. (2006). "An Ethnographic Study of the Social Context of Migrant Health in the US." *Public Library of Science Medicine, 3*(10).

#1: When asked why very few Triqui people were harvesting apples, the field job known to pay the most, the Tanaka Farm's apple crop supervisor explained in detail that "they are too short to reach the apples, and, besides, they don't like ladders anyway." He continued that Triqui people are perfect for picking berries because they are "lower to the ground." When asked why Triqui people have only berry-picking jobs, a mestiza Mexican social worker in Washington state explained that "a los Oaxaquenos les gusta trabajar agachado [Oaxacans like to work bent over]," whereas, she told me, "Mexicanos [mestizo Mexicans] get too many pains if they work in the fields." In these examples and the many other responses they represent, perceived bodily difference along ethnic lines serves to justify or naturalize inequalities, making them appear purely or primarily natural and not also social in origin. Thus, each kind of ethnic body is understood to deserve its relative social position.

#2: The urgent-care doctor he first saw explained that Abelino should not work, but should rest and let his knee recover. The occupational health doctor he saw the following week said Abelino could work but without bending, walking, or prolonged standing…. After a few weeks, the occupational health doctor passed Abelino to a reluctant physiatrist who told Abelino that he must work hard picking strawberries in order to make his knee better. She told Abelino that he had been picking incorrectly and hurt his knee because he "didn't know how to bend over correctly." Once Abelino had recovered, this doctor explained to the researcher that Abelino no longer felt pain, not because he got better, but because the picking season was over and he could no longer apply for worker's compensation…. Knee and back pain continue to be the most common health complaints among pickers on the Tanaka Farm.

YOUR ARROW DIAGRAM EXERCISE

1) *In the space below, write out your personal trajectory. What has your trajectory been up until now, as a provider/trainee and otherwise? You may want to include why you chose to go into healthcare. If you are early in your career, you could consider the possible trajectories you could have moving forward.*
2) *Next, in a different color, if available, identify the structures that have influenced (or might influence) your trajectory. What structures have given you advantages? What harmful/unjust structures have you encountered? Have any structures put you at risk for feeling burned out?*
3) *For Module 3: what strategies could modify your trajectory moving forward?*

COMPONENTS OF STRUCTURAL COMPETENCY

Structural Competency *is the capacity for health professionals to recognize and respond to health and illness as the downstream effects of broad social, political, and economic structures.*

"A shift in medical education … toward attention to forces that influence health outcomes at levels above individual interactions."

– Metzl and Hansen 2014

Components of Structural Competency

1. Recognizing influences of structures on patient health
2. Recognizing influences of structures on the clinical encounter, including implicit frameworks common in healthcare
3. Responding to structures in the clinic
4. Responding to structures beyond the clinic
5. Structural humility

Structural Humility

An orientation emphasizing collaboration with patients and communities in developing responses to structural vulnerability, rather than assuming that health professionals know best. This includes (but is not limited to) awareness of interpersonal privilege and power hierarchies in healthcare.

Levels of Intervention

1. Individual
2. Interpersonal
3. Clinic/Institutional
4. Community
5. Policy
6. Research

LEVELS OF INTERVENTION

Listed below are potential structural challenges and interventions at each of the levels. Note that many items could potentially fall under multiple headings.

Level	Challenges	Strategies
**Individual**	• "Implicit Bias"<br>• Discrimination: Racism, sexism, heteronormativity, ageism<br>• Moral judgments of patient behavior<br>• Negative/blaming language<br>• Concern for medical education debt and choice of career path<br>• Ignorance of structural problems and solutions/services	• Education<br>• Find way to one-self accountable<br>• Use neutral language<br>• Ask more questions of your patients<br>• Talk less, listen more<br>• Cultivate structural humility
**Interpersonal**	• Language Barriers (including complex medical jargon/terminology)<br>• Power imbalance between patient and provider<br>• Training and/or clinical team hierarchies<br>• The "Hidden" Curriculum<br>• Time constraints<br>• Student needs (learning, performance) balanced with patient needs<br>• Exploitation of patients (both historical and immediate)<br>• Preference for biomedical interpretation over patient interpretation	• Use existing support service (interpreters, etc.) and use real language<br>• Recognize the hierarchies, practice humility, resist where you can, use your status for good where appropriate/possible (med students).<br>• Understand that medical professionals have a culture as well.<br>• Structural vulnerability checklist (as a tool to avoid assumptions, address patient needs
**Clinic/ Institutional**	• Poor interpretation services<br>• Inaccessible for families (hours of operation, location, etc.)<br>• Disorganized, chaotic care (different providers)<br>• Not adapted to patient/community needs<br>• Providers feeling overstretched, time pressures<br>• Underfunding	• Restructure clinic within constraints to best meet patient needs, advocate to change the restraints<br>• Community engagement –ask what they need<br>• Case management<br>• Integration of behavioral services with mental health services

LEVELS OF INTERVENTION

Level	Challenges	Strategies
**Community**	• Lack of community representation<br>• Exploitation of communities<br>• Community policing practices leading to violence and trauma<br>• Poor access to clean water<br>• Poor access to affordable gas and electricity<br>• Poor access to healthy food<br>• High levels of toxicity, environmental racism/classism	• Create opportunities for community voices/leadership<br>• Work to educate police about the health costs of policing/incarceration<br>• Partner with CBOs working on structural issue outside of clinical settings<br>• Affordable and safe ride share opportunities for lower income communities<br>• Community food gardens<br>• Community organizing for safe water, lower neighborhood toxicity<br>• Home/phone visits<br>• Group visits<br>Use your white coat/title as symbolic capital
**Policy**	• Immigration and housing policies<br>• SSI benefits that require mental health diagnosis<br>• Prison industrial complex and criminalization of drug use<br>• Medicare value measurements that contribute to pressures<br>• Access to/Cost of pharmaceuticals<br>• Lack of diversity/inclusion in health professional education instructors<br>• Lack of formal curriculum on structural determinants of health in health profession schools	• Refuse to report undocumented migrants<br>• Contact media, seek out radio speaking opportunities<br>• Write media articles, editorials, and position statements demonstrating the relationship between policies and poor health<br>• Challenge claims (e.g. based on genetics) that naturalize inequality<br>• Research the historical effects of policies<br>• Make pharmaceutical access inequity transparent through blog posts, social media, and formal media (e.g. Shkreli)<br>• Activism - Be a medic or wear your white coat (with permission from organizers) at rallies, marches, etc.<br>• #whitecoats4blacklives and other student movements to change admissions policies, national policies about policing and incarceration<br>• Medical education reform

LEVELS OF INTERVENTION

Level	Challenges	Strategies
**Research**	• Emphasis on quantitative research that takes for granted social categories<br>• Demand for particular kinds of evidence<br>• Lack of funding for social science research relative to basic science<br>• Publishing bias-research preferentially published from elite universities	• Engage patients in defining important research questions and aims<br>• Situate research in a structural context<br>• Use the accepted forms of evidence to point to structural causes for health disparities<br>• Research the historical effects of policies<br>Advocate for better funding for qualitative research

RECOMMENDED READING

This training barely scratches the surface. Here is a list of reading to go a little deeper.

Defining, Teaching, & Operationalizing Structural Competency

- Metzl J, & Hansen H. (2014). "Structural competency: Theorizing a new medical engagement with stigma and inequality." *Social Science and Medicine*.
- Neff J, Knight KR, Satterwhite S, Nelson N, Matthews J, & Holmes SM. (2017). "Teaching Structure: A Qualitative Evaluation of a Structural Competency Training for Resident Physicians." *Journal of General Internal Medicine*.
- Bourgois P, Holmes SM, Sue K, & Quesada J. (2017). "Structural Vulnerability: Operationalizing the Concept to Address Health Disparities in Clinical Care." *Academic Medicine*, *92*(3): 299-307.

The Need for Structural Competency in Healthcare Education

- Bourgois P, & Schonberg J. (2009). Male Love. In, *Righteous Dopefiend* (Chapter 7). Berkeley: University of California Press.
- Farmer PE, Nizeye B, Stulac S, & Keshavjee S. (2006). "Structural Violence & Clinical Medicine." *Public Library of Science Medicine*.
- Holmes S. (2006). "An Ethnographic Study of the Social Context of Migrant Health in the US." *Social Science and Medicine*, *3*(10).
- Rivkin-Fish M. (2011). "Learning the Moral Economy of Commodified Health Care: 'Community Education,' Failed Consumers, and the Shaping of Ethical Clinician-Citizens." *Culture, Medicine and Psychiatry 35*(2): 183-205.

Limitations of Behavioral, "Cultural," & Biological Framing of Health Disparities

- Baum F, & Fisher M. (2014). "Why Behavioral Health Promotion Endures Despite Its Failure to Reduce Health Inequities." *Sociology of Health and Illness, 36*(2): 213-225.
- Gregg J, & Saha S. (2006). "Losing Culture on the Way to Competence: The Use and Misuse of Culture in Medical Education." *Academic Medicine, 81*(6): 542-546.
- Hunt LM, Schneider S, & Comer B. (2004). "Should "acculturation" be a variable in health research? A critical review of research on US Hispanics." *Social Science & Medicine*.
- Jenks A. (2011). "From 'Lists of Traits' to 'Open-Mindedness': Emerging Issues in Cultural Competence Education." *Culture, Medicine, and Psychiatry, 35*(2): 209-231.

- Kleinman & Benson. (2006). "Anthropology in the Clinic: The Problem of Cultural Competency and How to Fix It." *Public Library of Science Medicine*.
- Metzl JM, & Roberts DE. (2014). "Structural competency meets structural racism: race, politics, and the structure of medical knowledge." *Virtual Mentor, 16*(9): 674-690.
- Tervalon M & Murray-García J. "Cultural Humility Versus Cultural Competence." *Journal of Healthcare for the Poor and Underserved*, *9*(2): 117-123.

Structural Violence & Structural Racism

- Bourgois P. (2010). Recognizing Invisible Violence: A Thirty-Year Ethnographic Retrospective. In Rylko-Bauer B, Whiteford L, & Farmer P (Eds.) *Global Health in Times of Violence* (17-40). Santa Fe, NM: School for Advanced Research Press.
- Coates TN. (June 2014). The Case for Reparations. *The Atlantic.*
- Crenshaw K. (1989). "Demarginalizing the Intersection of Race and Sex: A Black Feminist Critique of Antidiscrimination Doctrine, Feminist Theory and Antiracist Politics." *University of Chicago Legal Forum*.
- Farmer P. (1996). "On Suffering and Structural Violence: A View from Below." *Daedalus*, *125*(1): 261-283.
- Holmes, SM. (2013). *Fresh Fruit, Broken Bodies.* University of California Press.
- Knight, KR. (2015). *addicted.pregnant.poor.* Duke University Press.
- West C. (1993). The Pitfalls of Racial Reasoning. In *Race Matters* (Chapter 2) Boston: Beacon Press.

Foundational Social Theory & Structural Analyses of Healthcare

- Brown W. (2006). "American nightmare: neoliberalism, neoconservatism, and de-democratization." *Political theory, 34*(6): 690-714.
- Fanon F. (1965). Medicine and Colonialism. In *A Dying Colonialism* (121-145) New York: Grove Press.
- Navarro V. (1988). "Professional Dominance or Proletarianization?: Neither." *The Milbank Quarterly*.
- Wacquant LJ. (2006). Pierre Bourdieu. In Stones R (Ed.) *Key Contemporary Thinkers* (Chapter 16) London and New York: Macmillan.

Writings on Teaching & Framing

- Boler M. (2004). Teaching for Hope: The Ethics of Shattering World Views. In Liston D & Garrison J (Eds.) *Teaching, Learning, and Loving* (114-129) New York and London: Routledgefalmer.

- Lakoff G. (2014). Framing 101. In *Don't Think of an Elephant! Know Your Values and Frame the Debate*. (Chapter 1).
- Wear D, & Aultman J. (2005). "The Limits of Narrative: Medical Student Resistance to Confronting Inequality and Oppression in Literature and Beyond." *Medical Education*, *39*(10): 1056-1064.
- Willen S. (2013). "Confronting a 'Big Huge Gaping Wound': Emotion and Anxiety in a Cultural Sensitivity Course for Psychiatry Residents." *Culture, Medicine and Psychiatry 37*(2): 253-276.

Social Medicine: Examples of Healthcare-Based Responses to Harmful Social Structures

- Breilh J. (2008). "Latin American critical ('Social') epidemiology: new settings for an old dream." *International Journal of Epidemiology 37*(4): 745-750.
- Geiger JH. (1984). Community health centers: health care as an instrument of social change. In Sidel VW & Sidel R (Eds.) *Reforming Medicine: Lessons of the Last Quarter Century* (11-32) New York: Pantheon Books.
- Holmes, Stonington, & Green. (2014). "Locating global health in social medicine." *Global Public Health.*
- Messac L, Ciccarone D, Draine J, & Bourgois P. (2013). "The Good-Enough Science-and-Politics of Anthropological Collaboration with Evidence-Based Clinical Research: Four Ethnographic Case Studies." *Social Science and Medicine, 99*:176-186.
- Nelson A. (2016). "The longue durée of Black Lives Matter." *American Journal of Public Health*, *106*(10): 1734–1737.
- Phelan, Link, & Tehranifar. (2010). "Social Conditions as Fundamental Causes of Health Inequalities: Theory, Evidence, and Policy Implications." *Journal of Health and Social Behavior*.
- Porter D. (2006). "How Did Social Medicine Evolve, and Where Is It Heading?" *Public Library of Science Medicine*, *10*: 1667-1671.
- Virchow R. (2006). "Report on the Typhus Epidemic in Upper Silesia." *American Journal of Public Health*, *96*(12): 2102-2105.
- Waitzkin H. (2001). "Social medicine in Latin America: productivity and dangers facing the major national groups." *The Lancet.*
