# Supplementary material for: Structural Competency: Curriculum for Medical Students, Residents, and Interprofessional Teams on the Structural Factors That Produce Health Disparities
Source: MedEdPORTAL. 2020 Mar 13;16:10888. doi: 10.15766/mep_2374-8265.10888 (PMC7182045; doi:10.15766/mep_2374-8265.10888)
Supplement: Supplementary file 1 — A. Manual Background Info.docx B. Manual Intro.docx C. Manual Module 1.docx D. Manual Module 2.docx E. Manual Module 3.docx F. Manual Conclusion and Evaluation.docx G. Supplemental Reading List.docx H. Training Slides Intro.pptx I. Training Slides Module 1.pptx J. Training Slides Module 2.pptx K. Training Slides Module 3.pptx L. Participant Workbook.pdf M. Posttraining Survey.pdf N. Facilitator Guidelines.docx O. Facilitator Preparation - Terms and Concepts.docx P. Participant Sign-in Sheet.docx [file mep-16-10888-s001.zip › M. Posttraining Survey.pdf]

Please write legibly and in complete sentences. Note that positive and constructive feedback is solicited on the 2nd page of the survey.

1) Which aspects of the material presented today were new to you? Which aspects were already familiar?

2) Which elements of today's training did you find most valuable? Any tools/ concepts/ strategies that you found particularly useful?

3) In what ways (if any) do you expect structural competency frameworks to be useful to you in the coming weeks and months, or in your career/training in the longer term?

4) After this session, please describe any ways that you feel structural competency could influence (a) your or others' empathy for patients, and/or (b) your or others' personal sense of empowerment/burnout?

5) a) Please share your candid thoughts on this training. What parts worked well? What parts did you like? What expectations of yours did we meet or exceed?

b) What should we change? How can we make this session more effective? Any ways we could have better met your expectations?

6) Please briefly summarize the meaning of the following terms:

   (a) structural violence

   (b) naturalizing inequality

   (c) structural competency

7) Thank you for your participation during the session and for filling out this survey. Any additional comments or feedback?
