# Supplementary material for: Structural Competency: Curriculum for Medical Students, Residents, and Interprofessional Teams on the Structural Factors That Produce Health Disparities
Source: MedEdPORTAL. 2020 Mar 13;16:10888. doi: 10.15766/mep_2374-8265.10888 (PMC7182045; doi:10.15766/mep_2374-8265.10888)
Supplement: Supplementary file 1 — A. Manual Background Info.docx B. Manual Intro.docx C. Manual Module 1.docx D. Manual Module 2.docx E. Manual Module 3.docx F. Manual Conclusion and Evaluation.docx G. Supplemental Reading List.docx H. Training Slides Intro.pptx I. Training Slides Module 1.pptx J. Training Slides Module 2.pptx K. Training Slides Module 3.pptx L. Participant Workbook.pdf M. Posttraining Survey.pdf N. Facilitator Guidelines.docx O. Facilitator Preparation - Terms and Concepts.docx P. Participant Sign-in Sheet.docx [file mep-16-10888-s001.zip › N. Facilitator Guidelines.docx]

STRUCTURAL COMPETENCY:

A Framework for Recognizing & Responding to Social, Political & Economic Structures to Improve Health


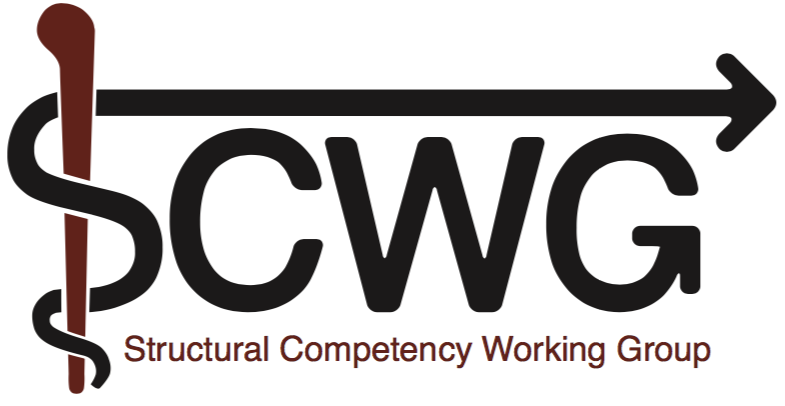


FACILITATOR GUIDELINES

Updated September 2018

Copyright:

The Structural Competency Working Group ([www.structcomp.org](http://www.structcomp.org)), in order to promote the widest possible dissemination of health curricula, has adopted an open copyright policy for its structural competency training materials. This means that we will grant permission to translate, adapt, or borrow our materials without charging fees or royalties under the following conditions:

- that you credit the Structural Competency Working Group for any borrowed/adapted Working Group materials and inform your audience about who we are and how to learn more about our organization ([structcomp.org](http://structcomp.org)). We suggest the following attribution (and request that it appear on any copyright page):
  - “*These materials have been [borrowed][adapted] from the Structural Competency Working Group,* [*www.structuralcompetency.org*](http://www.structuralcompetency.org)*; you can contact the Group at* [*structuralcompentency@gmail.com*](mailto:structuralcompentency@gmail.com)*”;*
- that your materials are distributed at no cost (or for your production cost only), that is, not-for-profit;
- that you allow others to reproduce/ adapt your edition or adaptation with no fees, royalties, etc. so long as they also do so at no cost or for production cost only, that is, not-for-profit;
- that you provide us with digital versions of your materials (including PDF and/or Microsoft Word files) and work with us so that we can host it on our website;
- that you send us your contact information so we can post it on our website and provide it to people who want to contact you about your edition, adaptation or publication;
- that you contact and stay in touch with the Structural Competency Working Group so that we can learn about your project and make sure you are using the most up-to-date materials.

If you decide to begin translating, adapting, or borrowing our materials, please use the most recently updated versions. Please contact us at [structuralcompetency@gmail.com](mailto:structuralcompetency@gmail.com) to find out if we are currently working on updating our materials, and if anyone else is working on a project similar to your own.

This training curriculum was prepared by Josh Neff, Seth M. Holmes, Kelly R. Knight, Shirley Strong, Ariana Thompson-Lastad, Cara McGuinness, Laura Duncan, Michael J. Harvey, Nimish Saxena, Katiana L. Carey-Simms, Alice Langford, Sara Minahan, Shannon Satterwhite, Lillian Walkover, Jorge De Avila, Brett Lewis, Gregory Chin, Jenifer Matthews, and Nick Nelson of the Structural Competency Working Group ([structcomp.org](http://structcomp.org/)), in collaboration with Sonia Lee and Caitlin Ruppel of Health Outreach Partners ([outreach-partners.org](http://outreach-partners.org/)).

The Structural Competency Working Group’s efforts have been supported by the Berkeley Center for Social Medicine and Deborah Lustig as well as the University of California Humanities Research Institute. Josh Neff’s work on this project has been supported by the UCSF Resource Allocation Program for Trainees (RAPtr), the Greater Good Science Center Hornaday Fellowship, UC Berkeley-UCSF Joint Medical Program Thesis Grant, and the Helen Marguerite Schoeneman Scholarship.

| Required Reading for Facilitator: | - Suarez-Orozco, C., Casanova, S., Martin, M., Katsiaficas, D., Cuellar, V., Smith, N. A., & Dias, S. A. (2015). Toxic Rain in Class: Classroom Interpersonal Microaggressions. *Educational Researcher,* 44(3), 151-160. - Sue, D., Lin, A., Torino, G., Capodilupo, C., & Rivera, D. (2009). Racial Microaggressions and Difficult Dialogues on Race in the Classroom. *Cultural Diversity and Ethnic Minority Psychology,* 15(2), 183-190. |
| --- | --- |
|  |  |

Facilitator Guidelines

A Note to the Facilitator

This guide was created to help prepare facilitators of structural competency trainings. It is meant specifically to accompany the structural competency training designed by the Structural Competency Working Group (SCWG) for delivery to a healthcare professional audience. Along with this guide and curriculum, facilitators will need to read and be familiar with the accompanying slide deck, required facilitator reading, the helpful terms and concepts list, and the Participant Workbook handouts prior to the delivery of the training.

SCWG’s structural competency training engages participants around topics such as health equity, social justice, politics, and identity. These topics can be sensitive in nature, elicit strong emotional reactions, and require conversations that may be uncomfortable for some participants. Establishing and maintaining a respectful, inclusive training environment is crucial.

Importantly, the facilitator needs to be prepared, as the facilitator will set the tone, establish trust, provide guidance, and model the behaviors and language needed to create an inclusive and engaging learning environment. Below, we share some guidelines to help the facilitator(s) feel equipped and ready to deliver the structural competency training.

**Positionality:** At the start of each training, acknowledge the positionality/privilege and blind spots of the facilitator(s). It is important to remember that no matter who we are, we all have our blind spots. Our perspectives are often determined by factors consisting of our race, ethnicity, sexual orientation, gender identity, family dynamics, culture, language, nationality, social class, and lived experiences. These factors also contribute to how we are perceived and treated daily. Remember that privilege can lead to blind spots in our thinking, which can make us less aware or even unaware of issues that that do not directly affect us. If we don’t recognize such blind spots, we risk causing harm even if unintended. Therefore, we encourage self-reflection prior to and throughout your role as the training facilitator.

**Employing “Expertise” Humility:** As with acknowledging blind spots, it is important to also share with the training participants that you do not claim to be an expert in structural competency. There is always more to learn! This shows a sense of humility. You can state that this is information that you believe in, so you want to share it with them, but that you will not have all of the answers and there are some things that you still do not know or still need to flesh out for yourself. More importantly, as a facilitator, when you do not know something, be upfront about it. You can state, for example, the following: “I don’t know the answer to that,” or, “I need to think more about that. I will research it and get back to you.”

**Safe Learning Environment:** It is important to set a tone that fosters a learning environment that feels safe and comfortable to participants. Although it is important to explicitly state the desire for this type of learning environment, simply stating, “This is a safe space,” is not enough. Again, the facilitator can play a strong role in creating this space by modeling behavior that reflects openness, respect, and non-judgement. Facilitators also need to know when to step in or step back. Encouraging active participation, especially during group discussions and small group activities, is part of the training approach. However, the participants will rely and “trust” the facilitator(s) to hold the group in check when needed, and steer discussions back where they need to go. Although remaining somewhat “neutral” and encouraging different perspectives is important, the facilitator(s) will need to be able to advocate for and defend the content being presented. To cultivate a safe learning environment, first start by working with the group to establish group agreements (see next tip).

**Group Agreements:** Converse with participants to establish group agreements (“ground rules”) and expectations for the session. Although you are asking the group to develop these agreements, it is usually best to come prepared with some ideas and propose it to the group, and then ask them to suggest any others that they would add or remove from the list. Write the agreements on flipchart paper. Once the list is complete, ask the group for their verbal agreement or you can use a consensus approach. This approach consists of three hand signals: thumbs up – agree, thumbs sideways – don’t fully agree but I can live with it, and thumbs down – don’t agree but I can offer an alternative. Once all are in agreement or there is consensus, hang the group agreements on the wall for the remainder of the training, and state that they will be in effect throughout the session. This is helpful for cases when you are faced with a challenging training participant or situation. You can refer back to the group agreements as a reminder and see how to resolve any issues based on the agreed upon code of behavior. Some examples of group agreements include:

1. Assume positive intent;
2. Respect the value of everyone’s opinions;
3. If you state a problem, try to offer a solution;
4. Everyone is actively participating and listening;
5. It’s ok to disagree, respectfully and openly;
6. Recognize your blind spots;
7. Be on time returning from breaks;
8. Turn off your cell phones and other devices.

**Framing:** Before each training module section, explain its overall purpose and the learning objectives. Consider checking in after each section to confirm that the learning objectives were met.

**Timing:** Keep an eye on time and stay on course, as it is one of the main responsibilities of the facilitator to keep the training on track and moving forward. There is a lot of content to cover and the purpose is to maximize the time as efficiently as possible; however, the facilitator needs to exercise judgment and flexibility as well as be able to “read the room.” This may mean moving on from discussions that, even if interesting, are tangential or that distract from central themes—or simply are taking up more time than has been allotted. However, you may find that some discussions are valuable to the participants’ learning and that time needs to be given to explore or expand upon that conversation. It is up to the facilitator to make the call or to ask the group for their input. For example, the facilitator could say to the group, “I want to keep us on time; however, I see that this is an important discussion or idea that we have tapped into. I’d like to propose that we spend the next 5 minutes exploring it further. If not, we can move forward, but I don’t want to forget it so let’s put it in the parking lot.”

**Keep a “Parking Lot:”** This is a facilitation tool to record ideas, questions, and issues that come up during the training, but that may not be useful to the training at the time. This way they can be returned to at a later time. It is a way to acknowledge to the participants that they have been heard, that what they have said is important, and that it is not forgotten. Write “Parking Lot” at the top of a flipchart paper, post it on the wall, and add items to it throughout the training, as needed, At the end of the training, spend 5-10 minutes revisiting the parking lot with the group, and ask them to help identify the items that need to be addressed either “now,” “later,” or “removed.”

**Start and End on Time:** Managing time for trainings is challenging and requires flexibility, and may result in the need for shifting or changing the time originally allocated to the sections of the training. However, the facilitator needs to be mindful of the participants’ time and of their expectations for the training. Participants are relying on the facilitator for this. Although adjustment to time might need to be made throughout the training, please make every effort to ensure that the training starts on time, and that even if it doesn’t, it is important that the training ends at the time stated in the agenda. Do not keep participants beyond the stated ending time.

**Do Not Compromise Time Allocated for Breaks:** Being in a training for multiple hours is not always easy. Maintaining focus and attention can be challenging, as a lot of content is being shared with participants. They will need breaks to decompress, rest, or move around. Therefore, it is best not to shorten the time allocated to them for these activities, and from our experience, participants are mostly never happy to compromise these times and may get angry or irritated with the facilitators if these times are disturbed. Additionally, this is where the facilitator’s ability to “read the room” will come in handy. If you feel or see that the group is having a hard time staying engaged, acknowledge it with them and take an impromptu 5-minute break, which can involve some guided stretching or just ask people to move around in whatever way feels rejuvenating for them.

**Acknowledge that the content is challenging:** The concept of structural competency is asking that we expand our understanding of what makes people sick or vulnerable and at risk for adverse health outcomes and diminished quality of life. This approach challenges the primacy of more commonly discussed interventions, such as prescribing medications or changing individual behavior through health education. Not everyone will be willing to accept what they are learning at first, and it may take time to really understand the concepts or even be open to it. Being upfront with the training participants may help to manage expectations and prepare for some challenging questions or comments.

**Provide real-life examples:** During the training, many concepts and definitions will be shared with the participants. Providing real-life examples, perhaps that you have experienced or know about, can help to better explain these concepts. Also, ask participants to share relevant experiences throughout the training session. According to adult learning principles, training participants will better understand and remember things they are taught if they can apply their own experiences and knowledge to it.

**Use Feedback to Maintain a Collaborative, Safe Environment:** If needed, provide nonjudgmental, specific feedback as you facilitate the session – focusing on describing a comment or behavior and its impact rather than speaking to the intent you infer from it. Don’t be hesitant to ask clarifying questions, if you don’t understand what a participant is sharing. At times, they also are trying to figure out what they are thinking or trying to say, so asking them some clarifying or probing questions may help them.

**Promote Active Engagement:** Encourage participants to be involved and take different roles in discussion that best suit their learning needs. Consider challenging participants to grapple with new terminology and ideas before answering their questions. Be sure to define what we mean by active participation and listening, as stated in the group agreements.

**Promote Balanced Participation:**  Open up conversations dominated by one individual to the larger group; create space for quieter individuals to participate if they would like to. You can often listen to one individual, and then call on another and ask them about what they think about what they heard or ask them pointedly a question. Small group activities or pair activities can be really useful in cases like this, and report backs can encourage participants to speak in front of the group. However, again it is important for the facilitator to “read the room” or more specifically, “read individuals.” You don’t want to put people on the spot, and this may turn people off from learning or put them on the defensive.

**Learn to be comfortable with silence or pauses:** As a facilitator, you may find “awkward” pauses or silences throughout the training, especially when asking a question to the group or facilitating a discussion. This may not always mean that the group is not engaged or do not have anything to share. Sometimes, people need a little more time to reflect and formulate what they want to say. Some may be hesitant to speak at first and may need a little more time to respond. You may ask the group for any last thoughts or questions, and if no one responds immediately, your first inclination is to just move on. However, it is ok to take 30 seconds or a minute to pause, let it be silent, and give the group a little time to speak up before you move on.

**Expand your knowledge of training content:** Speaking about structural factors requires that you as a facilitator have a base understanding of these structures. This does not mean that you need to have an expertise in all of history, policies and laws, and other structural factors. However, knowing the basics would be helpful for the training. In addition to the required readings, facilitators need to review Appendix O: Facilitator Preparation: Terms and Concepts, and it is encouraged to do your own research and exploration beyond what is provided to you in training modules and facilitators guide.

**Co-Facilitate:** If possible, co-facilitate this training with facilitators with backgrounds/training that may be different than your own. This can help to complement and expand the level of knowledge, experience, and perspectives of the facilitator. We recommend 2 or 3 facilitators for a 4-hour training.

**Don’t Compress:** This training is designed to run for at least four hours. It can be extended or split into sessions, but we discourage attempts to shorten it. As is, our training condenses a large amount of complex content. In our experience it is significantly less effective when truncated.
