# Supplementary material for: Structural Competency: Curriculum for Medical Students, Residents, and Interprofessional Teams on the Structural Factors That Produce Health Disparities
Source: MedEdPORTAL. 2020 Mar 13;16:10888. doi: 10.15766/mep_2374-8265.10888 (PMC7182045; doi:10.15766/mep_2374-8265.10888)
Supplement: Supplementary file 1 — A. Manual Background Info.docx B. Manual Intro.docx C. Manual Module 1.docx D. Manual Module 2.docx E. Manual Module 3.docx F. Manual Conclusion and Evaluation.docx G. Supplemental Reading List.docx H. Training Slides Intro.pptx I. Training Slides Module 1.pptx J. Training Slides Module 2.pptx K. Training Slides Module 3.pptx L. Participant Workbook.pdf M. Posttraining Survey.pdf N. Facilitator Guidelines.docx O. Facilitator Preparation - Terms and Concepts.docx P. Participant Sign-in Sheet.docx [file mep-16-10888-s001.zip › O. Facilitator Preparation - Terms and Concepts.docx]

STRUCTURAL COMPETENCY:

A Framework for Recognizing & Responding to Social, Political & Economic Structures to Improve Health


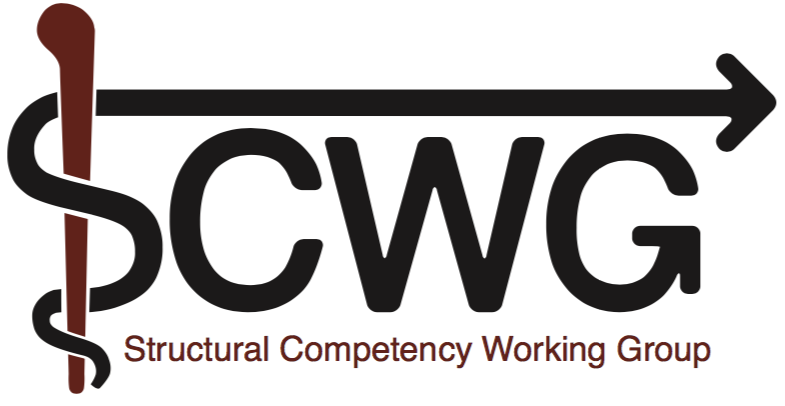


FACILITATOR PREPARATION: HELPFUL TERMS & CONCEPTS

Updated September 2018

Copyright:

The Structural Competency Working Group ([www.structcomp.org](http://www.structcomp.org)), in order to promote the widest possible dissemination of health curricula, has adopted an open copyright policy for its structural competency training materials. This means that we will grant permission to translate, adapt, or borrow our materials without charging fees or royalties under the following conditions:

- that you credit the Structural Competency Working Group for any borrowed/adapted Working Group materials and inform your audience about who we are and how to learn more about our organization ([structcomp.org](http://structcomp.org)). We suggest the following attribution (and request that it appear on any copyright page):
  - “*These materials have been [borrowed][adapted] from the Structural Competency Working Group,* [*www.structuralcompetency.org*](http://www.structuralcompetency.org)*; you can contact the Group at* [*structuralcompentency@gmail.com*](mailto:structuralcompentency@gmail.com)*”;*
- that your materials are distributed at no cost (or for your production cost only), that is, not-for-profit;
- that you allow others to reproduce/ adapt your edition or adaptation with no fees, royalties, etc. so long as they also do so at no cost or for production cost only, that is, not-for-profit;
- that you provide us with digital versions of your materials (including PDF and/or Microsoft Word files) and work with us so that we can host it on our website;
- that you send us your contact information so we can post it on our website and provide it to people who want to contact you about your edition, adaptation or publication;
- that you contact and stay in touch with the Structural Competency Working Group so that we can learn about your project and make sure you are using the most up-to-date materials.

If you decide to begin translating, adapting, or borrowing our materials, please use the most recently updated versions. Please contact us at [structuralcompetency@gmail.com](mailto:structuralcompetency@gmail.com) to find out if we are currently working on updating our materials, and if anyone else is working on a project similar to your own.

This training curriculum was prepared by Josh Neff, Seth M. Holmes, Kelly R. Knight, Shirley Strong, Ariana Thompson-Lastad, Cara McGuinness, Laura Duncan, Michael J. Harvey, Nimish Saxena, Katiana L. Carey-Simms, Alice Langford, Sara Minahan, Shannon Satterwhite, Lillian Walkover, Jorge De Avila, Brett Lewis, Gregory Chin, Jenifer Matthews, and Nick Nelson of the Structural Competency Working Group ([structcomp.org](http://structcomp.org/)), in collaboration with Sonia Lee and Caitlin Ruppel of Health Outreach Partners ([outreach-partners.org](http://outreach-partners.org/)).

The Structural Competency Working Group’s efforts have been supported by the Berkeley Center for Social Medicine and Deborah Lustig as well as the University of California Humanities Research Institute. Josh Neff’s work on this project has been supported by the UCSF Resource Allocation Program for Trainees (RAPtr), the Greater Good Science Center Hornaday Fellowship, UC Berkeley-UCSF Joint Medical Program Thesis Grant, and the Helen Marguerite Schoeneman Scholarship.

Facilitator Preparation: Helpful Terms and Concepts

A Note to the Facilitator

This list of terms and concepts was created to help prepare facilitators of structural competency trainings. It is meant specifically to accompany the structural competency training designed by the Structural Competency Working Group (SCWG) for delivery to a healthcare professional audience. The information contained in this document is meant to supplement the materials that the facilitator must be familiar with prior to the delivery of training, including the required facilitator reading, the curriculum, and the accompanying slide deck.

The following terms and concepts are organized in order as they appear in the structural competency curriculum and accompanying slide deck.

**Prevalence vs. Incidence**

- Definition 1: “Prevalence is a measure of the number of cases of a disease in a certain population for a specific period of time while incidence is a measure of the number of new cases of the disease.”
- Definition 2: “Prevalence is used to refer to how widespread a disease has become while incidence is used to refer to the rate at which the disease is manifested in a certain population.”
- Definition 3: Prevalence takes into consideration both the number of old and new cases of a disease as well as their duration while incidence only takes into consideration the number of new cases.”
- Definition 4: “Compared to prevalence, incidence is more reliable in determining the risk of a certain disease to a population.”

Source: *Difference Between Prevalence and Incidence*. Retrieved from http://www.differencebetween.net/science/health/difference-between-prevalence-and-incidence/#ixzz5GzQIoVYE

**Upstream Factors vs. Downstream Factors**

- Upstream determinants of public health are those overarching factors that are largely outside of the control of the individual and which have significant trickle down effects on other, more proximal, determinants of public health. Much the same way that pollution upstream of a river will have lasting and far reaching effects on those populations downstream, despite these populations having little to no control over this pollution.
- Downstream determinants of public health can be seen as the outcomes of up and midstream factors and variables. In some respects, downstream determinants are more easily mitigated or prevented by the individual, such as a change in eating habits or reducing risk of injury on the job.
- The following chart better illustrates the differences:

| Upstream | Midstream | Downstream |
| --- | --- | --- |
| Policies and programs   - Corporations and other businesses - Government agencies - Schools   Social inequities   - Class - Race/ethnicity - Gender - Immigration status - Sexual orientation | **Physical environment**   - Housing - Land use - Transportation - Residential segregation   **Behavior**   - Smoking - Nutrition - Physical activities - Violence | **Disease and injury**   - Infectious disease - Chronic disease - Injury   **Mortality**   - Infant mortality - Life expectancy |

- Source: *What are the definitions of upstream and downstream determinants of public health?* Retrieved from: https://www.quora.com/What-are-the-definitions-of-upstream-and-downstream-determinants-of-public-health.

**Colonialism and the Legacy of Colonialism in Mexico**

- Definition 1: “Colonialism is the policy of a polity seeking to extend or retain its authority over other people or territories, generally with the aim of developing or exploiting them to the benefit of the colonizing country and of helping the colonies modernize in terms defined by the colonizers, especially in economics, religion and health.”

Source: *Colonialism*. Retrieved from https://en.wikipedia.org/wiki/Colonialism

- Description: “‘Today,’ observes Collier, ‘Chiapas is almost an internal colony for the rest of Mexico, providing oil, electricity, timber, cattle, corn, sugar, coffee, and beans, but receiving very little in return.’ As our hosts in Moises Gandhi had explained, this internal colony had a long history, one they had not forgotten. When the Spanish arrived in the early sixteenth century, they sniffed out and played on longstanding local enmities in order to advance the conquest…. In part because of epidemics of imported disease, this first contact with the Spanish ended in defeat for the indigenous population, helpless against Europeans’ hunger for gold and domination. But in 1524 uprisings against the Spanish began, and in 1527 a group of indigenous people laid siege to the town of San Cristobal. They failed, of course, and the region’s history has since been notable for the almost ceaseless movements of its indigenous population. ‘Migration,’ notes historian John Womack, ‘is Chiapas’s oldest story.’”

Source: Paul Farmer, *Pathologies of Power,* Ch. 3: “Lessons from Chiapas.” 2003.

**Free Trade and the North American Free Trade Agreement (NAFTA)**

- Definition 1: “Free trade is a free market policy followed by some international markets in which countries' governments do not restrict imports from, or exports to, other countries. In government, free trade is predominately advocated by political parties that hold right-wing economic positions.”

Source: *Free Trade*. Retrieved from https://en.wikipedia.org/wiki/Free_trade#The_politics_of_free_trade

- Definition 2: “NAFTA stands for North American Free Trade Agreement. Signed in 1994 by President Bill Clinton, NAFTA eliminated all tariffs between the United States and Mexico within 15 years. While Canada and the United States had already been trading under the Canada-United States Free Trade Agreement, NAFTA went even further to eliminate trade barriers between the US and Canada.”

Source: *What is NAFTA? Definition, Effects & History*. Retrieved from https://study.com/academy/lesson/what-is-nafta-definition-effects-history.html

- Descriptive Example: “*In the heart of Mexico’s breadbasket — where plowing the land is still done the old fashioned way — farmers like Tirso Alvares Correa worry about their corn crops… ‘Corn from abroad is taking a toll on us,’ he says. ‘We can’t sell our corn anymore.’ Where are those products coming from? Up North. The United States and Canada have been selling tons of corn in Mexico for over a decade, thanks to NAFTA — the North American Free Trade Agreement. It eliminated tariffs on most agricultural trade and was supposed to be a win-win for the United States and Mexico. For Alvares Correa, NAFTA has been a disaster. While the trade agreement opened up U.S. markets to Mexican corn farmers, they haven’t been able to sell any corn in America. Meanwhile, American farmers have flooded Mexico with cheap corn thanks to generous U.S. government subsidies — subsidies left unchecked by NAFTA. A U.S. corn grower receives an average annual subsidy of $20,000 a year. The Mexican government gives their farmers just $100. Farmers said that entire towns are emptying because thousands of small farms have gone out of business. As many as 2 million farm workers have lost their jobs — the vast majority headed north across the U.S. border looking for better pay. No one questions that Mexico’s small corn farmers are hurting — due in large part to the impact of NAFTA.*

*‘Free trade has been a disaster for us,’ says Tirso Alvares Correa. But his worst fear is that soon there won’t be anyone left to work the land that has been in his family for generations.*”

Source: Clark, Amy (July 1, 2006) Is NAFTA Good for Mexico’s Farmers? *CBS News*. Retrieved from https://www.cbsnews.com/news/is-nafta-good-for-mexicos-farmers/

**Industrial Agriculture/Agribusiness**

- Description: “Today, the majority of American farmland is dominated by industrial agriculture—the system of chemically intensive food production developed in the decades after World War II, featuring enormous single-crop farms and animal production facilities. Back then, industrial agriculture was hailed as a technological triumph that would enable a skyrocketing world population to feed itself. Today, a growing chorus of agricultural experts—including farmers as well as scientists and policymakers—sees industrial agriculture as a dead end, a mistaken application to living systems of approaches better suited for making jet fighters and refrigerators. The impacts of industrial agriculture on the environment, public health, and rural communities make it an unsustainable way to grow our food over the long term. And better, science-based methods are available.”

Source: Union of Concerned Scientists. Industrial Agriculture: The outdated, unsustainable system that dominates U.S. food production. Union of Concerned Scientists. Retrieved from https://www.ucsusa.org/our-work/food-agriculture/our-failing-food-system/industrial-agriculture#.Ww6uf4iFM2w

**Racialized Low Wage Labor Market**

- Description: “Decades of racial progress have led some researchers and policymakers to doubt that discrimination remains an important cause of economic inequality. To study contemporary discrimination, we conducted a field experiment in the low-wage labor market of New York City, recruiting white, black, and Latino job applicants who were matched on demographic characteristics and interpersonal skills. These applicants were given equivalent résumés and sent to apply in tandem for hundreds of entry-level jobs. Our results show that black applicants were half as likely as equally qualified whites to receive a callback or job offer. In fact, black and Latino applicants with clean backgrounds fared no better than white applicants just released from prison. Additional qualitative evidence from our applicants’ experiences further illustrates the multiple points at which employment trajectories can be deflected by various forms of racial bias. These results point to the subtle yet systematic forms of discrimination that continue to shape employment opportunities for low-wage workers.”

Source: Pager, D., Western, B., & Bonikowski, B. (2009). Discrimination in a Low-Wage Labor Market: A Field Experiment. *American Sociological Review, 74(5): 777-799.* Retrieved from https://www.ncbi.nlm.nih.gov/pmc/articles/PMC2915472/

**Neoliberalism**

- Definition: “Neoliberalism or neo-liberalism refers primarily to the 20th-century resurgence of 19th-century ideas associated with laissez-faire economic liberalism. Those ideas include economic liberalization policies such as privatization, austerity, deregulation, free trade, and reductions in government spending in order to increase the role of the private sector in the economy and society. These market-based ideas and the policies they inspired constitute a paradigm shift away from the post-war Keynesian consensus which lasted from 1945 to 1980…. Modern advocates of free market policies avoid the term ‘neoliberal’ and some scholars have described the term as meaning different things to different people….

“When the term [neoliberalism] re-appeared in the 1980s in connection with Augusto Pinochet's economic reforms in Chile…, [it] had not only become a term with negative connotations employed principally by critics of market reform, but it also had shifted in meaning from a moderate form of liberalism to a more radical and laissez-faire capitalist set of ideas. Scholars now tended to associate it with the theories of Mont Pelerin Society economists Friedrich Hayek, Milton Friedman and James M. Buchanan, along with politicians and policy-makers such as Margaret Thatcher, Ronald Reagan and Alan Greenspan. Once the new meaning of neoliberalism became established as a common usage among Spanish-speaking scholars, it diffused into the English-language study of political economy. By 1994, with the passage of NAFTA and with the Zapatistas' reaction to this development in Chiapas, the term entered global circulation. Scholarship on the phenomenon of neoliberalism has been growing over the last couple of decades. The impact of the global 2008–2009 crisis has also given rise to new scholarship that criticizes neoliberalism and seeks policy alternatives.”

Source: Neoliberalism. Retrieved from https://en.wikipedia.org/wiki/Neoliberalism

**Gentrification**

- Definition: “Gentrification is the process of social, cultural, and economic transformation that is taking place in many centrally located urban neighborhoods which have experienced historic disinvestment. It involves significant increases in rental and for-sale housing costs, the in-migration of higher-income, white, and college- educated residents and the out-migration of longtime residents, many of whom may be renters, low-income residents, and people of color…. ‘The impacts of this process then include things like loss of social, cultural and community cohesion, displacement, loss of housing security, environmental degradation, commodification and appropriation of culture and other affects,’ said Dawn Phillips of CJJC, one of the report’s lead authors. Dr. Muntu Davis, director of the Alameda County Public Health Department, said: ‘Gentrification’s impacts on public health are not often included in the public debate or understood, but it has serious public health consequences for longtime residents who stay, those forced to leave, and our broader society. For public health practitioners, preventing displacement may be the single greatest challenge and the most important task in our efforts to create healthy communities for all.’”

Source: Neighborhood Funders Group (June 2, 2014). Report Released: Development Without Displacement – Resisting Gentrification in the Bay Area. Retrieved from http://www.nfg.org/report_released_development_without_displacement

**Intersectionality**

- Intersectionality, n. The interconnected nature of social categorizations such as race, class, and gender, regarded as creating overlapping and interdependent systems of discrimination or disadvantage; a theoretical approach based on such a premise. (Oxford Dictionary)
- “Intersectionality is a framework for conceptualizing a person, group of people, or social problem as affected by a number of discriminations and disadvantages. It takes into account people’s overlapping identities and experiences in order to understand the complexity of prejudices they face.

“In other words, intersectional theory asserts that people are often disadvantaged by multiple sources of oppression: their race, class, gender identity, sexual orientation, religion, and other identity markers. Intersectionality recognizes that identity markers (e.g. “female” and “black”) do not exist independently of each other, and that each informs the others, often creating a complex convergence of oppression. For instance, a black man and a white woman make $0.74 and $0.78 to a white man’s dollar, respectively. Black women, faced with multiple forms of oppression, only make $0.64. Understanding intersectionality is essential to combatting the interwoven prejudices people face in their daily lives.

“Kimberlé Crenshaw, law professor and social theorist, first coined the term intersectionality in her 1989 paper “Demarginalizing The Intersection Of Race And Sex: A Black Feminist Critique Of Antidiscrimination Doctrine, Feminist Theory And Antiracist Politics.” The theory emerged two decades earlier, however, when black feminists began to speak out about the white, middle-class nature of the mainstream feminist movement.”

Source: YW Boston. “What is intersectionality, and what does it have to do with me?” *YW Boston Blog.* March 29, 2017. Retrieved from: https://www.ywboston.org/2017/03/what-is-intersectionality-and-what-does-it-have-to-do-with-me/

Optional Video: [TED Talk by Kimberlé Crenshaw](https://www.ted.com/speakers/kimberle_crenshaw)

**Social Determinants of Health**

- Definition: “The complex, integrated, and overlapping social structures and economic systems that are responsible for most health inequities. These social structures and economic systems include the social environment, physical environment, health services, and structural and societal factors. Social determinants of health are shaped by the distribution of money, power, and resources throughout local communities, nations, and the world”
- For more (optional): Transcripts from interviews with the SDOH framers: [Michael Marmot](https://unnaturalcauses.org/assets/uploads/file/Interview%20with%20Marmot_SocialMedicine.pdf) and [Nancy Krieger](https://unnaturalcauses.org/assets/uploads/file/krieger_interview.pdf)

Source: Centers for Disease Control and Prevention (March 21, 2014) NCHHSTP Social Determinants of Health Definitions. Retrieved from https://www.cdc.gov/nchhstp/socialdeterminants/definitions.html
